# Transition‐Metal‐Free Reductive Hydroxymethylation of Isoquinolines

**DOI:** 10.1002/anie.201908857

**Published:** 2019-09-24

**Authors:** Benjamin M. Reeves, Hamish B. Hepburn, Alexandru Grozavu, Peter J. Lindsay‐Scott, Timothy J. Donohoe

**Affiliations:** ^1^ Department of Chemistry University of Oxford Chemistry Research Laboratory Mansfield Road Oxford OX1 3TA UK; ^2^ Eli Lilly and Company Erl Wood Manor Windlesham Surrey GU20 6PH UK

**Keywords:** formaldehyde, reductive-functionalization, tandem reaction, tetrahydroisoquinoline, transition-metal-free synthesis

## Abstract

A transition‐metal‐free reductive hydroxymethylation reaction has been developed, enabling the preparation of tetrahydroisoquinolines bearing C4‐quaternary centers from the corresponding isoquinolines. Deuterium labelling studies and control experiments enable a potential mechanism to be elucidated which features a key Cannizzaro‐type reduction followed by an Evans–Tishchenko reaction. When isoquinolines featuring a proton at the 4‐position are used, a tandem methylation‐hydroxymethylation occurs, leading to the formation of 2 new C−C bonds in one pot.

## Graphical abstract

**Reductive Functionalization**: The transition‐metal‐free synthesis of tetrahydroisoquinolines that bear a quaternary center through a reductive hydroxymethylation process is reported. It is a simple and robust method using cheap and accessible reagents to prepare complex heterocyclic motifs.

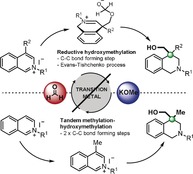

Saturated nitrogen‐containing heterocycles are found in a wide range of chemical structures such as natural products, pharmaceuticals and agrochemicals and are highly sought‐after structural motifs.^1^, ^2^ A key approach involving reduction of the corresponding aromatic heterocycle continues to be popular, due to the ready availability of starting materials, synthetic ease, and the lower step count in comparison to annulative chemistry. A multitude of different reaction systems have been reported to achieve arene reductions using catalytic Rh, Ir, and Pd, along with systems utilizing organic reducing agents.^3^, ^4^, ^5^, ^6^, ^7^ Despite these advances, most of these approaches only form C−H bonds and do not form any C−C bonds,^8^ precluding the ability to prepare saturated azacycles bearing quaternary centers (themselves an important class of azacycle that feature in a range of natural products and biologically active structures). To address this issue, we have recently reported an Ir‐catalyzed interrupted transfer hydrogenation reaction that results in the reduction of pyridines and quinolines (activated as the quaternary salt) along with the simultaneous installation of a CH_2_OH group in the 3‐position of the heterocycle.^9^ This dearomative functionalization leads to the direct formation of saturated azacycles bearing a hydroxymethyl group, a class of structures that the pharmaceutical industry has highlighted as an important, yet relatively underexplored, class of compound.^10^

Given the advantages of the new catalytic system, we were interested in subjecting other heterocycles to the dearomative functionalization reaction. Of particular interest was the preparation of tetrahydroisoquinolines (THIQs), and especially those containing a quaternary center in the 4‐position. These molecules are found in a range of natural product motifs (Scheme 1) and can be particularly challenging to prepare. Very few methodologies have been reported to access tetrahydroisoquinolines bearing a C‐4 quaternary center.^11^, ^12^ Furthermore, even less precedent exists for the preparation of the all‐carbon centers present in the natural products shown (Scheme 1).

**Scheme 1:**
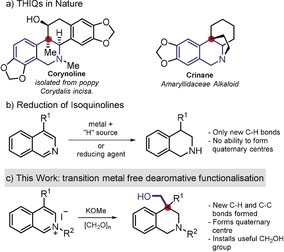
THIQs in Nature and current approaches to such motifs.

Initially, a model substrate, 4‐butyl‐*N*‐benzylisoquinolinium iodide **1 a**, was subjected to the previous optimal conditions for the dearomative functionalization of quinolinium salts: [IrCp*Cl_2_]_2_ (1 mol %), Mg(OMe)_2_ (0.75 equiv), paraformaldehyde (10 equiv), KI (2 equiv) in MeOH at 65 °C for 16 hours,^9^ Table 1 entry 1. However, upon analysis, the majority (92 %) of the product formed was undesired protonated product **3 a** with only a trace (3 %) of the desired hydroxymethylated THIQ **2 a**. A brief optimisation study showed that when the base was changed to NaOMe (3 equiv), KI was removed, and the paraformaldehyde content was increased, we formed product **2 a** in 49 %, with the rest of the mass consisting of multiple unidentified side products (Table 1, entry 2). However, as the optimization of this reaction unfolded, it was found to be capricious and the yield of product **2 a** fluctuated between runs, even when using identical conditions (for example, the conditions in Table 1, entry 2, gave a yield of **2 a** ranging from 10 % to 49 %). In an attempt to investigate the reason behind this irreproducibility, a control experiment was undertaken without any iridium catalyst, and we were surprised to find that these conditions furnished product **2 a** in a 30 % yield; no formation of **3 a** was observed, with unreacted **1 a**, as the only detectable side product, indicating that the transition metal catalyst is not required for this transformation, see below (Table 1, entry 3). Given the unsolved problems we encountered when using an iridium catalyst, and the potential advantages of a catalyst‐free system we decided to optimize the transition‐metal‐free conditions further.

**Table 1:** Reaction optimisation studies 
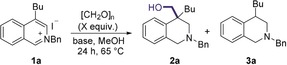

Entry	Base [equiv]	MeOH/[CH_2_O]_*n*_	Yield of **2 a** ^[a]^
1^[b]^	Mg(OMe)_2_ (0.75)	12:1	3 (**3 a** 92 %)
2^[c,d]^	NaOMe (3)	4:1	49 (see text)
3	NaOMe (3)	4:1	30
4	Mg(OMe)_2_ (1.5)	4:1	0
5	KOMe (3)	4:1	37
6^[d]^	KOMe (3)	4:1	38
7	Cs_2_CO_3_ (1.5)	4:1	44
8	KOMe (3.5)	3:1	42
9	KOMe (5)	2:1	49
10	KOMe (7.5)	1.5:1	61
11	**KOMe (10)**	**1:1**	**64^e^**
12	KOMe (12.5)	0.8:1	53
13	KOMe (10)	1.5:1	56
14	KOMe (10)	2:1	46

Performed on a 0.25 mmol scale in 1.25 mL of MeOH (≈30 mmol); [a] Determined by ^1^H NMR spectroscopy using trimethoxybenzene as a standard; [b] With [IrCp*Cl_2_]_2_ (1 mol %), KI (2 equiv); [c] With [Ir(Cp*Cl_2_]_2_ (1 mol %); [d] reaction run for 60 h; [e] isolated yield.

Changing the base back to Mg(OMe)_2_ resulted in no reaction, while KOMe (37 % after 24 h, 38 % after 60 h) and Cs_2_CO_3_ (44 % after 60 h) were also found to be suitable conditions (Table 1, entries 4–7). KOMe was chosen for further optimization due to its higher solubility in MeOH and low cost. Keeping the ratio of base to paraformaldehyde constant, while increasing the equivalents of base to 3.5 led to an increase in yield to 42 % (Table 1, entry 8).

Further increasing the quantity of KOMe (and paraformaldehyde) resulted in an increase in yield up to 64 % when 10 equivalents of base and a MeOH/paraformaldehyde ratio of 1:1 was used (Table 1, entries 9–11). Even higher base loadings were found to diminish the yield (53 %) (Table 1, entry 12). Attempts were made to decrease the paraformaldehyde loading, but a reduction was detrimental to the yield (Table 1, entries 13,14). The high loading of both KOMe and paraformaldehyde are not problematic as both reagents are inexpensive and readily available (Sigma Aldrich price^13^ KOMe: £0.02 per mmol, paraformaldehyde: £0.001 per mmol). Importantly, the transition‐metal‐free reaction does not suffer from reproducibility issues and was repeated reliably.

Following the development of these optimal conditions, the scope of the reductive functionalization was investigated (Scheme 2). A variety of alkyl groups in the 4‐position of the isoquinoline were tolerated well, giving the product in good yields, including a methyl (**2 b**), chains bearing aryl groups (**2 c**–**2 d**), and chains featuring an unprotected alcohol (**2 e**). Additionally, a variety of groups were tolerated on the heterocyclic nitrogen, including alkyl (**2 f**–**2 g**), electron rich benzylic groups (**2 h** + **2 j**) and alkyl groups featuring an unprotected alcohol (**2 i**) and ester functionality (**2 k**). Unfortunately, it was found that sterically encumbered substrates, such as those bearing isobutyl (**2 l**) and *p*‐tolyl (**2 m**) substituents at the C4‐position, and groups at the C‐3 position (**2 n**), were not suitable for the reaction and no desired product was formed.

**Scheme 2:**
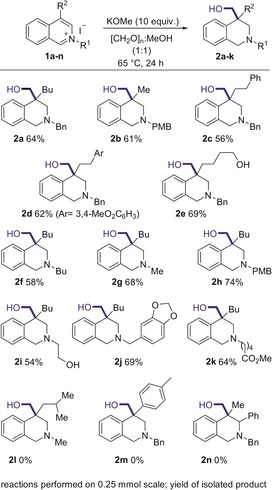
Scope of the reductive hydroxymethylation.

Interestingly, when a C4‐unsubstituted isoquinolinium salt was used in this methodology, the isolated product featured a quaternary center bearing a methyl group in addition to the CH_2_OH group, (cf. entry **2 b**, Scheme 2). This reaction has installed 2 new carbon‐carbon bonds in addition to 2 new carbon‐hydrogen bonds all in one‐pot. Substituents on the nitrogen and every position of the carbocyclic backbone could be altered, all leading to the formation of the C4‐methyl‐substituted hydroxymethylated product (**2 o**–**u**, Scheme 3). This transformation highlights the ability of this methodology to install complex functionality in only a single step from simple aromatic starting materials.

**Scheme 3:**
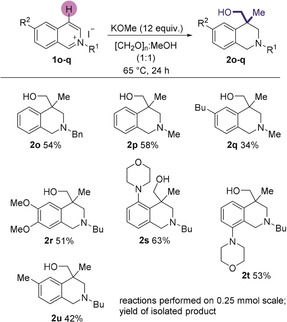
Methylation‐hydroxymethylation sequence.

Next, we attempted to understand the mechanism of both transformations. Firstly, we set out to ensure that these reactions were truly transition‐metal‐free because literature reports have indicated that reactions that appear transition‐metal‐free can actually be catalyzed by small quantities of metals trapped in laboratory equipment due to either degradation of the stirrer bar, or imperfections in the glassware.^14^ A control reaction was undertaken in a new flask using a new stirrer bar and gave the same result as entry 11 in Table 1. Some of the starting isoquinolines are prepared through a number of different pathways such as Suzuki (Pd) or Kumada (Ni) couplings, or are commercially available; they are then reacted with multiple alkyl iodides. However, all substrates behave similarly, suggesting that any systematic transition metal contamination of multiple substrates is unlikely. Treatment of isoquinolone **S1** and isoquinolinium **1 g** with a metal scavenger resin (Biotage Si‐TMT) gave material that behaved normally in the reductive hydroxymethylation reaction. Finally, inductively coupled plasma mass spectrometry (ICP‐MS) was performed on both the KOMe, and compounds **1 a**, **1 g**, and confirmed that transition metals were not present in any appreciable quantity.^15^

Having confirmed beyond reasonable doubt that the reaction was transition‐metal‐free, experiments were performed to elucidate the reaction pathway (Scheme 4). First, salt **1 a** was heated in methanol in the presence of methoxide but without formaldehyde (Reaction 1). Analysis of the reaction confirmed complete consumption of **1 a** and the formation of two products, amide **4 a** and enamine **5 a**. Amide **4 a** was isolated successfully and resubjection of **4 a** to the parent reaction conditions did not lead to any product formation, indicating that **4 a** is not an intermediate in the reaction pathway. Unfortunately, enamine **5 a** could not be isolated and was found to degrade upon work up or flash column chromatography. However, it was characterized through analysis of the crude ^1^H NMR spectra and was identified through comparison of similar compounds reported in the literature.^17^

**Scheme 4:**
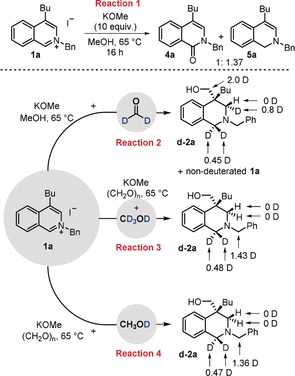
Mechanistic and deuterium labelling studies.

Initially, we suspected that the presence of **5 a** in this reaction meant that methoxide itself was a competent reductant of **1 a**, presumably via a Meerwein–Ponndorf–Verley type process. Note that methoxide has been reported to deliver hydride in such a fashion.^18^ However, labelling studies using CD_3_OH (with potassium *tert*‐butoxide added as a base) gave products **4 a** and **5 a** without any incorporation of deuterium, suggesting their formation occurs through a disproportionation reaction mediated by either methoxide or adventitious hydroxide addition to the arene. Next, a range of deuterium labelling studies were undertaken, with the resulting products analyzed by ^1^H NMR and ^2^H NMR spectroscopy. Substrate **1 a** was subjected to the optimized conditions with the appropriate deuterium‐labelled reagent being used, as indicated in Scheme 4. The use of paraformaldehyde‐d^2^ (Reaction 2) led to the formation of **d**‐**2 a** with full deuterium incorporation in the CH_2_OH group, confirming paraformaldehyde, not oxidized MeOH, is the source of the CH_2_OH. Deuterium was also incorporated into the C1‐position of the THIQ, with no diastereoselectivity (0.45D v 0.45D). Intriguingly, highly diastereoselective deuterium incorporation at the C3‐position was observed (0.8D v 0.0D). NOESY analysis of the parent compound **2 a** confirmed that the deuterium incorporation occurred exclusively from the same face as the CH_2_OH group. The reaction using paraformaldehyde‐d^2^ was particularly slow and allowed the re‐isolation of the starting material **1 a**; analysis determined no deuterium incorporation indicating that it is unlikely that the reduction is reversible under the reaction conditions.

The corresponding reaction using paraformaldehyde and [D_4_]MeOH was then undertaken (Reaction 3, Scheme 4) and analysis of the product confirmed no deuterium incorporation at either the C3‐position or the CH_2_OH. However, deuterium incorporation (1.43D) was observed on the *N*‐benzyl group—this is to be expected, as this position is acidic in **1 a**. Notably, considerable deuterium incorporation (0.48D at each position) was observed at C1. A further experiment was undertaken using CH_3_OD (Reaction 4, Scheme 4), which generated **2 a** with significant D incorporation at both the *N*‐benzyl (1.36D) and C1‐positions (0.94D).

Following the deuterium labelling studies, our proposed mechanism is as follows (Scheme 5): addition of methoxide to formaldehyde leads to a hemi‐acetal which can deliver a hydride to the C1‐position of the heterocycle in a Cannizzaro‐type reduction.^19^ The absence of **4 a** in the crude reaction mixtures of the reactions shown in Scheme 2 and Scheme 3 and the relatively high level of C1 deuterium incorporation when using paraformaldehyde‐d^2^ indicate that any disproportionation pathway is not operative under the main reaction conditions. We suggest that the relatively high deuterium incorporation at the C1‐position when using [D_4_]MeOH is due to the reversible deprotonation/reprotonation of an allylic ylide as supported by the reaction using CH_3_OD. Following the reduction, newly formed enamine **5 a** attacks formaldehyde to form zwitterionic intermediate **6 a**. The lack of (D) diastereoselectivity at this C1‐position is consistent with the enamine reating with formaldehyde from either face. Due to the high (D) diastereoselectivity observed at the C3‐position, we propose that delivery of the second hydride is an intramolecular process via hemi‐acetal **7 a**, which transfers hydride in an intramolecular fashion through an Evans–Tishchenko process.^20^ This intramolecular hydride delivery results in the reduction of the iminium ion from the same face as the CH_2_O group, consistent with the observed deuterium incorporation and furnishes **2 a** after ester cleavage in situ. If this mechanistic postulation is correct, formate by‐products should be formed during the process and ^1^H NMR analysis of the crude reaction mixture from **1 a→2 a** revealed considerable quantities of potassium formate^21^ indicating that formaldehyde is indeed oxidized during the reaction.

**Scheme 5:**
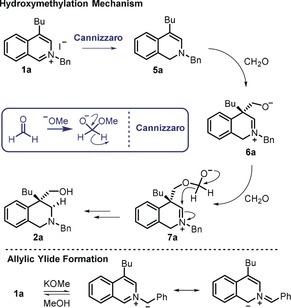
Proposed mechanism of hydroxymethylation.

The Supporting Information contains details of deuterium labelling experiments designed to understand the tandem methylation‐hydroxymethylation process. The conclusion from these experiments is that the reaction proceeds via the C‐4 methyl isoquinolinium salt formed in situ; and that the methyl group itself originates from formaldehyde after reaction with an enamine, loss of water and tautomerization.

In conclusion we have reported a highly useful reductive hydroxymethylation of C‐4 substituted isoquinolinium salts leading to the formation of THIQs bearing a quaternary center. This process is transition‐metal‐free and prepares complex heterocyclic motifs utilizing cheap and readily accessible reagents. Furthermore, if isoquinolinium salts featuring a hydrogen in the C4 position are utilized, a tandem methylation‐hydroxymethylation sequence occurs, forming two new C−C bonds and delivering THIQs bearing quaternary centers. Mechanistic experiments and deuterium labelling studies helped propose a feasible mechanism for both reactions, which includes a Cannizzaro‐type isoquinolinium salt. This is followed by a carbon–carbon bond forming step which initiates an intramolecular Evans–Tishchenko process to furnish the product.

## Conflict of interest

The authors declare no conflict of interest.

## Supporting information

As a service to our authors and readers, this journal provides supporting information supplied by the authors. Such materials are peer reviewed and may be re‐organized for online delivery, but are not copy‐edited or typeset. Technical support issues arising from supporting information (other than missing files) should be addressed to the authors.

Supplementary
