# Supplementary material for: Transition‐Metal‐Free Reductive Hydroxymethylation of Isoquinolines
Source: Angew Chem Int Ed Engl. 2019 Sep 24;58(44):15697–701. doi: 10.1002/anie.201908857 (PMC6856840; doi:10.1002/anie.201908857)
Supplement: Supplementary file 1 — Supplementary [file ANIE-58-15697-s001.pdf]

Angewandte Chemie
Eine Zeitschrift der Gesellschaft Deutscher Chemiker

Supporting Information

Transition-Metal-Free Reductive Hydroxymethylation of Isoquinolines

*Benjamin M. Reeves, Hamish B. Hepburn, Alexandru Grozavu, Peter J. Lindsay-Scott, and Timothy J. Donohoe**

anie_201908857_sm_miscellaneous_information.pdf

Contents

Experimental 3

Synthesis of isoquinolines 4

Synthesis of isoquinolinium iodides 10

Transition metal free reductive hydroxymethylation 19

Tandem methylation-hydroxymethylation 25

Mechanistic Experiments 29

Deuterium Labelling Studies 33

Deuterium NMR Spectra 35

NMR Spectra 40

Experimental

Unless stated otherwise, all chemicals were purchased from commercial suppliers (Sigma-Aldrich, Fluorochem, Alfa Aesar) and used without further purification. ^{1}H and ^{13}C spectra were recorded on a Bruker AVIII400 Spectrometer (400 MHz, and 101 MHz respectively), in $CDCl_3$, DMSO-d6 or MeOD-d4, and referenced to residual solvent peaks. ^{2}H spectra were recorded on a Bruker AVII500 (^{2}H: 500 MHz) in $CHCl_3$, referenced to trace $CDCl_3$ solvent peaks. Chemical shifts (δ) are quoted in parts per million (ppm) to the nearest 0.01 for ^{1}H and 0.1 for ^{13}C, coupling constants J are quoted in Hz to the nearest 0.1 and splitting are recorded as singlet (s), doublet (d), triplet (t), quartet (q), pentet (p), heptet (h), and multiplet (m). Assignments were based upon COSY, DEPT, HSQC, HMBC, and NOESY experiments. Chemical shifts and splitting patterns are recorded as observed. ^{1}H NMR yields were determined using a known quantity of trimethoxybenzene internal standard, which was added to the crude reaction mixture following evaporation under reduced pressure. Quantitative ^{1}H NMR experiments were run on a Bruker AVIII400 Spectrometer using a 25 second relaxation time between scans. The singlet at 6.08 ppm, allowed quantitative integration of known product peaks. Infrared spectra were recorded on a Bruker Tensor 27 FT-IR spectrometer fitted with an Attenuated Total Reflectance (ATR) sampling accessory. Absorption maxima are quoted in wavenumbers (cm^{-1}). Mass spectra were recorded on a Fisons Platform II spectrometer under electrospray ionisation (ESI). High resolution mass spectra are given to four decimal places and were recorded on a Bruker MicroTof (resolution = 10000 FWHM). Melting points (m.p.) were obtained using a Lecia VMGT heated-stage microscope. Analytical thin layer chromatography was performed on pre-coated silica gel aluminium sheets from Merck (TLC Silica Gel 60 F254s). Spots were visualized either by the quenching of UV fluorescence, and by staining with potassium permanganate or phosphomolybdic acid solution. All chiral compounds are racemic mixtures. ICP-OES analysis of the isoquinoliniums were conducted on an Agilent 5100/5110 SVDV instrument with a Burgener T-2100 polymeric parallel path nebulizer.

Synthesis of isoquinolines

General Procedure A: A solution of 4-bromoisoquinoline (1 equiv.), alkyl boronic acid (2 equiv.), potassium phosphate (4 equiv.), and SPhos (1 mol%) in toluene (10 mL per mmol) was sparged with argon for two minutes. Then Pd_2dba_3 (0.5 mol%) was added to this solution and the reaction mixture was heated at reflux for 12 h under a balloon of argon. The reaction was quenched with distilled water (10 mL) and extracted with EtOAc (20 mL x 3). The organic layers were combined and dried over anhydrous sodium sulphate, filtered and evaporated under reduced pressure. The crude material was purified by chromatography to give the pure product.

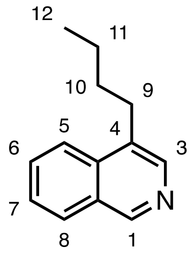

4-Butylisoquinoline (S1) The title compound was prepared according to General Procedure A using butyl boronic acid (0.98 g, 9.62 mmol), and purified by silica gel chromatography (10-30% EtOAc in pentane) to give **S1** as a yellow oil (2.041 g, 99%). ^{1}H NMR: ($CDCl_3$) δ 9.12 (s, 1H), 8.37 (s, 1H), 8.05 – 7.94 (m, 2H), 7.73 (ddd, *J* = 8.4, 6.9, 1.4 Hz, 1H), 7.60 (ddd, *J* = 8.1, 6.9, 1.1 Hz, 1H), 3.07 – 2.96 (m, 2H), 1.73 (tt, *J* = 7.8, 6.6 Hz, 2H), 1.46 (h, *J* = 7.4 Hz, 2H), 0.97 (t, *J* = 7.3 Hz, 3H) which is consistent with spectroscopic data reported previously.[1]

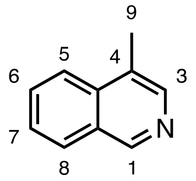

4-Methylisoquinoline (S2) To a solution of 4-bromoisoquinoline (1.00 g, 4.81 mmol) in THF (30 mL) under argon was added $Ni(PPh)_3Cl_2$ (26 mg, 1 mol%) and methylmagenisum bromide (1.92 mL, 5.77 mmol, 3 M in Et_2O). The solution was stirred at room temperature for 18 hours then acidified to pH 1-3 with 1M HCL and extracted with EtOAc (3 x 30 mL). The organic layers were combined, dried (Na_2SO_4), and concentrated *in vacuo*. The crude material was purified by silica gel chromatography (20% EtOAc in pentane) to give *isoquinoline* **S2** (0.35 g, 50%) as a yellow oil. ^{1}H NMR: ($CDCl_3$) δ 9.12 (s, 1H), 8.38 (d, *J* = 1.0 Hz, 1H), 8.09 – 7.89 (m, 2H), 7.74 (ddd, *J* = 8.5, 6.9, 1.4 Hz, 1H), 7.61 (ddd, *J* = 8.0, 6.8, 1.3 Hz, 1H), 2.63 (t, *J* = 0.8 Hz, 3H) which is consistent with the literature.[1]

Alternatively:

The title compound was prepared according to General Procedure A using methyl boronic acid (0.576 mg, 9.62 mmol), and purified by silica gel chromatography (10-30% EtOAc in pentane) to give **S2** as a yellow oil (0.570 g, 81%).

[1] D. Chen, G. Xu, Q. Zhou, L. W. Chung, W. Tang, *J. Am. Chem. Soc.* **2017**, *139*, 9767-9770

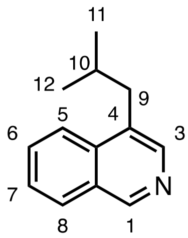

4-(*iso*-Butyl)isoquinoline (S3) The title compound was prepared according to General Procedure A using *iso*-butyl boronic acid (0.816 g, 8.00 mmol) to give **S3** as a yellow oil (0.758 g, 99%). This compound was used in the next reaction without further purification. ^{1}H NMR ($CDCl_3$) δ 9.09 (s, 1H), 8.31 (s, 1H), 7.98 – 7.85 (m, 2H), 7.66 (ddd, *J* = 8.4, 6.8, 1.4 Hz, 1H), 7.53 (ddd, *J* = 8.0, 6.8, 1.2 Hz, 1H), 2.82 (d, *J* = 7.2 Hz, 2H), 2.00 (dt, *J* = 13.5, 6.7 Hz, 1H), 0.94 (d, *J* = 6.6 Hz, 6H).

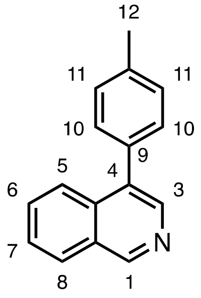

4-Tolylisoquinoline (S4) A solution of 4-bromoisoquinoline (1 equiv.), 4-(*p*-tolyl)-boronic acid (1.2 equiv.), triphenylphosphine (10 mol%), and potassium carbonate (2.7 equiv.) in DME/ H_2O (25:5 mL) was sparged with argon. $Pd(OAc)_2$ (2.5 mol%) was added to the this solution, and the reaction mixture was heated at reflux for 18 h under a balloon of argon. The reaction was quenched with distilled water (10 mL) and extracted with EtOAc (20 mL x 3). The organic layer was dried over anhydrous sodium sulphate, filtered and evaporated under reduced pressure. The crude compound was purified by silica gel chromatography (10-30% EtOAc in pentane) to give **S4** (1.05 g, 99%) as a red oil. ^{1}H NMR ($CDCl_3$) δ 9.24 (d, *J* = 0.9 Hz, 1H), 8.48 (s, 1H), 8.13 – 7.99 (m, 1H), 7.94 (dd, *J* = 8.5, 1.2 Hz, 1H), 7.73 – 7.58 (m, 2H), 7.42 (d, *J* = 8.1 Hz, 2H), 7.37 – 7.31 (m, 2H), 2.47 (s, 3H) which is consistent with spectroscopic data reported previously.[2]

General Procedure B: A solution of 4-bromoisoquinoline (1 equiv.), the appropriate alkyne (1.1 equiv.), copper(I) iodide (6 mol%), triethylamine (10 equiv.), triphenylphosphine (6 mol%), and $Pd(OAc)_2$ (3 mol%) in MeCN (3 mL per mmol) was prepared under an argon atmosphere, and heated at reflux for 40 h. The reaction was quenched with aqueous sodium carbonate (10 mL) and extracted in CH_2Cl_2 (20 mL x 3). The combined organic layers were dried over anhydrous sodium sulphate, filtered and evaporated under reduced pressure to give the crude compound, which was taken through to the next step without further purification. A solution of 10% Pd/C (20% w/w) in methanol was added to the crude product. The solution was sparged with hydrogen and the reaction mixture was stirred rigorously under a hydrogen atmosphere for 12 h, to give the product after purification by chromatography.

[2] V. K. Tiwari, G. G. Pawar, H. S. Jena, M. Kapor, *Chem. Commun.* **2014**, *50*, 7322-7325.

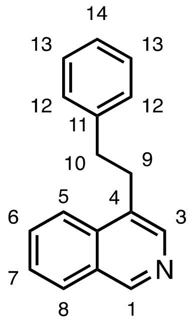

4-Phenethylisoquinoline (S5) The title compound was prepared according to General Procedure B using phenylacetylene (0.6 mL, 5.5mmol) and purified by silica gel chromatography (10-20% EtOAc in pentane) to give substrate **S5** (0.534 g, 46 %) as a red oil. ^{1}H NMR (CDCl$_3$) δ 9.14 (s, 1H, C^{1}**H**), 8.34 (s, 1 H, C^{3}**H**), 8.02 (ddt, *J* = 13.2, 8.1, 1.0 Hz, 2H, C^{5+8}**H**), 7.75 (ddd, *J* = 8.4, 6.8, 1.4 Hz, C$^{6/7}$**H**), 7.62 (ddd, *J* = 8.1, 6.9, 1.1 Hz, 1H, C$^{6/7}$**H**), 7.34 – 7.27 (m, 2H, 2 x Ar**H**), 7.25 – 7.19 (m, 3H, 3 x Ar**H**), 3.39 – 3.24 (m, 2H, 2 x C^{9}**H**), 3.13 – 2.92 (m, 2H, 2 x C^{10}**H**); ^{13}C NMR (CDCl$_3$) δ 151.6 (C^{1}), 142.9 (C^{3}), 141.4 (C^{Q}), 134.7 (C^{Q}), 130.8 (C^{Q}), 128.7 (3 x C^{Ar}), 128.6 (C^{Q}), 128.5 (2 x C^{Ar}), 128.5 (C$^{5/8}$), 127.0 (C$^{6/7}$), 127.0 (C$^{6/7}$), 122.9 (C$^{5/8}$), 37.0 (C^{10}), 32.3 (C^{9}). HRMS (ESI): Exact mass calculated for $C_{17}H_{16}N$ [M+H]$^{+}$ = 234.1279, found: 234.1277; IR (neat) (cm^{-1}): 3124, 2986, 1702, 1681, 1615, 1577, 1562, 1512, 1466, 630.

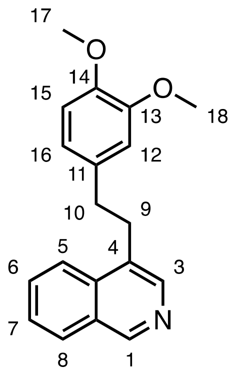

4-(3,4-Dimethoxyphenethyl)isoquinoline (S6) The title compound was prepared according to General Procedure B using 4-ethynyl-1,2-dimethoxybenzene (0.892 g, 5.5 mmol) and purified by silica gel chromatography (10-30% EtOAc in pentane) to give substrate **S6** (0.714 g, 49%) as a red oil. ^{1}H NMR (CDCl$_3$) δ 9.13 (s, 1H, C^{1}**H**), 8.33 (s, 1H, C^{3}**H**), 8.00 (ddt, *J* = 7.2, 4.5, 1.0 Hz, 2H, C^{5+8}**H**), 7.73 (ddd, *J* = 8.3, 6.9, 1.3 Hz, 1H, C$^{6/7}$**H**), 7.61 (ddd, *J* = 8.1, 6.8, 1.0 Hz, 1H, C$^{6/7}$**H**), 6.80 (d, *J* = 8.1 Hz, 1H, C^{15}**H**), 6.74 (dd, J= 8.1, 2.0 Hz, 1H, C^{16}**H**), 6.66 (d, *J* = 2.0 Hz, 1H, C^{12}**H**), 3.86 (s, 3H, 3 x C$^{17/18}$**H**), 3.81 (s, 3H, 3 x C$^{17/18}$**H)**, 3.34 – 3.25 (m, 2H, 2 x C^{9}**H**), 3.03 – 2.96 (m, 2H, 2 x C^{10}**H**); ^{13}C NMR (CDCl$_3$) δ 151.8 (C^{1}), 149.2 (C^{Q}), 147.9 (C^{Q}), 143.2 (C^{3}), 135.1 (C^{Q}), 134.3 (C^{Q}), 131.1 (C^{Q}), 130.7 (C$^{6/7}$), 128.8 (C^{Q}), 128.8 (C$^{5/8}$), 127.3 (C$^{6/7}$), 123.2 (C$^{5/8}$), 120.6 (C^{12}), 112.2 (C^{12}), 111.7 (C^{15}), 56.4 (C$^{17/18}$), 56.3 (C$^{17/18}$), 36.9 (C^{10}), 32.7 (C^{9}); HRMS (ESI): Exact mass calculated for $C_{19}H_{20}NO_2$ [M+H]$^{+}$ = 294.1494, found: 294.1487. IR (neat) cm^{-1}: 3119, 3001, 2258, 2152, 2034, 1620, 1536, 1260, 1251, 1005.

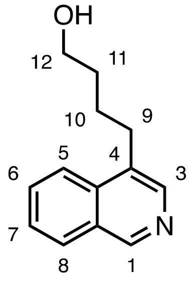

4-(Butan-1-ol)isoquinoline (S7) The title compound was prepared according to General Procedure B using 3-butyn-1-ol (0.45 mL, 6 mmol) to give substrate **S7** (0.875 g, 87%) as a red oil. This compound was used in the next reaction without further purification. ^{1}H NMR (CDCl$_3$) δ 9.11 (s, 1H, C^{1}**H**), 8.37 (s, 1H, C^{3}**H**), 8.02 – 7.95 (m, 2H, C^{5+8}**H**), 7.73 (ddd, *J* = 8.4, 6.8, 1.4 Hz, 1H C$^{6/7}$**H**), 7.60 (ddd, *J* = 8.1, 6.8, 1.1 Hz, 1H, C$^{6/7}$**H**), 3.71 (t, *J* = 6.4 Hz, 2H, 2 x C^{12}**H**), 3.10 – 2.98 (m, 2H, 2 x C^{9}**H**), 1.89 – 1.80 (m, 2H, 2 x C^{10}**H**), 1.76 – 1.67 (m, 2H, 2 x C^{11}**H**), 1.63 (s, 1H, O**H**).

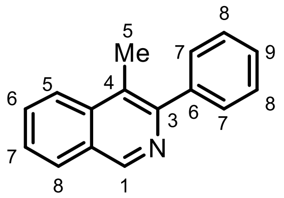

3-Phenyl-4-methylisoquinoline (S8) The title compound was prepared according to the procedure of Donohoe.[3]

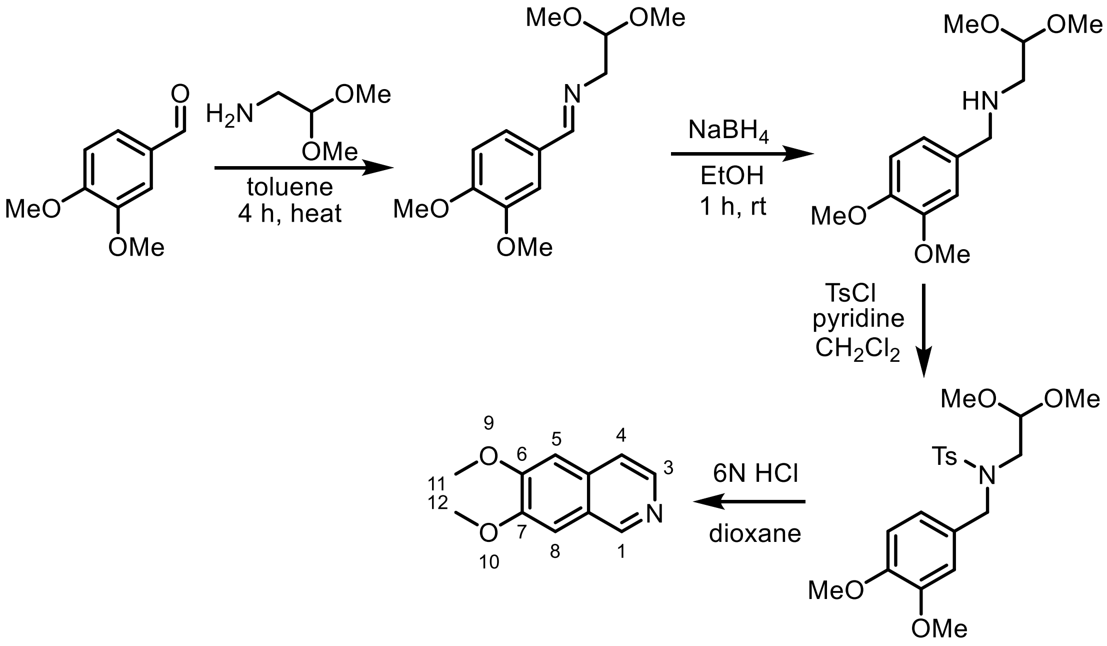

6,7-Dimethoxyquinoline (S9) The title compound was prepared from 3,4-dimethoxybenzaldehyde according a modified procedure of the one reported by Knochel.[4] 3,4-Dimethoxybenzaldehyde (4.15 g, 25 mmol) and aminoacetaldehyde dimethylacetal (4.1 mL, 37.5 mmol) were heated at reflux in toluene (75 mL) using Dean-Stark apparatus. After the appropriate volume of water (ca. 4.5 mL) was collected in the Dean-Stark apparatus (approximately 4 hours) the reaction was cooled to room temperature and concentrated in vacuo. Note: the imine appeared to be unstable on TLC, and as such this reaction cannot be monitored by TLC. The material was dissolved in CH2Cl2 and dried (MgSO4), filtered, and concentrated in vacuo to give imine which was carried on without further purification.

Imine (assume 25 mmol) was dissolved in EtOH (25 mL) and sodium borohydride (1.9 g, 50 mmol) was added portionwise. After 1 hour, TLC analysis indicated complete conversion and the reaction was concentrated in vacuo. The crude material was dissolved in H2O (50 mL) and CH2Cl (50 mL) and separated. The aqueous layer was further extracted with CH2Cl2 (2 x 50 mL). The organic layers were

[3] T. J. Donohoe, B. S. Pilgrim, G. R. Jones, J. A. Bassuto, *Proc. Nat. Acad. Sci.* **2012**, *109*, 11605-11608.

combined, dried ($MgSO4$), filtered, and concentrated in vacuo to give pure amine (4.45 g, 70% over 2 steps) as a colourless oil.

The amine (4.45 g, 17.5 mmol) was dissolved in $CH2Cl2$ (50 mL) and pyridine (2.1 mL) and cooled to 0 °C. Tosyl chloride (4.4 g, 22.75 mmol) was added portionwise and the reaction was stirred at room temperature for 14 hours. The reaction was quenched with saturated $NaHCO3$ solution (50 mL) and extracted with $CH2Cl2$ (3 x 50 mL). The organic layers were combined, dried ($MgSO4$), filtered, and concentrated in vacuo. The crude material was purified by FCC (10-40% EtOAc in pentane) to give sulfonamide (5.56 g, 78%) as a yellow oil.

The sulfonamide (4.07 g, 10 mmol) was dissolved in dioxane (90 mL) and 6N HClaq (7 mL) was added. The solution was heated at reflux for 5 hours, cooled, and washed with $Et2O$ (100 mL). The aqueous layer was made basic with NaOHaq and extracted with $CH2Cl2$. The organic layers were combined, dried ($MgSO4$), filtered and concentrated in vacuo. The crude material was purified by FCC (0-4% MeOH in $CH2Cl2$) to give isoquinoline **S9** (1.63 g, 86%) as a yellow oil which solidified on standing (ca. 1 hour). For each step, the spectroscopic data matched those previously reported by Knochel.[4]

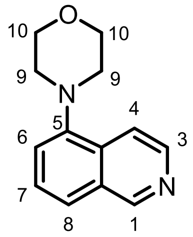

5-Morpholinoisoquinoline (S10) The title compound was prepared from 5-bromoisoquinoline according to the procedure of Novartis.[5]

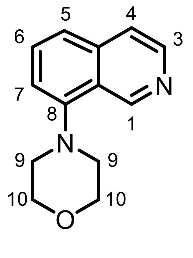

8-Morpholinoisoquinoline (S11) To a three-necked flask fitted with a condenser was added 8-bromoisoquinoline (2.08 g, 10 mmol), cesium carbonate (6.5 g, 20 mmol), *rac*-BINAP (311 mg, 5 mol%), morpholine (1.75 mL, 20 mmol), and toluene 80 mL. The solution was sparged with argon for 10 minutes before palladium acetate (110 mg, 5 mol%) was added and the solution heated at reflux for 14 hours. The solution as cooled to room temperature and diluted with 100 mL EtOAc and 100 mL water. The biphasic solution was separated, and the aqueous layer was further extracted with EtOAc (2 x 50 mL). The organic layers were combined, dried ($MgSO_4$), and concentrated *in vacuo*. The crude material was purified by FCC (0-30% EtOAc in pentane) to yield *isoquionline* **X** (1.60 g, 75%) as an orange oil that solidified on standing (ca. 2 hours). ^{1}H NMR ($CDCl_3$) δ 9.61 (1H, s, C^1**H**), 8.52 (1H, d, *J* = 5.7 Hz, C^3**H**), 7.65-7.58 (2H, m, C^4**H** + C^6**H**), 7.54-7.48 (1H, m, C^5**H**), 7.17-7.11 (1H, m, C^7**H**), 4.03-3.97 (4H, m, 2 x C^9**H**$_2$), 3.21-3.15 (4H, m, 2 x C^{10}**H**$_2$); ^{13}C NMR ($CDCl_3$) δ 148.8 (C^1), 143.0 (C^Q), 137.4 (C^Q)¸ 130.7 (C^3),

---

[4] A. Metzger, M. A. Schade, P. Knochel, *Org. Lett.* **2008**, *10*, 1107-1110

[5] D. Behnke, D. Carcache, P. Ertl, M. Koller, D. Orain, *US Patent* US2014/57902, **2014**, 754-755.

123.5 (C^5), 121.8 (C^4), 120.7 ($C^Q + C^6$), 115.6 (C^7), 67.2 (2 x C^9), 53.8 (2 x C^{10}); HRMS (ESI): Exact mass calculated for $C_{13}H_{15}NO_2$ $[M+H]^+$: 215.11789, found: 215.11795; IR (neat) (cm^{-1}) 1616, 1566, 1521, 1494, 1393, 1232, 1113, 990, 847, 754.

Synthesis of isoquinolinium iodides

General Procedure C: A mixture of the appropriate isoquinoline (1 equiv.) and aryl halide (1.2 equiv.) in acetone (5 mL per mmol) was stirred in the dark at room temperature for 16 hours. The reaction mixture was filtered under reduced pressure. The resulting solid was washed with ether (50 mL) then dried under vacuum for 10 minutes to give the *N*-Ar-isoquinolinium iodide salts as solids.

General Procedure D: A mixture of the appropriate isoquinoline (1 equiv.) and methyl iodide (2 equiv.) in CH_2Cl_2 (2.5 mL per mmol) was heated at 40 °C for 16 hours. The reaction mixture was filtered under reduced pressure. The resulting solid was washed with ether (50 mL) then dried under vacuum for 10 minutes to give the *N*-methyl-isoquinolinium iodide salts as solids.

General Procedure E: A mixture of the appropriate isoquinoline (1 equiv.) and alkyl iodide (2 equiv.) in 1,4-dioxane (2.5 mL per mmol) was heated at 90 °C for 16 hours. The reaction mixture was filtered under reduced pressure. The resulting solid was washed with ether (50 mL) then dried under vacuum for 10 minutes to give the *N*-alkyl-isoquinolinium iodide salts as solids.

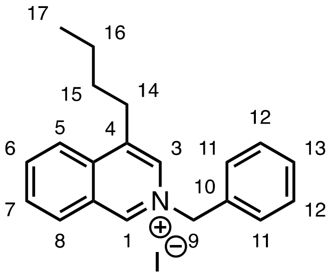

***N*-Benzyl-4-butylisoquinolinium iodide (1a)** The title compound was prepared according to General Procedure C using 4-butylisoquinoline (0.300 g, 1.62 mmol) and benzyl iodide (0.24 mL, 1.94 mmol) to give salt **1a** (0.652 g, 99 %) as a yellow solid. ^{1}H NMR ((CD_3)$_2$SO) δ 10.14 (s, 1H, C^1**H**), 8.77 (s, 1H, C^3**H**), 8.53 (d, *J* = 8.18 Hz, 1H, C$^{5/8}$**H**), 8.45 (d, *J* = 8.51 Hz, 1H, C$^{5/8}$**H**), 8.29 (ddd, *J* = 8.37, 7.01, 1.29 Hz, 1H, C$^{6/7}$**H**), 8.09 (t, *J* = 7.58 Hz, 1H, C$^{6/7}$**H**), 7.63 – 7.55 (m, 2H, 2 x Ar**H**), 7.49 – 7.38 (m, 3H, 3 x Ar**H**), 5.93 (s, 2H, 2 x C^9**H**), 3.18 (t, *J* = 7.7 Hz, 2H, 2 x C^{14}**H**), 1.66 (p, *J* = 7.60, 2H, 2 x C^{15}**H**), 1.38 (h, *J* = 7.36, 2H, 2 x C^{16}**H**), 0.91 (t, *J* = 7.34, 3H, 3 x C^{17}**H**); ^{13}C NMR ((CD_3)$_2$SO) δ 148.3 (C^1), 139.2 (C^Q), 137.1 (C$^{6/7}$), 136.1 (C^Q), 134.4 (C^Q), 133.3 (C^3), 131.4 (C$^{5/8}$), 131.1 (C$^{6/7}$), 129.3 (C^{Ar}), 129.2 (2 x C^{Ar}), 128.7 (2 x C^{Ar}), 127.4 (C^Q), 124.2 (C$^{5/8}$), 63.3 (C^9), 31.7 (C^{15}), 28.8 C^{14}), 21.9 (C^{16}), 13.7 (C^{17}); HRMS (ESI): Exact mass calculated for $C_{20}H_{22}N$ [M]$^+$: 276.17468, found: 276.17462; m.p. (acetone) 146-148 °C; IR (neat) (cm^{-1}): 3081, 2511, 1784, 1699, 1456, 1250, 848, 835, 724, 677.

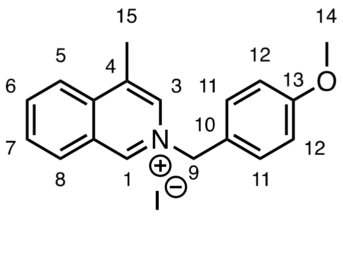

***N*-(*p*-Methoxy)benzyl-4-methylisoquinolinium iodide (1b)** The title compound was prepared according to General Procedure C using 4-methylisoquinoline (0.285 g, 1.96 mmol) and *p*-methoxybenzyl iodide (0.34 mL, 2.35 mmol) to give salt **1b** (0.568 g, 99 %) as a yellow solid. ^{1}H NMR ((CD_3)$_2$SO) δ 10.11 (s, 1H, C^1**H**), 8.75 (s, 1H, C^3**H**), 8.52 (d, *J* = 8.3

Hz, 1H, $C^{5/8}$**H**), 8.36 (d, *J* = 8.4 Hz, 1H, $C^{5/8}$**H**), 8.29 (ddd, *J* = 8.4, 6.8, 1.3 Hz, 1H, $C^{6/7}$**H**), 8.09 (ddd, *J* = 8.2, 6.8, 1.2 Hz, 1H, $C^{6/7}$**H**), 7.58 (d, *J* = 8.8 Hz, 2H, 2 x C^{12}**H**), 7.00 (d, *J* = 8.7 Hz, 2H, 2 x C^{11}**H**), 5.83 (s, 2H, 2 x C^{9}**H**), 3.75 (s, 3H, 3 x C^{14}**H**), 2.76 (s, 3H, 3 x C^{15}**H**); ^{13}C NMR ($(CD_3)_2SO$) δ 160.0 (C^Q), 148.0 (C^1), 136.9 ($C^{6/7}$), 136.6 (C^Q), 135.2 (C^Q), 133.1 (C^3), 131.1 ($C^{5/8}$ + $C^{6/7}$), 130.6 (2 x C^{12}), 126.9 (C^Q), 126.0 (C^Q), 124.4 ($C^{5/8}$), 114.5 (2 x C^{11}), 63.0 (C^9), 55.3 (C^{15}), 15.8 (C^{16}); HRMS (ESI): Exact mass calculated for $C_{18}H_{18}NO$ $[M]^+$: 264.1383, found: 264.1384; m.p. (acetone) 203 – 205 °C; IR (neat) (cm^{-1}) 3345, 2547, 2160, 2033, 1977, 1606, 1514, 1252, 781, 766.

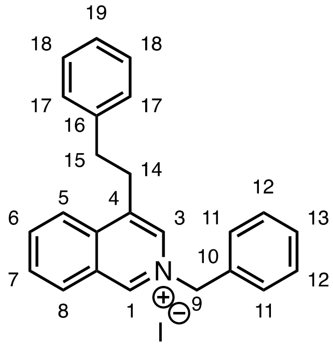

***N*-Benzyl-4-phenethylisoquinolinium iodide (1c)** The title compound was prepared according to General Procedure C using 4-phenethylisoquinoline (0.534 g, 2.29 mmol) and benzyl iodide (0.34 mL, 2.75 mmol) to give salt **1c** (0.683 g, 66 %) as a yellow solid. ^{1}H NMR ($(CD_3)_2SO$) δ 10.11 (s, 1H, C^1**H**), 8.71 (s, 1H, C^3**H**), 8.57 – 8.48 (m, 2H, C^{5+8}**H**), 8.29 (ddd, *J* = 8.5, 7.0, 1.3 Hz, 1H $C^{6/7}$**H**), 8.14 – 8.04 (m, 1H, $C^{6/7}$**H**), 7.51 – 7.41 (m, 5H, 5 x Ar**H**), 7.27 – 7.16 (m, 5H, 5 x Ar**H**), 5.88 (s, 2H, 2 x C^9**H**), 3.51 (t, 2H, *J* = 7.8 Hz, 2 x C^{14}**H**), 3.05 (t, *J* = 8.8 Hz, 2H, 2 x C^{15}**H**); ^{13}C NMR ($(CD_3)_2SO$) δ 148.5 (C^1H), 140.0 (C^Q), 138.1 (C^Q), 137.1 ($C^{6/7}$), 136.1 (C^Q), 134.3 (C^Q), 133.7 (C^3), 131.4 ($C^{5/8}$), 131.1 ($C^{6/7}$), 129.24 (C^{Ar}), 129.20 (2 x C^{Ar}), 128.6 (2 x C^{Ar}), 128.5 (C^{Ar}), 128.4 (2 x C^{Ar}), 127.3 (C^Q), 126.3 (2 x C^{Ar}), 124.2 ($C^{5/8}$), 63.3 (C^9), 35.1 (C^{15}), 30.8 (C^{14}); HRMS (ESI): Exact mass calculated for $C_{24}H_{22}N$ $[M]^+$: 324.17468, found: 324.17456; m.p. (acetone) 157 – 159 °C; IR (neat) (cm^{-1}): 3480, 3094, 2998, 2250, 2130, 1701, 1495, 1472, 728, 629.

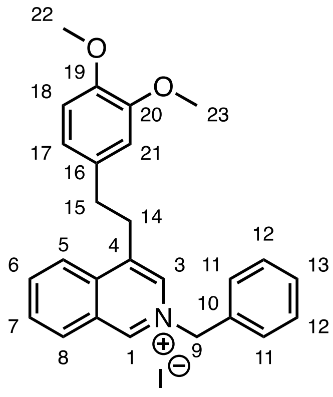

***N*-Benzyl-4-(3,4-dimethoxyphenethyl)isoquinolinium iodide (1d)** The title compound was prepared according to General Procedure C using 4-(3,4-dimethoxyphenethyl)isoquinoline (0.714 g, 2.43 mmol) and benzyl iodide (2.92 mmol) to give salt **1d** (0.64 g, 68 %) as a yellow solid. ^{1}H NMR ($(CD_3)_2SO$) δ 10.10 (s, 1H, C^1**H**), 8.71 (s, 1H, C^3**H**), 8.52 (d, *J* = 7.7 Hz, 2H, C^{5+8}**H**), 8.29 (ddd, *J* = 8.5, 7.0, 1.2, 1H, $C^{6/7}$**H**), 8.09 (ddd, *J* = 8.1, 7.0, 1.0, 1H, $C^{6/7}$**H**), 7.48 – 7.37 (m, 5H, 5 x C^{Ar}**H**), 6.83 (d, *J* = 2.0, 1H, C^{21}**H**), 6.75 (d, *J* = 8.2, 1H, C^{18}**H**), 6.65 (dd, *J* = 8.1, 2.0, 1H, C^{17}**H**), 5.89 (s, 2H, 2 x C^9**H**), 3.68 (d, *J* = 5.5, 6H, 3 x C^{22}**H** + 3 x C^{23}**H**), 3.50 (t, *J* = 7.7, 2H, 2 x C^{14}**H**), 2.98 (t, *J* = 7.7, 2H, 2 x C^{15}**H**); ^{13}C NMR ($(CD_3)_2SO$) δ 148.7 (C^Q), 148.3 (C^1), 147.3 (C^Q), 138.3 (C^Q), 137.0 ($C^{6/7}$), 136.1 (C^Q), 134.2 (C^Q), 133.7 (C^3), 132.3 (C^Q), 131.3 ($C^{5/8}$), 131.1 ($C^{6/7}$), 129.2 (C^{Ar}), 129.1 (2 x C^{Ar}), 128.4 (2 x C^{Ar}), 127.3 (C^Q), 124.2 ($C^{5/8}$), 120.3 (C^{17}), 112.3 (C^{21}), 111.7 (C^{18}), 63.3 (C^9), 55.49 ($C^{22/23}$), 55.46 ($C^{22/23}$), 34.8 (C^{15}), 30.9 (C^{14}); HRMS (ESI): Exact mass calculated for $C_{26}H_{26}NO_2$ $[M]^+$: 384.1964, found: 384.1956;

m.p. (acetone) 213 – 215 °C; IR (neat) (cm^{-1}): 3100, 3056, 1624, 1589, 1530, 1261, 1313, 1067, 702, 658.

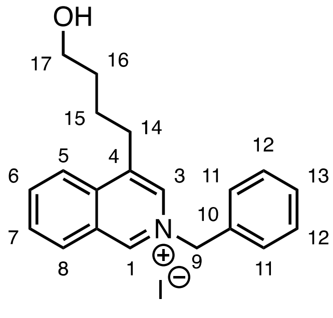

***N*-Benzyl-4-(butan-1-ol)isoquinolinium iodide (1e)** The title compound was prepared according to General Procedure C using 4-(butan-1-ol)isoquinoline (0.875 g, 4.35 mmol) and benzyl iodide (0.65 mL, 5.22 mmol) to give salt **1e** (1.221 g, 67 %) as a yellow solid. ^{1}H NMR ($(CD_3)_2SO$) δ 10.12 (s, 1H, C^{1}**H**), 8.76 (s, 1H, C^{3}**H**), 8.52 (dd, *J* = 8.3, 1.1 Hz, 1H, $C^{5/8}$**H**), 8.45 (d, *J* = 8.6 Hz, 1H, $C^{5/8}$**H**), (8.29 (ddd, *J* = 8.2, 7.0, 1.3, 1H, $C^{6/7}$**H**), 8.08 (ddd, *J* = 8.2, 7.0, 1.0, 1H, $C^{6/7}$**H**), 7.59 – 7.55 (m, 2H, 2 x C^{Ar}**H**), 7.48 – 7.41 (m, 3H, 3 x C^{Ar}**H**), 5.93 (s, 2H, 2 x C^{9}**H**), 4.45 (t, *J* = 5.0 Hz, 1H, O**H**), 3.44 (td, *J* = 6.3, 5.0, 2H, 2 x C^{17}**H**), 3.19 (t, *J* = 7.8, 2H, 2 x C^{14}**H**), 1.74 (dq, *J* = 12.3, 7.6, 2H, 2 x C^{15}**H**), 1.53 (p, *J* = 6.5, 2H, 2 x C^{16}**H**); ^{13}C NMR ($(CD_3)_2SO$) δ 148.3 (C^{1}), 139.2 (C^{Q}), 137.0 ($C^{6/7}$), 136.1 (C^{Q}), 134.3 (C^{Q}), 133.2 (C^{3}), 131.3 ($C^{5/8}$), 131.0 ($C^{6/7}$), 129.2 (C^{Ar}) , 129.1 (2 x C^{Ar}), 128.6 (2 x C^{Ar}), 127.3 (C^{Q}), 124.1 ($C^{5/8}$), 63.3 (C^{9}), 60.1 (C^{17}), 31.9 (C^{16}), 28.9 (C^{14}), 26.2 (C^{15}); HRMS (ESI): Exact mass calculated for $C_{20}H_{22}NO$ $[M]^+$: 292.1696, found: 292.1696; m.p. (acetone) 158 – 160 °C; IR (neat) (cm^{-1}) 3490, 3043, 2411, 1728, 1516, 1508, 1439, 1204, 971, 636.

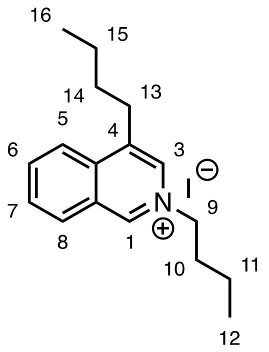

***N*-Butyl-4-butylisoquinolinium iodide (1f)** The title compound was prepared according to General Procedure E using 4-butylisoquinoline (0.556 g, 3.00 mmol) and butyl iodide (0.68 mL, 6.00 mmol) to give salt **1f** (1.02 g, 92 %) as a green solid. ^{1}H NMR ($(CD_3)_2SO$) δ 9.97 (s, 1H, C^{1}**H**), 8.73 (d, *J* = 1.4 Hz, 1H, C^{3}**H**), 8.51 – 8.41 (m, 2H, C^{5+8}**H**), 8.28 (ddd, *J* = 8.4, 7.0, 1.3 Hz, 1H, $C^{6/7}$**H**), 8.07 (ddd, *J* = 8.1, 7.0, 1.0 Hz, 1H, $C^{6/7}$**H**), 4.68 (t, *J* = 7.4 Hz, 2H, 2 x C^{9}**H**), 3.23 – 3.13 (m, 2H, 2 x C^{13}**H**), 2.01 (p, *J* = 7.6 Hz, 2H, 2 x C^{10}**H**), 1.71 (p, *J* = 7.5 Hz, 2H, 2 x C^{14}**H**), 1.44 (dt, *J* = 14.9, 7.4 Hz, 2H, 2 x C^{15}**H**), 1.33 (dt, *J* = 14.7, 7.4 Hz, 2H, 2 x C^{11}**H**), 0.97 – 0.91 (m, 6H, 3 x C^{12}**H** + 3 x C^{16}**H**); ^{13}C NMR ($(CD_3)_2SO$) δ 148.1 (C^{1}), 138.7 (C^{Q}), 136.7 ($C^{6/7}$), 135.9 (C^{Q}), 133.4 (C^{3}), 131.1 ($C^{5/8}$), 130.9 ($C^{6/7}$), 127.2 (C^{Q}), 124.0 ($C^{5/8}$), 60.4 (C^{9}), 32.4 (C^{10}), 31.7 (C^{14}), 28.8 (C^{13}), 21.9 (C^{15}), 18.9 (C^{11}), 13.7 ($C^{12/16}$), 13.4 ($C^{12/16}$); HRMS (ESI) Exact mass calculated for $C_{17}H_{24}N$ $[M]^+$ = 242.1903, found: 242. 1904; m.p. (1,4-dioxane) 134 – 136 °C; IR (neat) (cm^{-1}): 2958, 2926, 2857, 1639, 1458, 1437, 1343, 1178, 786, 762.

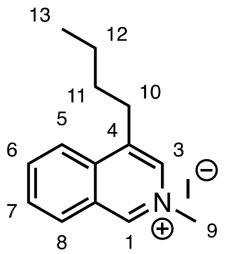

***N*-Methyl-4-butylisoquinolinium iodide (1g)** The title compound was prepared according to General Procedure D using 4-butylisoquinoline (0.556 g, 3.00 mmol) to give salt **1g** (1.09 g, 99 %) as a yellow solid. ^{1}H NMR ((CD_3)$_2$SO) δ 9.88 (s, 1H, C^{1}**H**), 8.62 (s, 1H, C^{3}**H**), 8.50 – 8.40 (m, 2H, C^{5+8}**H**), 8.27 (ddd, *J* = 8.5, 6.9, 1.3 Hz, 1H, C$^{6/7}$**H**), 8.06 (ddd, *J* = 8.1, 6.9, 1.0 Hz, 1H, C$^{6/7}$**H**), 4.44 (s, 3H, 3 x C^{9}**H**), 3.23 – 3.14 (m, 2H, 2 x C^{10}**H**), 1.71 (tt, *J* = 9.1, 7.5 Hz, 2H, 2 x C^{11}**H**), 1.44 (h, *J* = 7.4 Hz, 2H, 2 x C^{12}**H**), 0.95 (t, *J* = 7.4 Hz, 3H, 3 x C^{13}**H**); ^{13}C NMR ((CD_3)$_2$SO) δ 148.8 (C^{1}), 138.1 (C^{3}), 136.5 (C$^{6/7}$), 135.6 (C^{Q}), 134.4 (C^{3}), 130.9 (C$^{5/8}$), 130.8 (C$^{6/7}$), 127.1 (C^{Q}), 124.0 (C$^{5/8}$), 47.8 (C^{9}), 31.7 (C^{11}), 28.7 (C^{10}), 22.0 (C^{12}), 13.8 (C^{13}); HRMS (ESI) Exact mass calculated for $C_{14}H_{18}N$ [M]$^{+}$ = 200.143, found: 200.1436; m.p. (CH_2Cl_2) 144 – 146 °C; IR (neat) (cm^{-1}): 2957, 2294, 2836, 1642, 1606, 1404, 1189, 857, 789, 759.

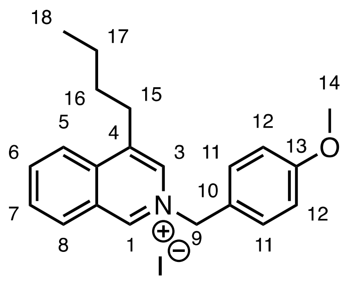

***N*-(*p*-Methoxy)benzyl-4-butylisoquinolinium iodide (1h)** The title compound was prepared according to General Procedure C using 4-butylisoquinoline (0.371 g, 2.00 mmol) and *p*-methoxybenzyl iodide (0.34 mL, 2.40 mmol) to give salt **1h** (0.986 g, 99 %) as a white solid. ^{1}H NMR ((CD_3)$_2$SO) δ 10.11 (s, 1H, C^{1}**H**), 8.76 (d, *J* = 1.4, 1H, C^{3}**H**), 8.53 (dd, *J* = 8.4, 1.3 Hz, 1H, C$^{5/8}$**H**), 8.44 (d, *J* = 8.6 Hz, 1H, C$^{5/8}$**H**), 8.28 (ddd, *J* = 8.5, 7.0, 1.3 Hz, 1H, C$^{6/7}$**H**), 8.08 (ddd, *J* = 8.1, 6.9, 1.0 Hz, 1H, C$^{6/7}$**H**), 7.62 – 7.54 (m, 2H, 2 x C^{12}**H**), 7.05 – 6.95 (m, 2H, 2 x C^{11}**H**), 5.86 (s, 2H, 2 x C^{9}**H**), 3.76 (s, 3H, 3 x C^{14}**H**), 3.21 – 3.15 (m, 2H, 2 x C^{15}**H**), 1.72 – 1.63 (m, 2H, 2 x C^{16}**H**), 1.40 (h, *J* = 7.3 Hz, 2H, 2 x C^{17}**H**), 0.93 (t, *J* = 7.3 Hz, 3H, 3 x C^{18}**H**); ^{13}C NMR ((CD_3)$_2$SO) δ 159.9 (C^{Q}), 147.9 (C^{1}), 139.1 (C^{Q}), 136.9 (C$^{6/7}$), 136.0 (C^{Q}), 133.1 (C^{3}), 131.3 (C$^{5/8}$), 131.0 (C$^{6/7}$), 130.6 (2 x C^{11}), 127.3 (C^{Q}), 126.2 (C^{Q}), 124.1 (C$^{5/8}$), 114.5 (2 x C^{12}), 63.0 (C^{9}), 55.3 (C^{14}), 31.7 (C^{16}), 28.8 (C^{15}), 21.9 (C^{17}), 13.7 (C^{18}); HRMS (ESI): Exact mass calculated for $C_{21}H_{24}NO$ [M]$^{+}$: 306.1852, found: 306.1852; m.p. (acetone) 164 – 166 °C; IR (neat) (cm^{-1}) 2981, 2361, 2160, 2029, 1976, 1642, 1607, 1512, 1251, 1024.

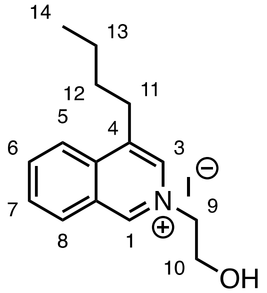

***N*-Hydroxyethyl-4-butylisoquinolinium iodide (1i)** The title compound was prepared according to General Procedure E using 4-butylisoquinoline (0.371 g, 2.00 mmol) and iodoethanol (0.38 mL, 4.00 mmol) to give salt **1i** (0.818 g, 92 %) as an orange solid. ^{1}H NMR ((CD_3)$_2$SO) δ 9.88 (s, 1H, C^{1}**H**), 8.68 (s, 1H, C^{3}**H**), 8.54 (dt, *J* = 8.2, 1.0 Hz, 1H, C$^{5/8}$**H**), 8.45 (dd, *J* = 8.5, 1.0 Hz, 1H, C$^{5/8}$**H**), 8.28 (ddd, *J* = 8.5, 7.0, 1.3 Hz, 1H, C$^{6/7}$**H**), 8.08 (ddd, *J* = 8.1, 7.0, 1.0 Hz, 1H, C$^{6/7}$**H**), 5.21 (t, *J* = 5.4 Hz, 1H, O**H**), 4.74 (t, *J* = 5.0 Hz, 2H, 2 x C^{9}**H**), 3.96 (q, *J* = 5.2 Hz, 2H, 2 x C^{10}**H**), 3.20 (t, *J* = 7.8 Hz, 2H, 2 x C^{11}**H**), 1.72 (tt, *J* = 9.1, 7.6 Hz, 2H, 2 x C^{12}**H**), 1.45 (h, *J* = 7.3 Hz, 2H, 2 x C^{13}**H**), 1.44 (dt, *J* = 7.3 Hz, 3H, 3 x C^{14}**H**); ^{13}C NMR ((CD_3)$_2$SO) 148.5 (C^{1}), 138.3 (C^{Q}), 136.6 (C$^{6/7}$), 136.0 (C^{Q}), 133.6

(C^3), 131.1 ($C^{5/8}$), 130.8 ($C^{6/7}$), 127.0 (C^Q), 124.0 ($C^{5/8}$), 63.1 (C^9), 59.9 (C^{10}), 31.6 (C^{12}), 28.8 (C^{11}), 21.9 (C^{13}), 13.7 (C^{14}); HRMS (ESI) Exact mass calculated for $C_{15}H_{20}NO$ $[M]^+$ = 230.1539, found: 230.1539; m.p. (1,4-dioxane) 131 – 133 °C; IR (neat) (cm^{-1}) 3346, 3052, 2999, 2957, 2161, 1641, 1375, 1074, 1059, 767.

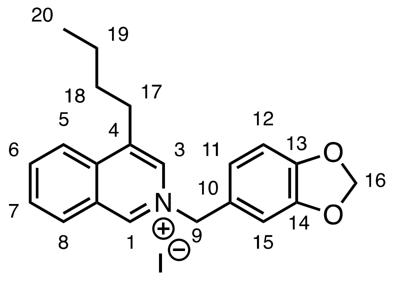

***N*-Piperonyl-4-butylisoquinolinium iodide (1j)** The title compound was prepared according to General Procedure C using 4-butylisoquinoline (0.371 g, 2.00 mmol) and piperonyl iodide (0.629 g, 2.40 mmol) to give salt **1j** (0.986 g, 99 %) as a yellow solid. ^1H NMR ($(CD_3)_2SO$) δ 10.08 (s, 1H, C^1**H**), 8.75 (s, 1H, C^3**H**), 8.52 (d, *J* = 7.9 Hz, 1H, $C^{5/8}$**H**), 8.43 (dd, *J* = 8.6, 1.0 Hz, 1H, $C^{5/8}$**H**), 8.28 (ddd, *J* = 8.4, 6.9, 1.3 Hz, 1H, $C^{6/7}$**H**), 8.08 (ddd, *J* = 8.1, 7.0, 1.0 Hz, 1H, $C^{6/7}$**H**), 7.27 (d, *J* = 1.7, 1H, C^{15}**H**), 7.18 (dd, *J* = 8.0, 1.8 Hz, 1H, C^{11}**H**), 6.99 (d, *J* = 8.0, 1H, C^{12}**H**), 6.04 (s, 2H, 2 x C^{16}**H**), 5.81 (s, 2H, 2 x C^9**H**), 3.22 – 3.12 (m, 2H, 2 x C^{17}**H**), 1.73 – 1.61 (m, 2H, 2 x C^{18}**H**), 1.40 (h, *J* = 7.3, 2H, 2 x C^{19}**H**), 0.93 (t, *J* = 7.4, 3H, 3 x C^{20}**H**); ^{13}C NMR ($(CD_3)_2SO$) δ 148.1 (C^Q), 148.0 (C^1), 147.8 (C^Q), 139.1 (C^Q), 136.9 ($C^{6/7}$), 136.0 (C^Q), 133.0 (C^3), 131.4 ($C^{5/8}$), 131.0 ($C^{6/7}$), 127.7 (C^Q), 127.3 (C^Q), 124.1 ($C^{5/8}$), 123.3 (C^{11}), 109.3 (C^{15}), 108.7 (C^{12}), 101.5 (C^{16}) 63.2 (C^9), 31.7 (C^{18}), 28.8 (C^{17}), 21.9 (C^{19}), 13.7 (C^{20}); HRMS (ESI): Exact mass calculated for $C_{21}H_{22}NO_2$ $[M]^+$: 320.1645, found: 320.1645; m.p. (acetone) 191 – 193 °C; IR (neat) (cm^{-1}) 3050, 1630, 1555, 1504, 1499, 1284, 1100, 952, 741, 728.

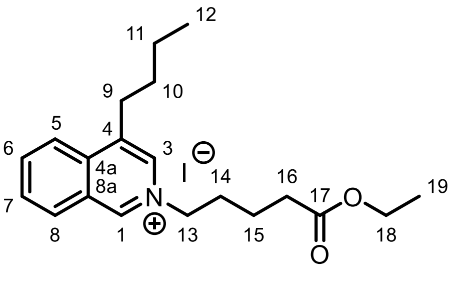

Ethyl *N*-(1-pentanoate)-4-butylisoquinolinium iodide (1k)
4-Butylisoquinoline (185 mg, 1 mmol) and ethyl 5-iodopentanoate (370 mg, 1.5 mmol) were heated in dioxane (5 mL) at 90 °C for 14 hours. The reaction was cooled and concentrated *in vacuo*, the crude material was purified by FCC (0-5% MeOH:CH_2Cl_2) to give *salt* **1k** (420 mg, 95%) as a yellow oil. ^{1} H NMR ($(CD_3)_2SO$) δ 9.97 (s, 1H, C^1**H**), 8.72 (d, *J* = 1.4 Hz, 1H, C^3**H**), 8.50-8.43 (m, 2H, C^{5+8}**H**), 8.28 (ddd, *J* = 8.4, 6.9, 1.3 Hz, 1H, $C^{6/7}$**H**), 8.02 (ddd, *J* = 8.1, 6.9, 1.0 Hz, 1H, $C^{6/7}$**H**), 4.70 (t, *J* = 7.2 Hz, 2H, C^{13}**H**$_2$), 4.03 (q, *J* = 7.1 Hz, 2H, C^{18}**H**$_2$), 3.19 (t, *J* = 7.8 Hz, 2H, C^9**H**$_2$), 2.38 (t, *J* = 7.4 Hz, 2H, C^{16}**H**$_2$), 2.05 (p, *J* = 7.4 Hz, 2H, C^{14}**H**$_2$), 1.76-1.68 (m, 2H, C^{10}**H**$_2$), 1.60-1.53 (m, 2H, C^{15}**H**$_2$), 1.48-1.39 (m, 2H, C^{11}**H**$_2$), 1.15 (t, *J* = 7.1 Hz, 3H, C^{19}**H**$_3$), 0.95 (t, *J* = 7.3 Hz, 3H, C^{12}**H**$_2$); ^{13}C NMR ($(CD_3)_2SO$) δ 172.9 (C^{17}), 148.6 (C^1), 139.2 (C), 137.2 ($C^{6/7}$), 136.4 (C), 133.9 (C^3), 131.6 ($C^{5/8}$), 131.3 ($C^{6/7}$), 127.7 (C^4), 124.5 ($C^{5/8}$), 60.7 (C^{13}), 60.3 (C^{18}), 33.2 (C^{16}), 32.1 (C^{10}), 30.2 (C^{14}), 29.3 (C^9), 22.4 (C^{11}), 21.4 (C^{15}), 14.6 (C^{19}), 14.2 (C^{12}); HRMS (ESI): Exact mass calculated for $C_{20}H_{28}NO_2$ $[M]^+$: 314.2115, found: 314.2114; IR (neat) (cm^{-1}): 2956, 1726 (C=O), 1640, 1444, 1373, 1177, 1028, 787, 759, 618.

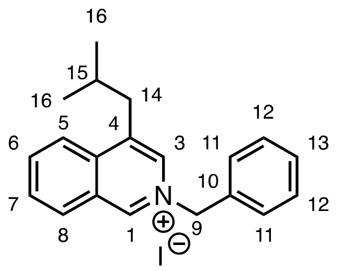

***N*-Benzyl-4-*iso*-butylisoquinolinium iodide (1l)** The title compound was prepared according to General Procedure C using 4-*iso*-butylisoquinoline (0.758 g, 4.09 mmol) and benzyl iodide (0.62 mL, 4.91 mmol) to give salt **1l** (1.210 g, 99 %) as a yellow solid. ^{1}H NMR ($(CD_3)_2SO$) δ 10.16 (s, 1H, C^1**H**), 8.75 (d, *J* = 1.4, 1H, C^3**H**), 8.53 (d, *J* = 7.9 Hz, 1H, C$^{5/8}$**H**), 8.45 (dd, *J* = 8.6, 1.0 Hz, 1H, C$^{5/8}$**H**), 8.28 (ddd, *J* = 8.5, 7.0, 1.3 Hz, 1H, C$^{6/7}$**H**), 8.08 (ddd, *J* = 8.1, 7.0, 1.0 Hz, 1H, C$^{6/7}$**H**), 7.64 – 7.55 (m, 2H, 2 x C^{Ar}**H**), 7.49 – 7.40 (m, 3H, 3 x C^{Ar}**H**), 5.95 (s, 2H, 2 x C^9**H**), 3.05 (d, *J* = 7.2 Hz, 2H, 2 x C^{14}**H**), 1.99 (h, *J* = 6.8 Hz, 1H, C^{15}**H**), 0.90 (d, *J* = 6.6 Hz, 6H, 6 x C^{16}**H**); ^{13}C NMR ($(CD_3)_2SO$) δ 148.3 (C^1), 138.0 (C^Q), 136.9 (C$^{6/7}$) 136.3 (C^Q), 134.4 (C^Q), 133.9 (C^3), 131.3 (C$^{5/8}$), 131.0 (C$^{6/7}$), 129.2 (C^{Ar}), 129.1 (2 x C^{Ar}), 128.6 (2 x C^{Ar}), 127.4 (C^Q), 124.3 (C$^{5/8}$), 63.3 (C^9), 37.7 (C^{14}), 29.0 (C^{15}), 22.1 (2 x C^{16}); HRMS (ESI): Exact mass calculated for $C_{20}H_{22}N$ [M]$^+$: 276.1747, found: 276.1747; m.p. (acetone) 157-159 °C; IR (neat) (cm^{-1}): 3130, 2979, 2160, 2020, 1621, 1476, 1253, 1156, 889, 699.

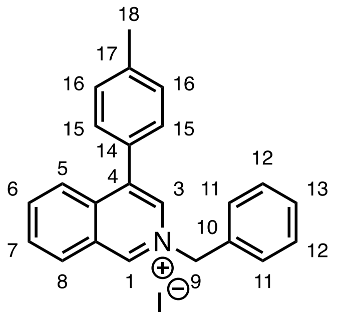

***N*-Benzyl-4-(*p*-tolyl)isoquinolinium iodide (1m)** The title compound was prepared according to General Procedure C using 4-(p-tolyl)isoquinoline (1.05 g, 4.79 mmol) and benzyl iodide (0.72 mL, 5.75 mmol) to give salt **1m** (1.603 g, 77 %) as a brown solid. ^{1}H NMR ($(CD_3)_2SO$) δ 10.27 (s, 1H, C^1**H**), 8.94 (s, 1H, C^3**H**), 8.65 – 8.58 (m, 1H, C$^{5/8}$**H**), 8.26 (ddd, *J* = 8.4, 6.9, 1.3 Hz, 1H, C$^{6/7}$**H**), 8.12 (dd, *J* = 8.1, 6.8 Hz, 2H, C$^{5/8}$**H** + C$^{6/7}$**H**), 7.69 – 7.62 (m, 2H, 2 x C^{Ar}**H**), 7.55 – 7. 52 (2H, m, 2 x C^{15}**H)**, 7.50 – 7.41 (m, 5H, 3 x C^{Ar}**H** + 2 x C^{16}**H**), 6.01 (s, 2H, 2 x C^9**H**), 2.44 (s, 3H, 3 x C^{18}**H**); ^{13}C NMR ($(CD_3)_2SO$) δ 148.8 (C^1), 139.4 (C^Q), 138.5 (C^Q), 137.6 (C$^{6/7}$), 135.5 (C^Q), 134.3 (C^Q), 133.8 (C^3), 131.3 (C$^{5/8}$ + C$^{5/8}$ or C$^{6/7}$), 131.3 (2 x C^{Ar}), 130.0 (2 x C^{Ar}), 130.0 (C^Q), 129.7 (C^{Ar}), 129.3 (C^{Ar}), 129.2 (C^{Ar}), 128.9 (2 x C^{Ar}), 127.8 (C^Q), 125.2 (C$^{6/7}$ or C$^{5/8}$), 63.4 (C^{16}), 20.9 (C^{18}); HRMS (ESI): Exact mass calculated for $C_{23}H_{20}N$ [M]$^+$: 310.1590, found: 310.1590; m.p. (acetone) 155 – 157 °C; IR (neat) (cm^{-1}): 3456, 2966, 2160, 2033, 1979, 1632, 1400, 1179, 740, 664.

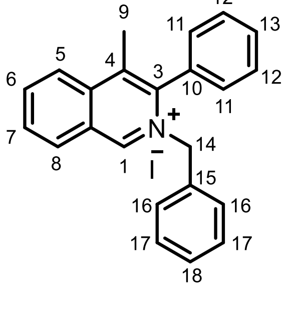

***N*-Butyl-4,5-dibutylisoquinolinium iodide (1n)** The title compound was prepared according to General Procedure C using 3-phenyl-4-butylisoquinoline (0.318 g, 1.45 mmol) and benzyl iodide (0.26 mL, 2.00 mmol). FCC (0-6% MeOH in CH_2Cl_2) gave salt **1n** (0.511 g, 81%) as a yellow solid. ^{1}H NMR ($(CD_3)_2SO$) δ 10.30 (1H, s, C^1**H**), 8.62 (1H, dt, *J* = 8.2, 1.1 Hz, C$^{5/8}$**H**), 8.48 (1H, dd, *J* = 8.6, 1.0 Hz, C$^{5/8}$**H**), 8.37 (1H, ddd, *J* = 8.5, 6.9, 1.3

Hz, $C^{6/7}$**H**), 8.16 (1H, ddd, *J* = 8.0, 6.9, 1.0 Hz, $C^{6/7}$**H**), 7.64-7.55 (1H, m, Ar**H**), 7.54-7.49 (2H, m, 2 x Ar**H**), 7.32-7.22 (5H, m, 5 x Ar**H**), 6.87-6.84 (2H, m, 2 x Ar**H**), 5.72 (2H, s, C^{14}**H**$_2$), 2.42 (3H, s, C^9**H**$_3$); ^{13}C NMR ($(CD_3)_2SO$) δ 149.9 (C^1), 143.5 (C^Q), 137.5 ($C^{6/7}$), 137.3 (C^Q), 134.4 (C^Q), 133.8 (C^Q), 131.3 ($C^{5/8}$), 131.1 ($C^{6/7}$ + C^Q), 130.3 (ArC), 129.9 (2 x ArC), 129.0 (2 x ArC), 128.6 (2 x ArC), 128.5 (ArC), 127.2 (2 x ArC), 126.3 (C^Q), 124.9 ($C^{5/8}$), 62.5 (C^{14}), 16.0 (C^9); HRMS (ESI): Exact mass calculated for $C_{23}H_{20}N$ $[M]^+$: 310.15903, found: 310.15891; m.p. (acetone) 192 – 194 °C; IR (neat) (cm^{-1}) 1628, 1594, 1572, 1478, 1452, 1271, 1110, 776, 704, 694.

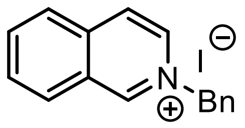

***N*-Benzyl-isoquinolinium iodide (1o)** The title compound was prepared according to literature precedence[6] using isoquinoline purchased from Alfa Aesar.

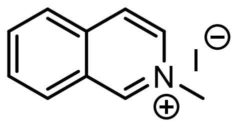

***N*-Methyl-isoquinolinium iodide (1p)** The title compound was prepared according to literature precedence[7] using isoquinoline purchased from Alfa Aesar

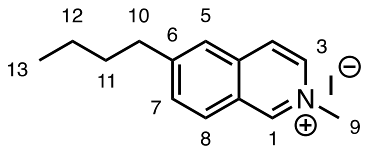

***N*-Methyl-6-butylisoquinolinium iodide (1q)** The title compound was prepared according to General Procedure D using 6-butylisoquinoline (0.432 g, 2.33 mmol) to give salt **1q** (0.563 g, 86 %) as a yellow solid. ^{1}H NMR ($(CD_3)_2SO$) δ 9.90 (s, 1H, C^1**H**), 8.64 (dd, *J* = 6.8, 1.4 Hz, 1H, C^3**H**), 8.44 (d, *J* = 6.8 Hz, 1H, C^4**H**), 8.39 (d, *J* = 8.5 Hz, 1H, C^8**H**), 8.14 (s, 1H, C^5**H**), 7.95 (dd, *J* = 8.5, 1.6 Hz, 1H, $C^{6/7}$**H**), 4.44 (s, 3H, 3 x C^9**H**), 2.93 (t, *J* = 7.7 Hz, 2H, 2 x C^{10}**H**), 1.78 – 1.60 (m, 2H, 2 x C^{11}**H**), 1.35 (h, *J* = 7.4 Hz, 2H, 2 x C^{12}**H**), 0.93 (t, *J* = 7.3 Hz, 3H, 3 x C^{13}**H**); ^{13}C NMR ($(CD_3)_2SO$) δ 152.6 (C^Q), 149.9 (C^1), 136.9 (C^4), 135.8 (C^3), 132.6 (C^7), 129.9 (C^8), 125.6 (C^Q), 125.4 (C^5), 124.6 (C^4), 47.6 (C^9), 35.4 (C^{10}), 32.2 (C^{11}), 21.7 (C^{12}), 13.7 (C^{13}); HRMS (ESI) Exact mass calculated for $C_{14}H_{18}N$ $[M]^+$ = 200.1434, found: 200.1435. m.p. (CH_2Cl_2) 174 – 176 °C; IR (neat) (cm^{-1}): 2988, 2161, 1646, 1370, 1185, 1162, 934, 901, 828, 656.

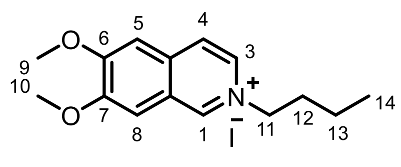

***N*-Butyl-6,7-dimethoxyisoquinolinium iodide (1r)** The title compound was prepared according to General Procedure E using 6,7-dimethoxyisoquinoline (0.378 g, 2.00 mmol) and 1-iodobutane (0.3 mL, 2.40 mmol) to give salt **1r** (0.594 g, 80%) as a peach solid. ^{1}H NMR ($(CD_3)_2SO$) δ) 9.64 (1H, s, C^1**H**), 8.59 (1H, dd, *J* = 6.7, 1.5 Hz, C^3**H**), 8.30 (1H, d, *J* = 6.7 Hz, C^4**H**), 7.77 (2H, app s, C^5**H** + C^8**H**), 4.63 (2H, t, *J* = 7.3 Hz, C^{11}**H**$_2$), 4.07 (3H, s, $C^{9/10}$**H**$_3$), 4.00 (3H, s, $C^{9/10}$**H**$_3$), 1.96 (2H, p, *J* = 7.4 Hz, C^{12}**H**$_2$), 1.31 (2H, dq, *J* = 14.5, 7.5 Hz, C^{13}**H**$_2$), 0.93 (3H, t, *J* = 7.4 Hz,

[6] Fang, Z; Wang, Y; Wang, Y *Org. Lett.* **2019**, *21*, 434-438.
[7] Xu, J.-H; Zheng, S.-C; Zhang, J.-W; Liu, X.-Y; Tan, B. *Angew. Chem. Int. Ed*. **2016**, *55*, 11834-11839.

C^{14}**H**$_3$); ^{13}C NMR ((CD$_3$)$_2$SO) δ 157.5 (C^{Q}), 152.5 (C^{Q})¸ 145.0 (C^{1}), 135.2 (C^{Q}), 133.6 (C^{3}), 123.8 (C^{Q}), 123.4 (C^{4}), 107.1 (C$^{5/8}$), 105.8 (C$^{5/8}$), 59.8 (C^{11}), 56.9 (C$^{9/10}$), 56.4 (C$^{9/10}$), 32.5 (C^{12}), 18.8 (C^{13}), 13.4 (C^{14}); HRMS (ESI): Exact mass calculated for $C_{15}H_{20}NO_2$ [M]$^+$: 246.14886, found: 246.14890; m.p. (acetone) 207 – 209 °C; IR (neat) (cm^{-1}) 1495, 1454, 1437, 1310, 1294, 1235, 1164, 991, 850, 753.

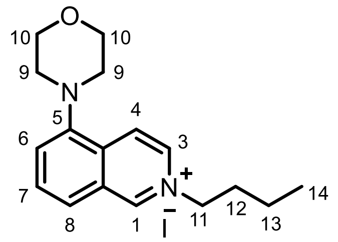

***N*-Butyl-5-morpholinoisoquinolinium iodide (1s)** The title compound was prepared according to General Procedure E using 5-morpholinoisoquinoline (0.428 g, 2.00 mmol) and 1-iodobutane (0.3 mL, 2.40 mmol) to give salt **1s** (0.612 g, 77%) as a yellow solid. ^{1}H NMR ((CD$_3$)$_2$SO) δ) 10.07 (1H, s, C^{1}**H**), 8.73 (1H, dd, *J* = 7.0, 1.5 Hz, C^{3}**H**), 8.57 (1H, d, *J* = 6.9 Hz, C^{4}**H**), 8.13 (1H, d, *J* = 8.2 Hz, C^{8}**H**), 7.99 (1H, t, *J* = 7.9 Hz, C^{7}**H**), 7.81 (1H, dd, *J* = 7.7, 1.1 Hz, C^{5}**H**), 4.74 (2H, t, *J* = 7.4 Hz, C^{11}**H**$_2$), 3.93-3.86 (4H, m, 2 x C^{10}**H**$_2$), 3.13-305 (4H, m, 2 x C^{9}**H**$_2$), 1,99 (2H, p, *J* = 7.6 Hz, C^{12}**H**$_2$), 1.35 (2H, h, *J* = 7.3 Hz, C^{13}**H**$_2$), 0.93 (3H, t, *J* = 7.3 Hz, C^{14}**H**$_3$); ^{13}C NMR ((CD$_3$)$_2$SO) δ 149.9 (C^{Q}), 148.8 (C^{1}), 134.4 (C^{3}), 132.4 (C^{Q})¸131.8 (C^{7}), 128.7 (C^{Q}), 124.9 (C^{6}), 124.5 (C^{8}), 122.2 (C^{4}), 66.3 (2 x C^{10}), 60.3 (C^{11}), 52.8 (2 x C^{9}), 32.5 (C^{12}), 18.8 (C^{13}), 13.4 (C^{14}); HRMS (ESI): Exact mass calculated for $C_{17}H_{23}N_2O$ [M]$^+$: 271.18049, found: 271.18043; m.p. (acetone) 179 – 181 °C; IR (neat) (cm^{-1}) 1638, 1592, 1573, 1451, 1403, 1231, 1114, 1030, 837, 759.

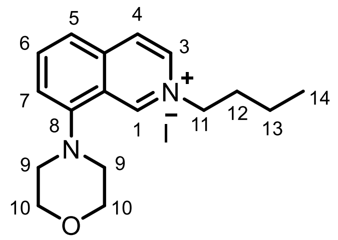

***N*-Butyl-8-morpholinoisoquinolinium iodide (1t)** The title compound was prepared according to General Procedure E using 8-morpholinoisoquinoline (0.428 g, 2.00 mmol) and 1-iodobutane (0.3 mL, 2.40 mmol) to give salt **1t** (0.610 g, 77%) as a yellow solid. ^{1}H NMR ((CD$_3$)$_2$SO) δ) 9.70 (1H, s, C^{1}**H**), 8.77 (1H, dd, *J* = 6.8, 1.4 Hz, C^{3}**H**), 8.54 (1H, d, *J* = 6.8 Hz, C^{4}**H**), 8.14 (1H, t, *J* = 8.0 Hz, C^{6}**H**), 7.95 (1H, d, *J* = 8.0 Hz, C^{5}**H**), 7.60 (1H, dd, *J* = 7.8, 0.9 Hz, C^{7}**H**), 4.80 (2H, t, *J* = 7.5 Hz, C^{11}**H**$_2$), 3.97-3.92 (4H, m, 2 x C^{10}**H**$_2$), 3.20-3.15 (4H, m, 2 x C^{9}**H**$_2$), 1.97 (2H, p, *J* = 7.5 Hz, C^{12}**H**$_2$), 1.36 (2H, h, *J* = 7.4 Hz, C^{13}**H**$_2$), 0.94 (3H, t, *J* = 7.4 Hz, C^{14}**H**$_2$); ^{13}C NMR ((CD$_3$)$_2$SO) δ 152.5 (C^{Q}), 145.8 (C^{1}), 138.6 (C^{Q})¸137.6 (C^{6}), 134.8 (C^{3}), 126.1 (C^{4}), 122.5 (C^{Q})¸121.4 (C^{5}), 119.7 (C^{7}), 66.0 (2 x C^{10}), 60.5 (C^{11}), 53.6 (2 x C^{9}), 32.7 (C^{12}), 18.9 (C^{13}), 13.4 (C^{14}); HRMS (ESI): Exact mass calculated for $C_{17}H_{23}N_2O$ [M]$^+$: 271.18049, found: 271.18063; m.p. (acetone) 203 – 205 °C; IR (neat) (cm^{-1}) 1637, 1593, 1570, 1367, 1303, 1190, 1111, 1033, 804, 759.

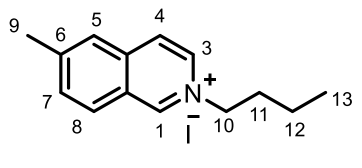

***N*-Butyl-6-methylisoquinolinium iodide (1u)** The title compound was prepared according to General Procedure E using 6-methylisoquinoline (0.286 g, 2.00 mmol) and 1-iodobutane (0.3 mL, 2.40 mmol). FCC (0-4% MeOH in CH_2Cl_2) gave salt **1u** (0.447 g, 68%) as a yellow solid. ^{1}H NMR ((CD$_3$)$_2$SO) δ) 10.00 (1H, s, C^{1}**H**), 8.75 (1H, dd, *J* = 6.8, 1.5 HZ, C^{3}**H**), 8.46 (1H,

d, J = 6.8 Hz, C^4**H**), 8.38 (1H, d, J = 8.5 Hz, C^8**H**), 8.13 (1H, s, C^5**H**), 7.92 (1H, dd, J = 8.5, 1.7 Hz, C^7**H**), 4.68 (2H, t, J = 7.4 Hz, C^{10}**H**$_2$), 2.66 (3H, s, C^9**H**$_3$), 1.99 (2H, p, J = 7.5 Hz, C^{11}**H**$_2$), 1.34 (2H, h, J = 7.4 Hz, C^{12}**H**$_2$), 0.93 (3H, t, J = 7.4 Hz, C^{13}**H**$_3$); ^{13}C NMR ($(CD_3)_2SO$) δ 149.1 (C^Q), 148.5 (C^1), 137.1 (C^Q), 135.0 (C^3), 133.3 (C^7), 130.0 (C^8), 126.0 (C^5), 125.6 (C^Q), 125.0 (C^4), 60.3 (C^{10}), 32.4 (C^{11}), 22.2 (C^9), 18.9 (C^{12}), 13.4 (C^{13}); HRMS (ESI): Exact mass calculated for $C_{14}H_{18}N$ $[M]^+$: 200.14338, found: 200.14352; m.p. (acetone) 95 – 97 °C; IR (neat) (cm^{-1}) 1644, 1606, 1571, 1539, 1462, 1366, 1186, 1112, 815, 717.

Transition metal free reductive hydroxymethylation

General Procedure F: A mixture of the appropriate isoquinolinium iodide (0.25 mmol, 1 equiv.), potassium methoxide (10 equiv.), and paraformaldehyde (120 equiv.) in methanol (0.25 mL) was heated at 65 °C for 24 hours in a sealed microwave vial. The reaction mixture was evaporated to dryness under reduced pressure, quenched with distilled water (10 mL) and extracted in EtOAc (20 mL x 3). The organic layer was dried over anhydrous sodium sulphate, filtered and evaporated under reduced pressure. The crude compound was purified using chromatography to obtain the pure product.

***rac-N*-Benzyl-4-butyl-4-hydroxymethyltetrahydroisoquinoline (2a)** The title compound was prepared according to General Procedure F using *N*-benzyl-4-butylisoquinolinium iodide (100.8 mg) and purified by silica gel chromatography (10-20% EtOAc in pentane) to give THIQ **2a** as a yellow oil (47 mg, 64%). ^{1}H NMR ($CDCl_3$) δ 7.36 (d, *J* = 4.0 Hz, 4H, 4 x C^{Ar}**H**), 7.34 – 7.19 (m, 3H, 3 x C^{Ar}**H**), 7.13 (td, *J* = 7.4, 1.6 Hz, 1H, C$^{6/7}$**H**), 6.98 (dd, *J* = 7.6, 1.3 Hz, 1H, C$^{5/8}$**H**), 5.65 (s, 1H, O**H**), 3.85 (dd, *J* = 14.8, 1.9 Hz, 1H, C^1**H**), 3.78 (d, *J* = 9.9 Hz, 1H, C^{14}**H**), 3.70 (d, *J* = 12.8 Hz, 1H, C^9**H**), 3.63 (d, *J* = 7.3 Hz, 1H, C^9**H**), 3.60 (dd, *J* = 7.1, 2.7 Hz, 1H, C^{14}**H**), 3.32 (d, *J* = 14.6 Hz, 1H, C^1**H**), 2.98 (dd, *J* = 11.4, 1.9 Hz, 1H, C^3**H**), 2.64 (dd, *J* = 11.4, 2.7 Hz, 1H, C^3**H**), 1.67 (ddd, *J* = 14.0, 12.2, 4.6 Hz, 1H, C^{15}**H**), 1.39 (ddd, *J* = 14.0, 12.1, 4.7 Hz, 1H, C^{15}**H**), 1.30 – 1.17 (m, 2H, 2 x C^{17}**H**), 1.16 – 0.93 (m, 2H, 2 x C^{16}**H**), 0.84 (t, *J* = 7.3 Hz, 3H, 3 x C^{18}**H**); ^{13}C NMR ($CDCl_3$) δ 138.0 (C^Q), 136.9 (C^Q), 135.6 (C^Q), 129.1 (2 x C^{Ar}), 128.6 (2 x C^{Ar}), 127.6 (C^{Ar}), 127.0 (C^{Ar}), 126.2 (C^{Ar}), 126.0 (C^{Ar}), 125.7 (C^{Ar}), 76.3 (C^{14}), 63.1 (C^9), 60.9 (C^3), 56.8 (C^1), 42.1 (C^Q), 35.0 (C^{15}), 26.0 (C^{16}), 23.5 (C^{17}), 13.9 (C^{18}); HRMS (ESI) Exact mass calculated for $C_{21}H_{28}NO$ [M + H]$^+$ = 310.2171, found 310.2163; IR (neat) (cm^{-1}): 3402, 2980, 2861, 2835, 1522, 1498, 1248, 1207, 729, 653.

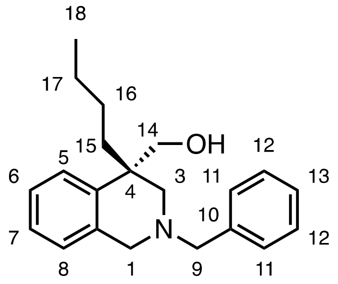

***rac-N*-(*p*-Methoxy)benzyl-4-methyl-4-hydroxymethyltetrahydroisoquinoline (2b)** The title compound was prepared according to General Procedure F using *N*-(*p*-methoxy)benzyl-4-methylisoquinolinium iodide (97.8 mg) and purified by silica gel chromatography (10-30% EtOAc in pentane) to give THIQ **2b** as a yellow oil (48 mg, 61%). ^{1}H NMR ($CDCl_3$) δ 7.34 (dd, *J* = 7.8, 1.3 Hz, 1H, C$^{5/8}$**H**), 7.28 (d, *J* = 2.1 Hz, 2H, 2 x C^{12}**H**), 7.25 – 7.21 (m, 1H, C$^{6/7}$**H**), 7.15 (td, *J* = 7.4, 1.4 Hz, 1H, C$^{6/7}$**H**), 7.00 (dd, *J* = 7.5, 1.4 Hz, 1H, C$^{5/8}$**H**), 6.90 (d, *J* = 8.6 Hz, 2H, 2 x C^{11}**H**), 5.75 – 5.57 (m, 1H, O**H**), 3.89 (dd, *J* = 14.9, 1.8 Hz, 1H, C^1**H**), 3.82 (s, 3H, 3 x C^{14}**H**), 3.75 (d, *J* = 9.9 Hz, 1H, C^9**H**), 3.64 (dd, *J* = 9.9, 2.7 Hz, 1H, C^9**H**), 3.60 (d, *J* = 1.1 Hz, 2H, 2 x C^{15}**H**), 3.35 (d, *J* = 14.8 Hz, 1H,

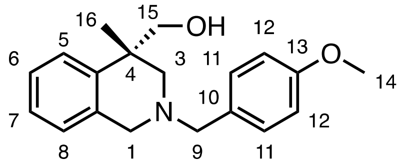

C^1**H**), 3.04 (dd, *J* = 11.4, 1.9 Hz, 1H, C^3**H**), 2.43 (dd, *J* = 11.4, 2.7 Hz, 1H, C^3**H**), 1.15 (s, 3H, 3 x C^{16}**H**); ^{13}C NMR ($CDCl_3$) δ 159.0 (C^Q), 139.6 (C^Q), 134.9 (C^Q), 130.3 (2 x C^{12}), 128.9 (C^Q), 127.0 ($C^{6/7}$), 126.2 ($C^{6/7}$), 126.1 ($C^{5/8}$), 125.5 ($C^{5/8}$), 113.9 (2 x C^{11}), 76.2 (C^9), 63.5 (C^3), 62.2 (C^{15}), 56.7 (C^1), 55.3 (C^{14}), 38.9 (C^Q), 22.4 (C^{16}); HRMS (ESI) Exact mass calculated for $C_{19}H_{24}NO_2$ $[M + H]^+$ = 298.1807, found: 298.104; IR (neat) (cm^{-1}): 3312, 3120, 2891, 1657, 1538, 1488, 1281, 1045, 742, 711.

***rac-N*-Benzyl-4-phenethyl-4-hydroxymethyltetrahydroisoquinoline (2c)** The title compound was prepared according to General Procedure F using *N*-benzyl-4-(3,4-phenethyl)isoquinolinium iodide (112.8 mg) and purified by silica gel chromatography (10-30% EtOAc in pentane) to give THIQ **2c** as a yellow oil (50 mg, 56%). ^1H NMR ($CDCl_3$) δ 7.39 – 7.36 (m, 4H, 4 x C^{Ar}**H**), 7.36 – 7.27 (m, 4H, 4 x C^{Ar}**H**), 7.25 (d, *J* = 7.6 Hz, 1H, C^{Ar}**H**), 7.20 – 7.14 (m, 2H, 2 x C^{Ar}**H**), 7.10 (dd, *J* = 8.1, 1.4 Hz, 2H, 2 x C^{Ar}**H**), 7.03 (dd, *J* = 7.7, 1.3 Hz, 1H, C^{Ar}**H**), 5.55 (s, 1H, O**H**), 3.88 (dd, *J* = 14.8, 1.8 Hz, 1H, C^1**H**), 3.81 (d, *J* = 9.9 Hz, 1H, C^{14}**H**), 3.73 (d, *J* = 12.8 Hz, 1H, C^9**H**), 3.68 (d, *J* = 7.1 Hz, C^9**H**), 3.65 (dd, 1H, C^{14}**H**), 3.39 (d, *J* = 14.7 Hz, 1H, C^1**H**), 3.06 (dd, *J* = 11.3, 1.9 Hz, 1H, C^3**H**), 2.75 (dd, *J* = 11.3, 2.7 Hz, 1H, C^3**H**), 2.45 (td, *J* = 13.0, 5.4 Hz, 1H, C^{16}**H**), 2.35 (ddd, *J* = 13.3, 12.5, 4.5 Hz, 1H, C^{16}**H**), 1.99 (ddd, *J* = 14.1, 12.5, 4.5 Hz, 1H, C^{15}**H**), 1.72 (ddd, *J* = 14.1, 12.2, 5.4 Hz, 1H, C^{15}**H**); ^{13}C NMR ($CDCl_3$) δ 142.2 (C^Q), 137.4 (C^Q), 136.8 (C^Q), 135.7 (C^Q), 129.1 (2 x C^{Ar}), 128.6 (2 x C^{Ar}), 128.33 (2 x C^{Ar}), 128.30 (2 x C^{Ar}), 127.6 (C^{Ar}), 127.1 (C^{Ar}), 126.3 (C^{Ar}), 126.1 (C^{Ar}), 125.8 (C^{Ar}), 125.6 (C^{Ar}), 75.9 (C^{14}), 63.0 (C^9), 60.5 (C^3), 56.7 (C^1), 42.3 (C^Q), 37.3 (C^{15}), 30.3 (C^{16}); HRMS (ESI) Exact mass calculated for $C_{25}H_{28}NO$ $[M + H]^+$ = 358.2171, found: 358.2163; IR (neat) (cm^{-1}): 3328, 3005, 2934, 2910, 2887, 1553, 1503, 1109, 1084, 758.

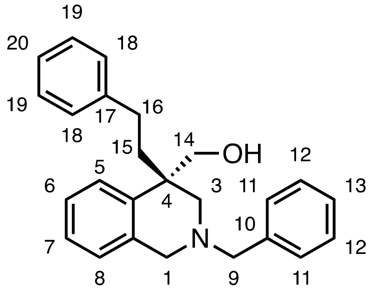

***rac-N*-Benzyl-4-(3,4-dimethoxyphenethyl)-4-hydroxymethyltetrahydroisoquinoline (2d)** The title compound was prepared according to General Procedure F using *N*-benzyl-4-(3,4-dimethoxyphenethyl)isoquinolinium iodide (175.3 mg) and purified by silica gel chromatography (10-40% EtOAc in pentane) to give THIQ **2d** as a yellow oil (65 mg, 62%). ^1H NMR ($CDCl_3$) δ 7.37 (d, *J* = 4.5 Hz, 4H, 4 x C^{Ar}**H**), 7.36 – 7.28 (m, 3H, 3 x C^{Ar}**H**), 7.18 (td, *J* = 7.4, 1.4 Hz, 1H, $C^{6/7}$**H**), 7.03 (dd, *J* = 7.6, 1.3 Hz, 1H, $C^{5/8}$**H**), 6.77 (d, *J* = 8.1 Hz, 1H, C^{19}**H**), 6.63 (dd, *J* = 8.1, 2.0 Hz, 1H, C^{18}**H**), 6.59 (d, *J* = 2.0 Hz, 1H, C^{22}**H**), 5.55 (s, 1H, O**H**), 3.91 – 3.86 (m, 1H, C^1**H**), 3.85 (d, *J* = 2.0 Hz, 6H, 3 x C^{23}**H** + 3 x C^{24}**H**), 3.82 (d, *J* = 9.9 Hz, 1H, C^{14}**H**), 3.72 (d, *J* = 12.8 Hz, 1H, C^9**H**), 3.69 – 3.64 (m, 2H, C^{9+14}**H**), 3.38 (d, *J* = 14.7 Hz, 1H, C^1**H**), 3.06 (dd, *J* = 11.3, 1.8 Hz, 1H, C^3**H**), 2.73 (dd, *J* = 11.4, 2.6 Hz, 1H, C^3**H**), 2.41 (td, *J* = 13.1, 5.4 Hz, 1H, C^{16}**H**), 2.30 (ddd, *J* = 13.7, 12.1, 4.5 Hz, 1H, C^{16}**H**), 1.98 (ddd, *J* = 14.1, 12.4,

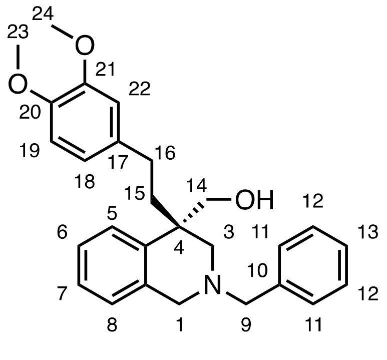

4.6 Hz, 1H, C^{15}**H**), 1.72 (ddd, *J* = 14.1, 12.1, 5.4 Hz, 1H, C^{16}**H**); ^{13}C NMR ($CDCl_3$) δ 148.8 (C^{Q}), 147.1 (C^{Q}), 137.5 (C^{Q}), 136.8 (C^{Q}), 135.7 (C^{Q}), 134.9 (C^{Q}), 129.1 (2 x C^{Ar}), 128.6 (2 x C^{Ar}), 127.6 (C^{Ar}), 127.2 (C^{Ar}), 126.4 (C$^{5/8}$), 126.2 (C$^{6/7}$), 125.6 (C^{Ar}), 119.8 (C^{18}), 111.4 (C^{22}), 111.2 (C^{19}), 76.1 (C^{14}), 63.1 (C^{9}), 60.6 (C^{3}), 56.8 (C^{1}), 55.9 (C$^{23/24}$), 55.8 (C$^{23/24}$), 42.4 (C^{Q}), 37.3 (C^{15}), 30.0 (C^{16}); HRMS (ESI) Exact mass calculated for $C_{27}H_{32}NO_3$ $[M + H]^+$ = 418.2382, found: 418.2366. IR (neat) (cm^{-1}): 3351, 2910, 2887, 1652, 1528, 1469, 1283, 1275, 1100, 732.

***rac-N*-Benzyl-4-(butan-1-ol)-4-hydroxymethyltetrahydroisoquinoline (2e)** The title compound was prepared according to General Procedure F using *N*-benzyl-4-(butan-1-ol)isoquinolinium iodide (101.5 mg) and purified by column chromatography (10-40% EtOAc in pentane) to give THIQ **2e** as a yellow oil (56 mg, 69%). ^{1}H NMR ($CDCl_3$) δ 7.38 – 7.33 (m, 4H, 4 x C^{Ar}**H**), 7.34 – 7.19 (m, 3H, 3 x C^{Ar}**H**), 7.13 (ddd, *J* = 8.5, 6.8, 1.8 Hz, 1H, C^{Ar}**H**), 6.98 (d, *J* = 7.6, 1H, C^{Ar}**H**), 3.84 (dd, *J* = 14.8, 1.8 Hz, 1H, C^{1}**H**), 3.77 (d, *J* = 9.8 Hz, 1H, C^{14}**H**), 3.69 (d, *J* = 12.8 Hz, 1H, C^{9}**H**), 3.63 (d, *J* = 9.0 Hz, 1H, C^{9}**H**), 3.60 (dd, *J* = 8.0, 1.9 Hz, 1H, C^{14}**H**), 3.50 (dd, *J* = 6.7, 3.3 Hz, 2H, 2 x C^{18}**H**), 3.33 (d, *J* = 14.6 Hz, 1H, C^{1}**H**), 2.97 (dd, *J* = 11.4, 1.8 Hz, 1H, C^{3}**H**), 2.65 (dd, *J* = 11.4, 2.6 Hz, 1H, C^{3}**H**), 1.69 (ddd, *J* = 13.9, 12.5, 4.4 Hz, 1H, C^{16}**H**), 1.50 – 1.43 (m, 2H, 2 x C^{17}**H**), 1.42 – 1.36 (m, 1H, C^{16}**H**), 1.28 – 1.15 (m, 1H, C^{15}**H**), 1.15 – 0.98 (m, 1H, C^{15}**H**); ^{13}C NMR ($CDCl_3$) δ 137.7 (C^{Q}), 136.7 (C^{Q}), 135.5 (C^{Q}), 129.0 (2 x C^{Ar}), 128.5 (2 x C^{Ar}), 127.5 (C^{Ar}), 127.0 (C^{Ar}), 126.2 (C^{Ar}), 126.0 (C^{Ar}), 125.5 (C^{Ar}), 76.0 (C^{14}), 63.0 (C^{9}), 62.3 (C^{18}), 60.6 (C^{3}), 56.6 (C^{1}), 42.0 (C^{Q}), 34.9 (C^{16}), 33.2 (C^{17}), 20.2 (C^{15}); HRMS (ESI) Exact mass calculated for $C_{21}H_{28}NO_2$ $[M + H]^+$ = 326.2120, found: 326.2114; IR (neat) (cm^{-1}): 3394, 2900, 2876, 1561, 1489, 1172, 1125, 900, 741, 720.

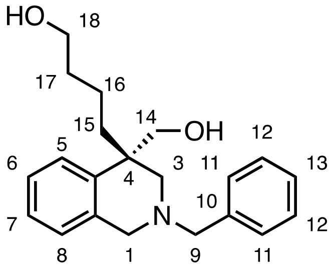

***rac-N*-Butyl-4-butyl-4-hydroxymethyltetrahydroisoquinoline (2f)** The title compound was prepared according to General Procedure F using *N*-butyl-4-butylisoquinolinium iodide (92.3 mg) and purified by silica gel chromatography (5–10% EtOAc in pentane) to give THIQ **2f** as a yellow oil (40 mg, 58%). ^{1}H NMR ($CDCl_3$) δ 7.28 (dd, *J* = 7.8, 1.6 Hz, 1H, C$^{5/8}$**H**), 7.23 (m, 1H, C$^{6/7}$**H**), 7.15 (td, *J* = 7.3, 1.6 Hz, 1H, C$^{6/7}$**H**), 7.04 (dd, *J* = 7.7, 1.3 Hz, 1H, C$^{5/8}$**H**), 6.02 (s, 1H, O**H**), 3.93 (dd, *J* = 14.6, 1.9 Hz, 1H, C^{1}**H**), 3.86 (d, *J* = 9.7 Hz, 1H, C^{13}**H**), 3.62 (dd, *J* = 9.7, 2.9 Hz, 1H, C^{13}**H**), 3.27 (dd, *J* = 14.6, 1.1 Hz, 1H, C^{1}**H**), 2.97 (dd, *J* = 11.4, 1.9 Hz, 1H, C^{3}**H**), 2.57 (dd, *J* = 11.4, 2.9 Hz, 1H, C^{3}**H**), 2.52 – 2.43 (m, 2H, 2 x C^{9}**H**), 1.72 – 1.52 (m, 3H, C^{14}**H** + 2 x C^{10}**H**), 1.47 – 1.32 (m, 3H, C^{14}**H + ** 2 x C^{11}**H**), 1.32 – 1.21 (m, 2H, 2 x C^{16}**H**), 1.19 – 0.99 (m, 2H, 2 x C^{15}**H**), 0.95 (t, *J* = 7.3 Hz, 3H, 3 x C^{12}**H**), 0.85 (t, *J* = 7.3 Hz, 3H, 3 x C^{17}**H**); ^{13}C NMR ($CDCl_3$) δ 138.3 (C^{Q}), 135.8 (C^{Q}), 126.9 (C$^{6/7}$), 126.1 (C$^{5/8}$), 125.87 (C$^{6/7}$), 125.85 (C$^{5/8}$), 76.6 (C^{13}), 61.5 (C^{3}), 58.0 (C^{9}), 56.9 (C^{1}), 41.8

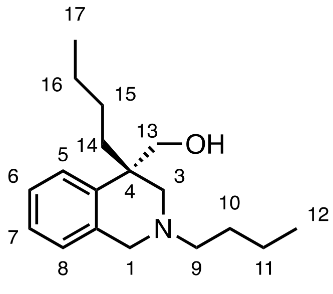

(C^Q), 35.0 (C^{14}), 28.9 (C^{10}), 26.0 (C^{15}), 23.5 (C^{16}), 20.5 (C^{11}), 13.94 (C^{12}), 13.88 (C^{17}); HRMS (ESI) Exact mass calculated for $C_{18}H_{30}NO$ [M + H]$^+$ = 276.2249, found: 276.2318; IR (neat) (cm^{-1}): 3381, 3347, 3004, 2991, 2874, 1438, 1426, 1161, 1123, 728.

***rac-N*-Methyl-4-butyl-4-hydroxymethyltetrahydroisoquinoline (2g)** The title compound was prepared according to General Procedure F using *N*-methyl-4-butylisoquinolinium iodide (81.8 mg) and purified by silica gel chromatography (10-30% EtOAc in pentane with 1% Et_3N) to give THIQ **2g** as a white solid (47 mg, 68%). ^{1}H NMR ($CDCl_3$) δ 7.28 (dd, *J* = 7.9, 1.6 Hz, 1H, $C^{5/8}$**H**), 7.23 (dt, *J* = 7.8, 1.0 Hz, 1H, $C^{6/7}$**H**), 7.15 (td, *J* = 7.3, 1.6 Hz, 1H, $C^{6/7}$**H**), 7.06 – 7.00 (m, 1H, $C^{5/8}$**H**), 5.92 (m, 1H, O**H**), 3.86 (s, 1H, C^1**H**), 3.85 – 3.80 (m, 1H, C^{10}**H**), 3.61 (dd, *J* = 9.7, 2.8 Hz, 1H, C^{10}**H**), 3.28 (dd, *J* = 14.5, 1.1 Hz, 1H, C^1**H**), 2.88 (dd, *J* = 11.4, 1.9 Hz, 1H, C^3**H**), 2.57 (dd, *J* = 11.4, 2.9 Hz, 1H, C^3**H**), 2.42 (s, 3H, 3 x C^9**H**), 1.66 (ddd, *J* = 13.9, 12.1, 4.7 Hz, 1H, C^{11}**H**), 1.41 (ddd, *J* = 14.0, 12.0, 4.7 Hz, 1H, C^{11}**H**), 1.32 – 1.20 (m, 2H, 2 x C^{13}**H**), 1.18 – 0.97 (m, 2H, 2 x C^{12}**H**), 0.84 (t, *J* = 7.3 Hz, 3H, 3 x C^{14}**H**); ^{13}C NMR ($CDCl_3$) δ 137.9 (C^Q), 135.7 (C^Q), 127.0 ($C^{6/7}$), 126.0 ($C^{5/8}$), 126.9 ($C^{6/7}$), 125.7 ($C^{5/8}$), 76.7 (C^{10}), 63.1 (C^3), 58.7 (C^1), 45.7 (C^9), 42.0 (C^Q), 35.0 (C^{11}), 26.1 (C^{12}), 23.5 (C^{13}), 13.9 (C^{14}); HRMS (ESI) Exact mass calculated for $C_{15}H_{24}NO$ [M + H]$^+$ = 234.1858, found: 234.1853; m.p. (chloroform) 83 – 85 °C; IR (neat) (cm^{-1}): 3230, 3010, 2931, 2918, 1476, 1465, 1223, 1204, 705, 692.

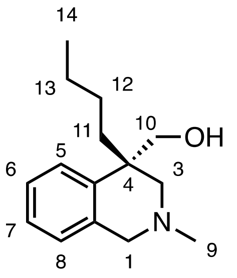

***rac-N*-(*p*-Methoxybenzyl)-4-butyl-4-hydroxymethyltetrahydroisoquinoline (2h)** The title compound was prepared according to General Procedure F using *N-(p*-methoxybenzyl)-4-butylisoquinolinium iodide (108.3 mg) and purified by silica gel chromatography (10–30% EtOAc in pentane) to give THIQ **2h** as a yellow oil (63 mg, 74%). ^{1}H NMR ($CDCl_3$) δ 7.29 – 7.24 (m, 3H, $C^{5/8}$**H** + 2 x C^{12}**H**), 7.23 (m, 1H, $C^{6/7}$**H**), 7.13 (td, *J* = 7.3, 1.6 Hz, 1H, $C^{6/7}$**H**), 7.00 – 6.96 (d, *J* = 8.4 Hz, 1H, $C^{5/8}$**H**), 6.92 – 6.86 (m, 2H, 2 x C^{11}**H**), 5.66 (s, 1H, O**H**), 3.86 – 3.80 (m, 4H, C^1**H + **3 x C^{14}**H**), 3.76 (d, *J* = 9.8 Hz, 1H, C^{15}**H**), 3.66 – 3.52 (m, 3H, C^{15}**H** + 2 x C^9**H**), 3.30 (d, *J* = 14.6 Hz, 1H, C^1**H**), 2.97 (dd, *J* = 11.4, 1.9 Hz, 1H, C^3**H**), 2.60 (dd, *J* = 11.4, 2.7 Hz, 1H, C^3**H**), 1.66 (ddd, *J* = 14.0, 12.2, 4.6 Hz, 1H, C^{16}**H**), 1.38 (ddd, *J* = 13.9, 12.0, 4.7 Hz, 1H, C^{16}**H**), 1.30 – 1.18 (m, 2H, 2 x C^{18}**H**), 1.17 – 0.91 (m, 2H, 2 x C^{17}**H**), 0.84 (t, *J* = 7.3 Hz, 3H, 3 x C^{19}**H**); ^{13}C NMR ($CDCl_3$) δ 159.0 (C^Q), 138.2 (C^Q), 135.7 (C^Q), 130.3 (2 x C^{12}), 128.9 (C^Q), 127.0 ($C^{6/7}$), 126.2 ($C^{5/8}$), 125.9 ($C^{6/7}$), 125.7 ($C^{5/8}$), 113.9 (2 x C^{11}), 76.3 (C^{15}), 62.4 (C^9), 60.7 (C^3), 56.7 (C^1), 55.3 (C^{14}), 42.1 (C^Q), 35.0 (C^{16}), 26.1 (C^{17}), 23.5 (C^{18}), 13.9 (C^{19}); HRMS (ESI) Exact mass calculated for $C_{22}H_{30}NO_2$ [M + H]$^+$ = 340.2277, found: 340.2266. IR (neat) (cm^{-1}): 3402, 3058, 2997, 1634, 1524, 1486, 1272, 1089, 781, 766.

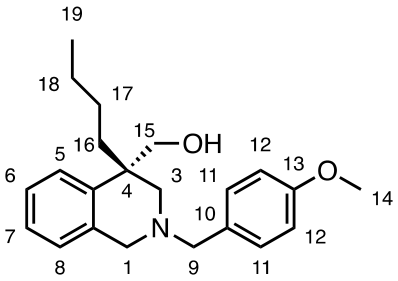

***rac-N*-Hydroxyethyl-4-butyl-4-hydroxymethyltetrahydroisoquinoline (2i)** The title compound was prepared according to General Procedure F using *N*-Hydroxyethyl-4-butylisoquinolinium iodide (89.3 mg) and purified by silica gel chromatography (2% methanol in CH_2Cl_2) to give THIQ **2i** as a yellow oil (34 mg, 54%). ^{1}H NMR ($CDCl_3$) δ 7.29 – 7.25 (m, 1H, $C^{5/8}$**H**), 7.23 (dd, *J* = 7.9, 1.4 Hz, 1H, $C^{6/7}$**H**), 7.15 (td, *J* = 7.3, 1.7 Hz, 1H, $C^{6/7}$**H**), 7.06 – 7.00 (m, 1H, $C^{5/8}$**H**), 3.89 (dd, *J* = 14.6, 1.8 Hz, 1H, C^1**H**), 3.84 – 3.70 (m, 3H, 2 x C^9**H** + C^{11}**H**), 3.67 (dd, *J* = 10.0, 2.1 Hz, 1H, C^{11}**H**), 3.44 (d, *J* = 14.5 Hz, 1H, C^1**H**), 3.02 (dd, *J* = 11.5, 1.9 Hz, 1H, C^3**H**), 2.77 – 2.51 (m, 3H, C^3**H** + 2 x C^{10}**H**), 1.68 (ddd, *J* = 14.0, 12.2, 4.6 Hz, 1H, C^{12}**H**), 1.46 (ddd, *J* = 14.0, 12.1, 4.6 Hz, 1H, C^{12}**H**), 1.32 – 1.19 (m, 2H, 2 x C^{14}**H**), 1.18 – 0.94 (m, 2H, 2 x C^{13}**H**), 0.84 (t, *J* = 7.3 Hz, 3H, 3 x C^{15}**H**); ^{13}C NMR ($CDCl_3$) δ 138.1 (C^Q), 135.6 (C^Q), 127.0 ($C^{6/7}$), 126.2 ($C^{5/8}$), 126.0 ($C^{5/8}$), 125.9 ($C^{6/7}$), 75.1 (C^{11}), 59.9 (C^3), 59.6 (C^{10}), 59.0 (C^9), 57.3 (C^1), 42.2 (C^Q), 35.2 (C^{12}), 26.1 (C^{13}), 23.4 (C^{14}), 13.9 (C^{15}); HRMS (ESI) Exact mass calculated for $C_{16}H_{26}NO_2$ [MH^+] = 264.1965, found: 264.1957; IR (neat) (cm^{-1}): 3410, 2951, 2908, 1459, 1441, 1168, 824, 755, 743, 690.

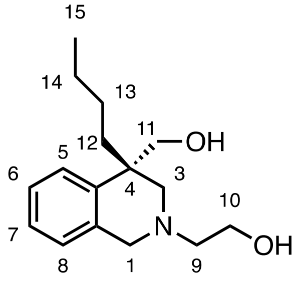

***rac-N*-Piperonyl-4-butyl-4-hydroxymethyltetrahydroisoquinoline (2j)** The title compound was prepared according to General Procedure F using *N*-piperonyl-4-butylisoquinolinium iodide (111.8 mg) and purified by silica gel chromatography (10-20% EtOAc in pentane) to give THIQ **2j** as a yellow oil (56 mg, 69%). ^{1}H NMR ($CDCl_3$) δ 7.27 (d, *J* = 6.5 Hz, 1H, $C^{5/8}$**H**), 7.23 (ddd, *J* = 8.4, 7.2, 1.4 Hz, 1H, $C^{6/7}$**H**), 7.13 (td, *J* = 7.3, 1.6 Hz, 1H, $C^{6/7}$**H**), 6.99 (dd, *J* = 7.6, 1.3 Hz, 1H, $C^{5/8}$**H**), 6.84 (d, *J* = 0.8 Hz, 1H, C^{15}**H**), 6.82 – 6.76 (m, 2H, C^{11+12}**H**), 5.96 (q, *J* = 1.4 Hz, 2H, 2 x C^{16}**H**), 5.45 (s, 1H, O**H**), 3.83 (dd, *J* = 14.6, 1.8 Hz, 1H, C^1**H**), 3.77 (d, *J* = 9.8 Hz, 1H, C^{17}**H**), 3.60 (d, *J* = 12.3 Hz, 2H, C^{9+14}**H**), 3.52 (d, *J* = 12.7 Hz, 1H, C^9**H**), 3.29 (d, *J* = 14.6 Hz, 1H, C^1**H**)), 2.97 (dd, *J* = 11.4, 1.9 Hz, 1H, C^3**H**), 2.60 (dd, *J* = 11.4, 2.7 Hz, 1H, C^3**H**), 1.67 (ddd, *J* = 14.0, 12.2, 4.6 Hz, 1H, C^{18}**H**), 1.40 (ddd, *J* = 14.0, 12.1, 4.7 Hz, 1H, C^{18}**H**), 1.30 – 1.19 (m, 2H, 2 x C^{20}**H**), 1.17 – 0.91 (m, 2H, 2 x C^{19}**H**), 0.84 (t, *J* = 7.3 Hz, 3H, 3 x C^{21}**H**); ^{13}C NMR ($CDCl_3$) δ 147.9 (C^Q), 147.0 (C^Q), 138.1 (C^Q), 135.6 (C^Q), 130.7 (C^Q), 127.0 ($C^{6/7}$), 126.2 ($C^{5/8}$), 126.0 ($C^{6/7}$), 125.7 ($C^{5/8}$), 122.4 ($C^{11/12}$), 109.5 (C^{15}), 108.2 ($C^{11/12}$), 101.1 (C^{16}), 76.2 (C^{17}), 62.9 (C^9), 60.7 (C^3), 56.6 (C^1), 42.1 (C^Q), 35.1 (C^{18}), 26.1 (C^{19}), 23.5 (C^{20}), 13.9 (C^{21}); HRMS (ESI) Exact mass calculated for $C_{22}H_{28}NO_3$ $[M + H]^+$ = 354.2069, found: 354.2059; IR (neat) (cm^{-1}): 3415, 2902, 2881, 1524, 1500, 1427, 1218, 1041, 905, 767.

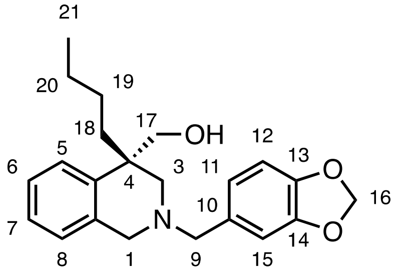

***rac-N*-Methyl 5-pentanoate-4-butyl-4-hydroxymethyltetrahydroisoquinoline (2k)**

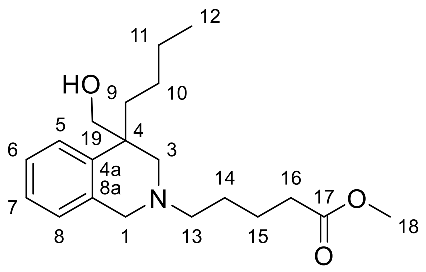

The title compound is prepared according to General Procedure F using salt **1k** (110 mg, 0.25 mmol). The crude material was purified by FCC (0-30% EtOAc:pentane) to give THIQ **2k** (51 mg, 64%) as a colourless oil.

^{1} H NMR ($CDCl_3$) δ 7.30-7.23 (m, 2H, $C^{5/8}$**H** + $C^{6/7}$**H**), 7.16 (td, *J* = 7.3, 1.7 Hz, 1H, $C^{6/7}$**H**), 7.04 (dd, *J* = 7.6, 1.3 Hz, $C^{5/8}$**H**), 3.92 (dd, *J* = 14.6, 1.8 Hz, 1H, C^{1}**H**$_2$), 3.84 (d, *J* = 9.8 Hz, 1H, C^{19}**H**), 3.68 (s, 3H, C^{18}**H**$_3$), 3.63 (dd, *J* = 9.8, 2.7 Hz, 1H, C^{19}**H**$_2$), 3.29 (d, *J* = 14.5 Hz, 1H, C^{1}**H**$_2$), 2.96 (dd, *J* = 11.4, 1.9 Hz, 1H, C^{3}**H**$_2$), 2.57 (dd, *J* = 11.4, 2.8 Hz, 1H, C^{3}**H**$_2$), 2.51 (t, *J* = 6.9 Hz, 2H, C^{13}**H**$_2$), 2.37 (t, *J* = 6.8 Hz, 2H, C^{16}**H**$_2$), 1.73-1.62 (m, 5H, 2 x C^{14}**H**$_2$ + 2 x C^{15}**H**$_2$ + C^{9}**H**$_2$), 1.42 (ddd, *J* = 14.0, 12.0, 4.7 Hz, 1H, C^{9}**H**$_2$), 1.31-1.20 (m, 2H, C^{11}**H**$_2$), 1.14-0.99 (m, 2H, C^{10}**H**$_2$), 0.85 (t, *J* = 7.3 Hz, 3H, C^{12}**H**$_3$);

^{13}C NMR ($CDCl_3$) δ 173.8 (C^{17}), 138.2 (C), 135.6 (C), 127.0 ($C^{5/6/7/8}$), 126.1 ($C^{5/8}$), 125.9 ($C^{6/7}$), 125.7 ($C^{5/6/7/8}$), 76.4 (C^{19}), 61.3 (C^{3}), 57.9 (C^{13}), 56.9 (C^{1}), 51.5 (C^{18}), 41.9 (C^{4}), 35.0 (C^{9}), 33.7 (C^{16}), 26.3 ($C^{14/15}$), 26.1 (C^{10}), 23.5 (C^{11}), 22.7 ($C^{14/15}$), 13.9 (C^{12}); HRMS (ESI): Exact mass calculated for $C_{20}H_{32}NO_3$ $[M+H]^+$: 334.2377, found: 334.2377; IR (neat) (cm^{-1}): 2931, 1734 (C=O), 1491, 1452, 1171, 1039, 907, 760, 730, 619.

Tandem methylation-hydroxymethylation

General Procedure G: A mixture of the appropriate isoquinolinium iodide (0.25 mmol, 1 equiv.), potassium methoxide (12 equiv.), and paraformaldehyde (120 equiv.) in methanol (0.25 mL) was heated at 65 °C for 24 hours in a sealed microwave vial. The reaction mixture was evaporated to dryness under reduced pressure, quenched with distilled water (10 mL) and extracted in EtOAc (20 mL x 3). The organic layers were combined and dried over anhydrous sodium sulphate, filtered and evaporated under reduced pressure. The crude compound was purified by chromatography.

***rac-N*-Benzyl-4-methyl-4-hydroxymethyltetrahydroisoquinoline (2o)** The title compound was prepared according to General Procedure G using *N*-benzyl-isoquinolinium iodide[8] (86.8 mg) and purified by silica gel chromatography (10-30% EtOAc in pentane) to give THIQ **2o** as a yellow oil (40 mg, 54%). ^{1}H NMR ($CDCl_3$) δ 7.38 – 7.35 (m, 4H, 4 x C^{Ar}**H**), 7.35 – 7.29 (m, 2H, 2 x C^{Ar}**H**), 7.27 – 7.22 (m, 1H, C^{Ar}**H**), 7.16 (td, *J* = 7.4, 1.4 Hz, 1H, C^{Ar}**H**), 7.00 (dq, *J* = 7.6, 0.7 Hz, C^{Ar}**H**), 5.54 (s, 1H, O**H**), 3.90 (dd, *J* = 14.9, 1.8 Hz, 1H, C^1**H**), 3.77 (d, *J* = 9.9 Hz, 1H, C^{14}**H**), 3.72 – 3.66 (m, 2H, 2 x C^9**H**), 3.66 – 3.63 (m, 1H, C^{14}**H**), 3.38 (dd, *J* = 14.8, 1.0 Hz, 1H, C^1**H**), 3.06 (dd, *J* = 11.4, 1.9 Hz, 1H, C^3**H**), 2.48 (dd, *J* = 11.4, 2.6 Hz, 1H, C^3**H**), 1.16 (s, 3H, 3 x C^{15}**H**); ^{13}C NMR ($CDCl_3$) δ 139.5 (C^Q), 136.9 (C^Q), 134.8 (C^Q), 129.1 (2 x C^{Ar}), 128.6 (2 x C^{Ar}), 127.6 (C^{Ar}), 127.1 (C^{Ar}), 126.2 (C^{Ar}), 126.1 (C^{Ar}), 125.5 (C^{Ar}), 76.2 (C^{14}), 63.7 (C^3), 62.9 (C^9), 56.7 (C^1), 38.9 (C^Q), 22.3 (C^{15}); HRMS (ESI) Exact mass calculated for $C_{18}H_{22}NO$ $[M + H]^+$ = 268.1696, found: 268.1696; IR (neat) (cm^{-1}): 3027, 2930, 2807, 1493, 1452, 1140, 1092, 1030, 757, 690.

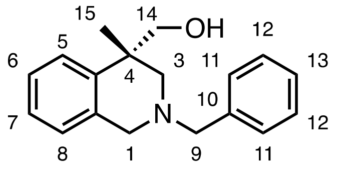

***rac-N*-Methyl-4-methyl-4-hydroxymethyltetrahydroisoquinoline (2p)** The title compound was prepared according to General Procedure G using *N*-methyl-isoquinolinium iodide[9] (67.8 mg) and purified by silica gel chromatography (50-70% EtOAc in pentane with 1% Et_3N) to give THIQ **2p** as a yellow oil (28 mg, 58%). ^{1}H NMR ($CDCl_3$) δ 7.36 (dd, *J* = 7.8, 1.3 Hz, 1H, C$^{5/8}$**H**), 7.25 (tt, *J* = 7.8, 1.1 Hz, 1H, C$^{6/7}$**H**), 7.17 (td, *J* = 7.4, 1.4 Hz, 1H, C$^{6/7}$**H**), 7.04 (dd, *J* = 7.6, 1.1 Hz, 1H, C$^{5/8}$**H**), 3.89 (dd, *J* = 14.7, 1.8 Hz, 1H, C^1**H**), 3.84 (d, *J* = 9.9 Hz, 1H, C^{10}**H**), 3.66 (dd, *J* = 9.9, 2.6 Hz, 1H, C^{10}**H**), 3.32 (d, *J* = 14.7 Hz, 1H, C^1**H**), 2.95 (dd, *J* = 11.4, 1.8 Hz, 1H, C^3**H**), 2.45 – 2.40 (m, 4H, 3 x C^9**H** + C^3**H**), 1.16 (s, 3H, 3 x C^{11}**H**); ^{13}C NMR ($CDCl_3$) δ 139.2 (C^Q), 134.9 (C^Q), 127.0 (C$^{6/7}$), 126.2 (C$^{6/7}$), 125.9 (C$^{5/8}$), 125.5 (C$^{5/8}$), 76.6 (C^{10}), 65.9 (C^3),

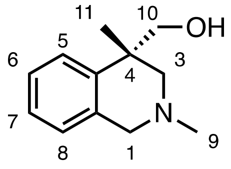

[8] Z. Fang, Y. Wang, Y. Wang, *Org. Lett.* **2019**, *21*, 434-438.
[9] F. Zeng, C.-F. Chen, *Org. Chem Biomol.* **2015**, *13*, 1988-1991.

58.6 (C^1), 45.5 (C^9), 38.7 (C^Q), 22.4 (C^{11}); HRMS (ESI) Exact mass calculated for $C_{12}H_{18}NO$ $[M + H]^+$ = 192.1388, found: 192.1385. IR (neat) (cm^{-1}): 3280, 3041, 2916, 1421, 1402, 1058, 1049, 767, 743.

***rac-N*-Benzyl-6-butyl-4-methyl-4-hydroxymethyltetrahydroisoquinoline (2q)** The title compound was prepared according to General Procedure G using *N*-methyl-6-butylisoquinolinium iodide (81.8 mg) and purified by silica gel chromatography (10-30% EtOAc in pentane with 1% Et_3N) to give THIQ **2q** as a yellow oil (23 mg, 34%). ^1H NMR ($CDCl_3$) δ 7.14 (d, *J* = 1.7 Hz, 1H, C^5**H**), 6.99 (dd, *J* = 7.8, 1.7 Hz, 1H, C^7**H**), 6.95 (d, *J* = 7.8 Hz, 1H, C^8**H**), 3.94 – 3.76 (m, 2H, C^{1+14}**H**), 3.66 (dd, *J* = 9.8, 2.8 Hz, 1H, C^{14}**H**), 3.28 (d, *J* = 14.5 Hz, 1H, C^1**H**), 2.93 (dd, *J* = 11.4, 1.8 Hz, 1H, C^3**H**), 2.62 – 2.51 (m, 2H, C^{10}**H**), 2.43 – 2.39 (m, 4H, 3 x C^9**H** + C^3**H**), 1.65 – 1.52 (m, 2H, C^{11}**H**), 1.42 – 1.25 (m, 2H, 2 x C^{12}**H**), 1.15 (s, 3H, 3 x C^{15}**H**), 0.93 (t, *J* = 7.3 Hz, 3H, 3 x C^{13}**H**); ^{13}C NMR ($CDCl_3$) δ 141.6 (C^Q), 138.9 (C^Q), 132.2 (C^Q), 126.4 (C^7), 125.7 (C^8), 125.4 (C^5), 76.7 (C^{14}), 66.1 (C^3), 58.5 (C^1), 45.6 (C^9), 38.7 (C^Q), 35.6 (C^{10}), 33.7 (C^{11}), 22.5 (C^{12}), 22.4 (C^{15}), 14.0 (C^{13}); HRMS (ESI) Exact mass calculated for $C_{16}H_{26}NO$ $[M + H]^+$ = 248.2014, found: 248.2011; IR (neat) (cm^{-1}): 3005, 2809, 1500, 1418, 1209, 1184, 1141, 1050, 749, 721.

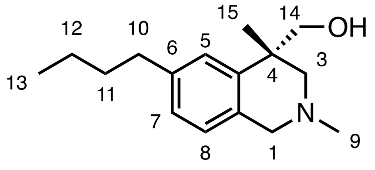

***rac-N*-Butyl-6,7-dimethoxy-4-methyl-4-hydroxymethyltetrahydroisoquinoline (2r)** The title compound was prepared according to General Procedure G using *N*-butyl-6,7-dimethoxyisoquinolinium iodide (93 mg) and purified by silica gel chromatography (10-40% EtOAc in pentane) to give THIQ **2r** as a colourless oil (37 mg, 51%). ^1H NMR ($CDCl_3$) δ 6.80 (1H, s, $C^{5/8}$**H**), 6.54 (1H, s, $C^{5/8}$**H**), 3.91 (1H, dd, *J* = 14.4, 1.7 Hz, C^1**H_2**), 3.87 (3H, s, $C^{9/10}$**H_3**), 3.84 (3H, s, $C^{9/10}$**H_3**), 3.82 (1H, d, *J* = 9.9 Hz, C^{12}**H_2**), 3.65 (1H, dd, *J* = 9.9, 2.8 Hz, C^{12}**H_2**), 3.26 (1H, d, *J* = 14.3 Hz, C^1**H_2**), 3.03 (1H, dd, *J* = 11.3, 1.7 Hz, C^3**H_2**), 2.55-2.43 (2H, m, C^{13}**H_2**), 2.40 (1H, dd, *J* = 11.4, 2.9 Hz, C^3**H_2**), 1.64-1.55 (2H, m, C^{14}**H_2**), 1.43-1.33 (2H, m, C^{15}**H_2**), 1.14 (3H, s, C^{11}**H_3**), 0.95 (3H, t, *J* = 7.3 Hz, C^{16}**H_3**); ^{13}C NMR ($CDCl_3$) δ 148.2 (C^Q), 147.7 (C^Q), 131.4 (C^Q), 127.2 (C^Q), 108.8 ($C^{5/8}$), 108.2 ($C^{5/8}$), 76.1 (C^{12}), 64.4 (C^3), 57.8 (C^{13}), 56.4 (C^1), 56.0 ($C^{9/10}$), 55.8 ($C^{9/10}$), 38.4 (C^4), 28.9 (C^{14}), 22.5 (C^{11}), 20.5 (C^{15}), 13.9 (C^{16}); HRMS (ESI) Exact mass calculated for $C_{17}H_{28}NO_3$ $[M + H]^+$ = 294.20637, found: 294.20621; IR (neat) (cm^{-1}): 2958, 1512, 1465, 1364, 1253, 1155, 1065, 907, 857, 726.

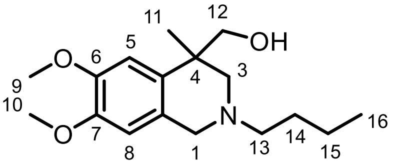

***rac-N*-Butyl-5-morpholino-4-methyl-4-hydroxymethyltetrahydroisoquinoline** **(2s)** The title compound was prepared according to General Procedure G using *N*-butyl-5-morpholinoisoquinolinium iodide (100 mg) and purified by silica gel chromatography (10-40% EtOAc in pentane) to give THIQ **2s** as a white solid (50 mg, 63%). ^{1}H NMR ($CDCl_3$) δ 7.26 (1H, d, *J* = 7.2 Hz, C^8**H**), 7.21 (1H, t, *J* = 7.6 Hz, C^7**H**), 6.94 (1H, d, *J* = 7.3 Hz, C^6**H**), 4.15 (1H, dd, *J* = 9.9, 2.8 Hz, C^{12}**H**$_2$), 3.95 (1H, dd, *J* = 14.7, 1.9 Hz, C^1**H**$_2$), 3.90 (2H, tt, *J* = 11.3, 2.9 Hz, 2 x C^{10}**H**$_2$), 3.80 (1H, d, *J* = 9.9 Hz, C^{12}**H**$_2$), 3.74 (2H, tt, *J* = 11.3, 2.5 Hz, 2 x C^{10}**H**$_2$), 3.33 (1H, d, *J* = 14.6 Hz, C^1**H**$_2$), 3.10 (1H, td, *J* = 11.6, 3.2 Hz, C^9**H**$_2$), 2.96 (2H, app td, *J* = 11.8, 3.2 Hz, C^3**H**$_2$ + C^9**H**$_2$), 2.73 (2H, ddd, *J* = 11.7, 4.1, 2.2 Hz, 2 x C^9**H**$_2$), 2.48 (2H, dq, *J* = 19.4, 6.8, 5.2 Hz, C^{13}**H**$_2$), 2.38 (1H, dd, *J* = 11.6, 2.8 Hz, C^3**H**$_2$), 1.60 (2H, dq, *J* = 7.9, 5.6 Hz, C^{14}**H**$_2$), 1.44-1.34 (5H, m, C^{11}**H**$_3$ + C^{15}**H**$_2$), 0.95 (3H, t, *J* = 7.3 Hz, C^{16}**H**$_2$); ^{13}C NMR ($CDCl_3$) δ 153.3 (C^Q), 136.9 (C^Q), 127.0 (C^7), 124.8 (C^6), 123.8 (C^8), 114.1 (C^Q), 74.3 (C^{12}), 67.6 (C^3), 67.2 (2 x C^{10}), 57.8 (C^1 + C^{13}), 55.1 (C^9), 53.9 (C^9), 40.0 (C^Q), 28.8 (C^{14}), 22.2 (C^{11}), 20.5 (C^{15}), 13.9 (C^{16}); HRMS (ESI) Exact mass calculated for $C_{19}H_{31}N_2O_2$ [M + H]$^+$ = 319.23800, found: 319.23795; m.p. (CH_2Cl_2) 143 – 145 °C; IR (neat) (cm^{-1}): 2956, 1605, 1570, 1539, 1462, 1365, 1338, 1259, 1111, 752.

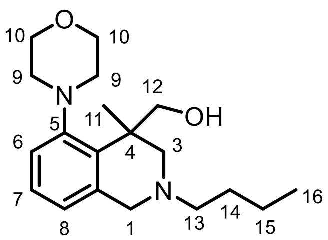

***rac-N*-Butyl-8-morpholino-4-methyl-4-hydroxymethyltetrahydroisoquinoline** **(2t)** The title compound was prepared according to General Procedure G using *N*-butyl-8-morpholinoisoquinolinium iodide (100 mg) and purified by silica gel chromatography (20-30% EtOAc in pentane) to give THIQ **2t** as a yellow oil (42 mg, 53%). ^{1}H NMR ($CDCl_3$) δ 7.27 (1H, t, *J* = 7.8 Hz, C^6**H**), 7.16 (1H, dd, *J* = 7.8, 1.1 Hz, C^5**H**), 6.99 (1H, dd, *J* = 7.8, 1.2 Hz, C^7**H**), 4.34 (1H, dd, *J* = 15.3, 2.0 Hz, C^1**H**$_2$), 3.90-3.78 (5H, m, 4 x C^{10}**H**$_2$ + C^{12}**H**$_2$), 3.70 (1H, dd, *J* = 9.9, 2.8 Hz, C^{12}**H**$_2$), 3.10-2.96 (4H, m, C^1**H**$_2$ + C^3**H**$_2$ + 2 x C^9**H**$_2$), 2.82-2.76 (2H, m, 2 x C^9**H**$_2$), 2.60-2.48 (2H, m, C^{13}**H**$_2$), 2.40 (1H, dd, *J* = 11.4, 2.8 Hz, C^3**H**$_2$), 1.64 (2H, app dddd, *J* = 14.5, 8.2, 6.9, 2.1 Hz, C^{14}**H**$_2$), 1.41 (2H, h, *J* = 7.4 Hz, C^{15}**H**$_2$), 1.17 (3H, s, C^{11}**H**$_2$), 0.97 (3H, t, *J* = 7.4 Hz, C^{16}**H**$_3$); ^{13}C NMR ($CDCl_3$) δ 149.1 (C^Q), 141.1 (C^Q), 130.6 (C^Q), 127.5 (C^6), 121.4 (C^5), 117.8 (C^7), C^{12} is obscured by the $CDCl_3$ at around 77.0 ppm as confirmed by HSQC, 67.5 (2 x C^{10}), 64.1 (C^3), 58.2 (C^{13}), 53.6 (C^1), 52.7 (2 x C^9), 39.1 (C^4), 29.0 (C^{14}), 22.8 (C^{11}), 20.6 (C^{15}), 14.0 (C^{16}); HRMS (ESI) Exact mass calculated for $C_{19}H_{31}N_2O_2$ [M + H]$^+$ = 319.23800, found: 319.23801; IR (neat) (cm^{-1}): 2957, 1584, 1449, 1373, 1328, 1115, 1045, 958, 795, 728.

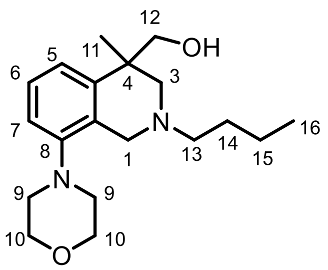

***rac-N*-Butyl-6-methyl-4-methyl-4-hydroxymethyltetrahydroisoquinoline (2u)** The title compound

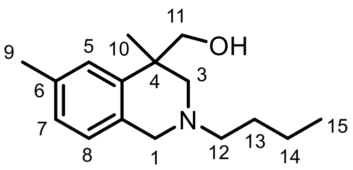

was prepared according to General Procedure G using *N*-butyl-6-methylisoquinolinium iodide (82 mg) and purified by silica gel chromatography (5-20% EtOAc in pentane) to give THIQ **2u** as a colourless oil (26 mg, 42%). ^{1}H NMR (CDCl$_3$) δ 7.15 (1H, s, C^{5}**H**), 7.00 (1H, d, *J* = 8.0 Hz, C$^{7/8}$**H**), 6.95 (1H, d, *J* = 7.8 Hz, C$^{7/8}$**H**), 3.96 (1H, d, *J* = 14.5 Hz, C^{1}**H**$_2$), 3.83 (1H, d, *J* = 9.9 Hz, C^{11}**H**$_2$), 3.67 (1H, dd, *J* = 9.9, 2.8 Hz, C^{11}**H**$_2$), 3.30 (1H, d, *J* = 14.5 Hz, C^{1}**H**$_2$), 3.05 (1H, dd, *J* = 11.4, 1.8 Hz, C^{3}**H**$_2$), 2.51 (1H, td, *J* = 7.0 , 3.8 Hz, C^{12}**H**$_2$), 2.42 (1H, dd, *J* = 11.4, 2.8 Hz, C^{3}**H**$_2$), 2.34 (3H, s, C^{9}**H**$_3$), 1.61 (2H, qd, *J* = 7.1, 6.6, 2.6 Hz, C^{13}**H**$_2$), 1.39 (2H, h, *J* = 7.4 Hz, C^{14}**H**$_2$), 1.16 (3H, s, C^{10}**H**$_3$), 0.96 (3H, t, *J* = 7.3 Hz, C^{15}**H**$_3$); ^{13}C NMR (CDCl$_3$) δ 139.3 (C^{Q}), 136.5 (C^{Q}), 131,2 (C^{Q}), 127.2 (C$^{7/8}$), 126.0 (C^{5} + C$^{7/8}$), 76.3 (C^{11}), 64.3 (C^{3}), 57.9 (C^{12}), 56.5 (C^{1}), 38.6 (C^{4}), 28.8 (C^{13}), 22.4 (C^{10}), 21.3 (C^{9}), 20.5 (C^{14}), 13.9 (C^{15}); HRMS (ESI) Exact mass calculated for $C_{16}H_{26}NO$ [M + H]$^{+}$ = 248.20199, found: 248.20091; IR (neat) (cm^{-1}): 2959, 1513, 1465, 1365, 1253, 1156, 1065, 906, 725, 646,

Mechanistic Experiments

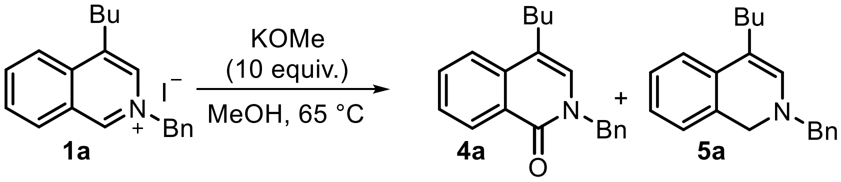

2 hours: 4a:5a 1:1.60
16 hours: 4a:5a 1:1.37

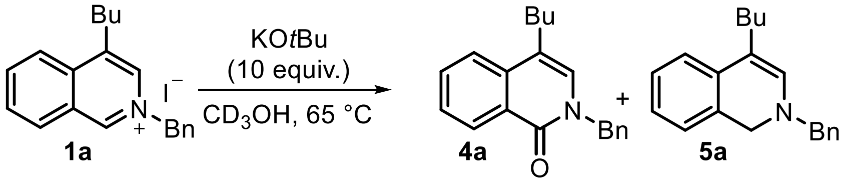

2 hours: 4a:5a 1:1.60
no deuterium incorporation

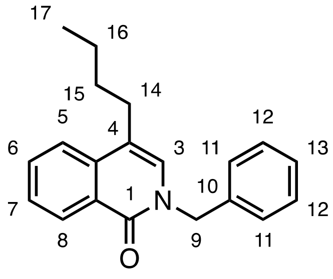

***N*-Benzyl-4-butyl-2-isoquinolinone (4a)** 4-Butylisoquinolinium iodide (40 mg, 0.1 mmol, 1 equiv.) and KOMe (10 equiv.) were heated in MeOH at 65 °C for 16 hours in a sealed microwave vial. The reaction mixture was evaporated to dryness under reduced pressure, quenched with distilled water (10 mL) and extracted in EtOAc (20 mL x 3). The organic layer was dried over anhydrous sodium sulphate, filtered and evaporated under reduced pressure. The crude material was purified by column chromatography (2% MeOH in CH_2Cl_2) to give the title compound **4a** (18 mg, 62%). We suspect that compound **4a** can be formed at least partially by air oxidation of **5a**. ^{1}H NMR ($CDCl_3$) δ 8.53 (dt, *J* = 8.1, 1.0 Hz, 1H, $C^{5/8}$**H**), 7.75 – 7.60 (m, 2H, $C^{5/8}$**H** + $C^{6/7}$**H**), 7.50 (ddd, *J* = 8.2, 6.5, 1.8 Hz, 1H, $C^{6/7}$**H**), 7.38 – 7.27 (m, 5H, 5 x C^{Ar}**H**), 6.89 (d, *J* = 0.9 Hz, 1H, C^3**H**), 5.22 (s, 2H, C^9**H**), 2.72 – 2.50 (m, 2H, C^{14}**H**), 1.66 – 1.51 (m, 2H, C^{15}**H**), 1.39 (h, *J* = 7.3 Hz, 2H, C^{16}**H**), 0.93 (t, *J* = 7.3 Hz, 3H, C^{17}**H**); ^{13}C NMR ($CDCl_3$) δ 161.9 (C^Q), 137.1 (C^Q), 136.7 (C^Q), 132.0 ($C^{5/8}$ or $C^{6/7}$), 128.8 (C^3), 128.8 (2 x C^{Ar}), 128.6 ($C^{5/8}$), 127.8 (2 x C^{Ar}), 127.7 (C^{Ar}), 126.6 ($C^{6/7}$), 126.4 (C^Q), 122.9 ($C^{5/8}$ or $C^{6/7}$), 116.9 (C^Q), 51.6 (C^9), 31.6 (C^{15}), 29.2 (C^{14}), 22.6 (C^{16}), 13.9 (C^{17}); HRMS (ESI): Exact mass calculated for $C_{20}H_{22}NO$ $[M + H]^+$ = 292.1696, found: 292.1698; IR (neat) (cm^{-1}): 3042, 2989, 1705, 1692, 1680, 1504, 1385, 812, 780, 668.

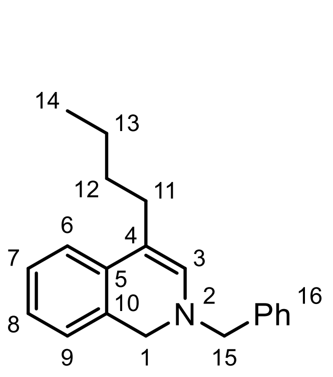

***N*-Benzyl-4-butyl-2-dihydroisoquinolinine (5a)** The crude NMR indicated the presence of compound **5a** with the following NMR spectra: ^{1}H NMR (CD_3OD) δ 7.36-7.34 (5H, m, 5 x Ar^{62}**H**), 7.13 (1H, td, *J* = 7.5, 1.4 Hz, $C^{7/8}$**H**), 7.01-6.98 (1H, m, $C^{7/8}$**H**), 6.96 (1H, dd, *J* = 7.2, 1.3 Hz, $C^{6/9}$**H**), 6.88 (1H, ddd, *J* = 7.5, 1.6, 0.8 Hz, $C^{6/9}$**H**), 6.16 (1H, s, C^3**H**), 4.10 (2H, s, $C^{1/15}$**H_2**), 3.97 (2H, s, $C^{1/15}$**H**), 2.32 (2H, t, *J* = 7.3 Hz, C^{11}**H_2**), 1.54-1.38 (4H,

m, $C^{12}\mathbf{H}_2$ + $C^{13}\mathbf{H}_2$), 0.95 (3H, t, *J* = 7.3 Hz, $C^{14}\mathbf{H}_3$), which correlated well with related compounds reported by others.[10]

[10] M. Kihara, J. Andoh, C. Yoshida, *Heterocycles*, **2000**, *53*, 359-372.

Palladium Scavenging Experiments

Two substrates were treated with Biotage ISOLUTE SI-TMT scavenging resin, particularly effective at removing trace quantities of palladium, rhodium, ruthenium, nickel, and platinum. Following treatment, these compounds showed no change of reactivity

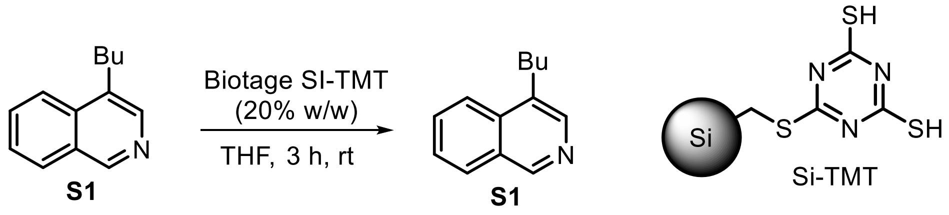

4-Butylisoquinoline **S1** (200 mg) was dissolved in THF (20 mL) and Biotage ISOLUTE Si-TMT scavenging resin (40 mg, 20% w/w) was added and the solution was stirred at room temperature for 3 h. The solution was filtered and concentrated *in vacuo* to return "*purified*" **S1**. This material was reacted with benzyl iodide (0.2 mL) to form salt **1a** which was subjected to the standard reaction conditions and gave **2a** in 65% (16 hours) and 49% (6 hours) indicating no loss of reactivity.

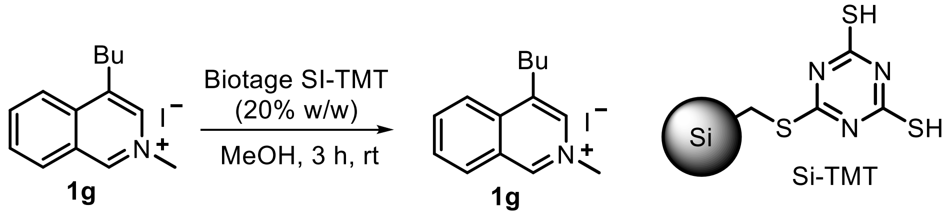

N-Methyl-4-butylisoquinolinium iodide **1g** (400 mg) was dissolved in MeOH (20 mL) and Biotage ISOLUTE Si-TMT scavenging resin (80 mg, 20% w/w) was added and the solution was stirred at room temperature for 3 h. The solution was filtered and concentrated *in vacuo* to return "*purified*" **1g**. This material was subjected to the standard reaction conditions and gave **2g** in 70% (16 hours) indicating no loss of reactivity.

ICP-MS analysis of salt **1a** and **1g** before and after treatment with Pd-scavenging resins determined that in all cases the Pd-contamination was less than 5 ppm (the detection limit of the machine).

Inductively Coupled Plasma Mass Spectrometry of KOMe (ICP-MS)

Sample Preparation: To a solution of potassium methoxide (2 g) in MeOH, was added HCl (2-3 equiv., 2M in Et_2O). The reaction was stirred for 10 minutes then concentrated *in vacuo* to furnish potassium chloride. Potassium chloride (1 g) was dissolved in 18 megohm water (10 ml), 1 mL of this solution was further diluted in 18 megohm water (10 mL).

The ICP-MS was undertaken by Phil Holdship (Department of Earth Science, University of Oxford).

The potassium methoxide sample (purchased from Sigma Aldrich) gave the following trace metal quantities:

Platinum Metals: Ru (0 ppm), Rh (0.037 ppm), Pd (0.141 ppm), Ir (0.681 ppm), Pt (0 ppm), Au (1.87 ppm).

Other: V (12.6 ppm), Cr (11.1 ppm), Mn (1.5 ppm), Cu (0.27 ppm), Zn (23.3 ppm), Ga (0.23 ppm), Zr (0.68 ppm), Mo (0.80 ppm), Sc (0.24 ppm), Rb (214.6 ppm), Sr (3.8 ppm), Ce (0.012 ppm), Pr (0.005 ppm), Nd (0.14 ppm), Eu (0.004 ppm), Dy (0.009 ppm), Er (0.005 ppm) Ti (1.6 ppm), Se (8.3 ppm), Cd (0.097 ppm), Sn (0.28 ppm), Pb (1.51 ppm).

The following metals were tested for and found not to be present: Co, Ge, Nb, As, Y, La, Sm, Gd, Tb, Ho, Tm, Yb, Lu, U, W, Re.

Deuterium Labelling Studies

d[4]-MeOH and CH_3OD were purchased from Sigma Aldrich and Cambridge Isotope Laboratories respectively and used without further purification.

d[2]-Paraformaldehyde was purchased from Sigma Aldrich and used without further purification.

All reactions were performed following General Procedure F, using deuterated reagents where specified in the reaction scheme.

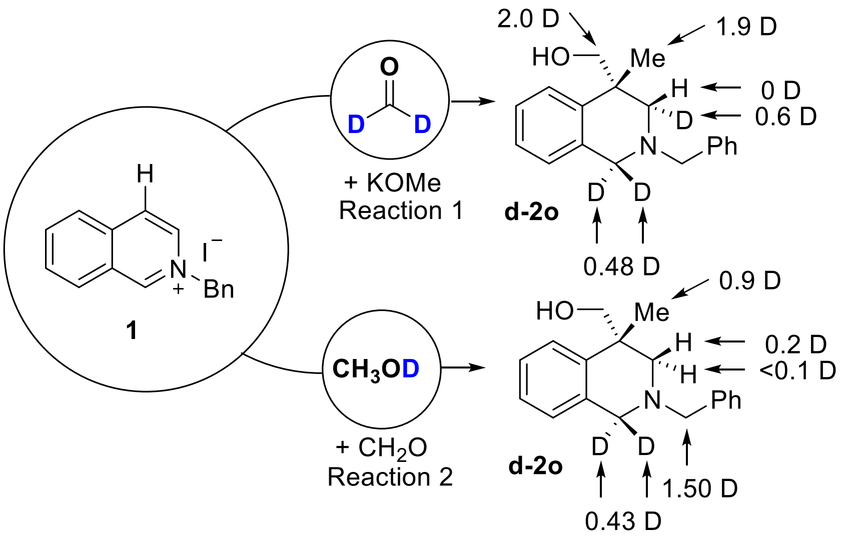

Tandem Methylation-Hydroxymethylation Mechanism

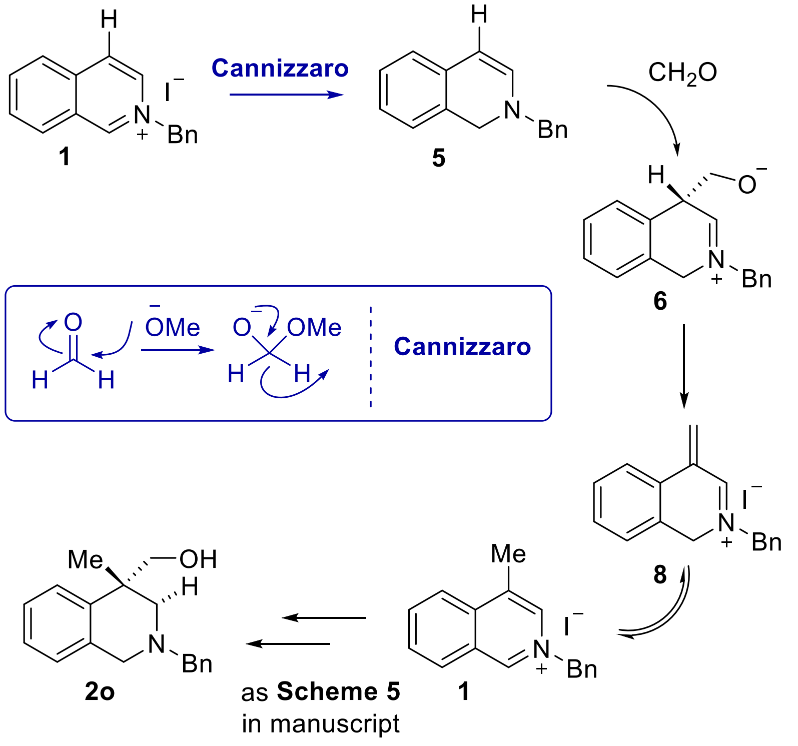

Scheme S1: Tandem methylation-hydroxymethylation reaction

To further understand the tandem methylation-hydroxymethylation process, the standard reaction but using paraformaldehyde-d^2 instead was undertaken (Reaction 1, Scheme S1) and **d-2o** was isolated with deuterium incorporation in the C1 (0.96D), one proton at the C3 (0.6D), the CH_2OH (2.0D), and the methyl (1.9D) positions. Similarly, when using CH_3OD in place of MeOH (Reaction 2, Scheme S1) deuterium incorporation was observed in the C1 (0.86D), benzylic (1.50D), C3 (0.3D) and methyl (0.9D) positions. In line with these results, we propose the following mechanism for tandem process (Scheme S1): Reduction of **1** and the trapping of formaldehyde, in the same manner as Scheme 5 in the manuscript, leads to the formation of intermediate **6**. At this point, elimination of -OH leads to exocyclic alkene **8** which can undergo an isomerization to form *N*-benzyl-4-methylisoquinolinum iodide **1** which then participates in the identical mechanism explained in Scheme 5 in the manuscript to furnish the methylated-hydroxymethylated product **2o**. This mechanism is consistent with our deuterium studies, particularly the presence of ≈1 proton in the methyl group when paraformalhyde-d^2 is used and ≈1 deuterium in the methyl group when CH_3OD is used.

Deuterium NMR Spectra

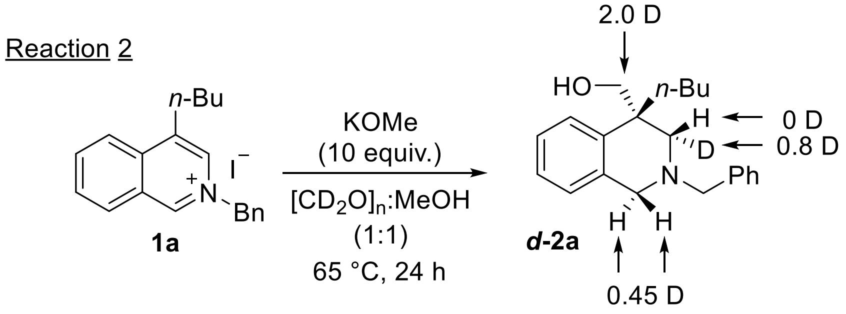

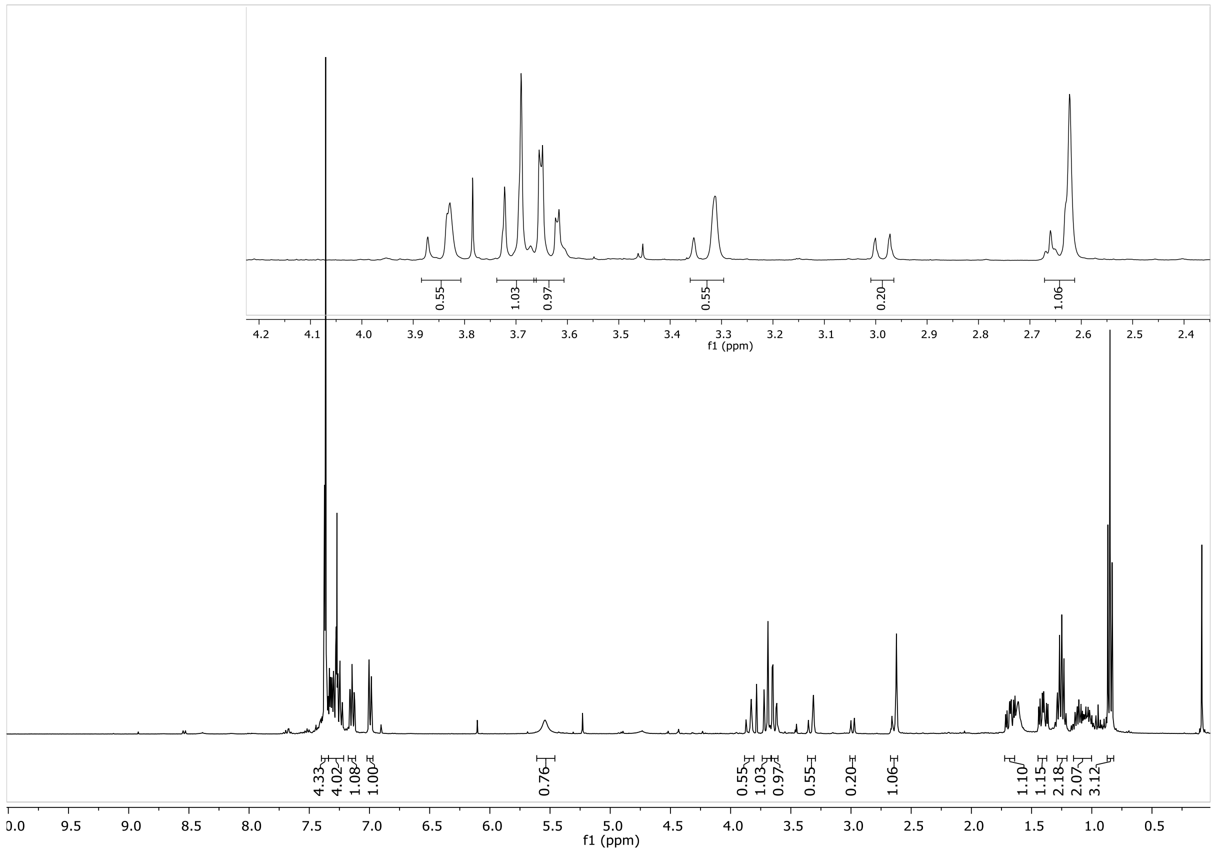

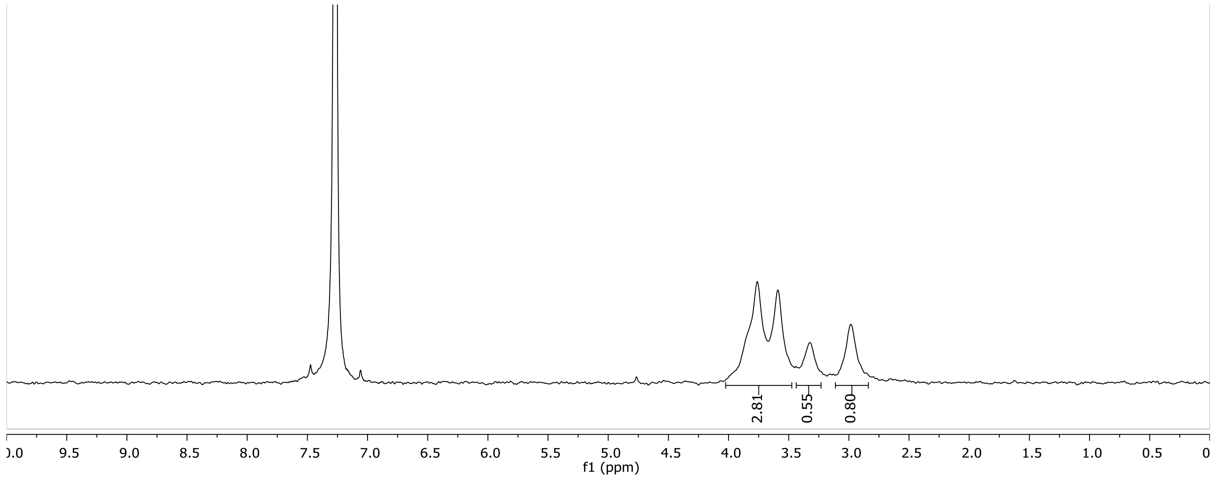

Reaction 3

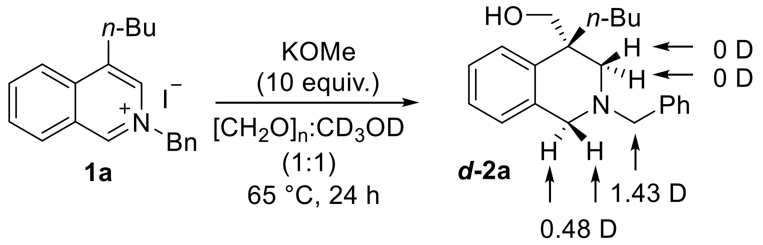

n-Bu
I⁻
N
Bn
1a
KOMe
(10 equiv.)
[CH2O]n:CD3OD
(1:1)
65 °C, 24 h
HO
n-Bu
H
H
N
Ph
0 D
0 D
d-2a
H
H
1.43 D
0.48 D

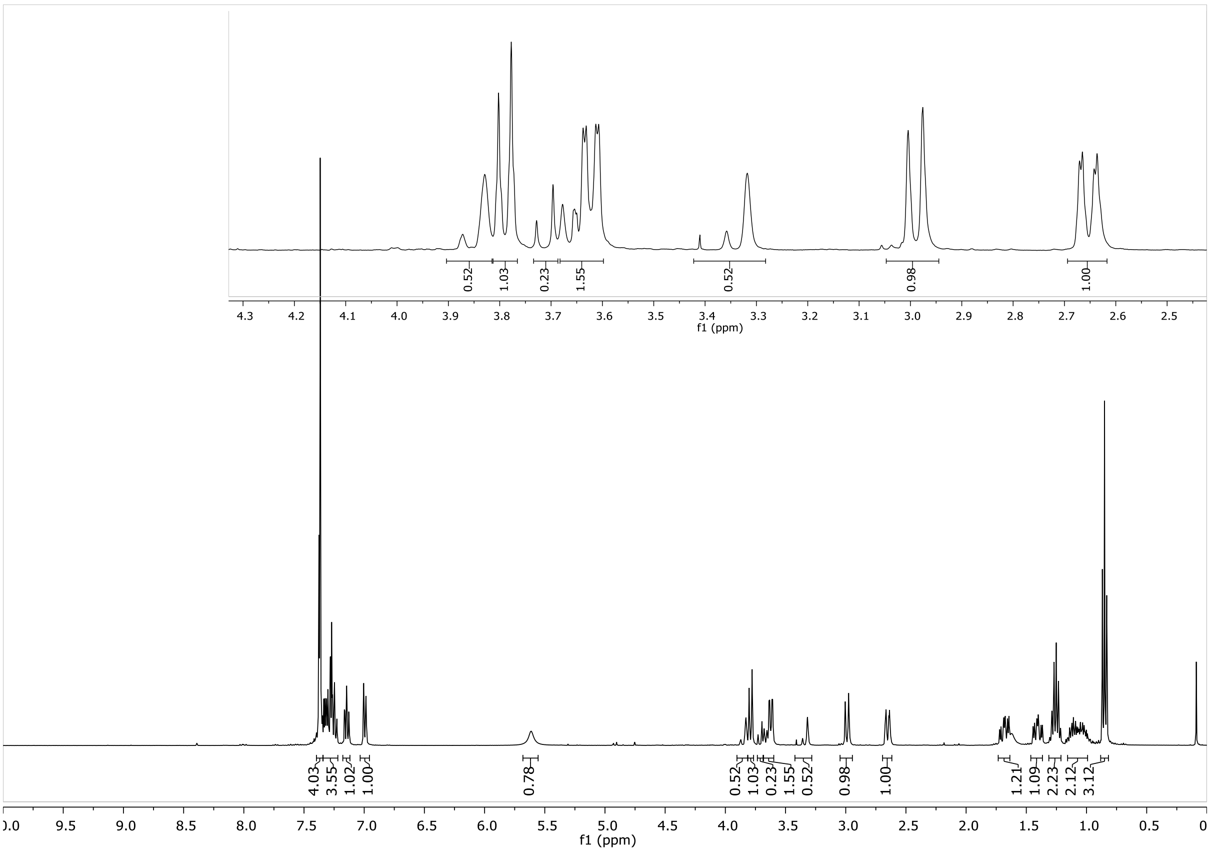

f1 (ppm)

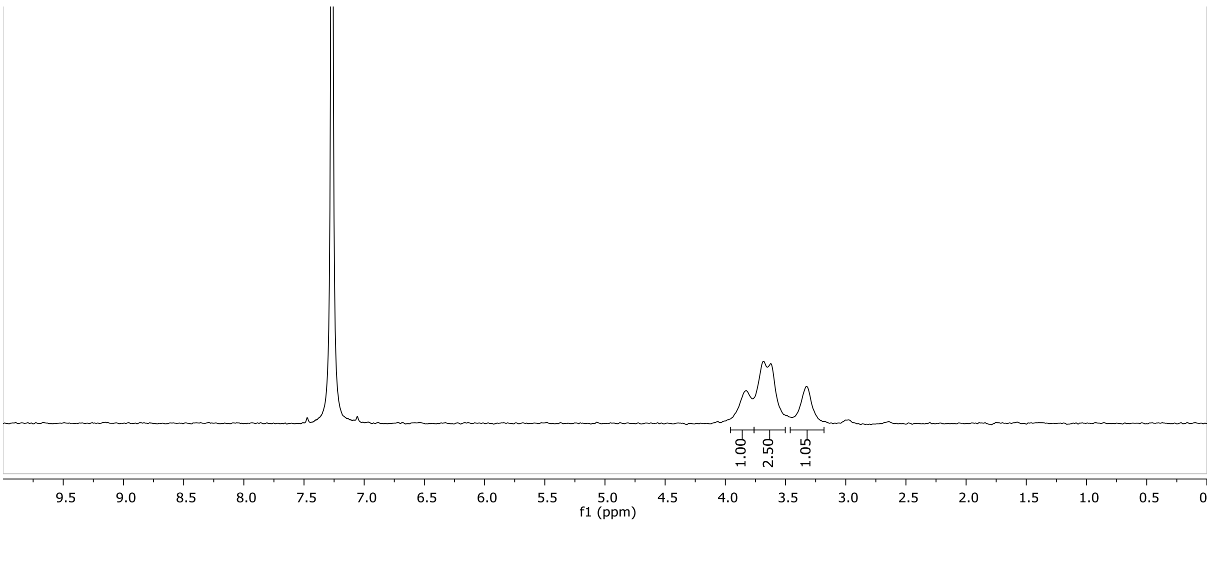

f1 (ppm)

Reaction 4

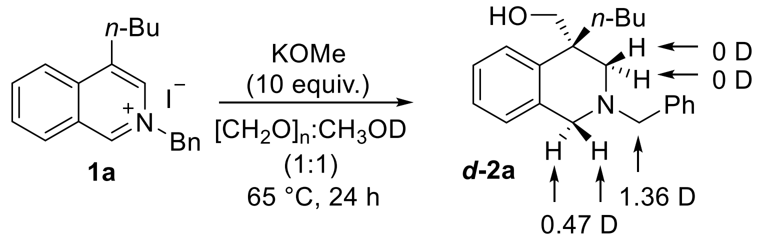
n-Bu
I⁻
N
Bn
1a
KOMe
(10 equiv.)
[CH2O]n:CH3OD
(1:1)
65 °C, 24 h
HO
n-Bu
H ← 0 D
H ← 0 D
N
Ph
d-2a
H
H
1.36 D
0.47 D

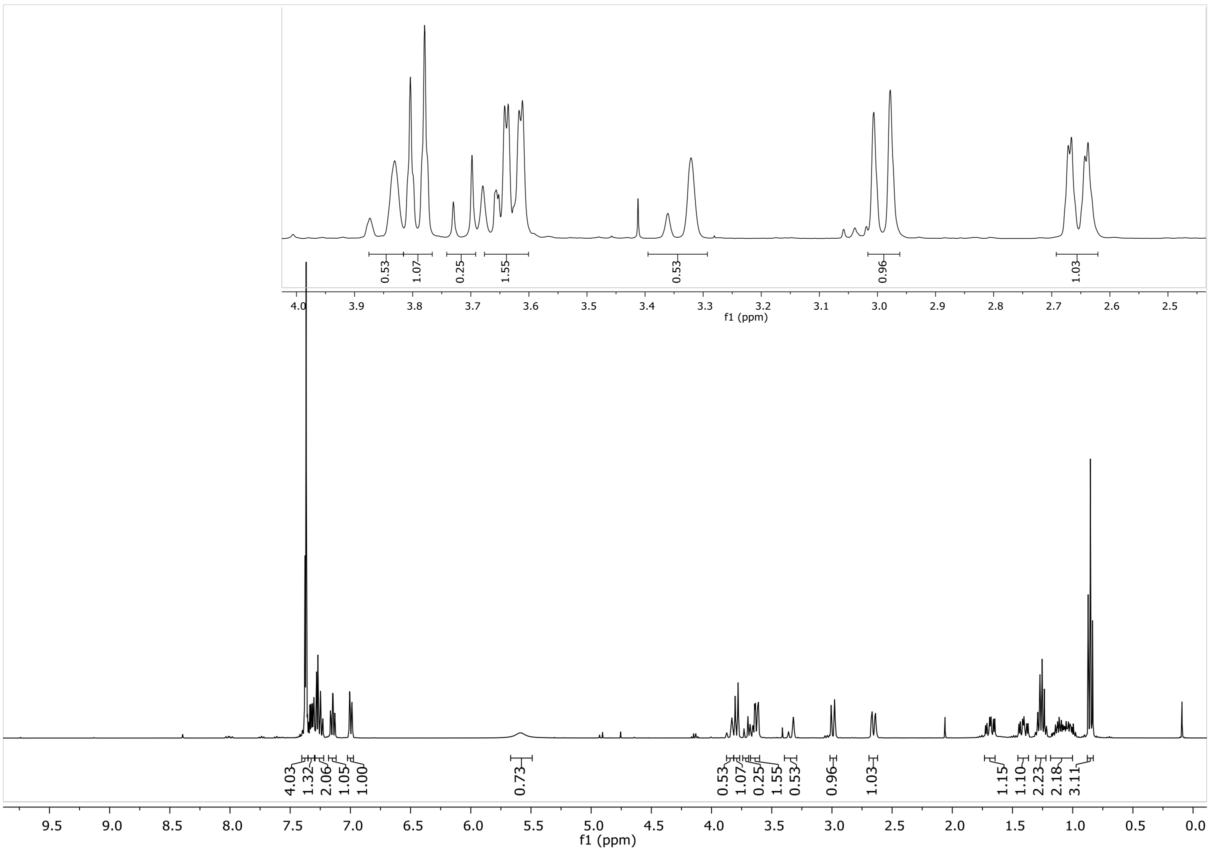
0.53
1.07
0.25
1.55
0.53
0.96
1.03
f1 (ppm)
4.03
1.32
2.06
1.05
1.00
0.73
0.53
1.07
0.25
1.55
0.53
0.96
1.03
1.15
1.10
2.23
2.18
3.11
f1 (ppm)

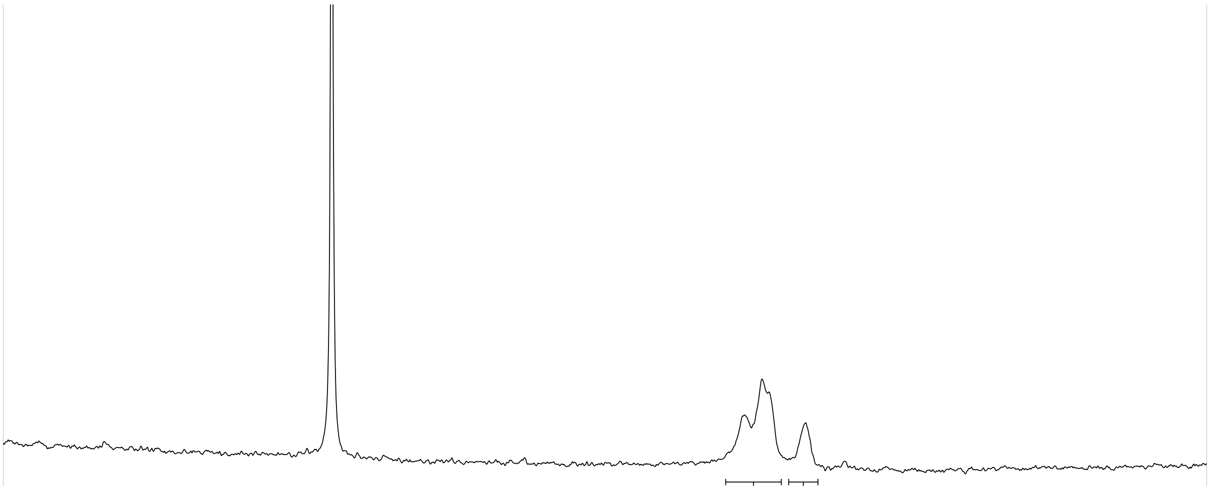
1.76
-1.00
f1 (ppm)

Reaction 5

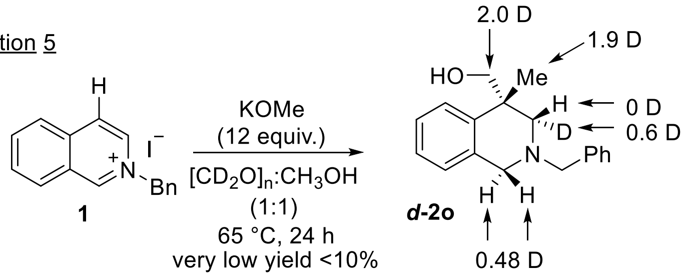

1
KOMe
(12 equiv.)
$[CD_2O]_n$:CH_3OH
(1:1)
65 °C, 24 h
very low yield <10%
d-2o
2.0 D
1.9 D
0 D
0.6 D
0.48 D

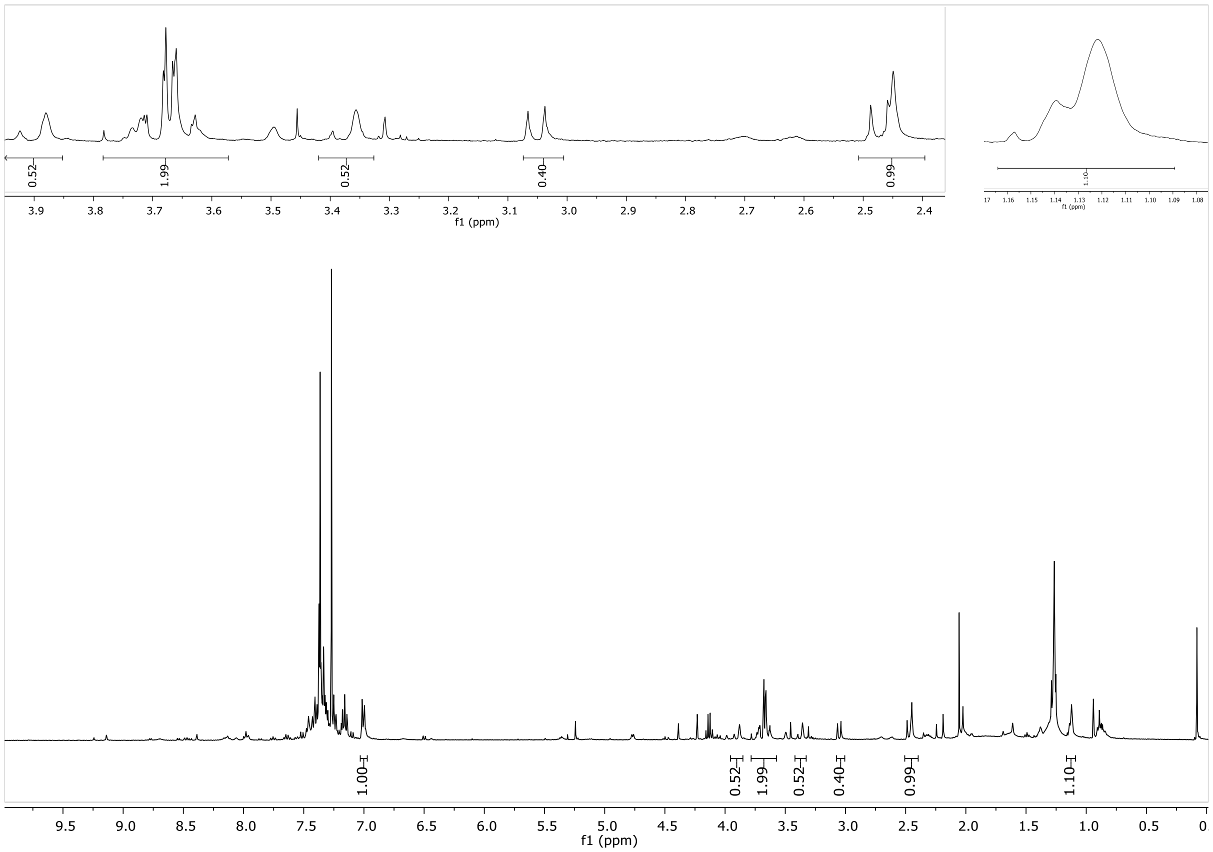

f1 (ppm)

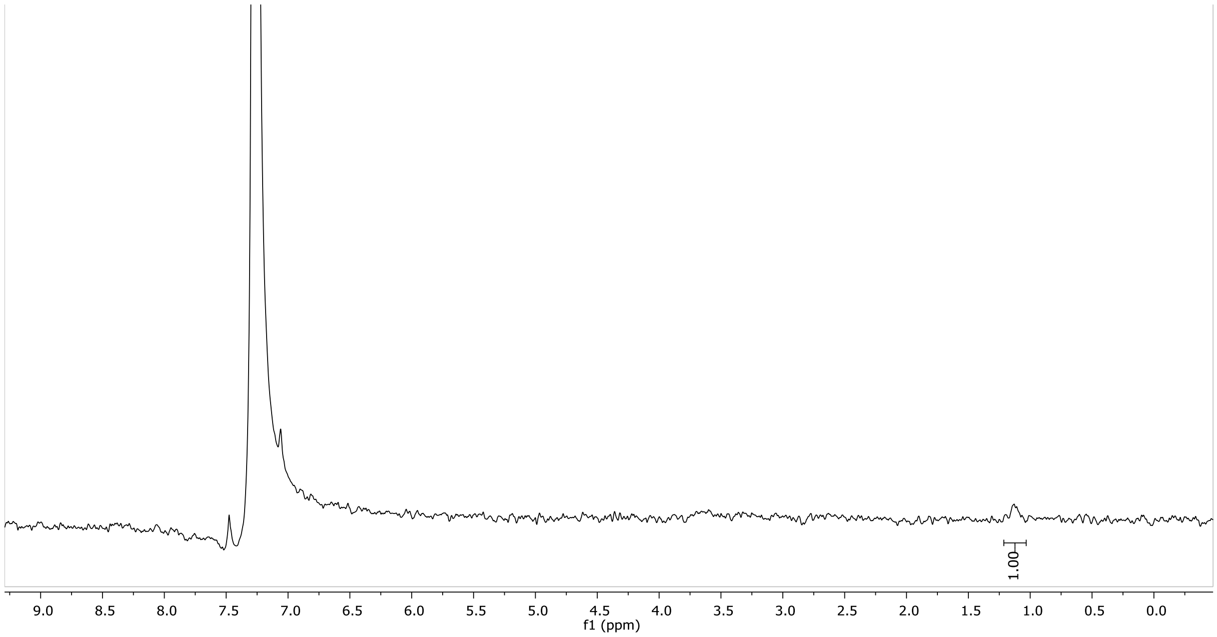

f1 (ppm)

Reaction 6

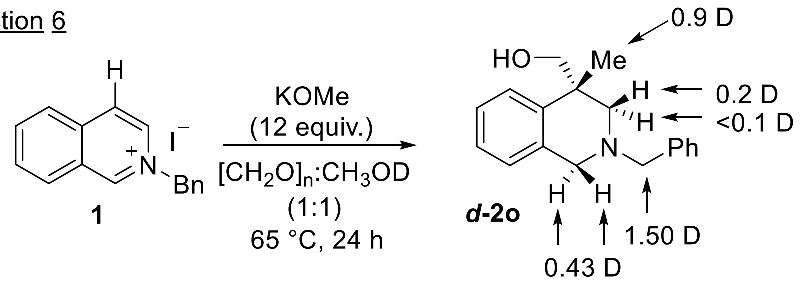

H
I⁻
N
Bn
1
KOMe
(12 equiv.)
[CH2O]n:CH3OD
(1:1)
65 °C, 24 h
HO
Me
0.9 D
H
0.2 D
H
<0.1 D
N
Ph
H
H
1.50 D
0.43 D
d-2o

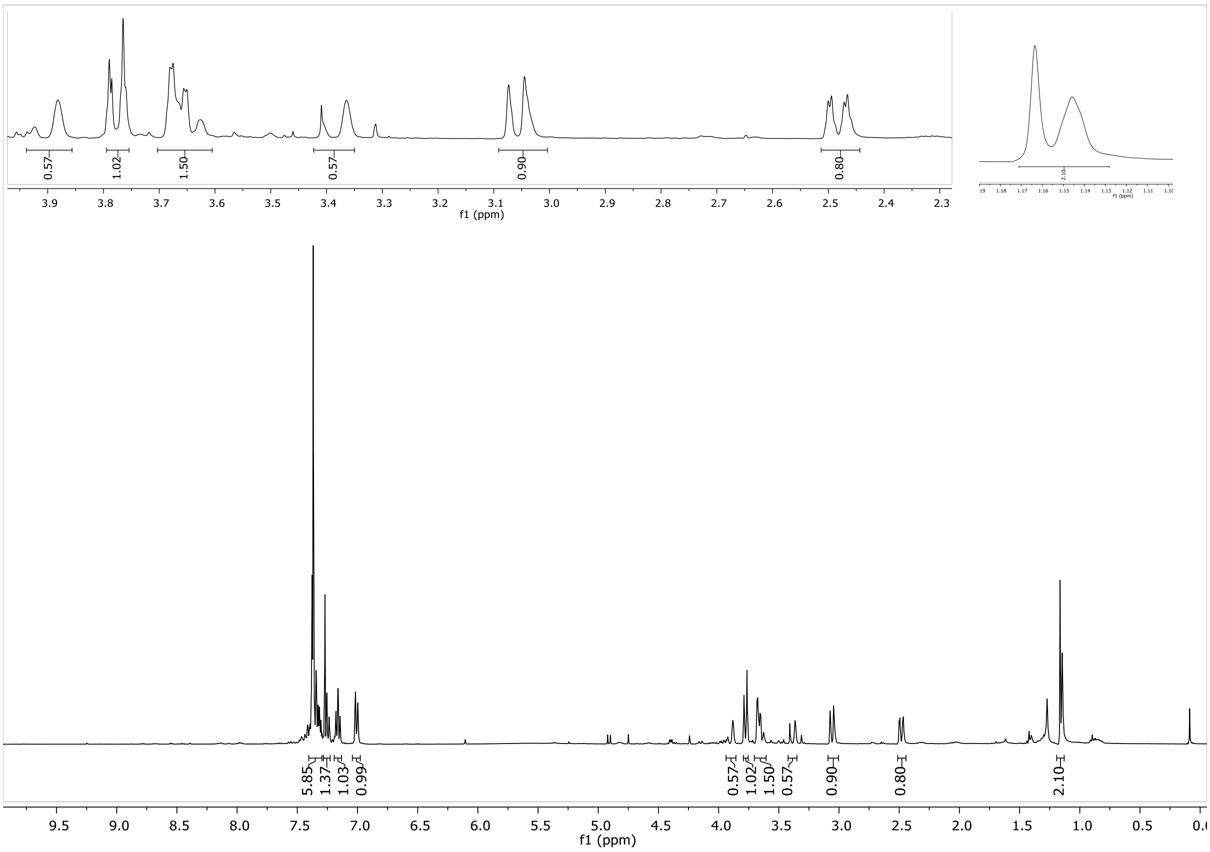

f1 (ppm)

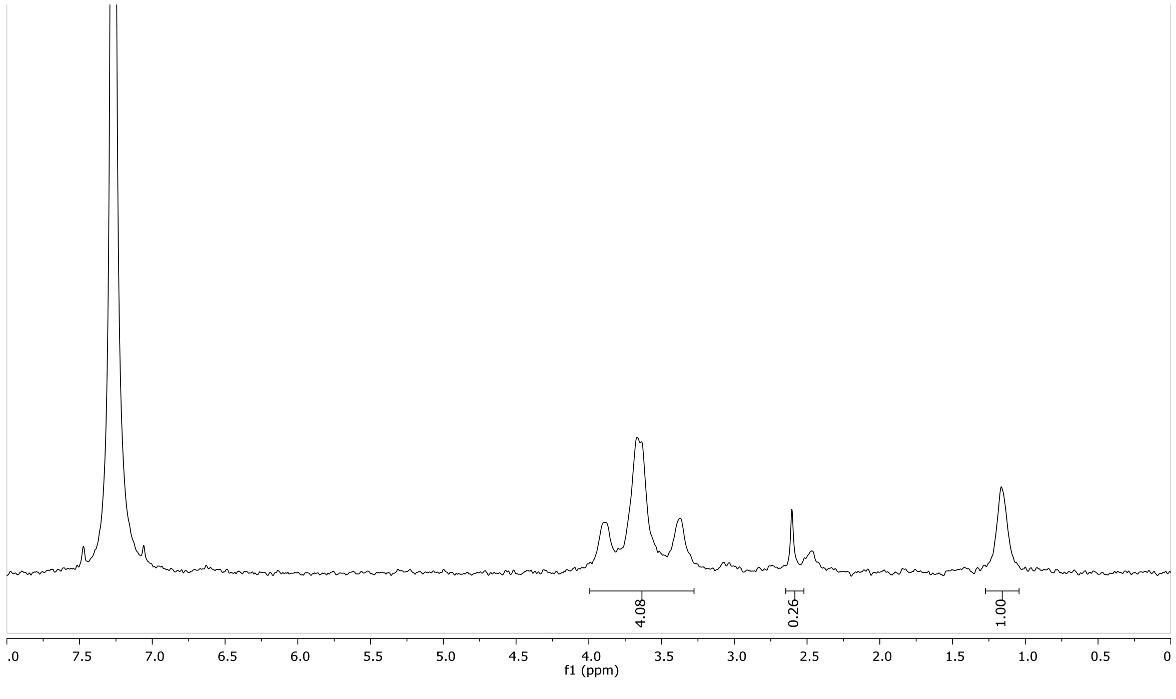

f1 (ppm)

NMR Spectra

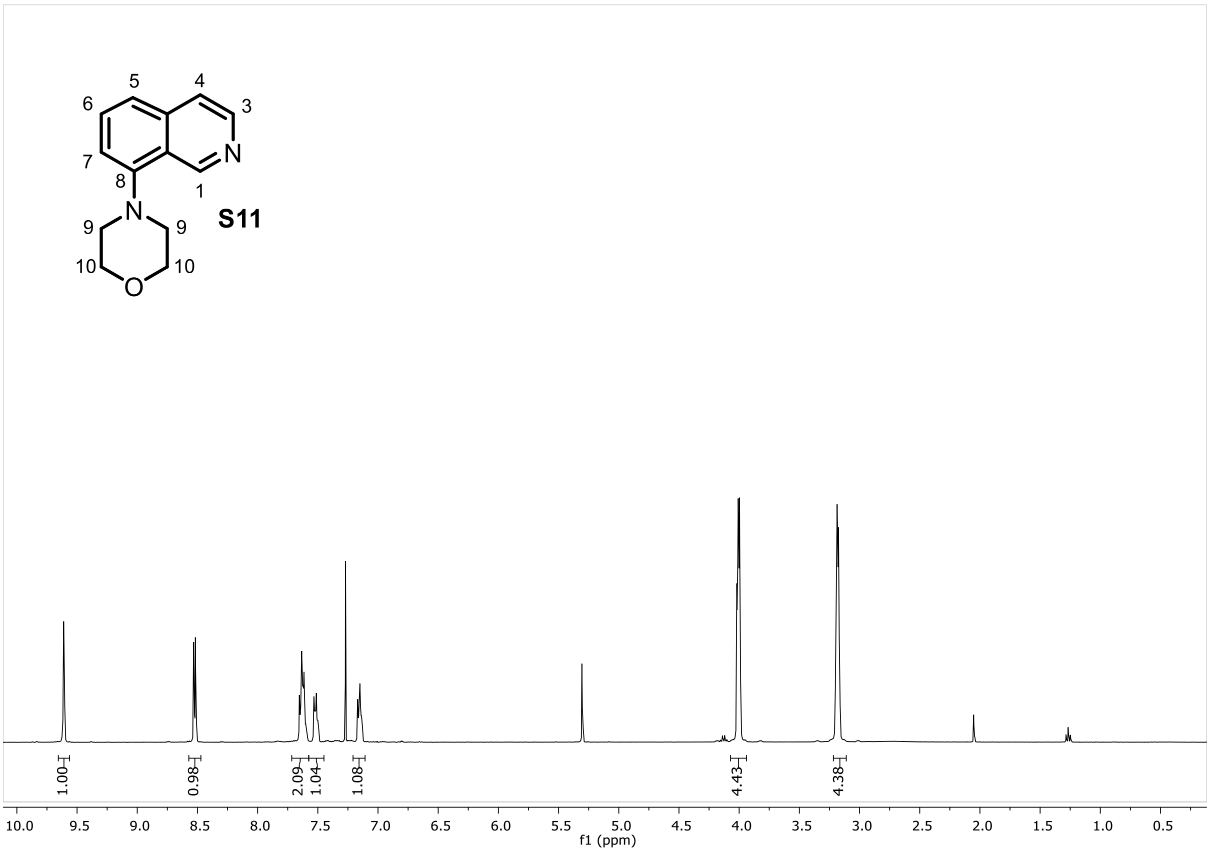
S11
1.00
0.98
2.09
1.04
1.08
4.43
4.38
10.0 9.5 9.0 8.5 8.0 7.5 7.0 6.5 6.0 5.5 5.0 4.5 4.0 3.5 3.0 2.5 2.0 1.5 1.0 0.5
f1 (ppm)

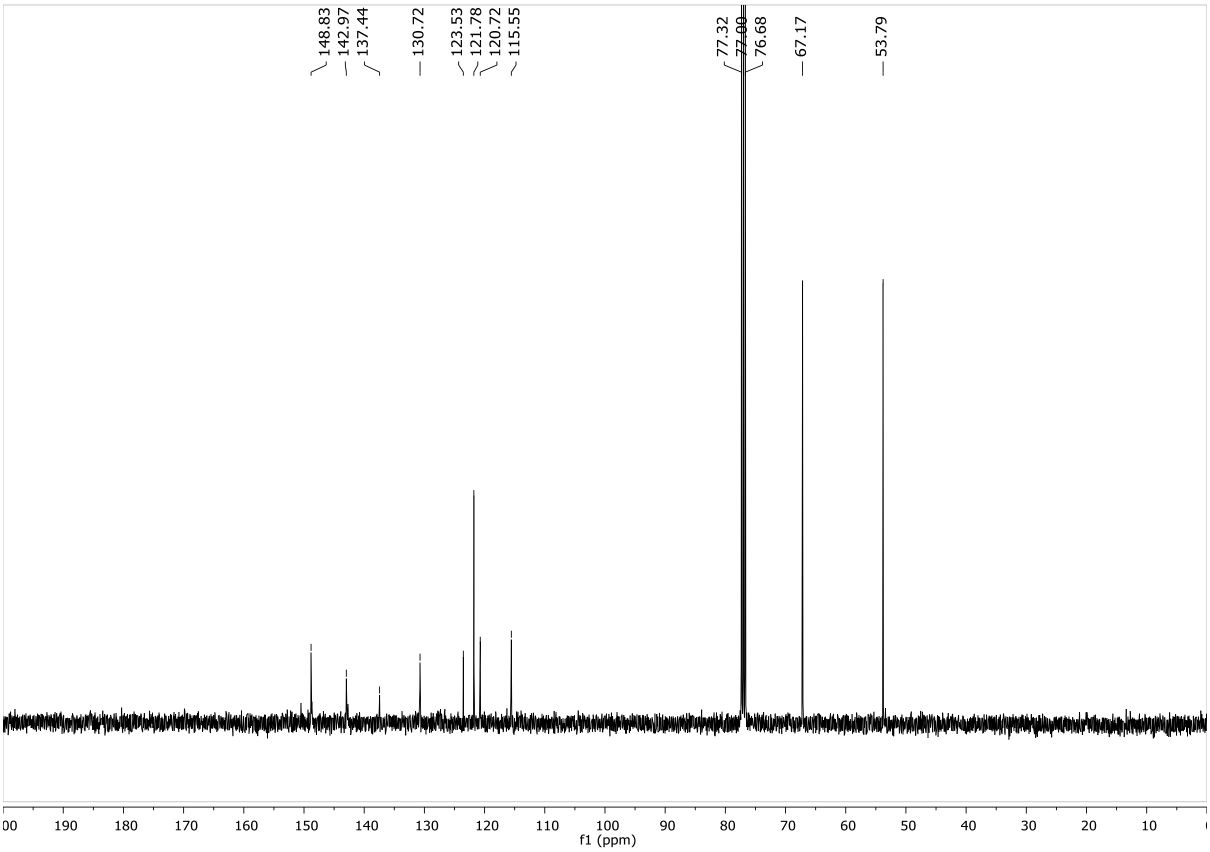
148.83
142.97
137.44
130.72
123.53
121.78
120.72
115.55
77.32
77.00
76.68
67.17
53.79
f1 (ppm)

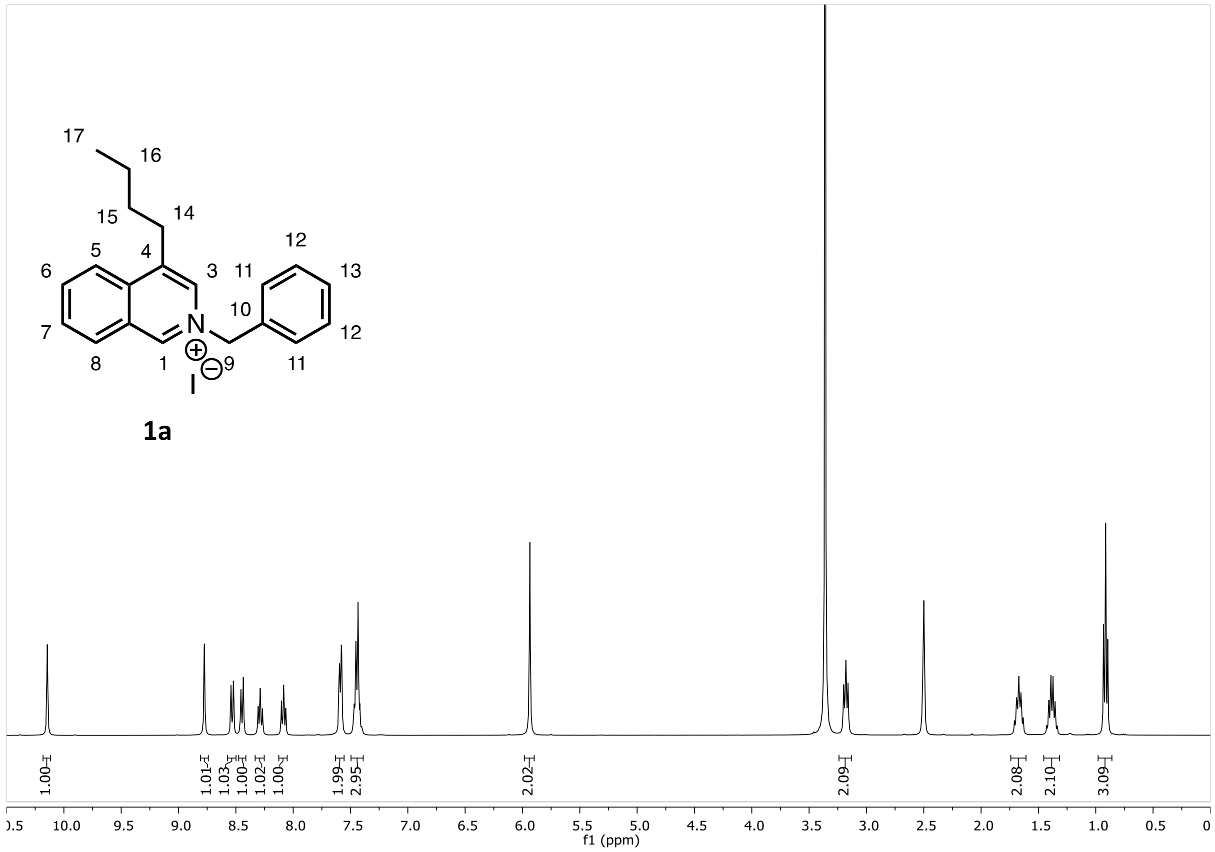
1a
1.00
1.01
1.03
1.00
1.02
1.00
1.99
2.95
2.02
2.09
2.08
2.10
3.09
f1 (ppm)

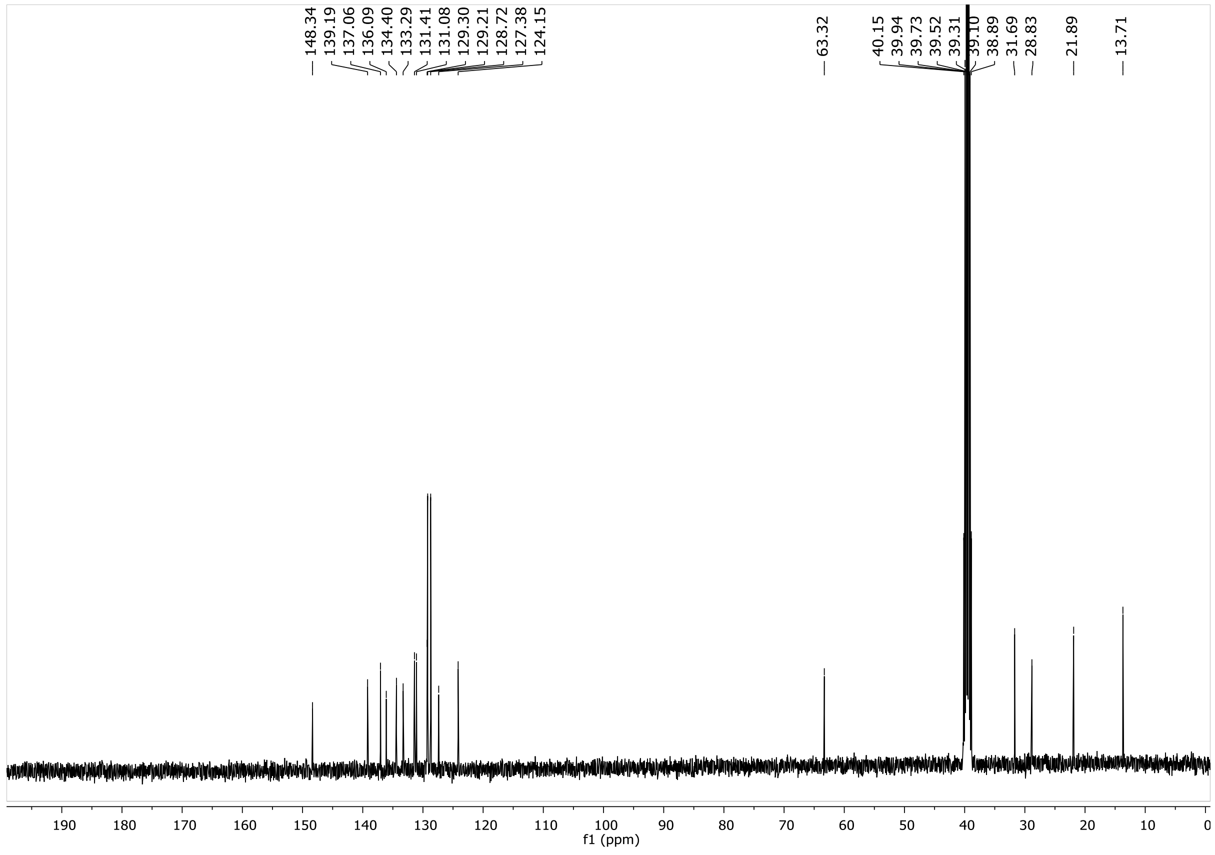
148.34
139.19
137.06
136.09
134.40
133.29
131.41
131.08
129.30
129.21
128.72
127.38
124.15
63.32
40.15
39.94
39.73
39.52
39.31
39.10
38.89
31.69
28.83
21.89
13.71
f1 (ppm)

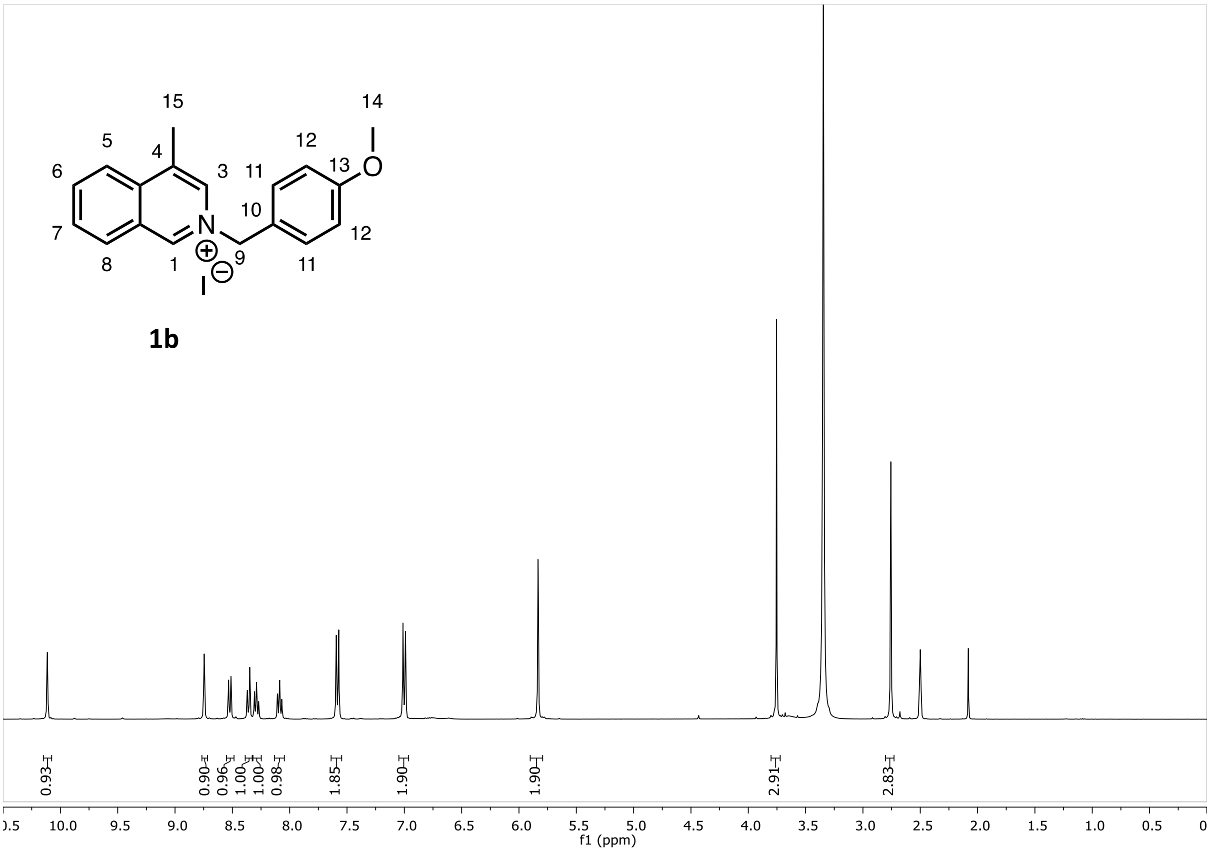
15
14
5
4
12
6
3
11
13
O
N
10
7
12
8
1
9
11
I
1b
0.93
0.90
0.96
1.00
1.00
0.98
1.85
1.90
1.90
2.91
2.83
10.0 9.5 9.0 8.5 8.0 7.5 7.0 6.5 6.0 5.5 5.0 4.5 4.0 3.5 3.0 2.5 2.0 1.5 1.0 0.5 0
f1 (ppm)

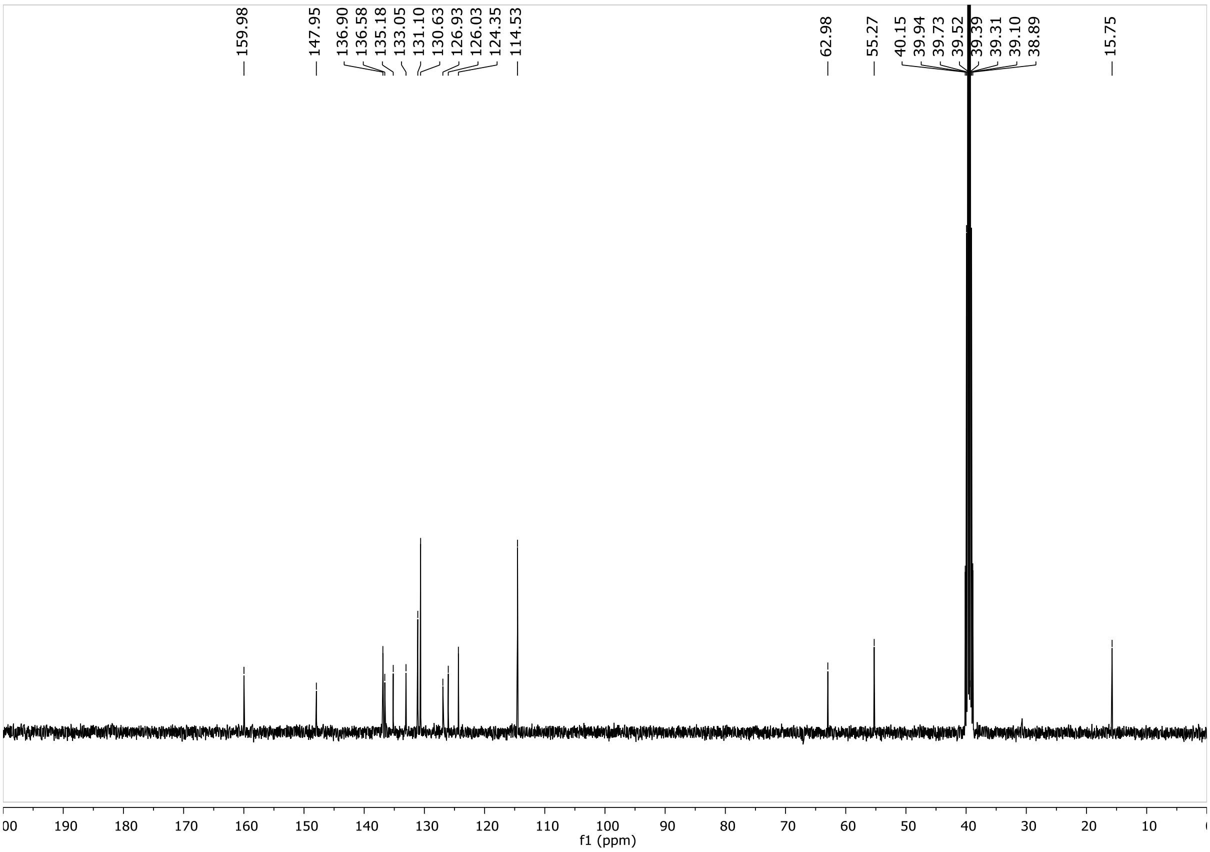
159.98
147.95
136.90
136.58
135.18
133.05
131.10
130.63
126.93
126.03
124.35
114.53
62.98
55.27
40.15
39.94
39.73
39.52
39.39
39.31
39.10
38.89
15.75
190 180 170 160 150 140 130 120 110 100 90 80 70 60 50 40 30 20 10
f1 (ppm)

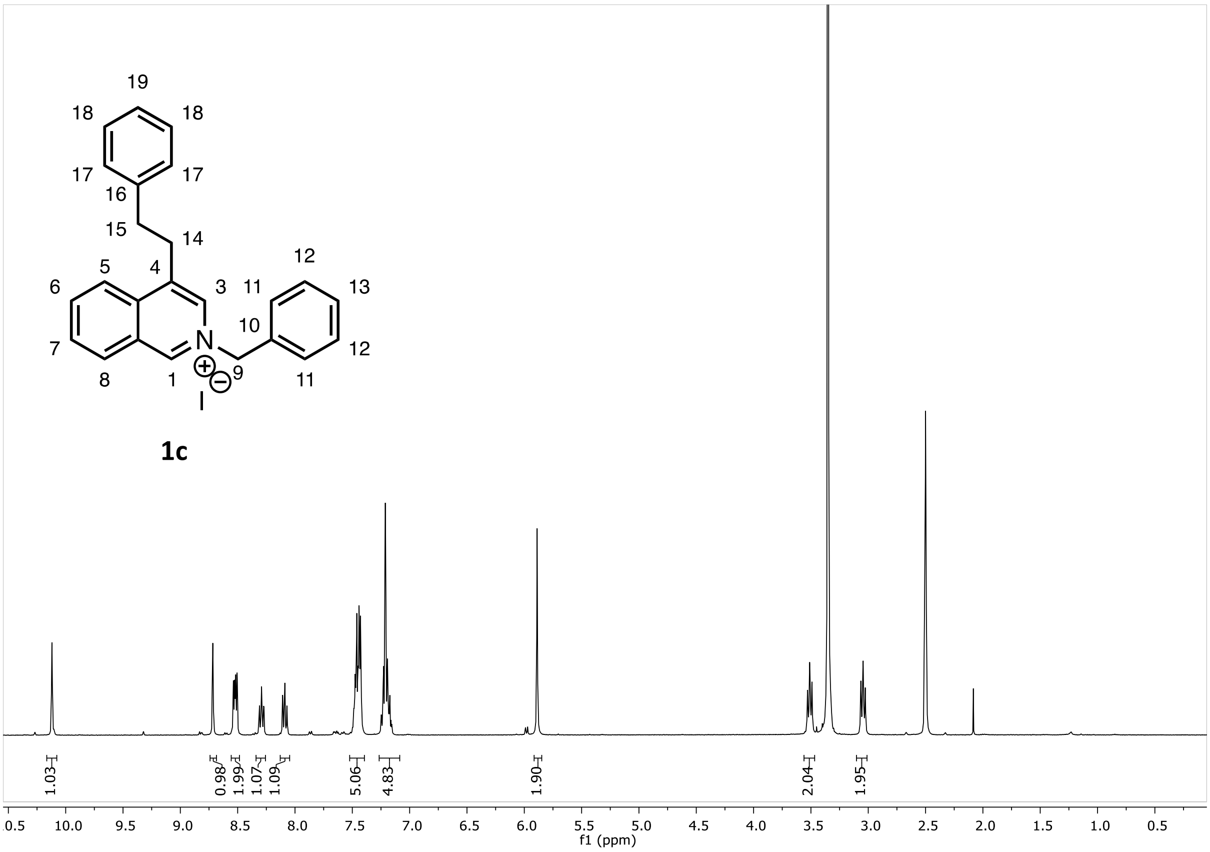

19
18
18
17
17
16
15
14
5
4
6
3
11
12
13
10
7
12
9
8
1
11
I
1c
1.03
0.98
1.99
1.07
1.09
5.06
4.83
1.90
2.04
1.95
10.0 9.5 9.0 8.5 8.0 7.5 7.0 6.5 6.0 5.5 5.0 4.5 4.0 3.5 3.0 2.5 2.0 1.5 1.0 0.5
f1 (ppm)

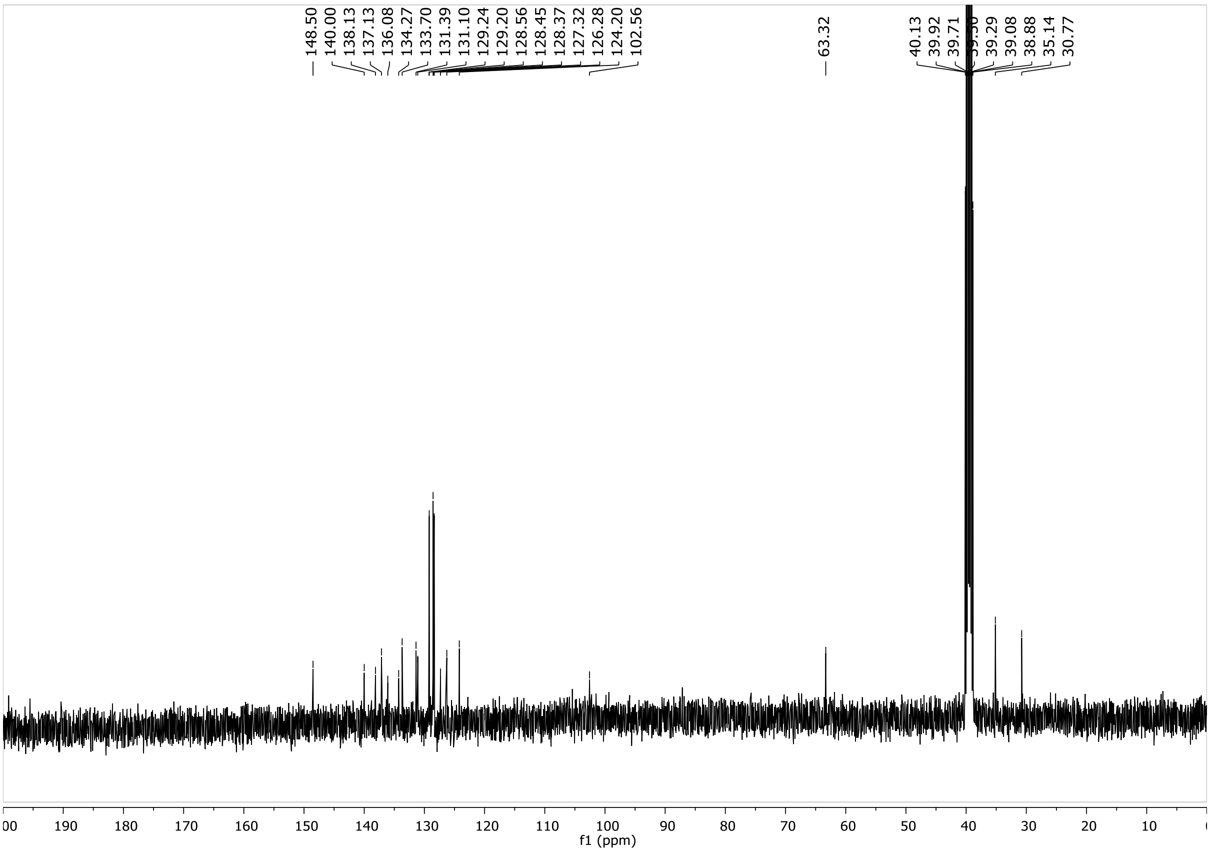

148.50
140.00
138.13
137.13
136.08
134.27
133.70
131.39
131.10
129.24
129.20
128.56
128.45
128.37
127.32
126.28
124.20
102.56
63.32
40.13
39.92
39.71
39.29
39.08
38.88
35.14
30.77
190 180 170 160 150 140 130 120 110 100 90 80 70 60 50 40 30 20 10
f1 (ppm)

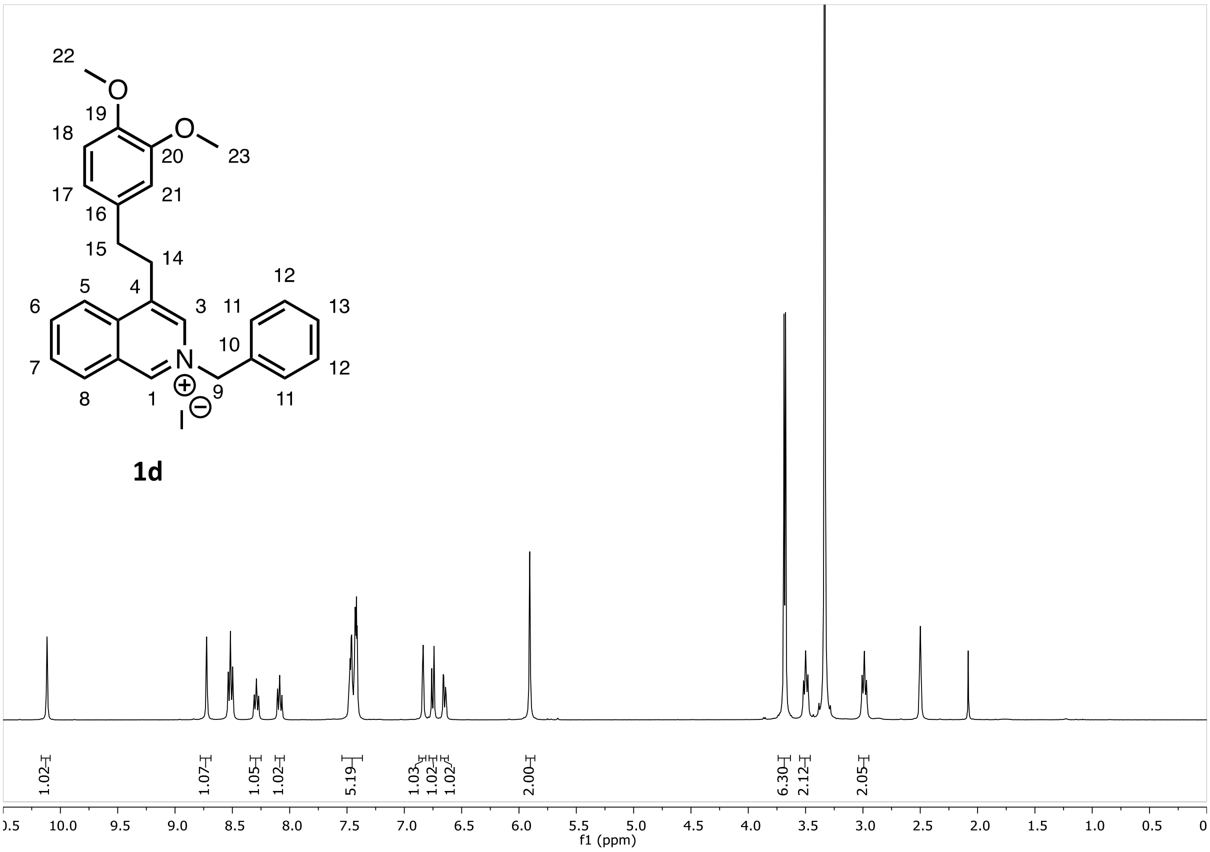

1d

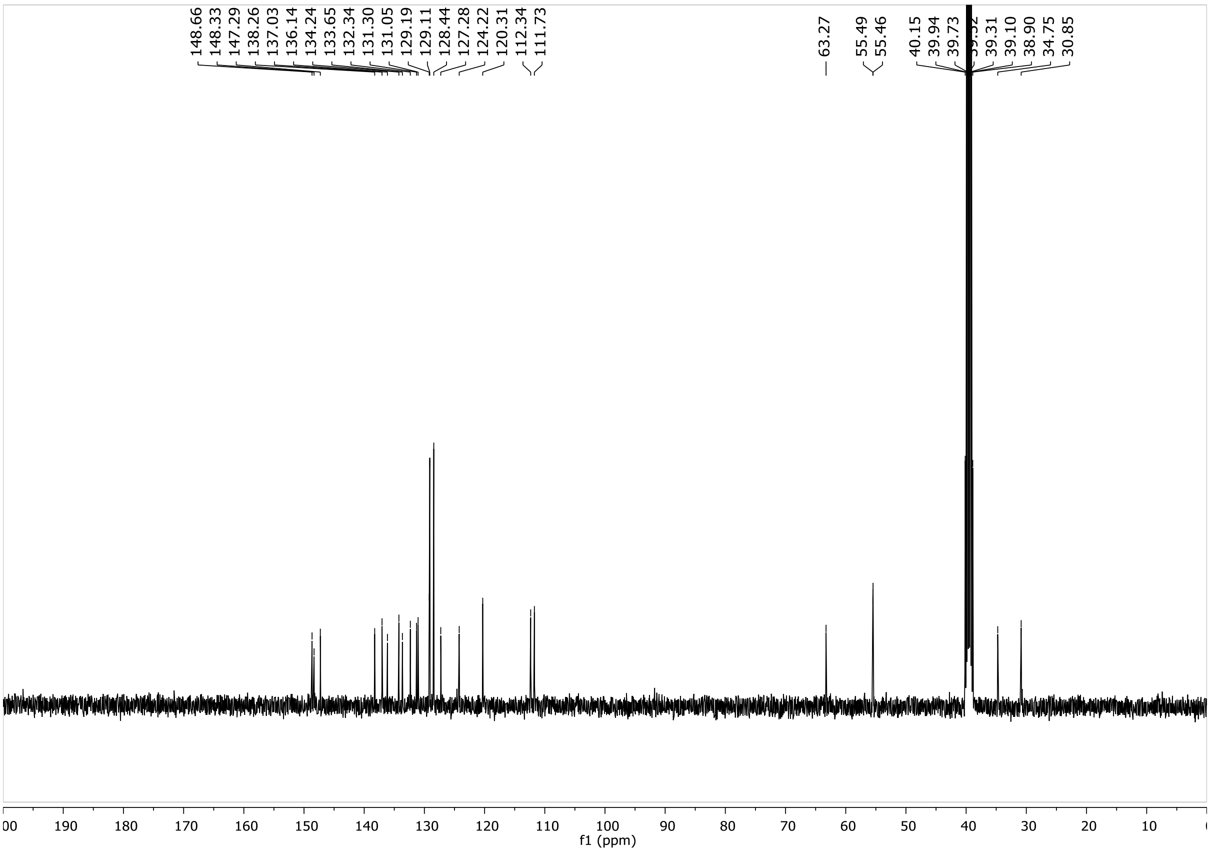

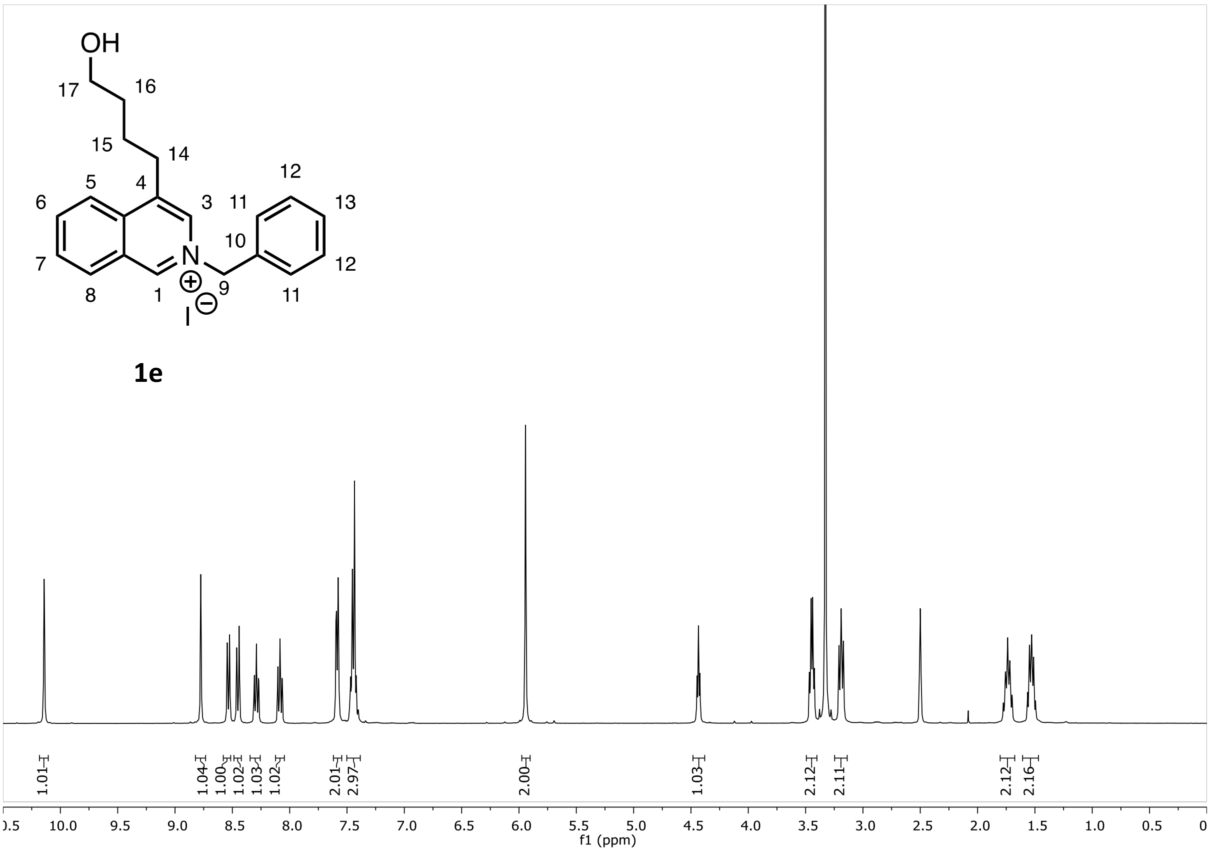
OH
17
16
15
14
5
4
6
3
11
12
13
10
7
12
8
1
9
11
I
1e
1.01
1.04
1.00
1.02
1.03
1.02
2.01
2.97
2.00
1.03
2.12
2.11
2.12
2.16
f1 (ppm)

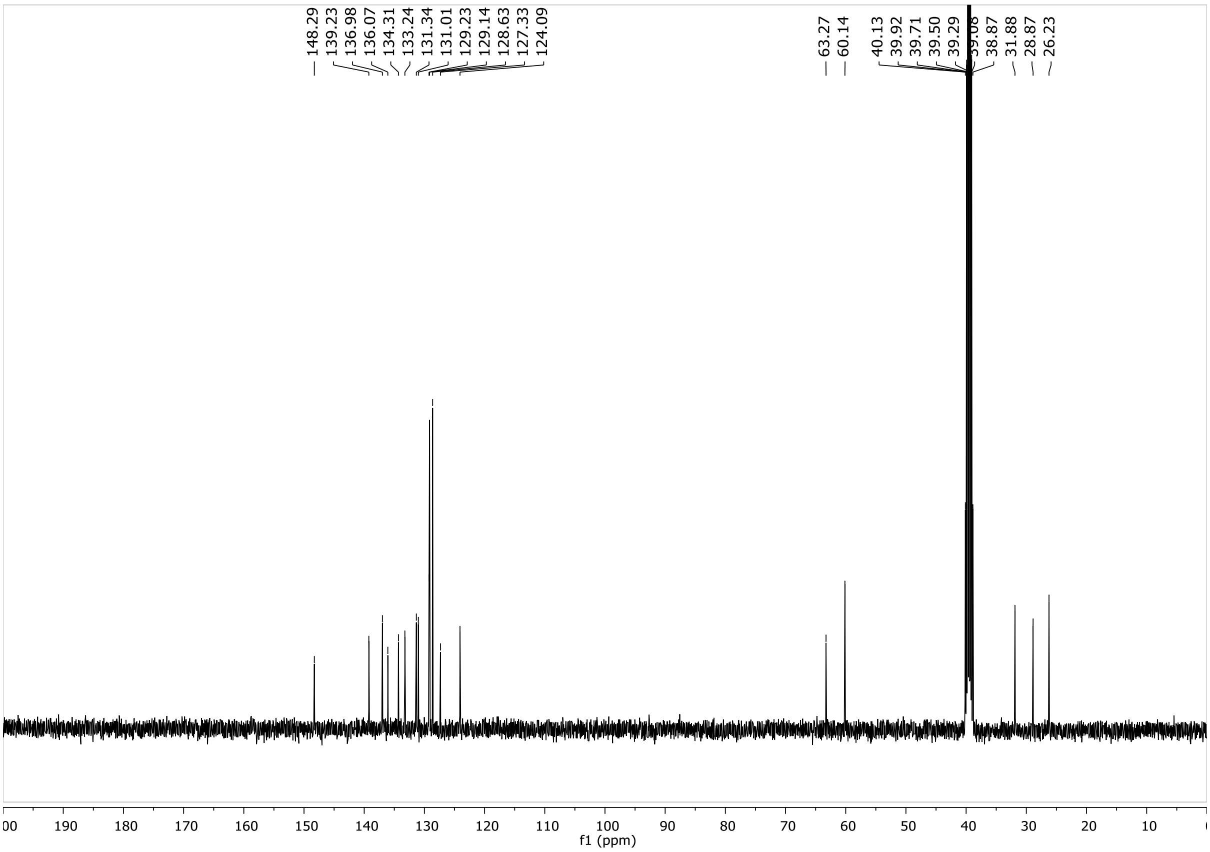
148.29
139.23
136.98
136.07
134.31
133.24
131.34
131.01
129.23
129.14
128.63
127.33
124.09
63.27
60.14
40.13
39.92
39.71
39.50
39.29
38.87
31.88
28.87
26.23
f1 (ppm)

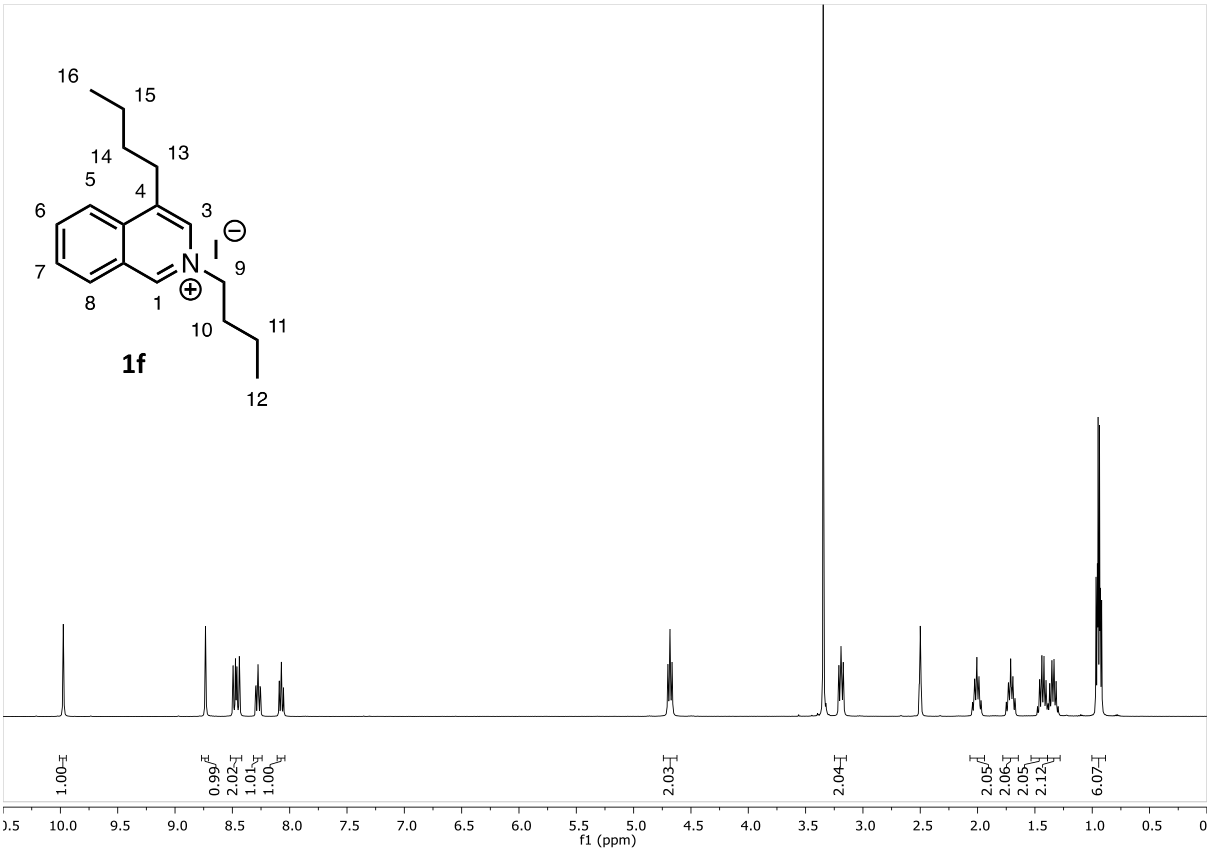

16
15
14
13
5
4
3
6
7
8
1
9
10
11
12
1f
1.00
0.99
2.02
1.01
1.00
2.03
2.04
2.05
2.06
2.05
2.12
6.07
f1 (ppm)

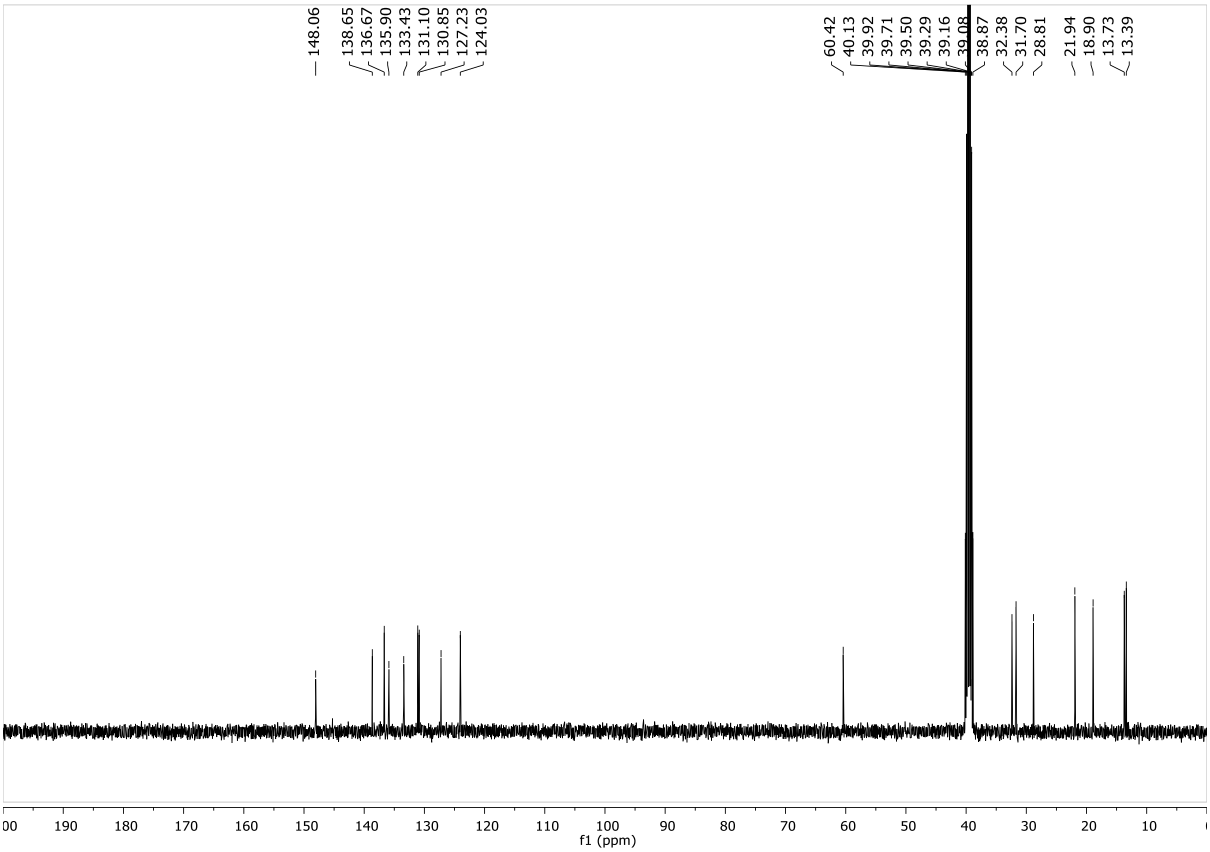

148.06
138.65
136.67
135.90
133.43
131.10
130.85
127.23
124.03
60.42
40.13
39.92
39.71
39.50
39.29
39.16
39.08
38.87
32.38
31.70
28.81
21.94
18.90
13.73
13.39
f1 (ppm)

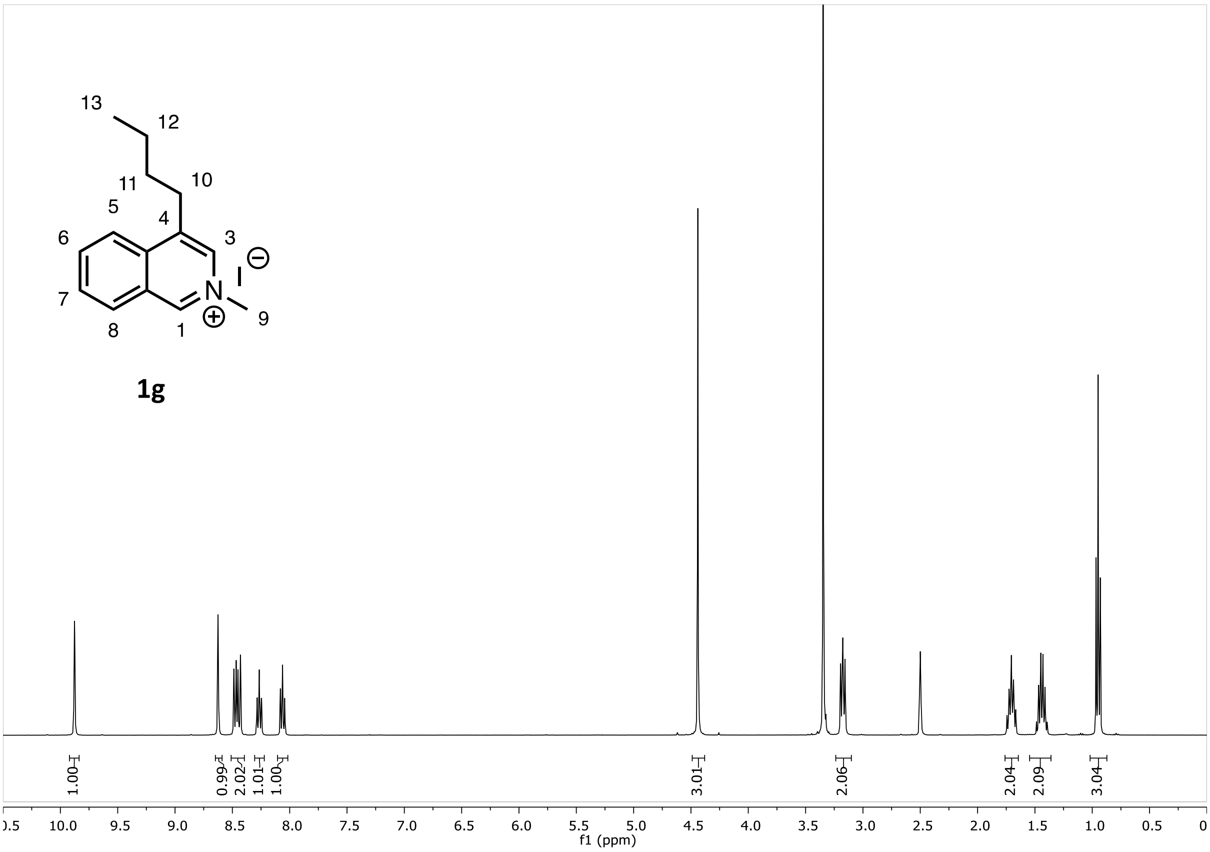

13
12
11
10
5
4
6
3
7
N
I
9
8
1
1g
1.00
0.99
2.02
1.01
1.00
3.01
2.06
2.04
2.09
3.04
f1 (ppm)

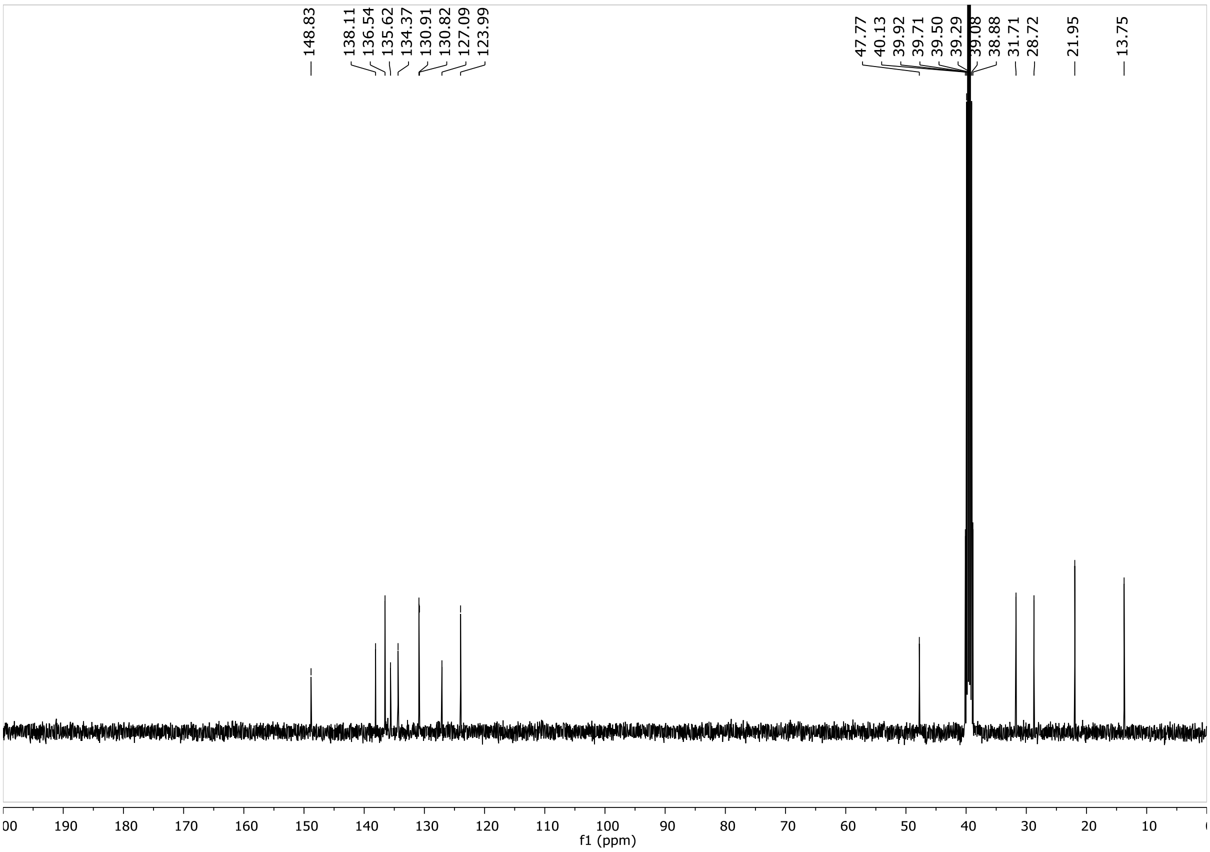

148.83
138.11
136.54
135.62
134.37
130.91
130.82
127.09
123.99
47.77
40.13
39.92
39.71
39.50
39.29
39.08
38.88
31.71
28.72
21.95
13.75
f1 (ppm)

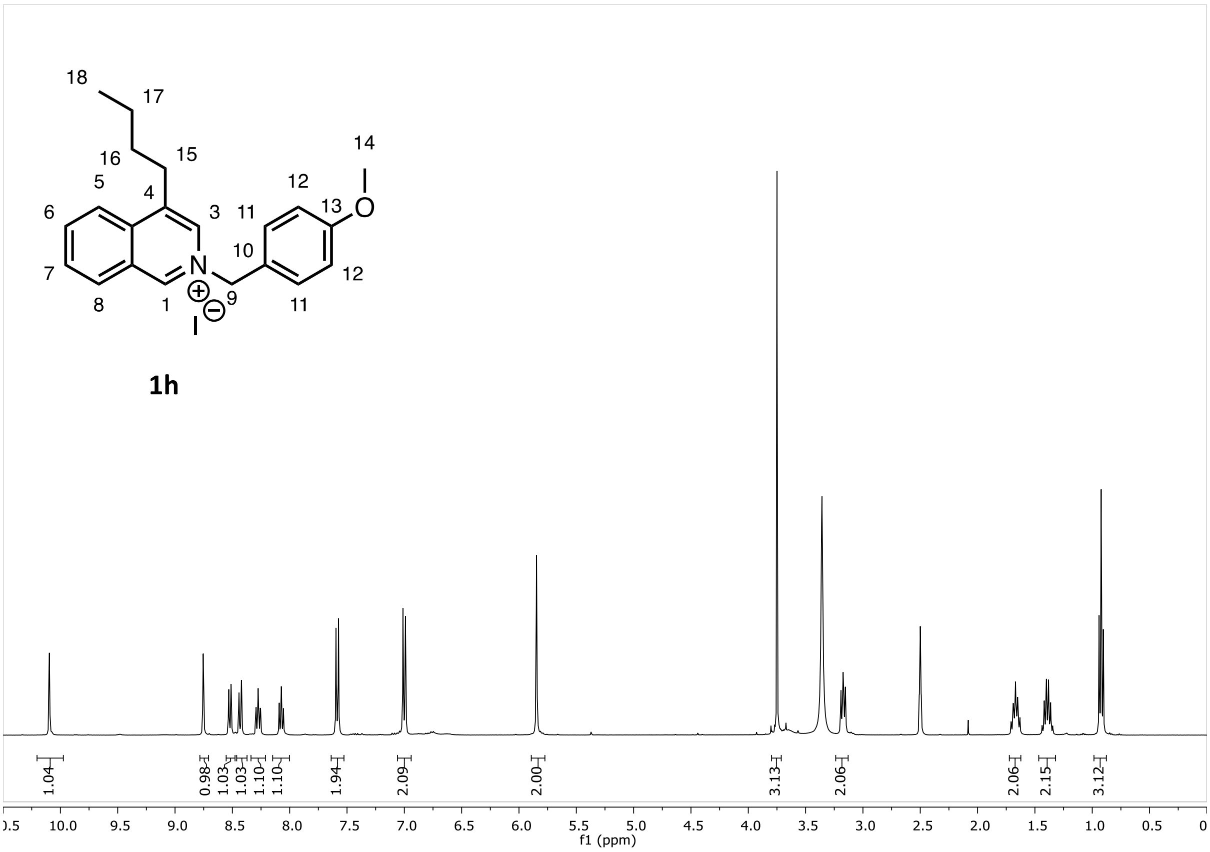

18
17
16
15
14
5
4
12
6
3
11
13
O
10
N
7
12
9
8
1
11
I
1h
1.04
0.98
1.03
1.03
1.10
1.10
1.94
2.09
2.00
3.13
2.06
2.06
2.15
3.12
f1 (ppm)

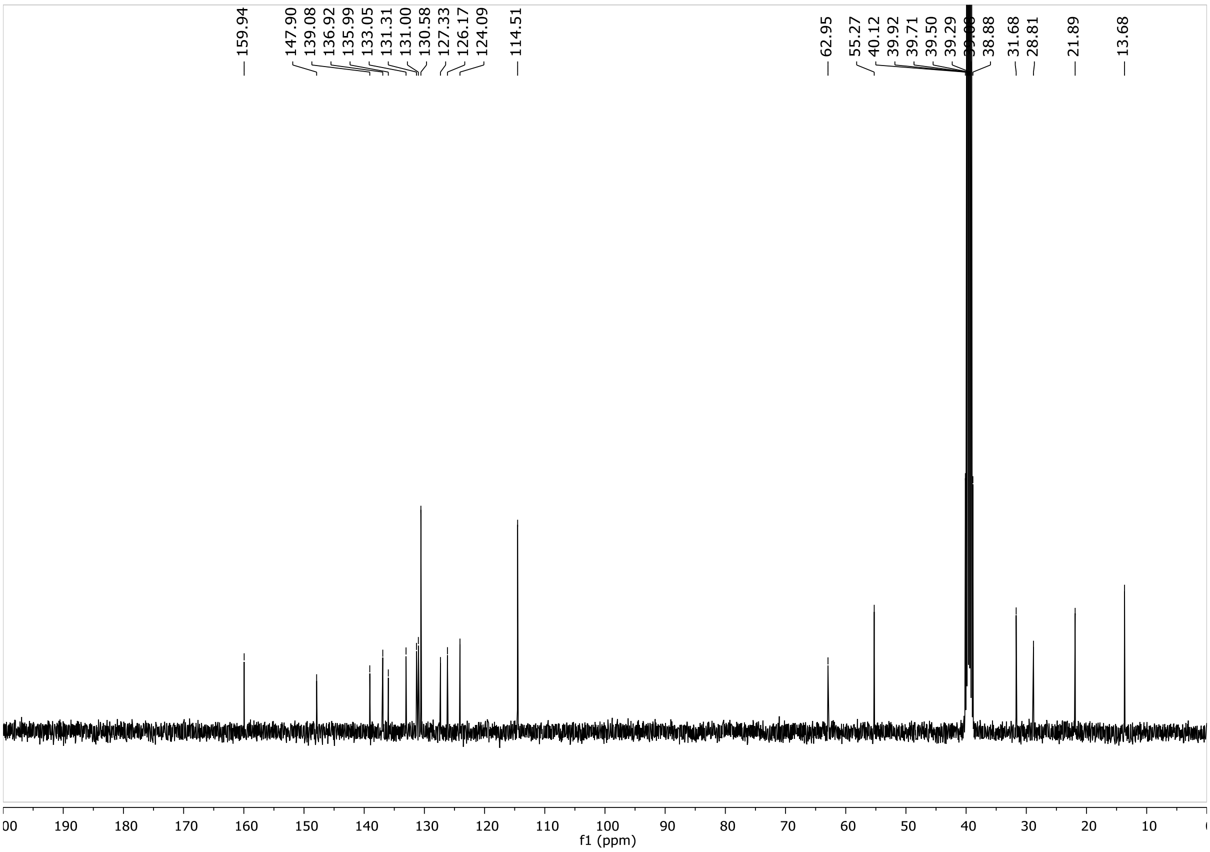

159.94
147.90
139.08
136.92
135.99
133.05
131.31
131.00
130.58
127.33
126.17
124.09
114.51
62.95
55.27
40.12
39.92
39.71
39.50
39.29
38.88
31.68
28.81
21.89
13.68
f1 (ppm)

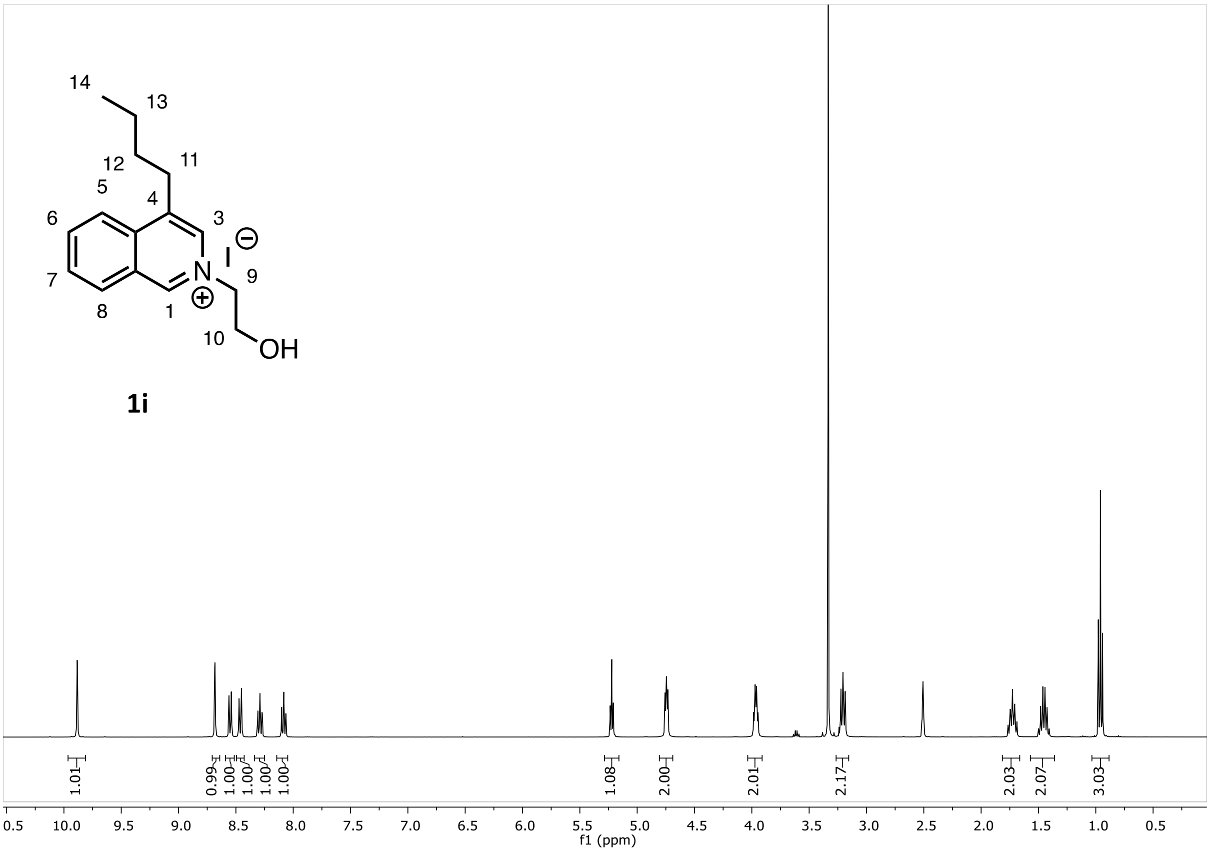
14
13
12
11
5
4
6
3
7
9
8
1
10
N
I
OH
1i
1.01
0.99
1.00
1.00
1.00
1.00
1.08
2.00
2.01
2.17
2.03
2.07
3.03
f1 (ppm)

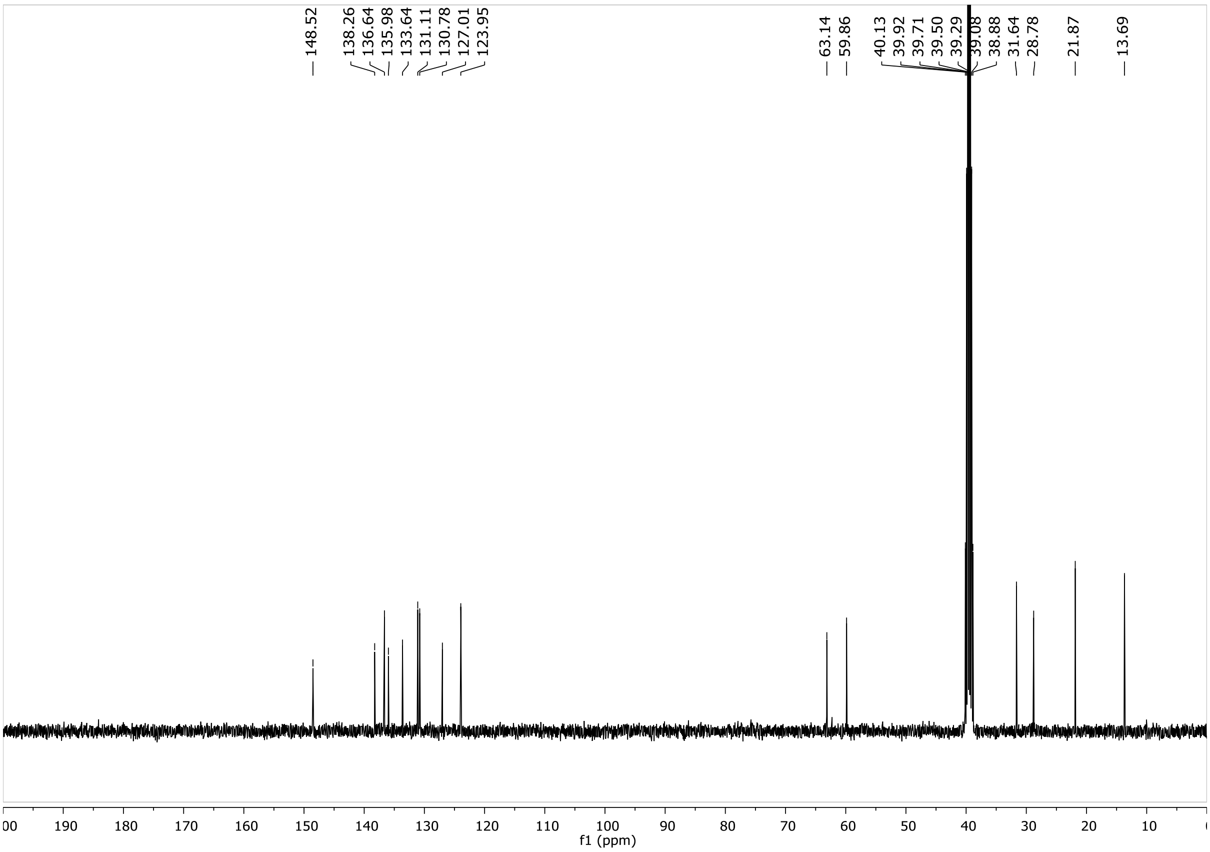
148.52
138.26
136.64
135.98
133.64
131.11
130.78
127.01
123.95
63.14
59.86
40.13
39.92
39.71
39.50
39.29
39.08
38.88
31.64
28.78
21.87
13.69
f1 (ppm)

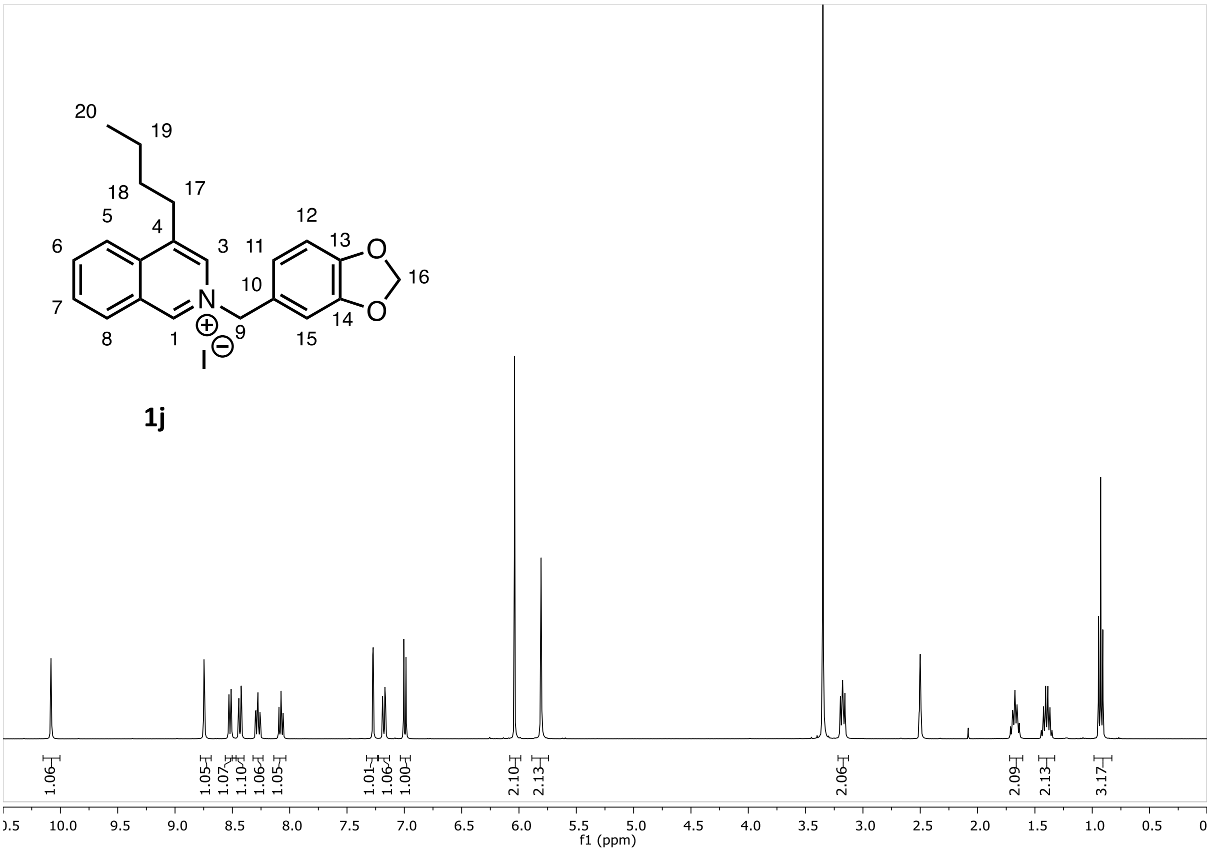
1j
f1 (ppm)

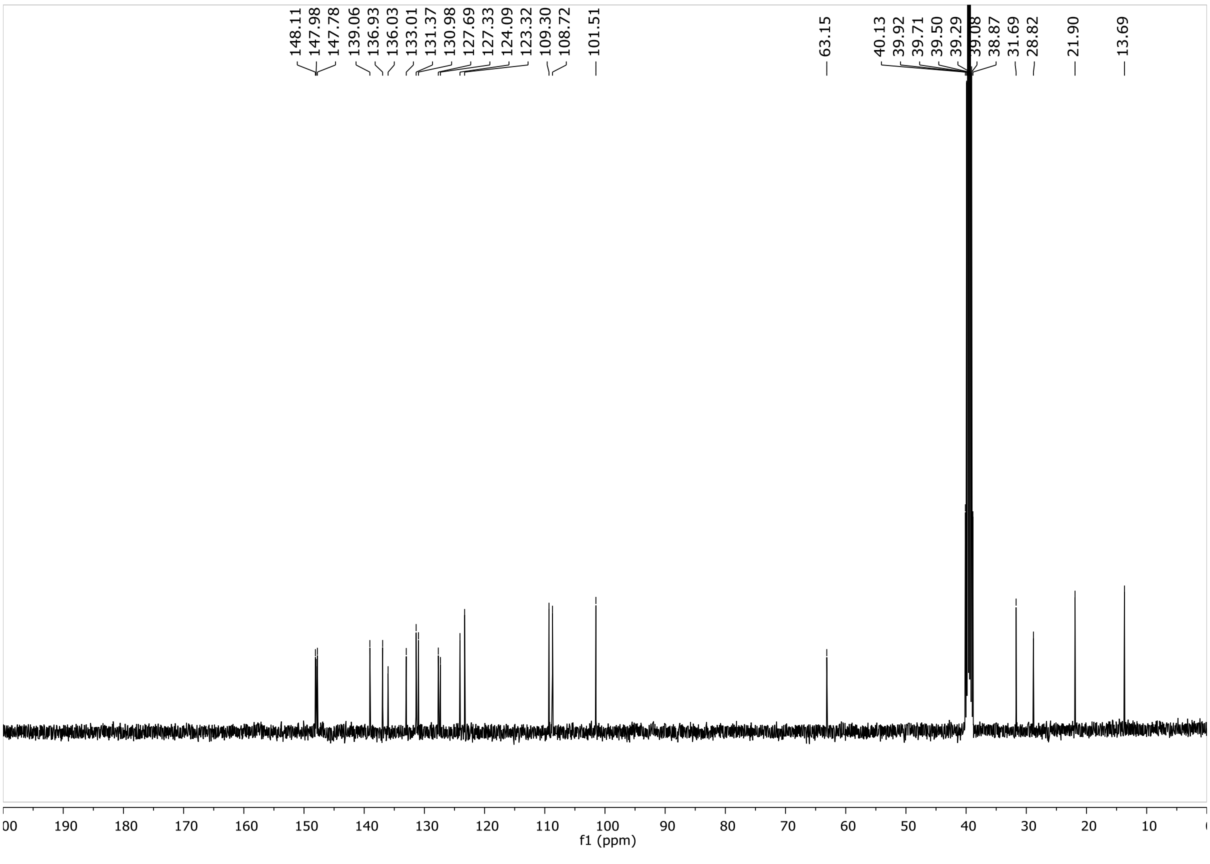
148.11
147.98
147.78
139.06
136.93
136.03
133.01
131.37
130.98
127.69
127.33
124.09
123.32
109.30
108.72
101.51
63.15
40.13
39.92
39.71
39.50
39.29
39.08
38.87
31.69
28.82
21.90
13.69
f1 (ppm)

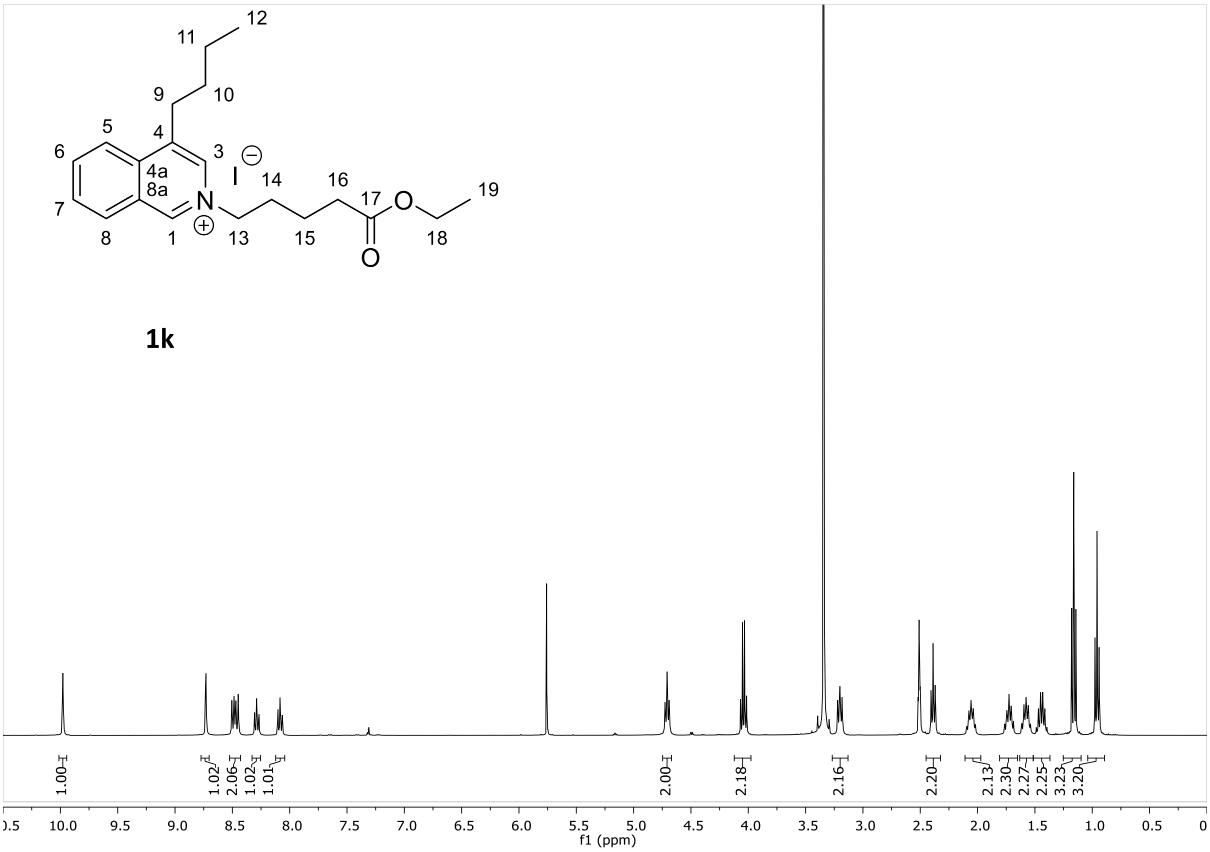

12
11
10
9
5
4
6
3
4a
8a
I
14
16
17
19
7
N
O
8
1
13
15
18
O
1k
1.00
1.02
2.06
1.02
1.01
2.00
2.18
2.16
2.20
2.13
2.30
2.27
2.25
3.23
3.20
f1 (ppm)

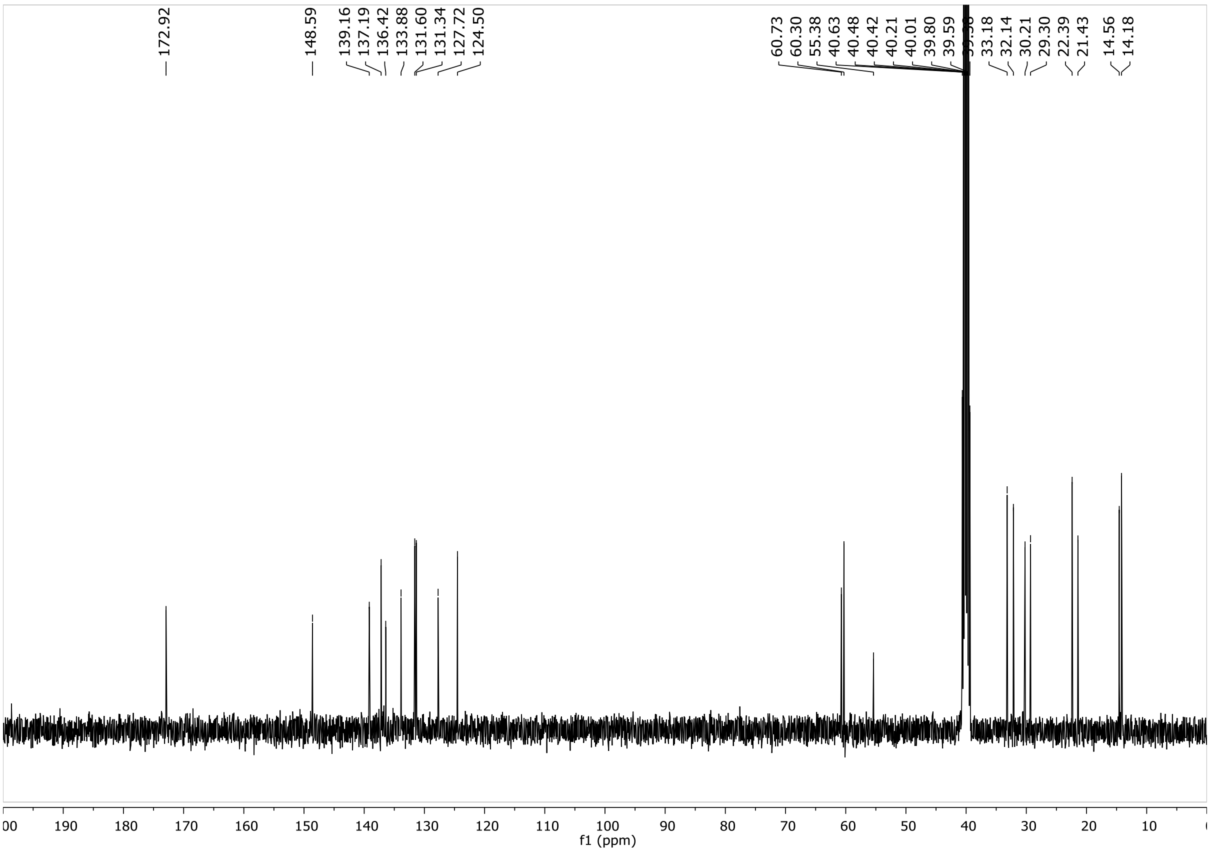

172.92
148.59
139.16
137.19
136.42
133.88
131.60
131.34
127.72
124.50
60.73
60.30
55.38
40.63
40.48
40.42
40.21
40.01
39.80
39.59
33.18
32.14
30.21
29.30
22.39
21.43
14.56
14.18
f1 (ppm)

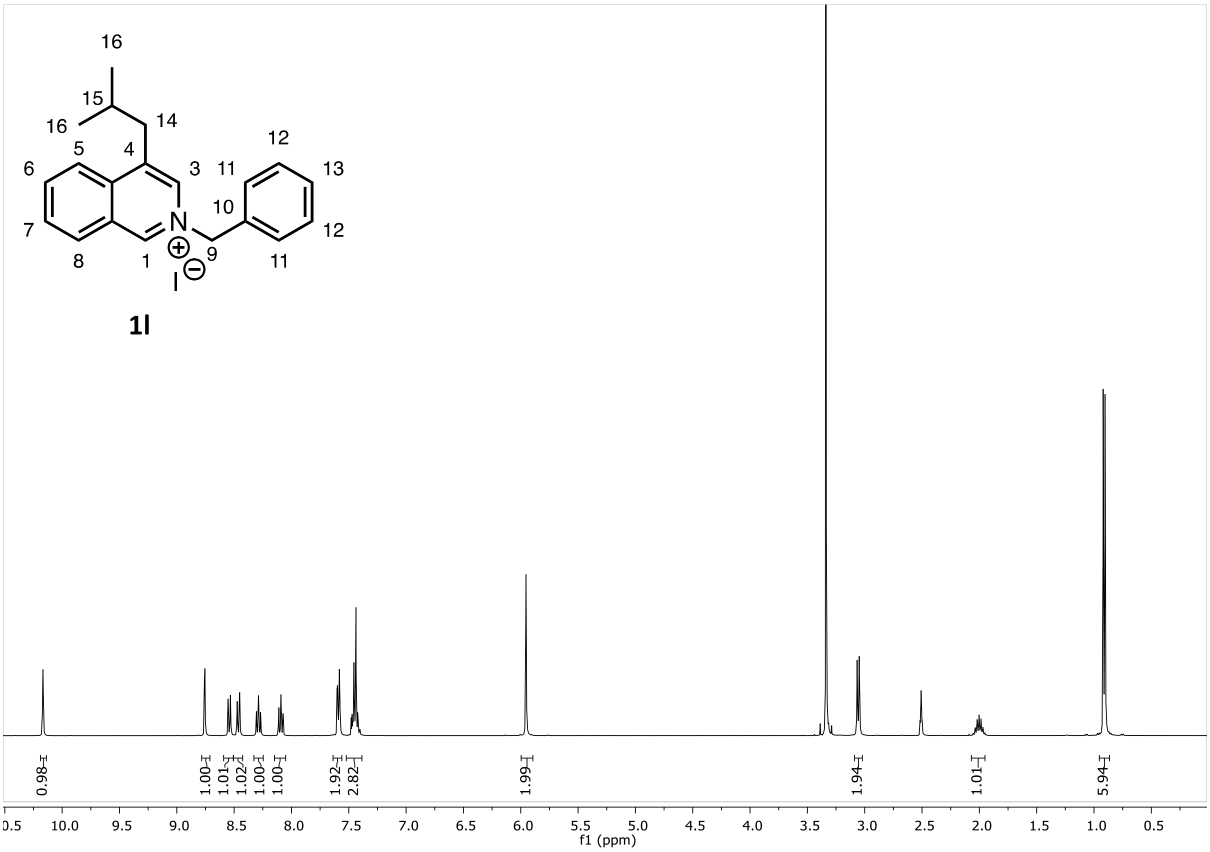
16
15
16
14
5
4
6
3
11
12
13
7
10
N
9
12
8
1
11
I
1l
0.98
1.00
1.01
1.02
1.00
1.00
1.92
2.82
1.99
1.94
1.01
5.94
f1 (ppm)

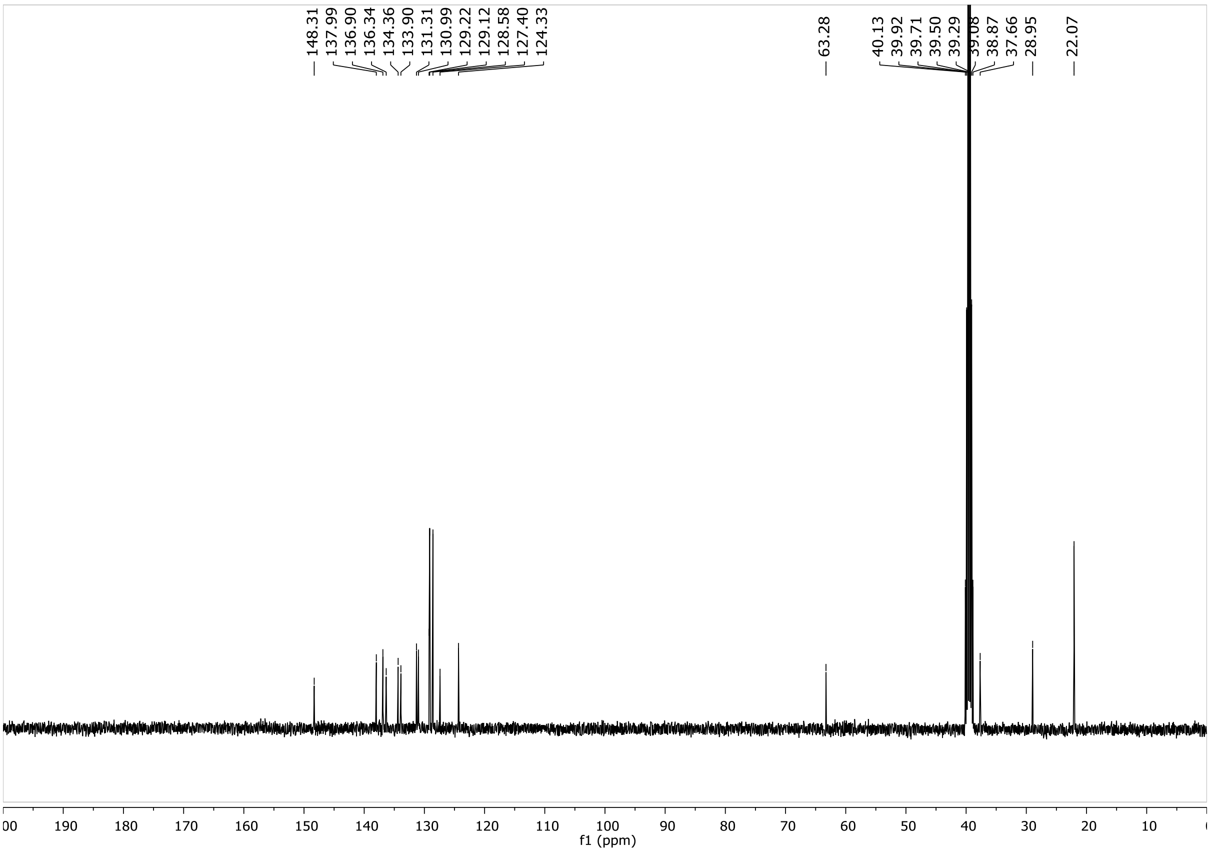
148.31
137.99
136.90
136.34
134.36
133.90
131.31
130.99
129.22
129.12
128.58
127.40
124.33
63.28
40.13
39.92
39.71
39.50
39.29
39.08
38.87
37.66
28.95
22.07
f1 (ppm)

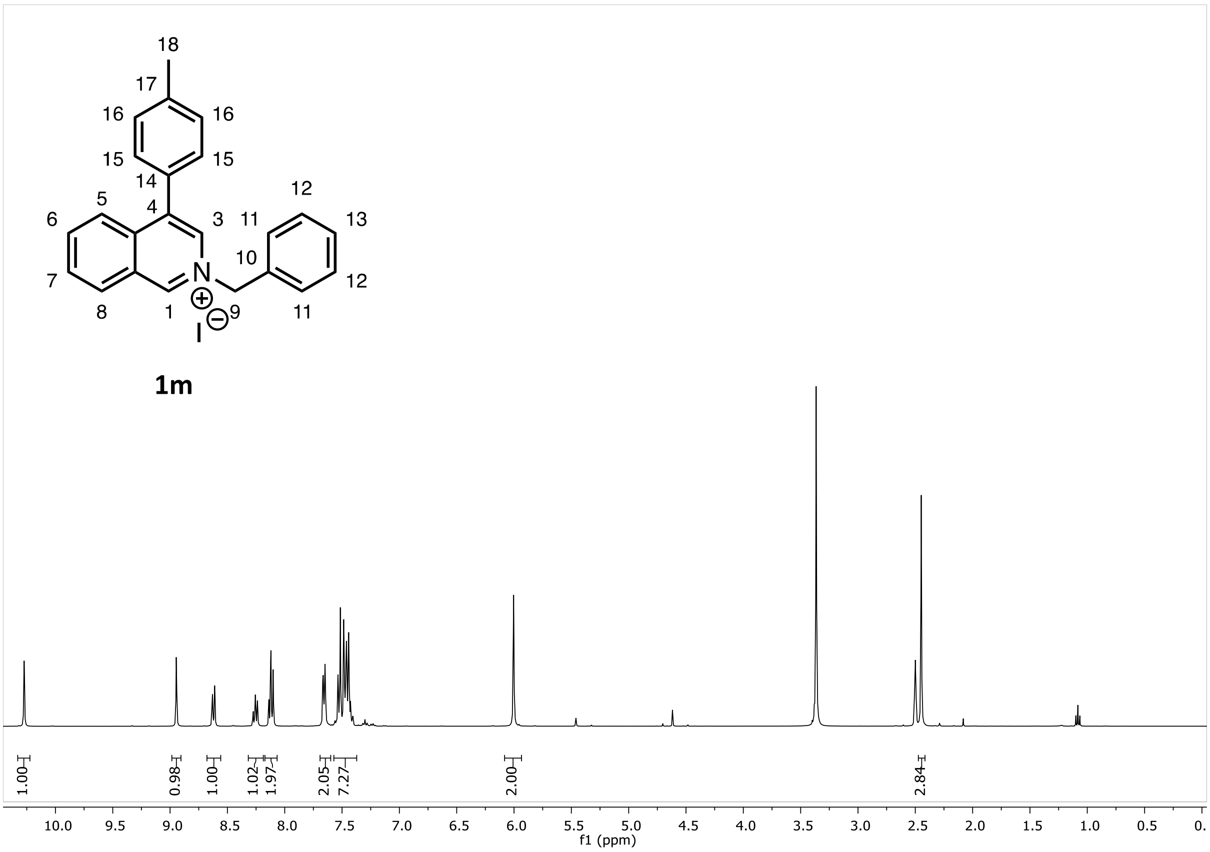
18
17
16
16
15
15
14
5
4
3
11
12
13
6
10
7
12
8
1
9
11
N
I
1m
1.00
0.98
1.00
1.02
1.97
2.05
7.27
2.00
2.84
10.0 9.5 9.0 8.5 8.0 7.5 7.0 6.5 6.0 5.5 5.0 4.5 4.0 3.5 3.0 2.5 2.0 1.5 1.0 0.5
f1 (ppm)

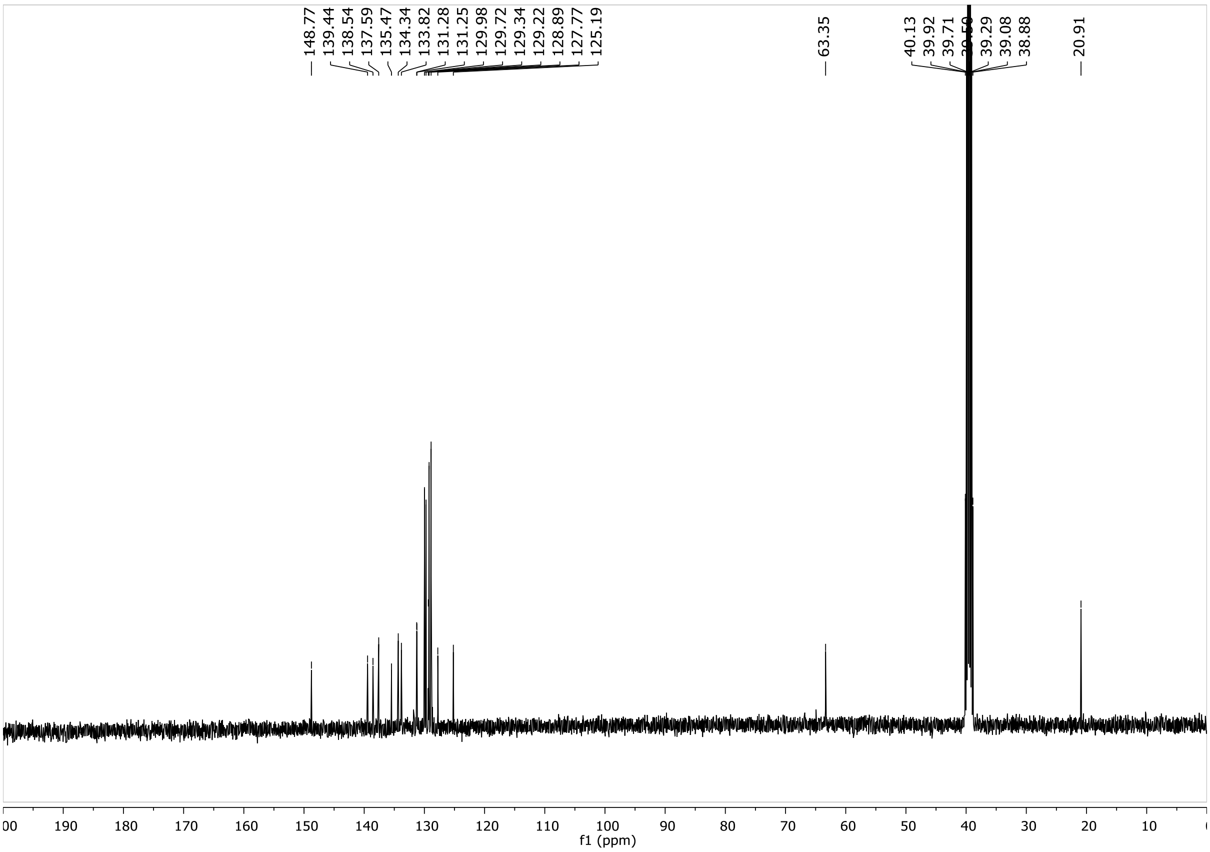
148.77
139.44
138.54
137.59
135.47
134.34
133.82
131.28
131.25
129.98
129.72
129.34
129.22
128.89
127.77
125.19
63.35
40.13
39.92
39.71
39.29
39.08
38.88
20.91
190 180 170 160 150 140 130 120 110 100 90 80 70 60 50 40 30 20 10
f1 (ppm)

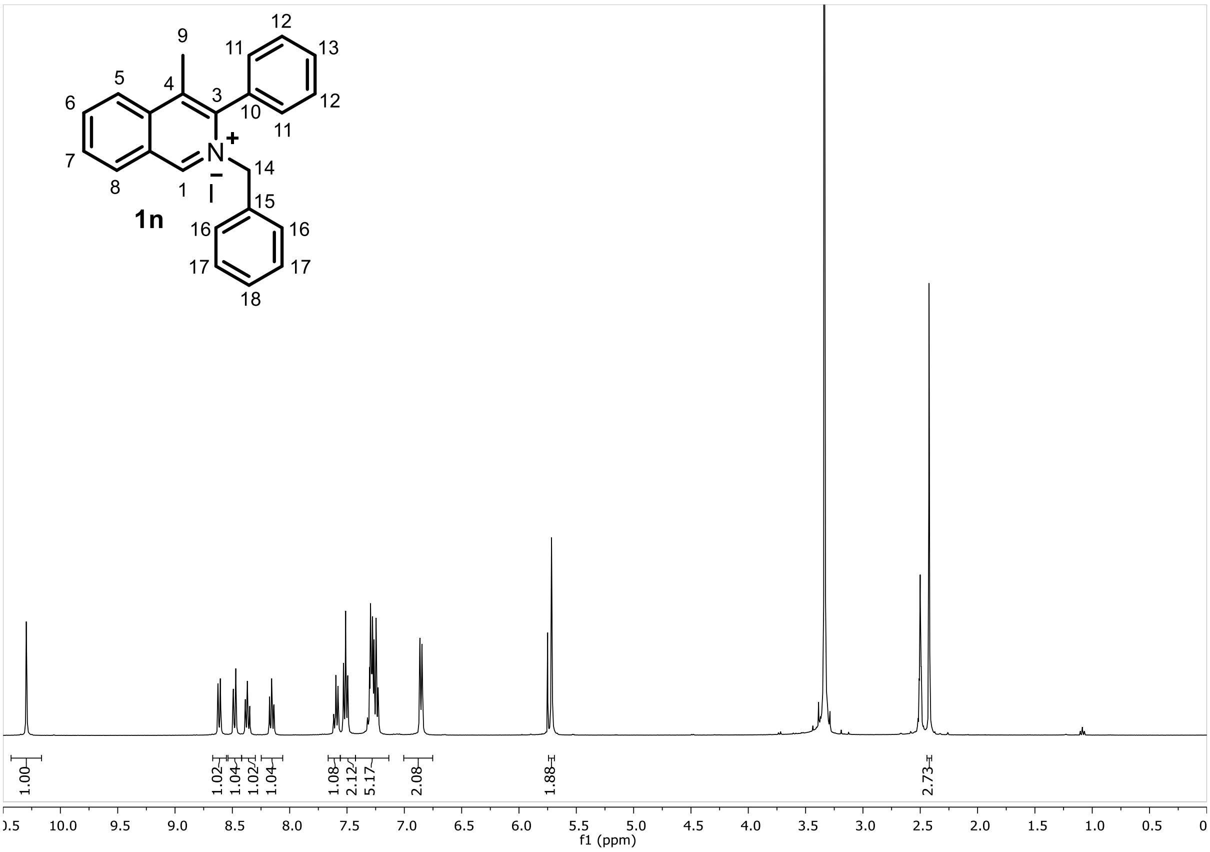
1n
1.00
1.02
1.04
1.02
1.04
1.08
2.12
5.17
2.08
1.88
2.73
f1 (ppm)

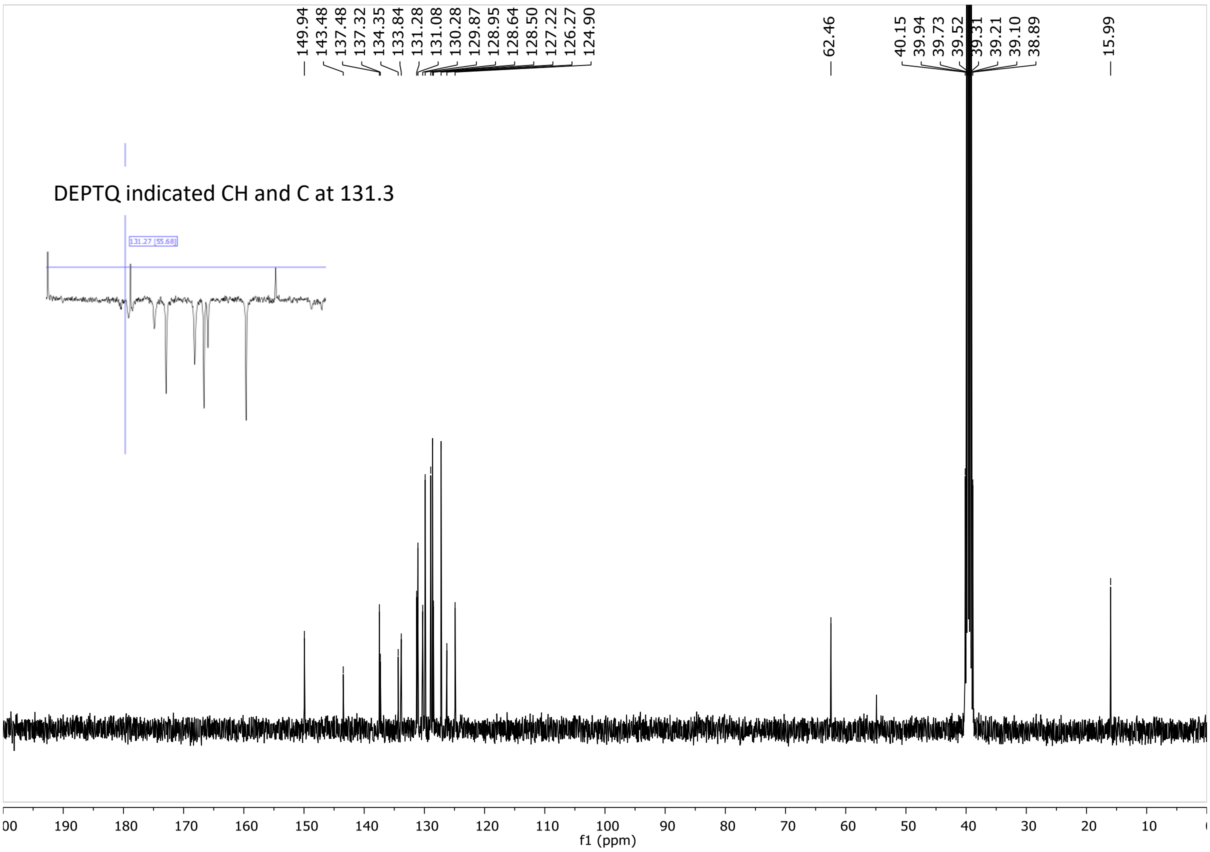
149.94
143.48
137.48
137.32
134.35
133.84
131.28
131.08
130.28
129.87
128.95
128.64
128.50
127.22
126.27
124.90
62.46
40.15
39.94
39.73
39.52
39.31
39.21
39.10
38.89
15.99
DEPTQ indicated CH and C at 131.3
f1 (ppm)

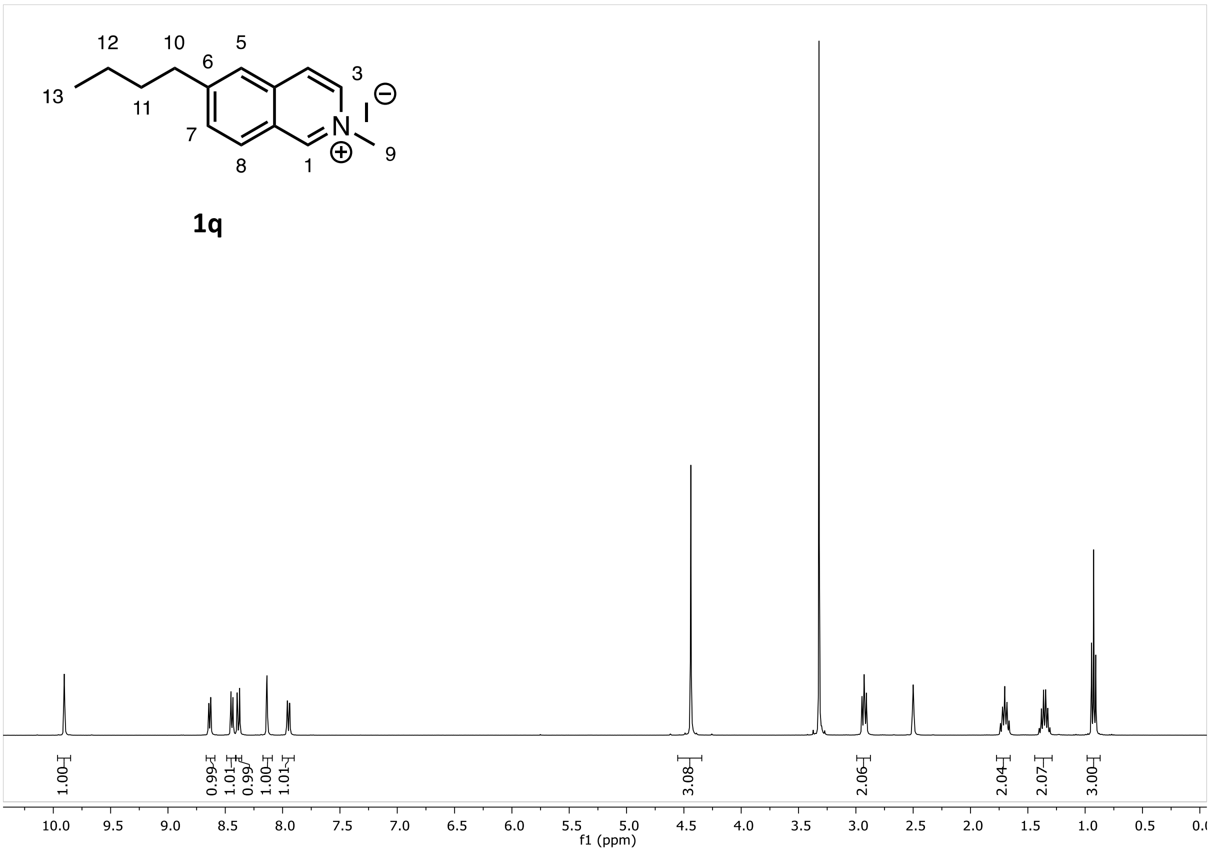

1q
1.00
0.99
1.01
0.99
1.00
1.01
3.08
2.06
2.04
2.07
3.00
f1 (ppm)

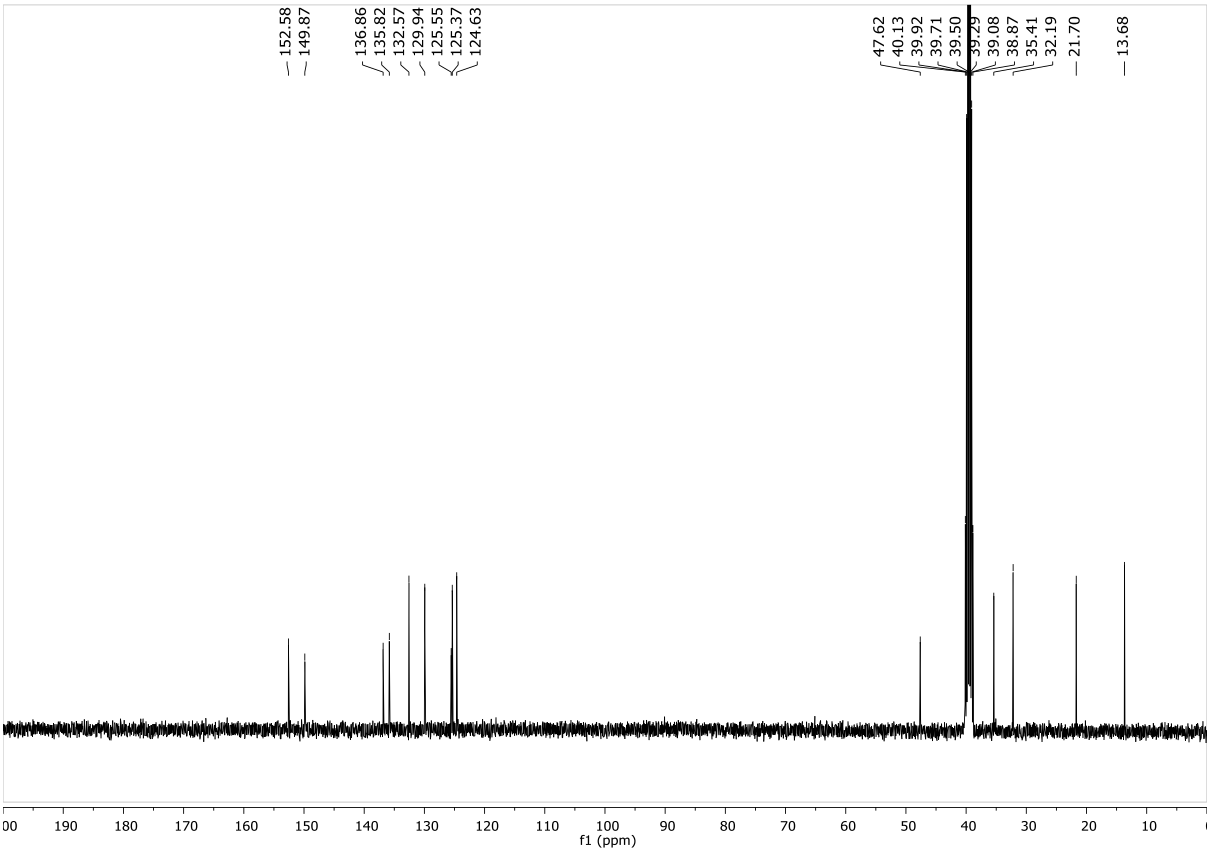

152.58
149.87
136.86
135.82
132.57
129.94
125.55
125.37
124.63
47.62
40.13
39.92
39.71
39.50
39.29
39.08
38.87
35.41
32.19
21.70
13.68
f1 (ppm)

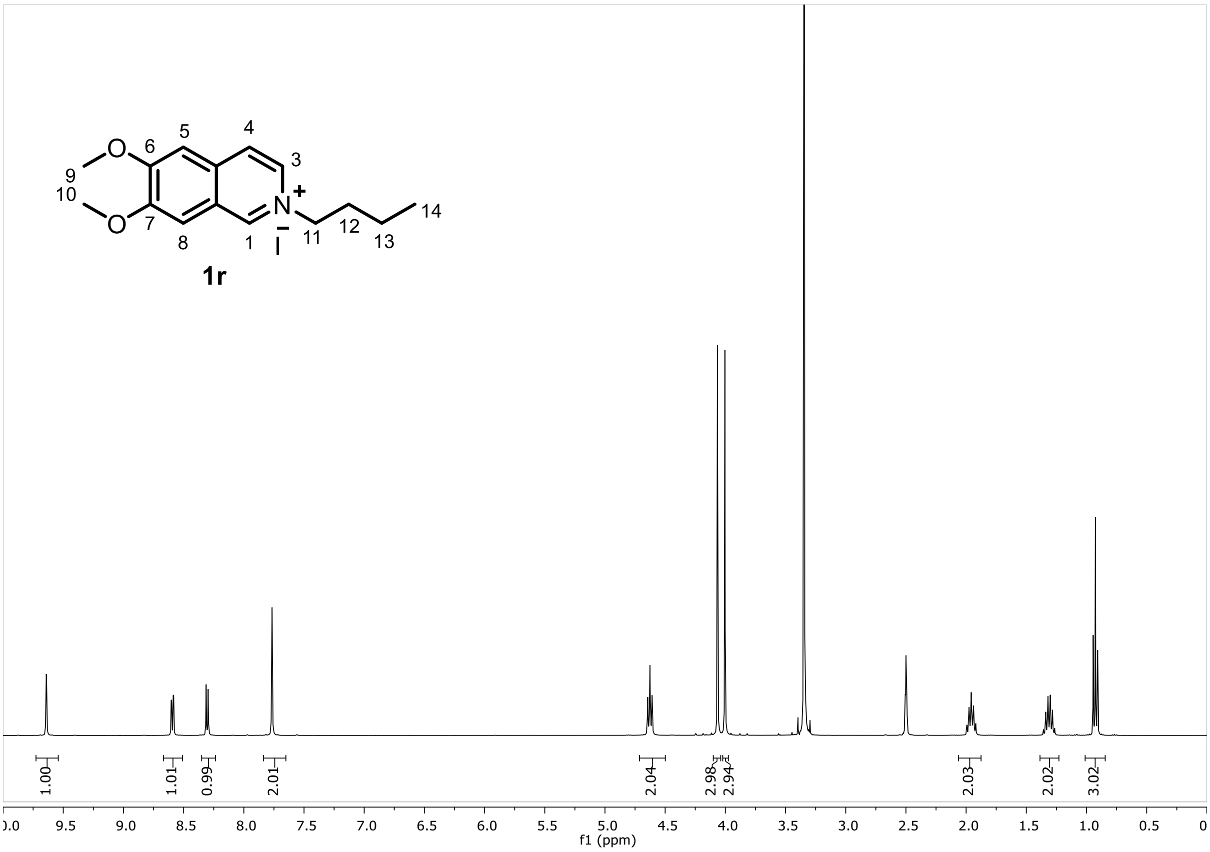

O
O
N
+
I
−
1
3
4
5
6
7
8
9
10
11
12
13
14
1r
1.00
1.01
0.99
2.01
2.04
2.98
2.94
2.03
2.02
3.02
9.5
9.0
8.5
8.0
7.5
7.0
6.5
6.0
5.5
5.0
4.5
4.0
3.5
3.0
2.5
2.0
1.5
1.0
0.5
f1 (ppm)

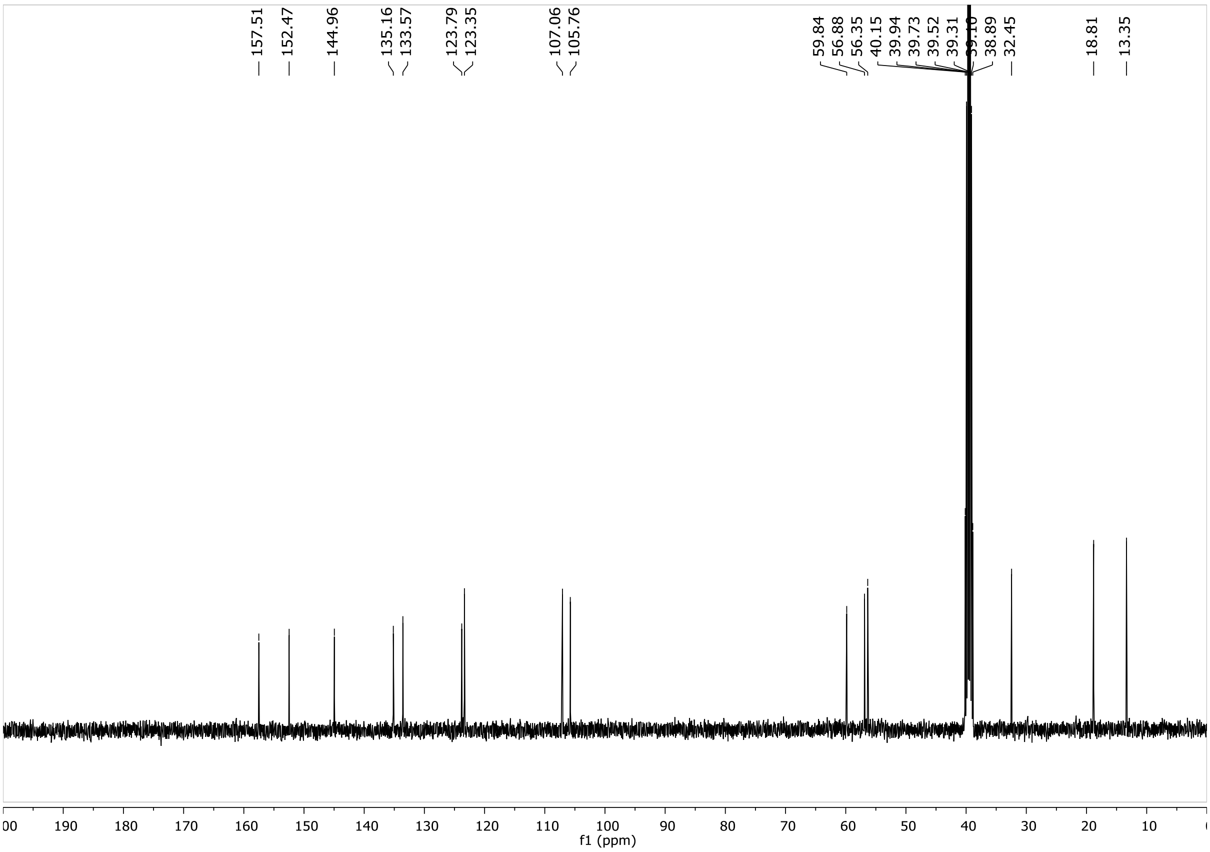

157.51
152.47
144.96
135.16
133.57
123.79
123.35
107.06
105.76
59.84
56.88
56.35
40.15
39.94
39.73
39.52
39.31
38.89
32.45
18.81
13.35
190
180
170
160
150
140
130
120
110
100
90
80
70
60
50
40
30
20
10
f1 (ppm)

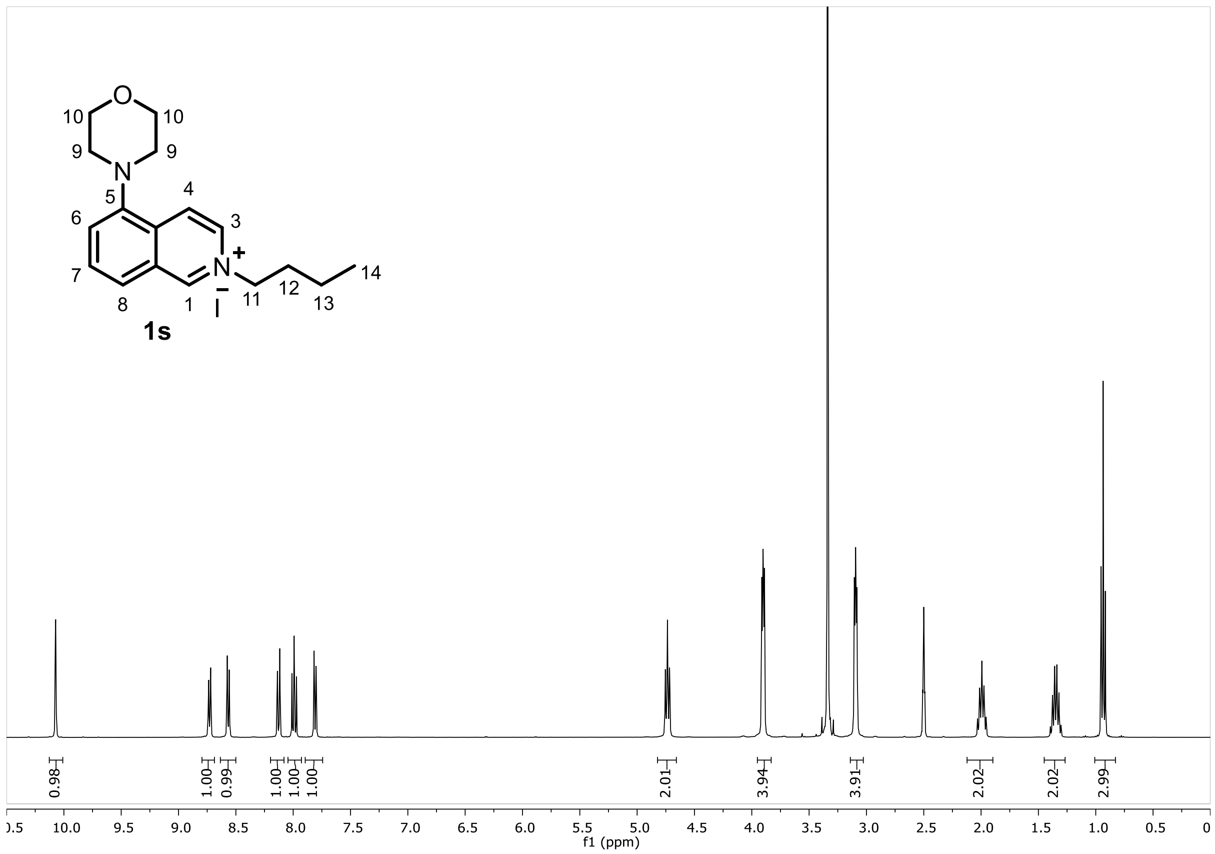
1s
0.98
1.00
0.99
1.00
1.00
1.00
2.01
3.94
3.91
2.02
2.02
2.99
f1 (ppm)

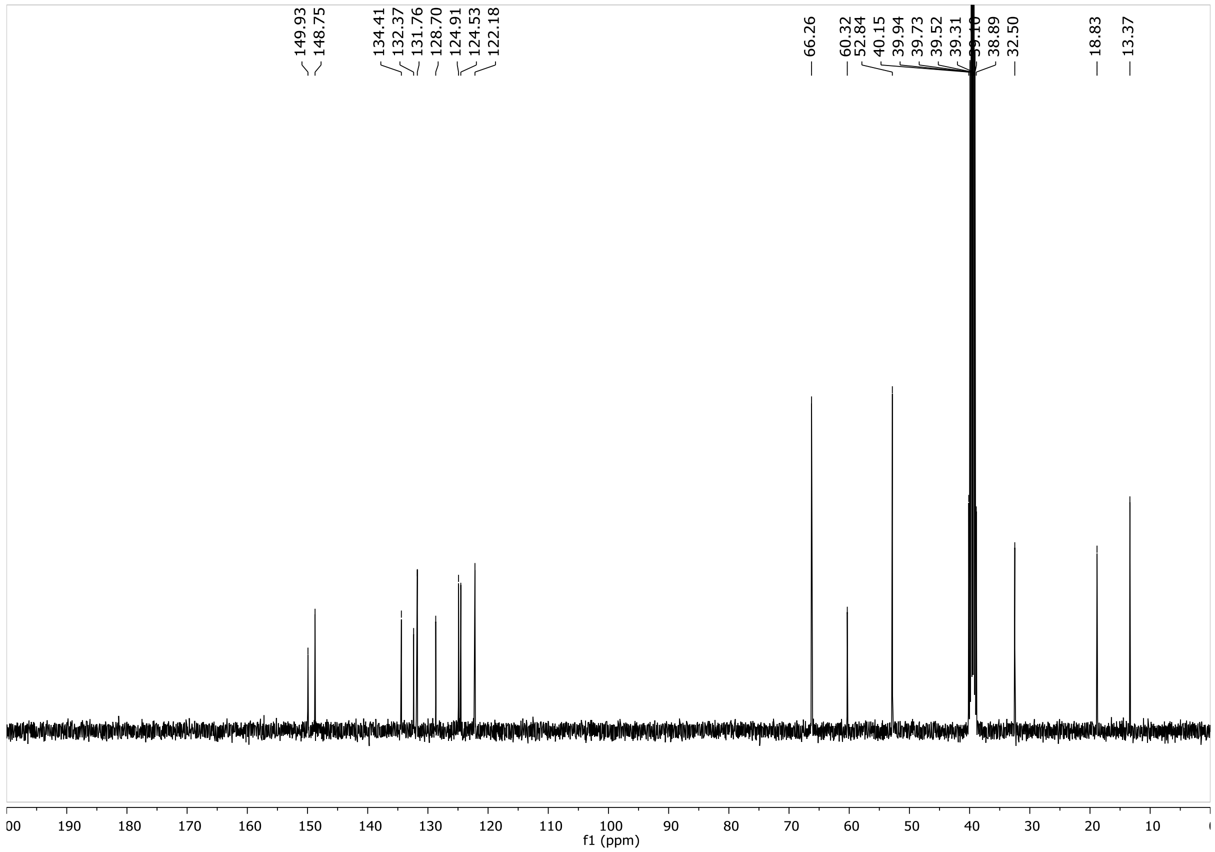
149.93
148.75
134.41
132.37
131.76
128.70
124.91
124.53
122.18
66.26
60.32
52.84
40.15
39.94
39.73
39.52
39.31
38.89
32.50
18.83
13.37
f1 (ppm)

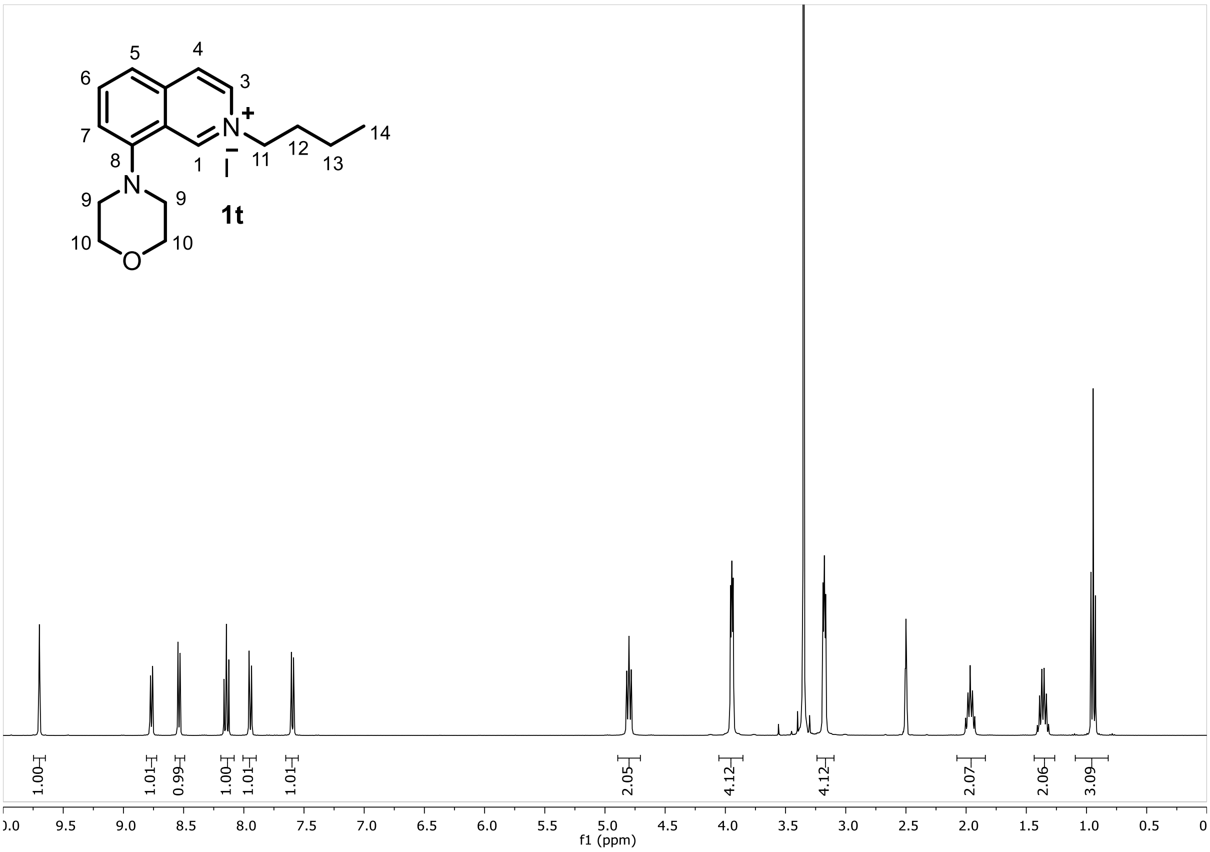
1t
1.00
1.01
0.99
1.00
1.01
1.01
2.05
4.12
4.12
2.07
2.06
3.09
f1 (ppm)

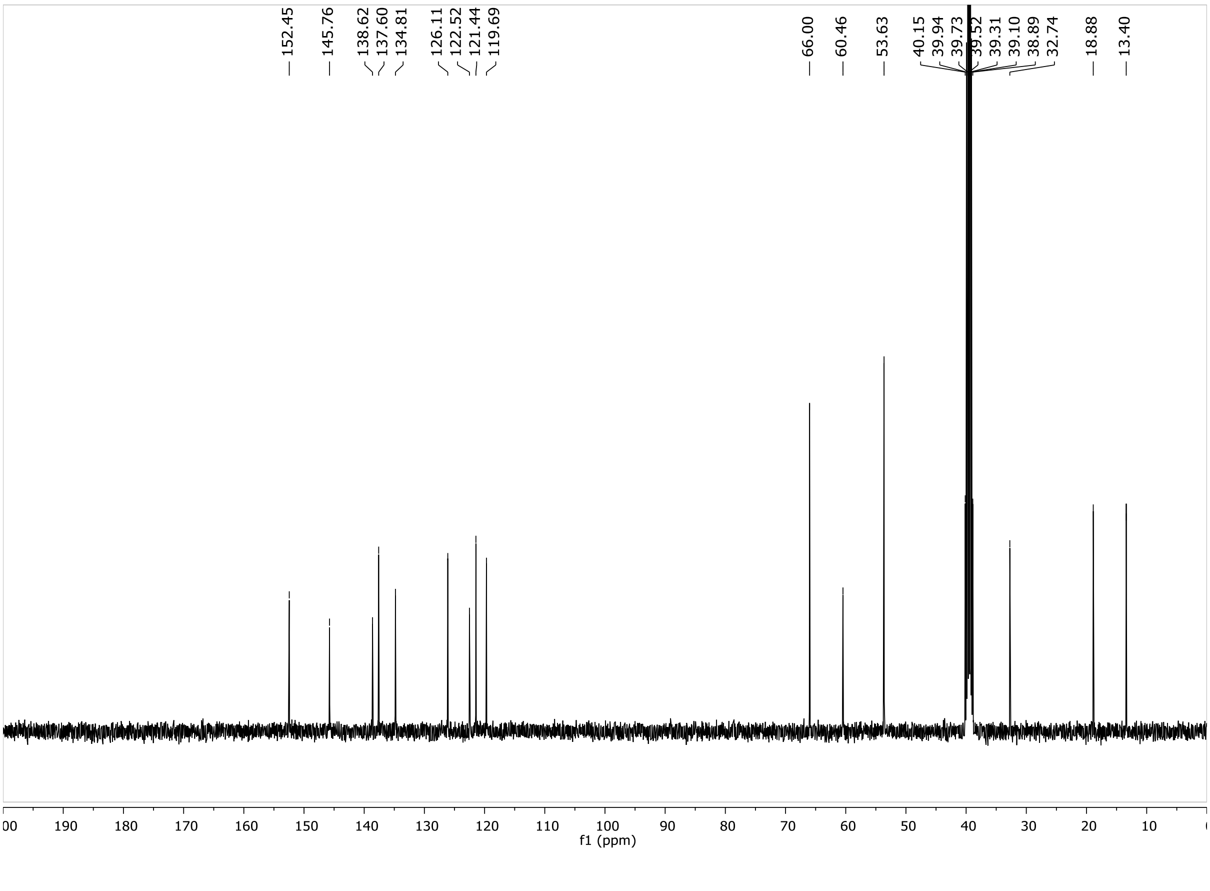
152.45
145.76
138.62
137.60
134.81
126.11
122.52
121.44
119.69
66.00
60.46
53.63
40.15
39.94
39.73
39.52
39.31
39.10
38.89
32.74
18.88
13.40
f1 (ppm)

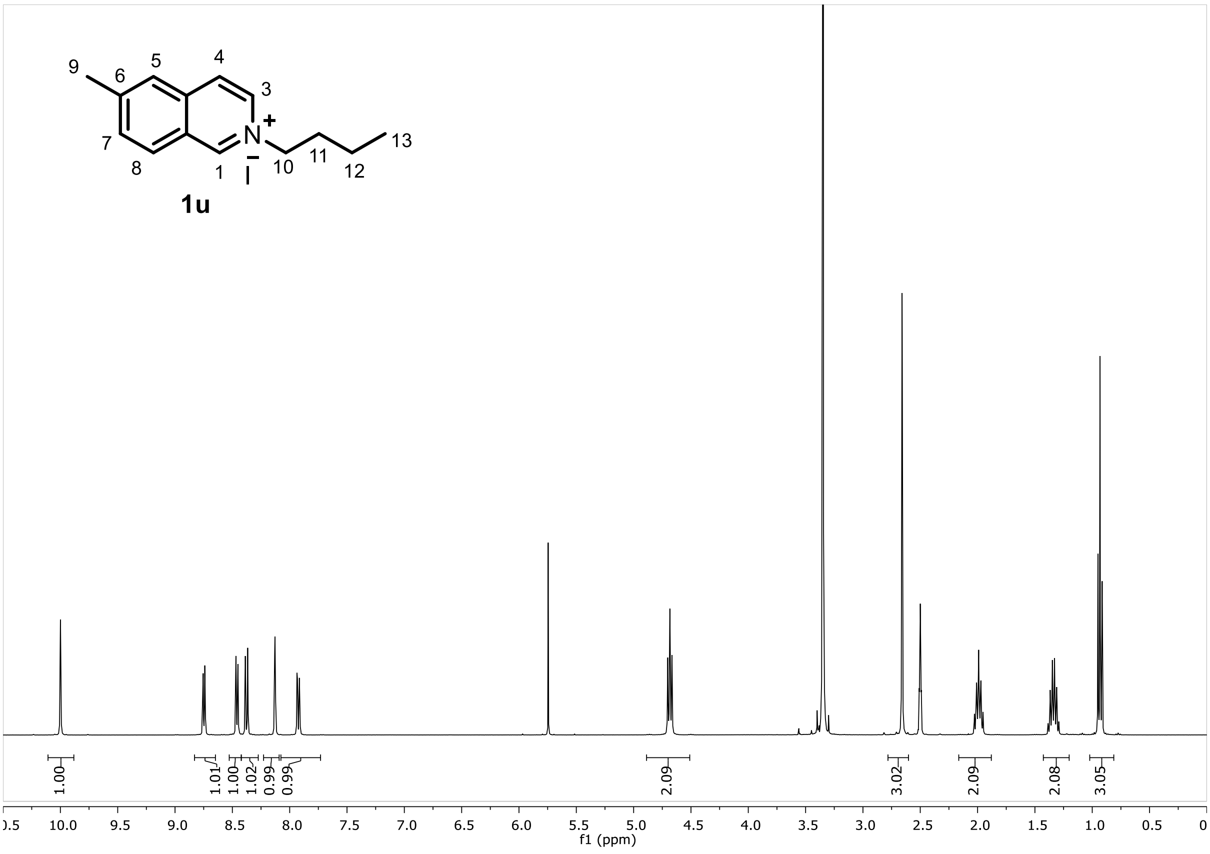
1u
1.00
1.01
1.00
1.02
0.99
0.99
2.09
3.02
2.09
2.08
3.05
f1 (ppm)

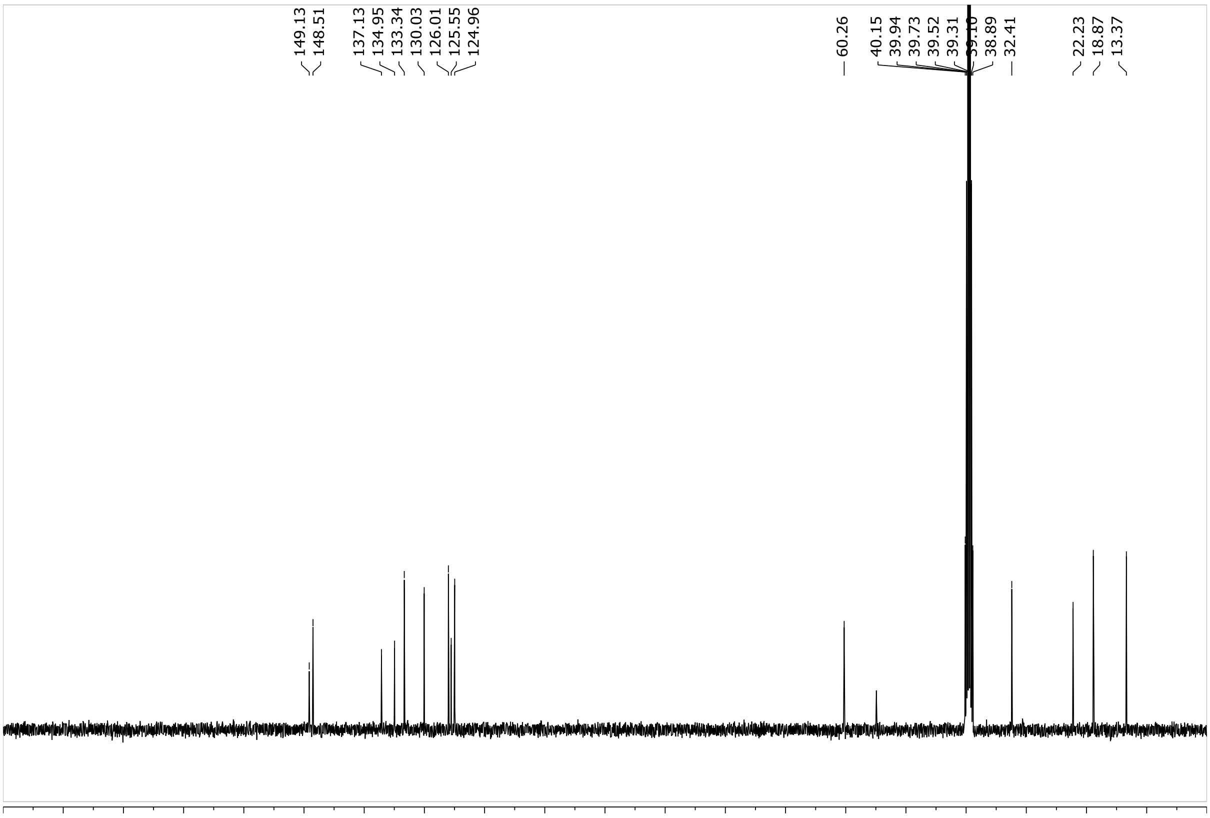
149.13
148.51
137.13
134.95
133.34
130.03
126.01
125.55
124.96
60.26
40.15
39.94
39.73
39.52
39.31
39.10
38.89
32.41
22.23
18.87
13.37
f1 (ppm)

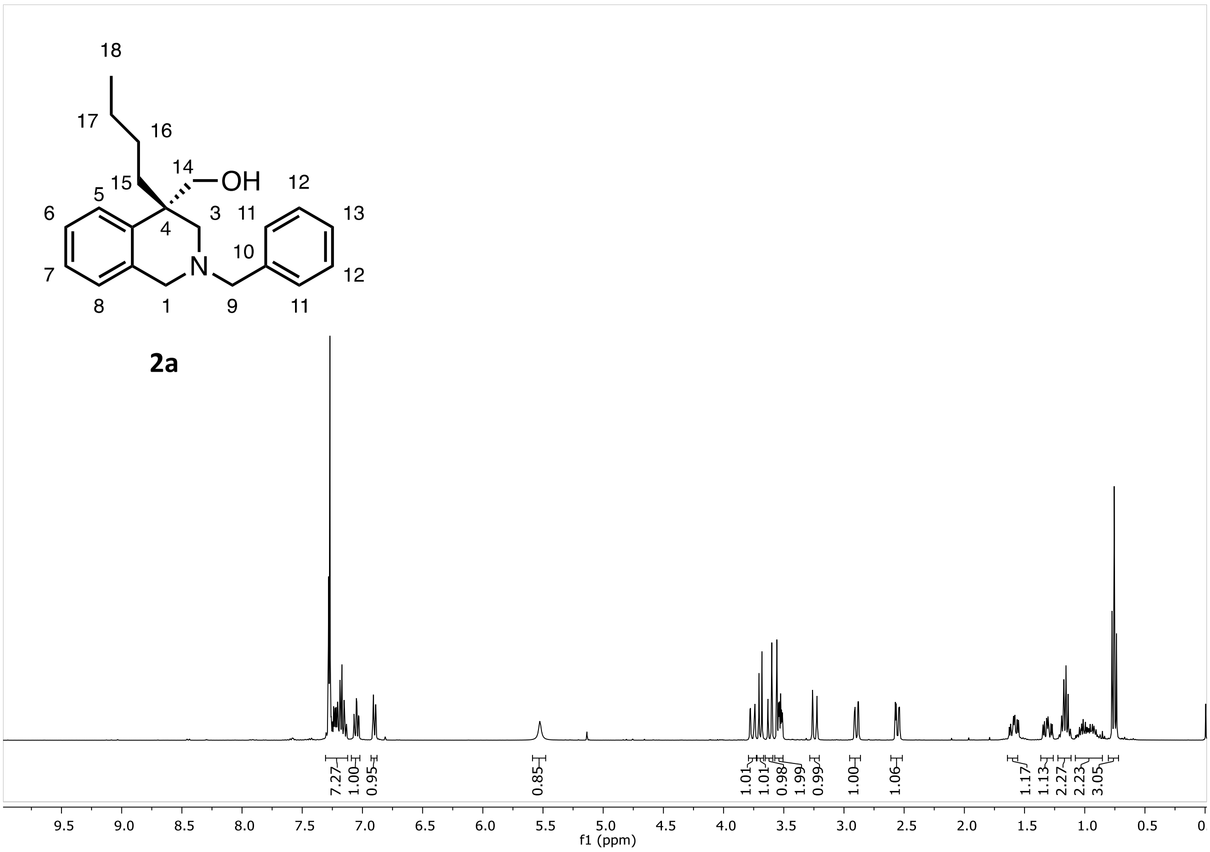
18
17
16
14
OH
15
5
6
4
3
11
12
13
10
7
N
12
8
1
9
11
2a
7.27
1.00
0.95
0.85
1.01
1.01
0.98
1.99
0.99
1.00
1.06
1.17
1.13
2.27
2.23
3.05
9.5 9.0 8.5 8.0 7.5 7.0 6.5 6.0 5.5 5.0 4.5 4.0 3.5 3.0 2.5 2.0 1.5 1.0 0.5 0
f1 (ppm)

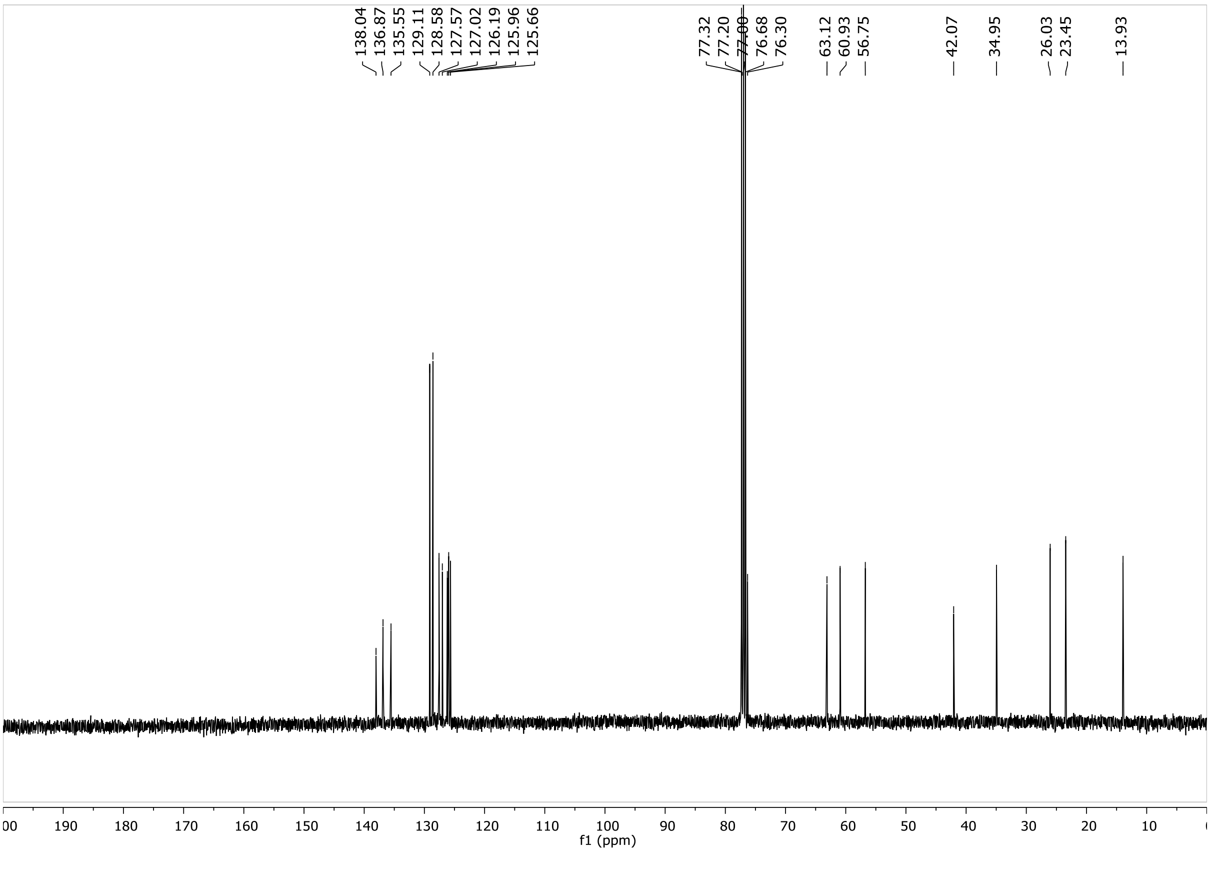
138.04
136.87
135.55
129.11
128.58
127.57
127.02
126.19
125.96
125.66
77.32
77.20
77.00
76.68
76.30
63.12
60.93
56.75
42.07
34.95
26.03
23.45
13.93
190 180 170 160 150 140 130 120 110 100 90 80 70 60 50 40 30 20 10
f1 (ppm)

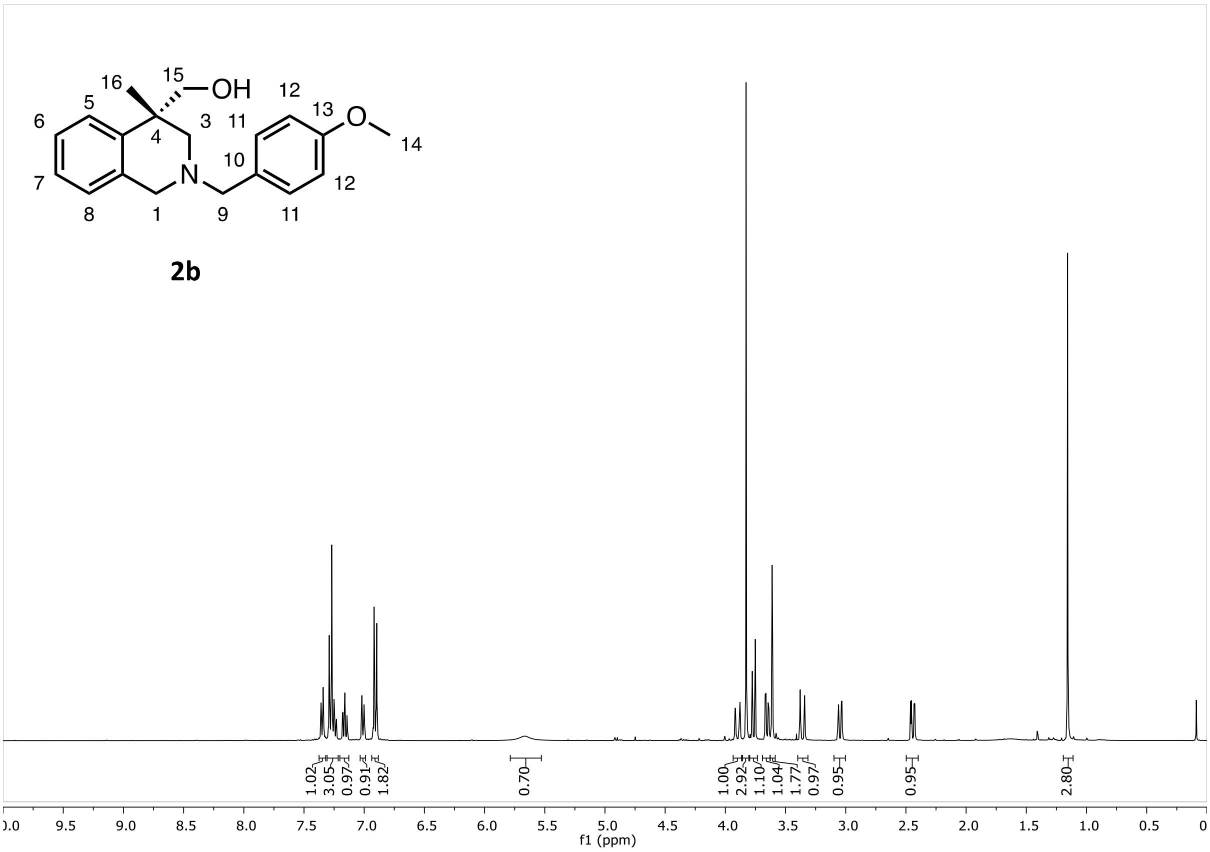
2b
1.02
3.05
0.97
0.91
1.82
0.70
1.00
2.92
1.10
1.04
1.77
0.97
0.95
0.95
2.80
f1 (ppm)

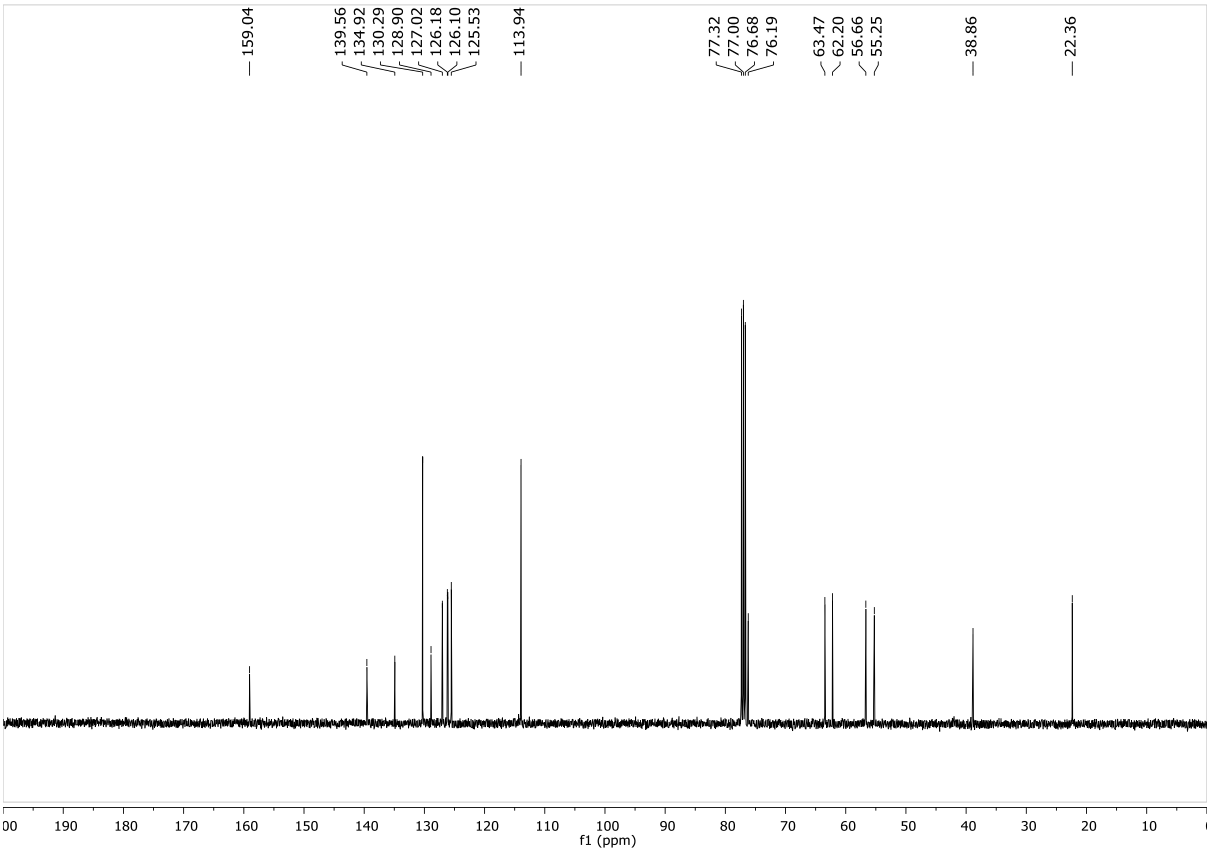
159.04
139.56
134.92
130.29
128.90
127.02
126.18
126.10
125.53
113.94
77.32
77.00
76.68
76.19
63.47
62.20
56.66
55.25
38.86
22.36
f1 (ppm)

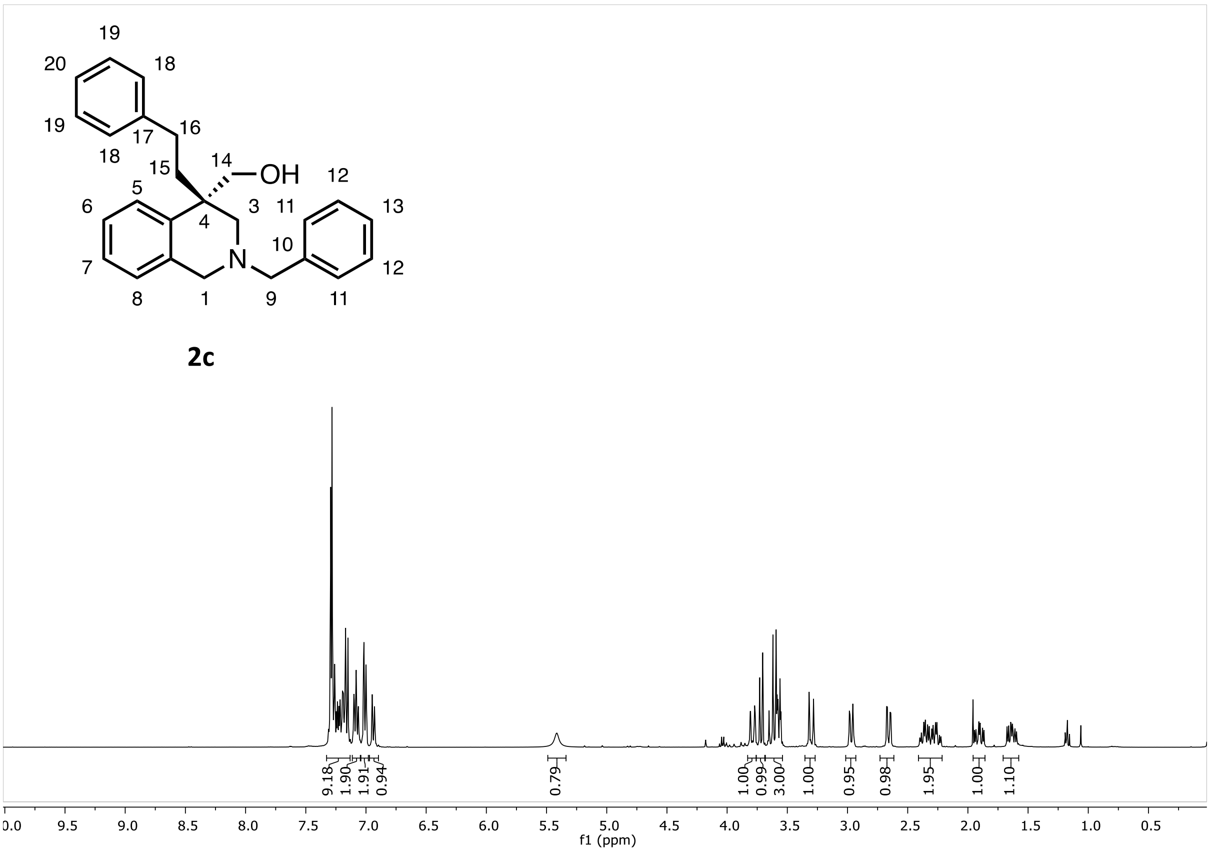
2c
OH
N
f1 (ppm)

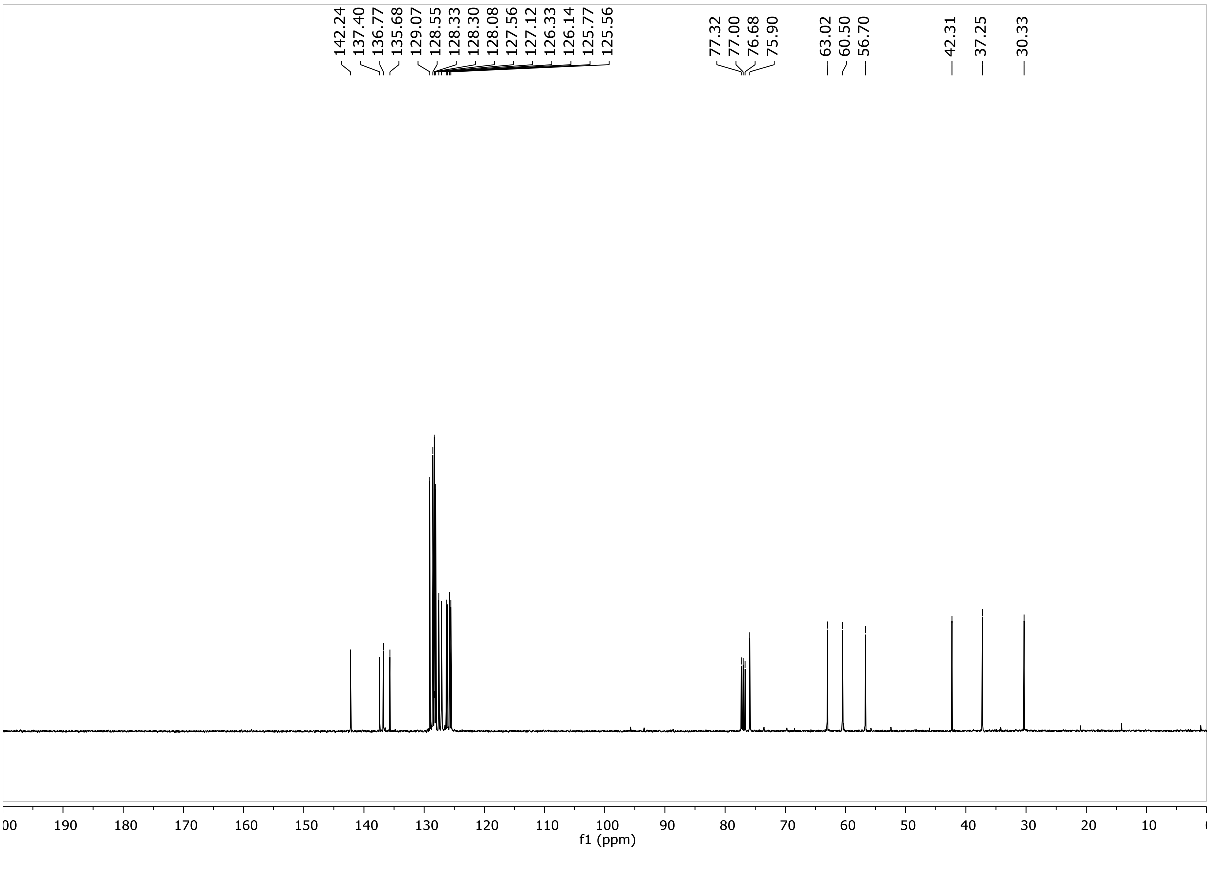
142.24
137.40
136.77
135.68
129.07
128.55
128.33
128.30
128.08
127.56
127.12
126.33
126.14
125.77
125.56
77.32
77.00
76.68
75.90
63.02
60.50
56.70
42.31
37.25
30.33
f1 (ppm)

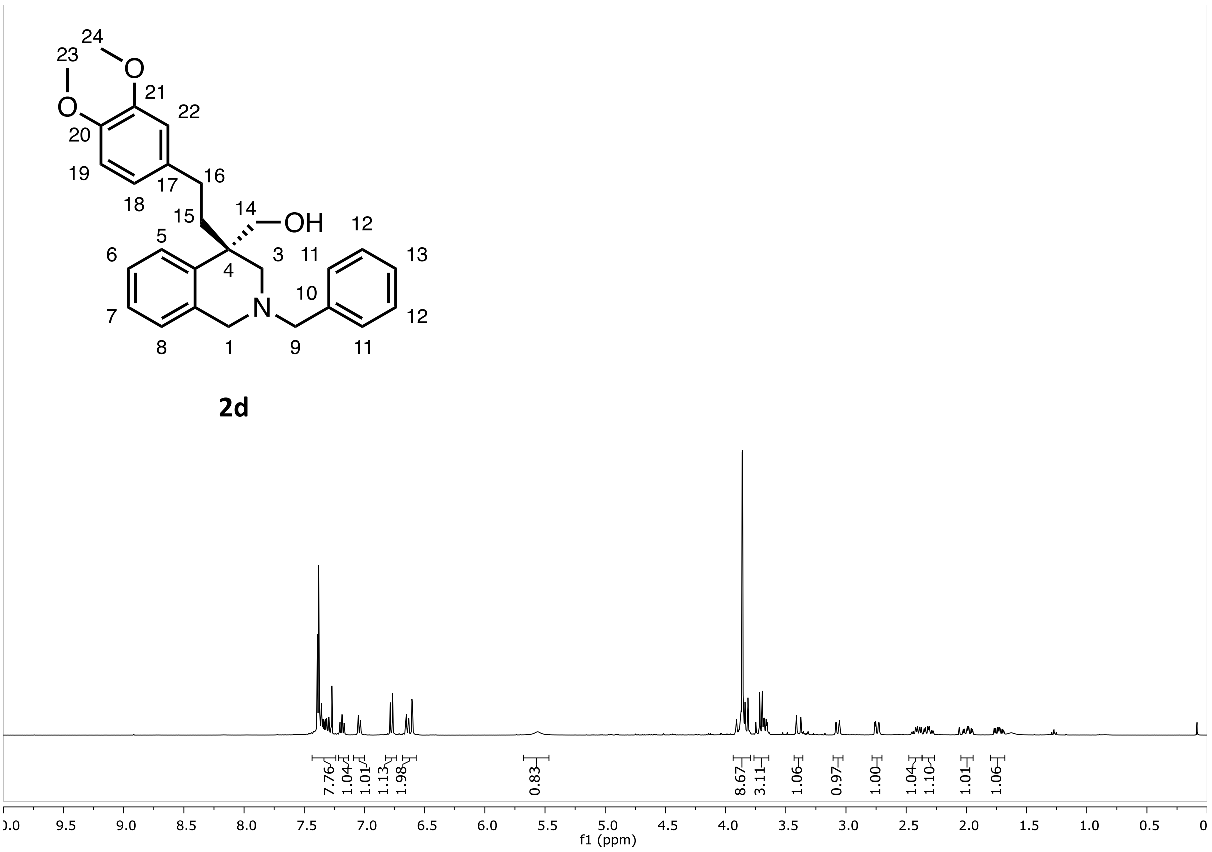

OH
N
O
O
2d
7.76
1.04
1.01
1.13
1.98
0.83
8.67
3.11
1.06
0.97
1.00
1.04
1.10
1.01
1.06
f1 (ppm)

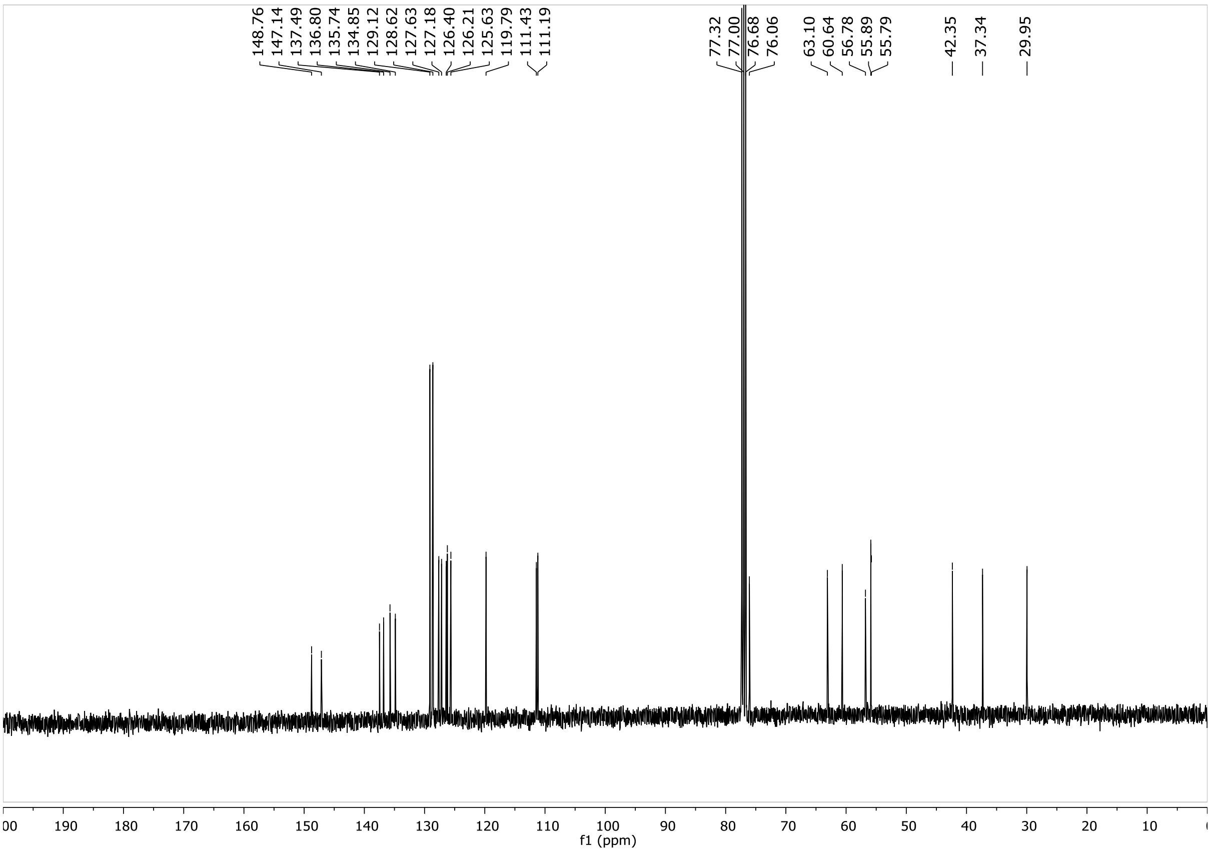

148.76
147.14
137.49
136.80
135.74
134.85
129.12
128.62
127.63
127.18
126.40
126.21
125.63
119.79
111.43
111.19
77.32
77.00
76.68
76.06
63.10
60.64
56.78
55.89
55.79
42.35
37.34
29.95
f1 (ppm)

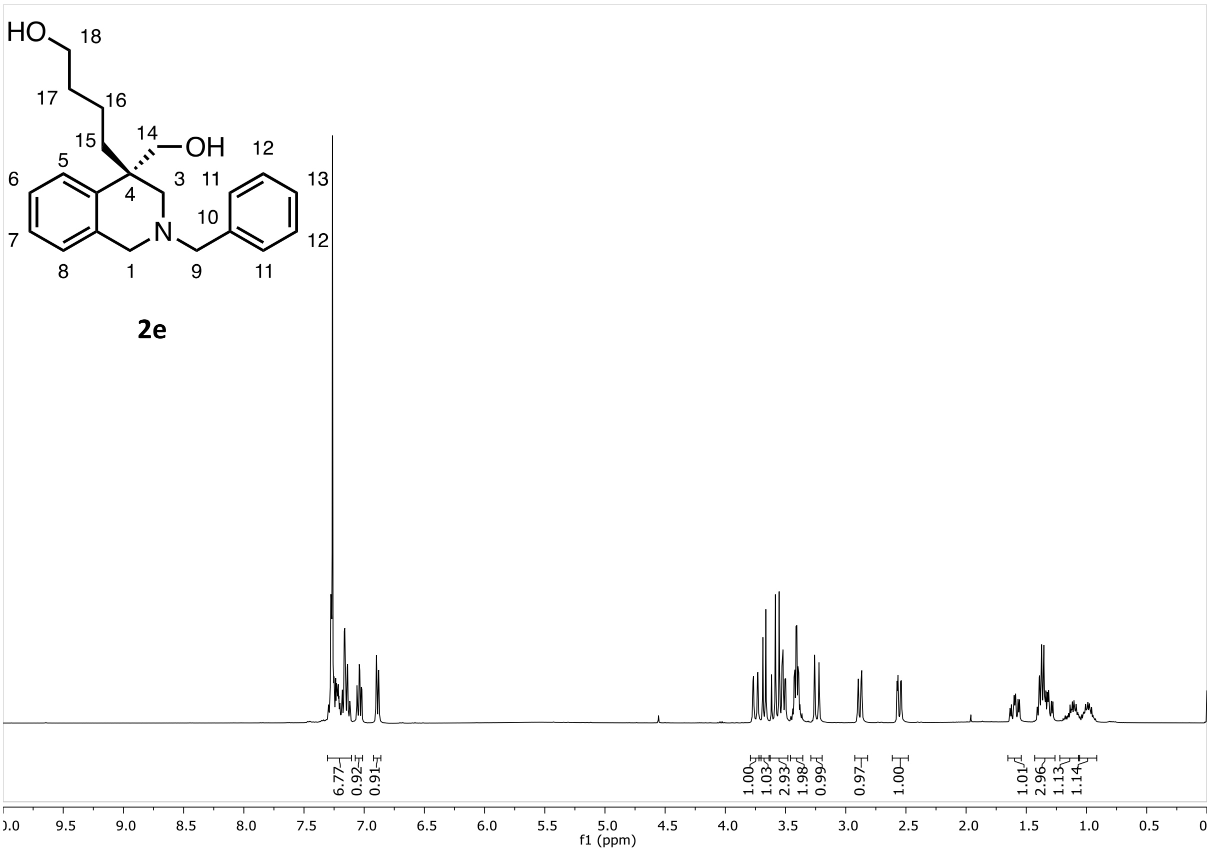
HO
OH
N
2e
6.77
0.92
0.91
1.00
1.03
2.93
1.98
0.99
0.97
1.00
1.01
2.96
1.13
1.14
f1 (ppm)

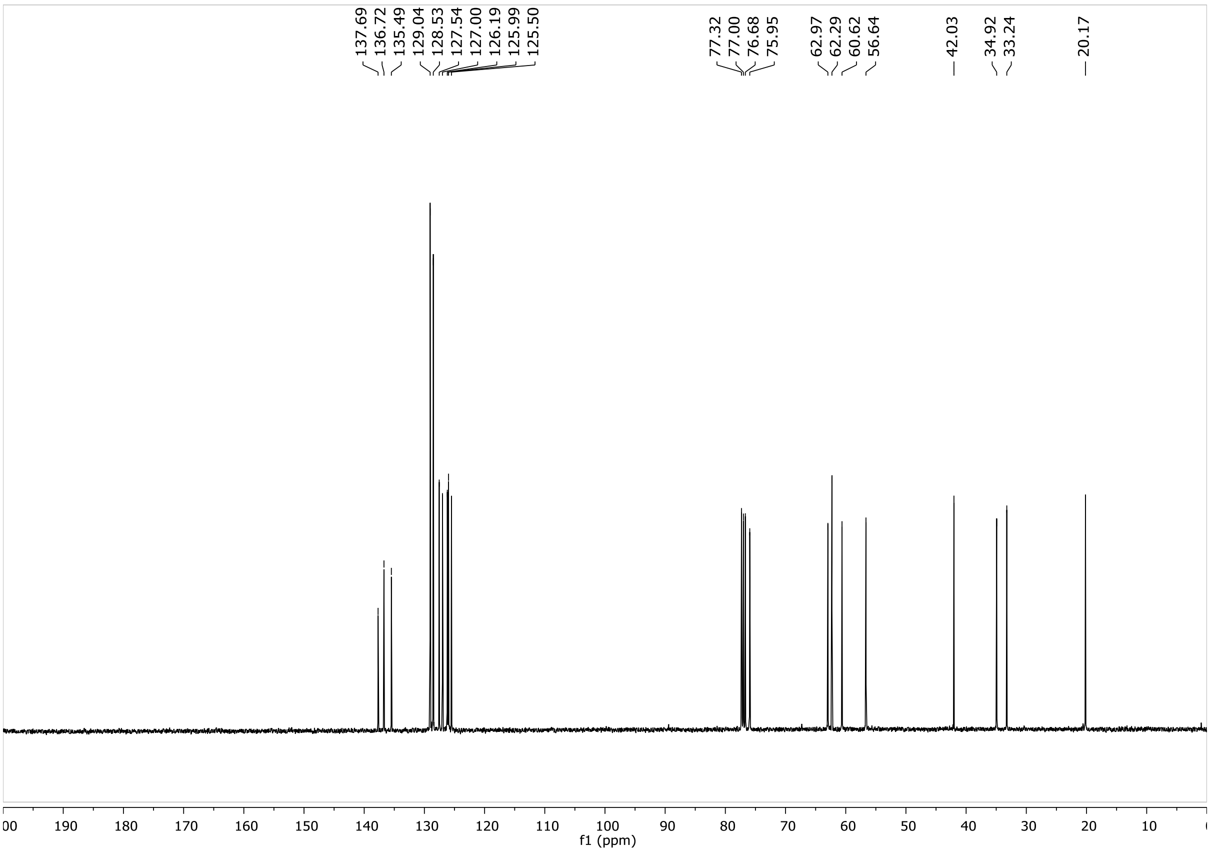
137.69
136.72
135.49
129.04
128.53
127.54
127.00
126.19
125.99
125.50
77.32
77.00
76.68
75.95
62.97
62.29
60.62
56.64
42.03
34.92
33.24
20.17
f1 (ppm)

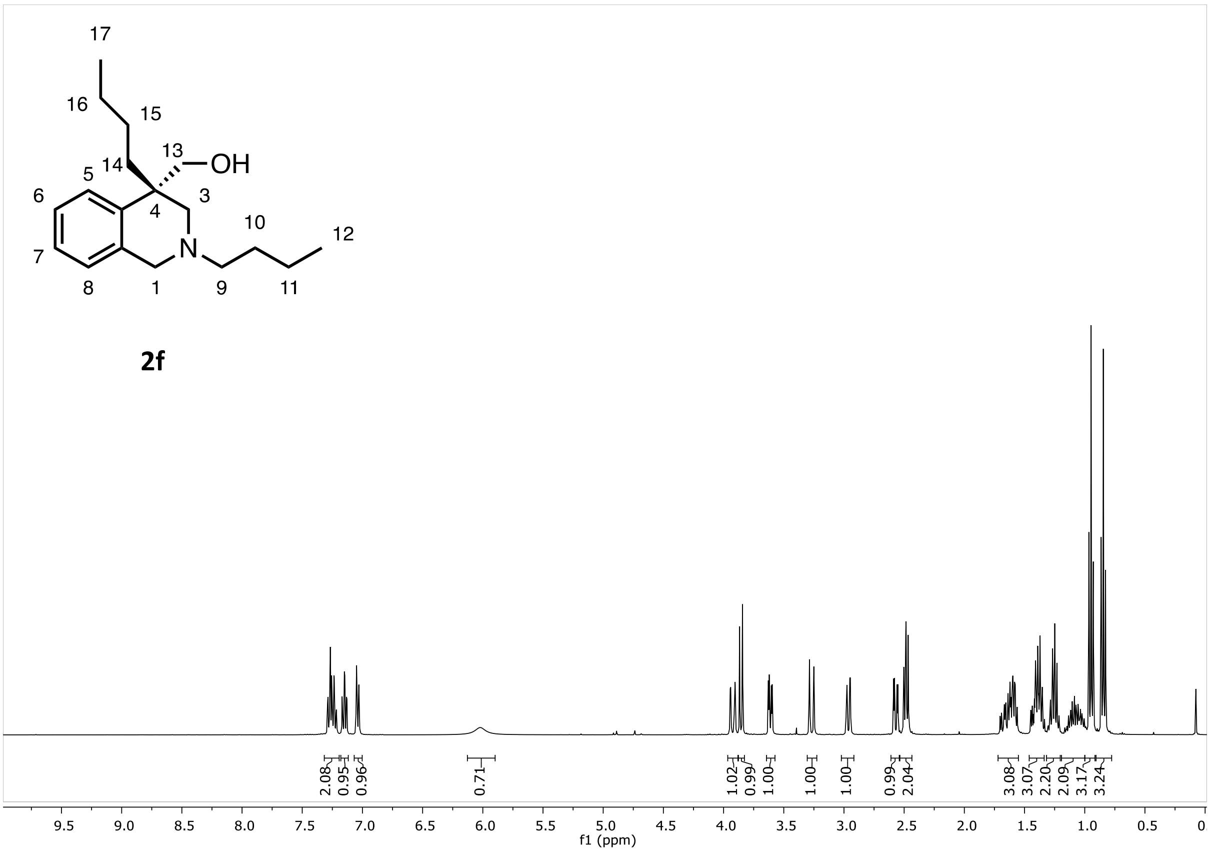
17
16
15
13
OH
14
5
6
4
3
10
N
12
7
8
1
9
11
2f
2.08
0.95
0.96
0.71
1.02
0.99
1.00
1.00
1.00
0.99
2.04
3.08
3.07
2.20
2.09
3.17
3.24
9.5
9.0
8.5
8.0
7.5
7.0
6.5
6.0
5.5
5.0
4.5
4.0
3.5
3.0
2.5
2.0
1.5
1.0
0.5
0
f1 (ppm)

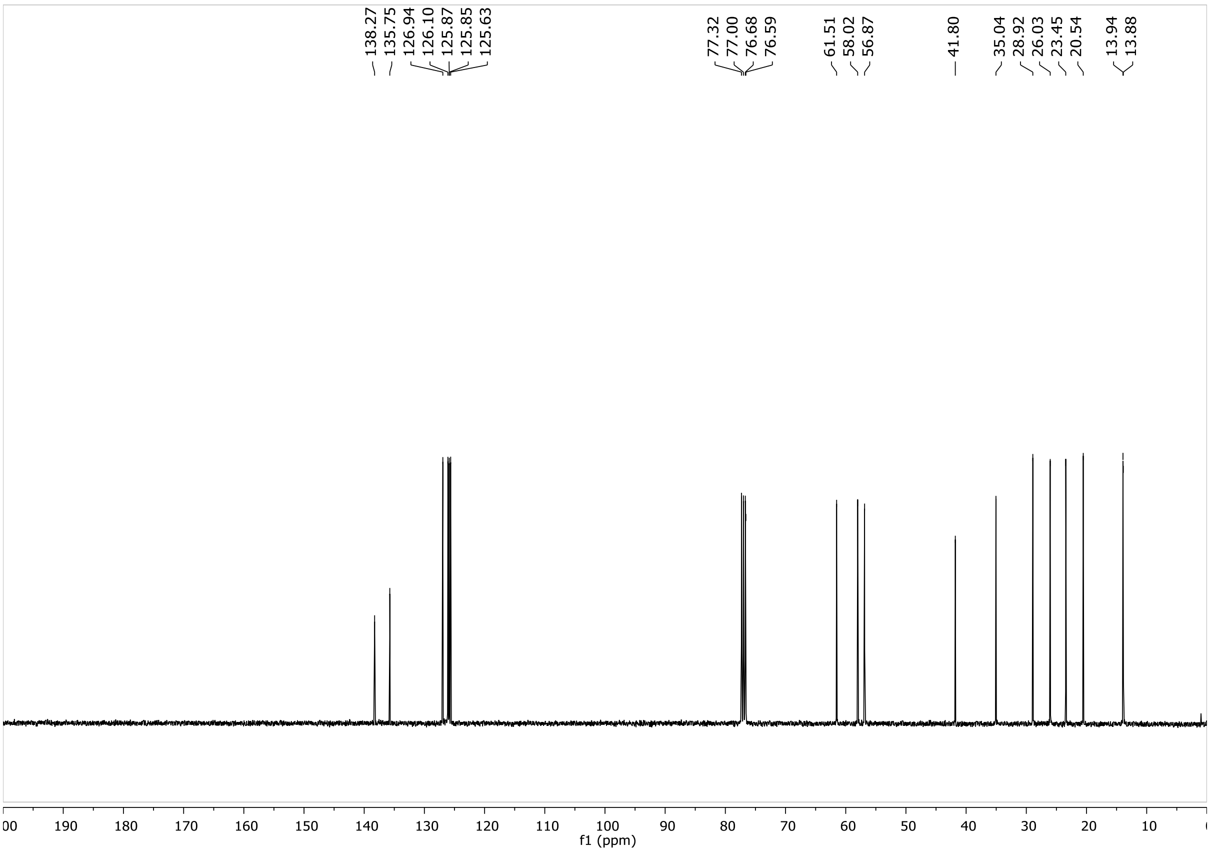
138.27
135.75
126.94
126.10
125.87
125.85
125.63
77.32
77.00
76.68
76.59
61.51
58.02
56.87
41.80
35.04
28.92
26.03
23.45
20.54
13.94
13.88
190
180
170
160
150
140
130
120
110
100
90
80
70
60
50
40
30
20
10
f1 (ppm)

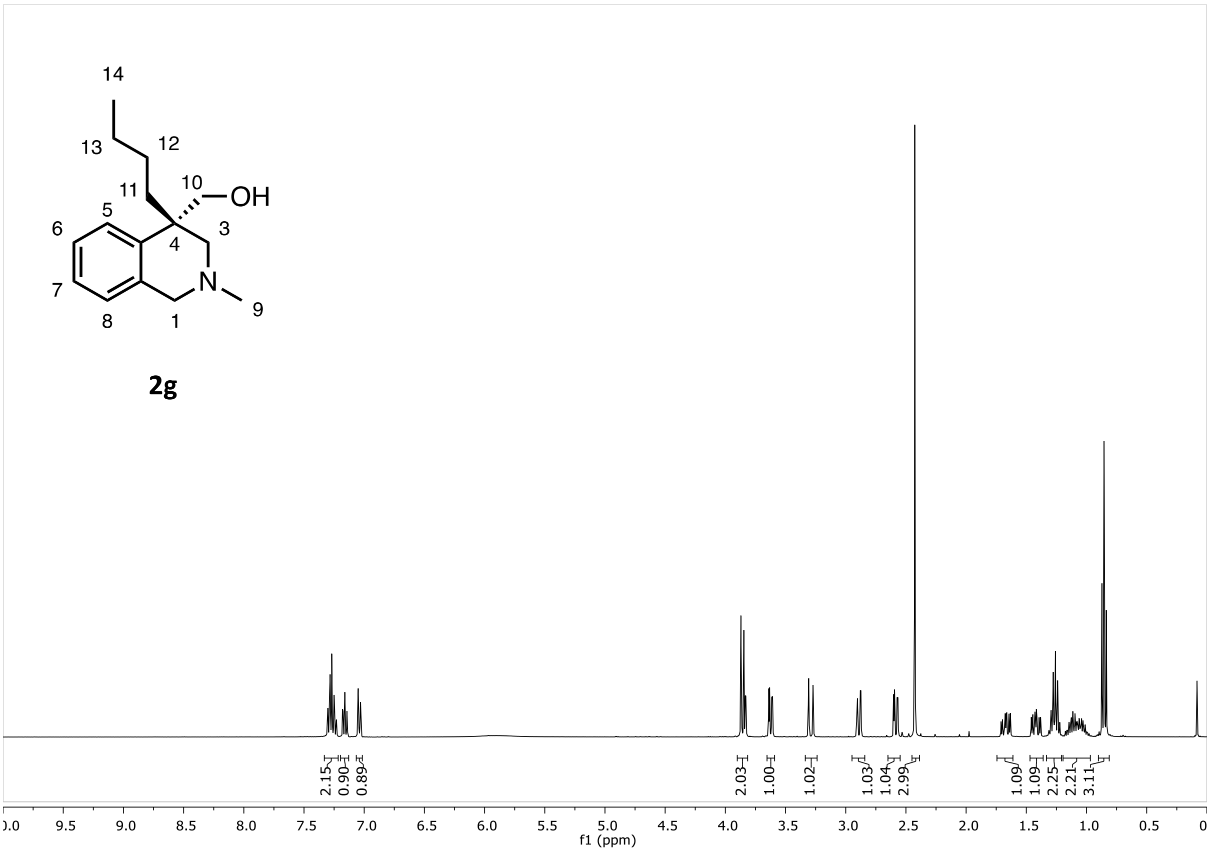
14
13
12
11
10
OH
5
6
4
3
7
N
9
8
1
2g
2.15
0.90
0.89
2.03
1.00
1.02
1.03
1.04
2.99
1.09
1.09
2.25
2.21
3.11
f1 (ppm)

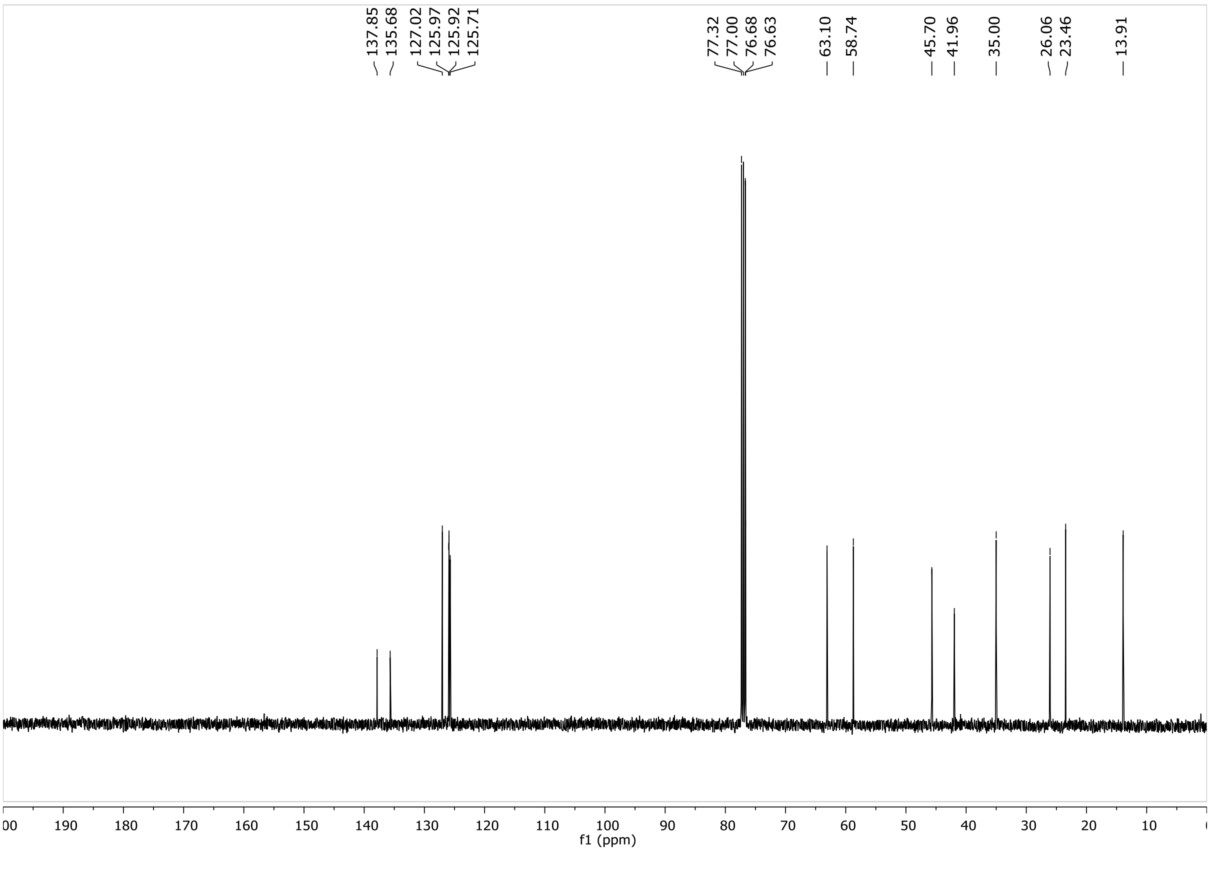
137.85
135.68
127.02
125.97
125.92
125.71
77.32
77.00
76.68
76.63
63.10
58.74
45.70
41.96
35.00
26.06
23.46
13.91
f1 (ppm)

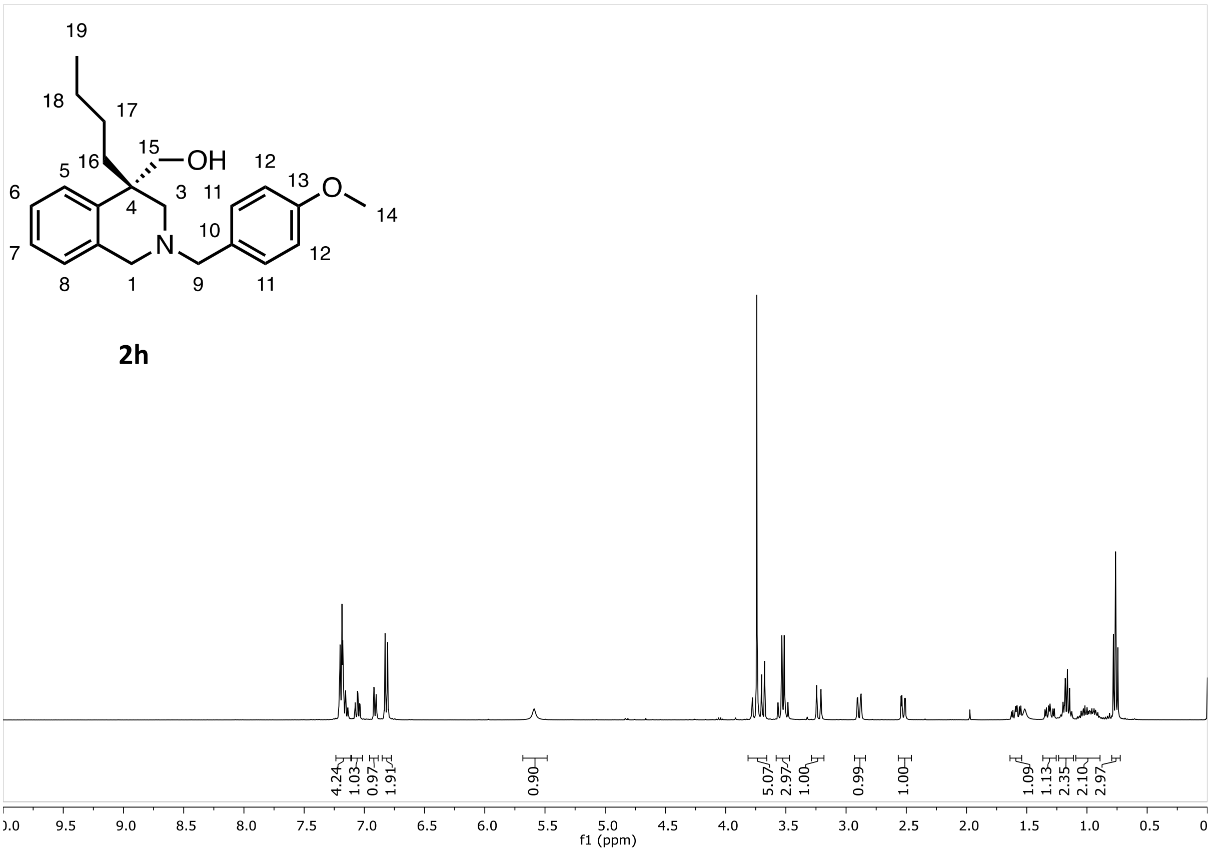
19
18
17
15
OH
16
5
12
13
O
6
3
11
4
14
N
10
7
12
8
1
9
11
2h
4.24
1.03
0.97
1.91
0.90
5.07
2.97
1.00
0.99
1.00
1.09
1.13
2.35
2.10
2.97
f1 (ppm)

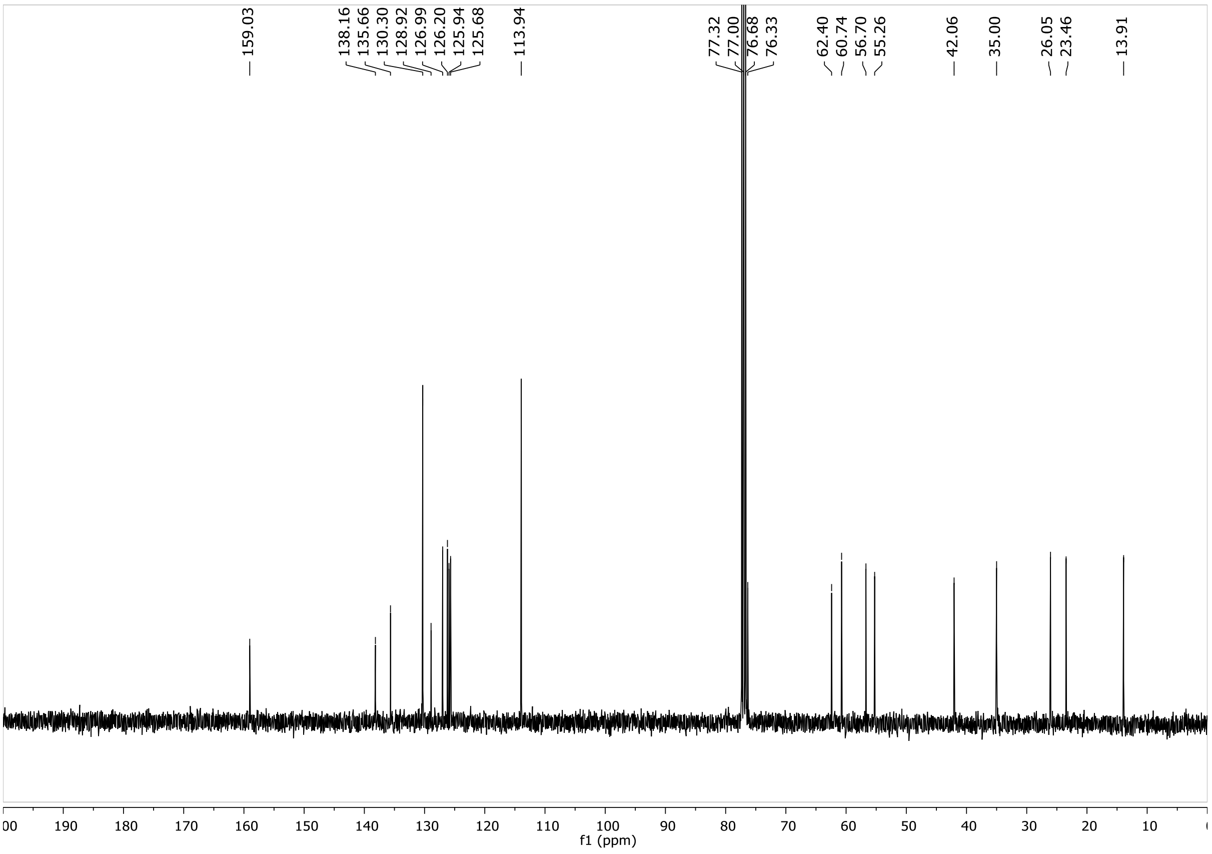
159.03
138.16
135.66
130.30
128.92
126.99
126.20
125.94
125.68
113.94
77.32
77.00
76.68
76.33
62.40
60.74
56.70
55.26
42.06
35.00
26.05
23.46
13.91
f1 (ppm)

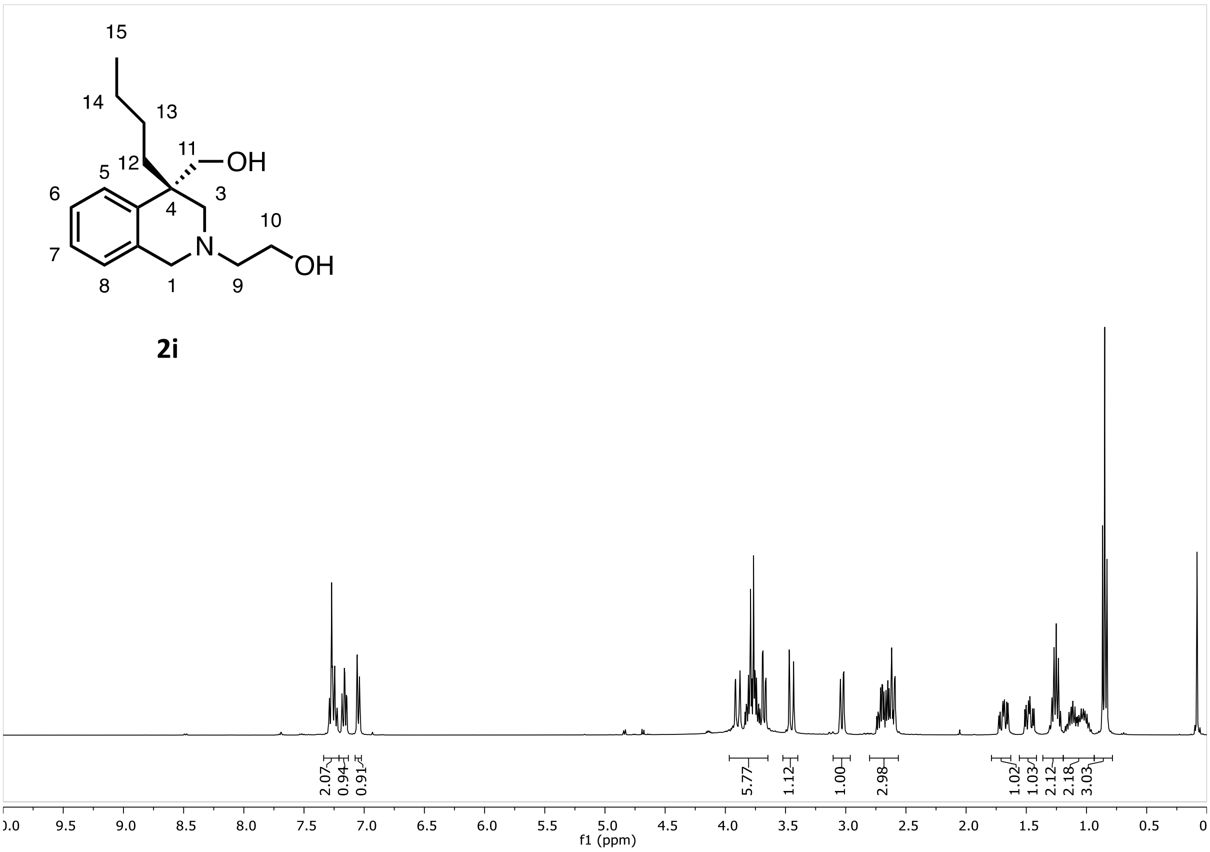

15
14
13
12
11
OH
5
6
4
3
10
7
N
OH
8
1
9
2i
2.07
0.94
0.91
5.77
1.12
1.00
2.98
1.02
1.03
2.12
2.18
3.03
f1 (ppm)

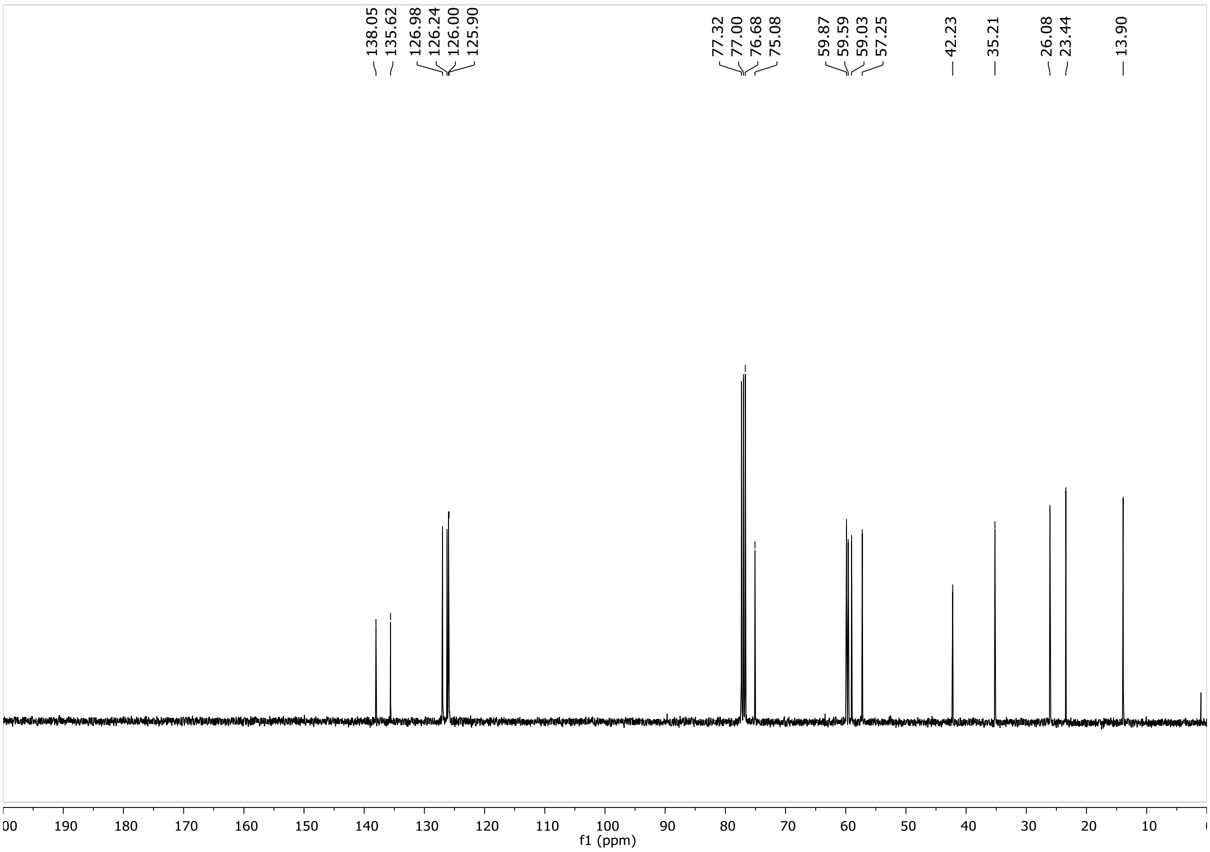

138.05
135.62
126.98
126.24
126.00
125.90
77.32
77.00
76.68
75.08
59.87
59.59
59.03
57.25
42.23
35.21
26.08
23.44
13.90
f1 (ppm)

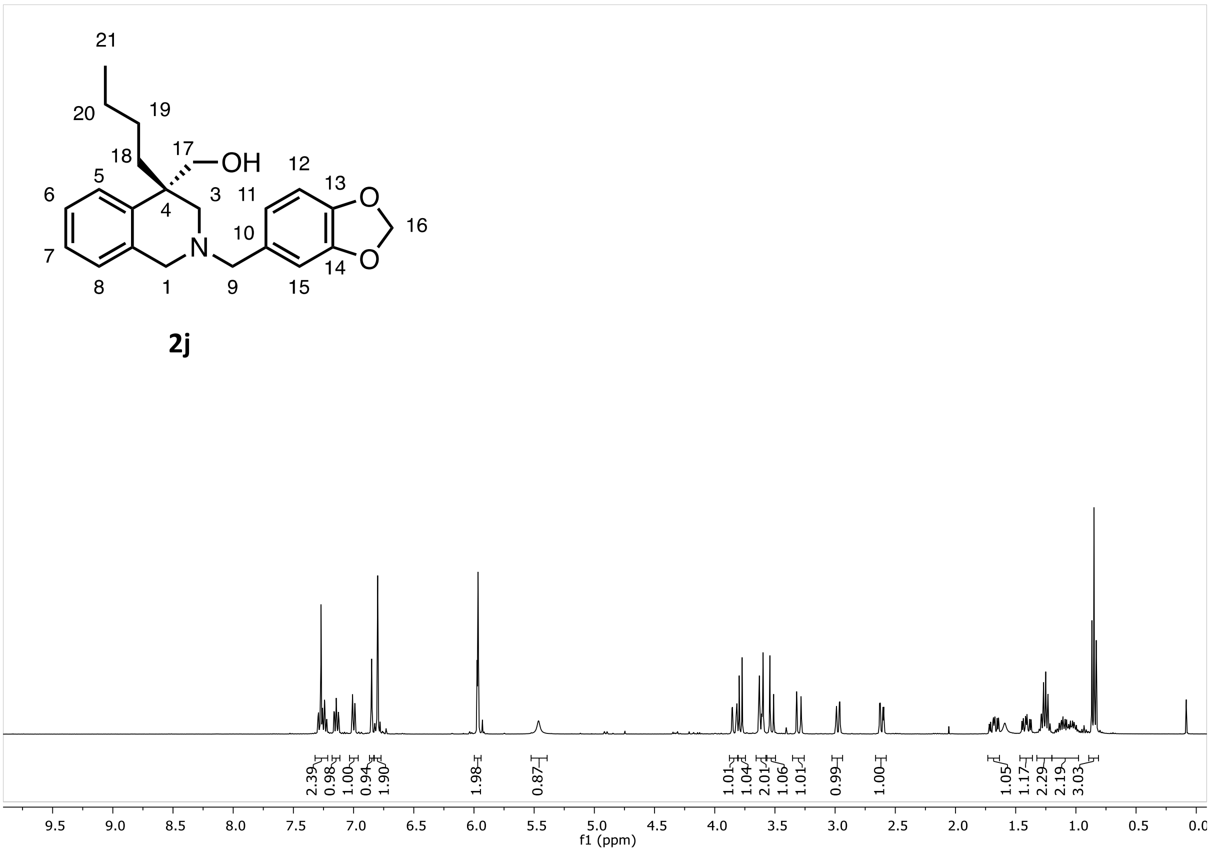
21
20
19
18
17
OH
12
13
5
6
4
3
11
O
16
10
N
7
O
14
8
1
9
15
2j
2.39
0.98
1.00
0.94
1.90
1.98
0.87
1.01
1.04
2.01
1.06
1.01
0.99
1.00
1.05
1.17
2.29
2.19
3.03
9.5
9.0
8.5
8.0
7.5
7.0
6.5
6.0
5.5
5.0
4.5
4.0
3.5
3.0
2.5
2.0
1.5
1.0
0.5
f1 (ppm)

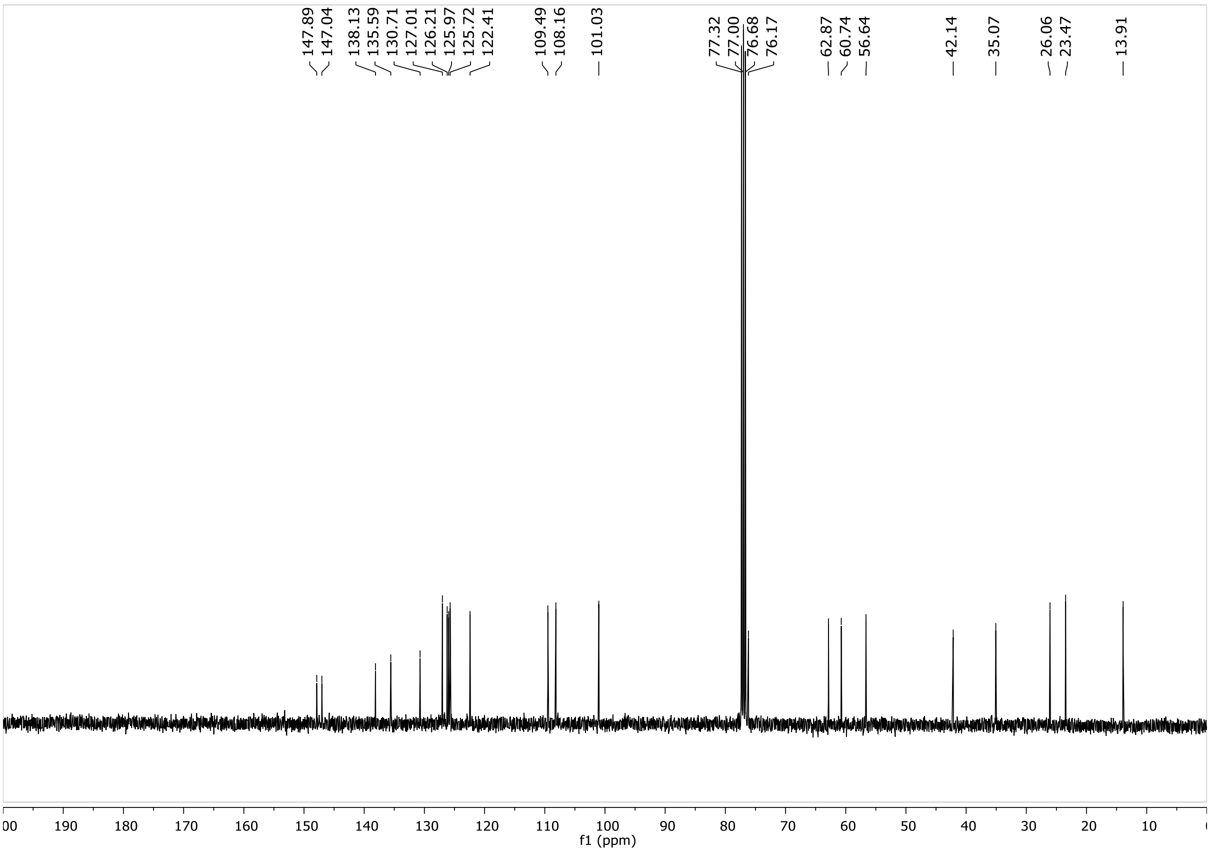
147.89
147.04
138.13
135.59
130.71
127.01
126.21
125.97
125.72
122.41
109.49
108.16
101.03
77.32
77.00
76.68
76.17
62.87
60.74
56.64
42.14
35.07
26.06
23.47
13.91
190
180
170
160
150
140
130
120
110
100
90
80
70
60
50
40
30
20
10
f1 (ppm)

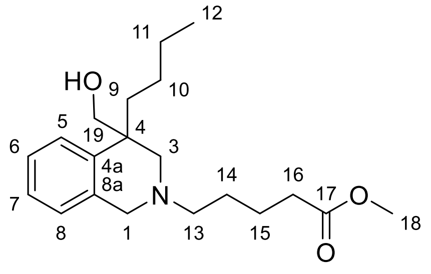

2k

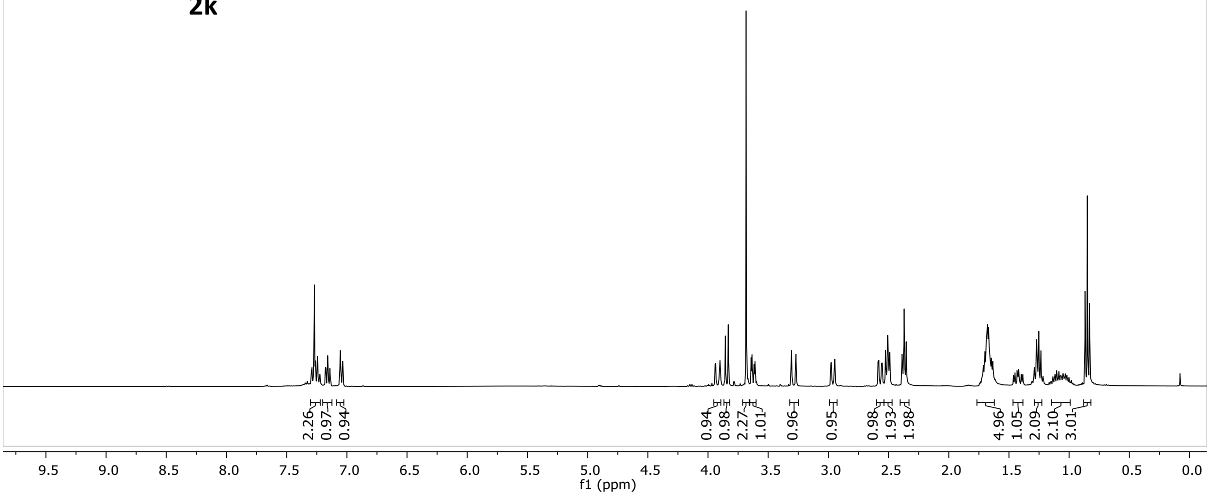

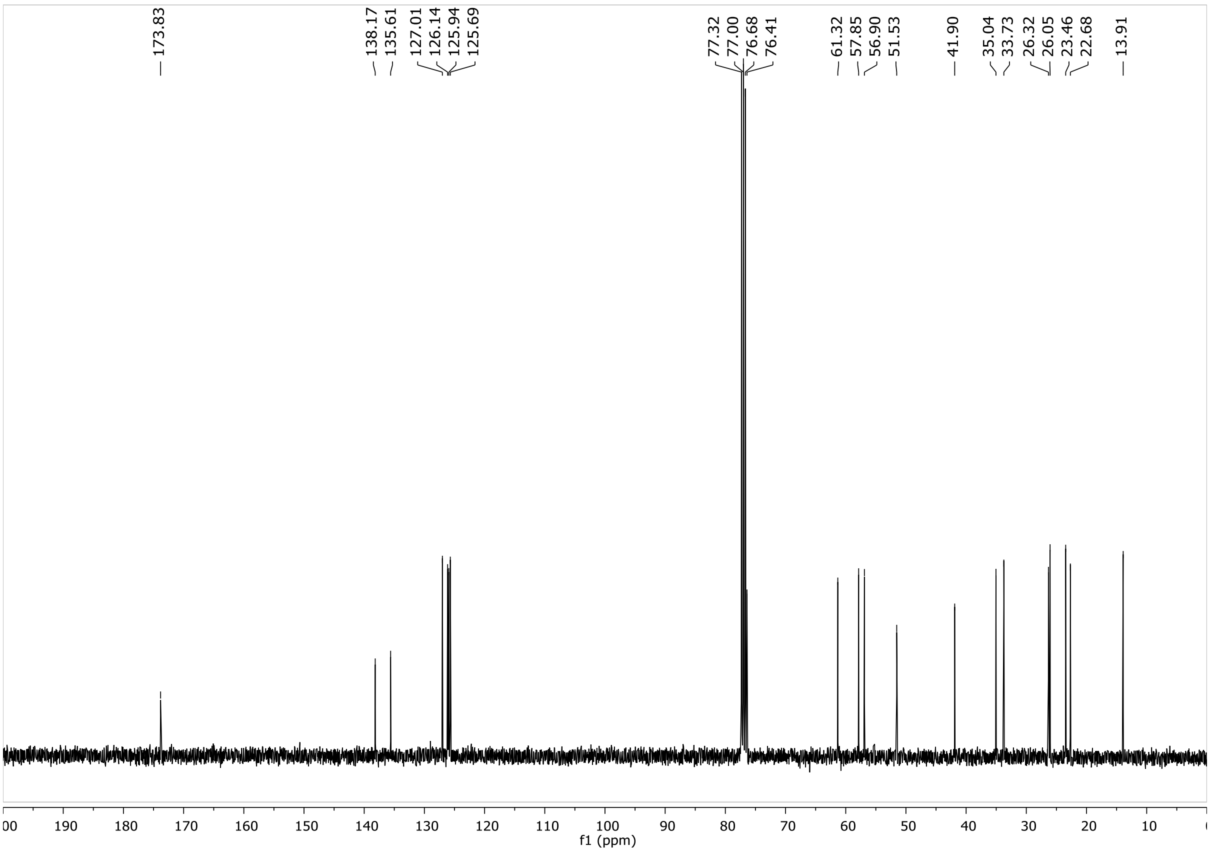

173.83
138.17
135.61
127.01
126.14
125.94
125.69
77.32
77.00
76.68
76.41
61.32
57.85
56.90
51.53
41.90
35.04
33.73
26.32
26.05
23.46
22.68
13.91
f1 (ppm)

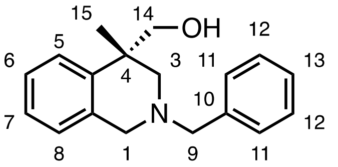
15
14
OH
12
5
6
3
11
13
4
10
7
N
12
8
1
9
11

2o

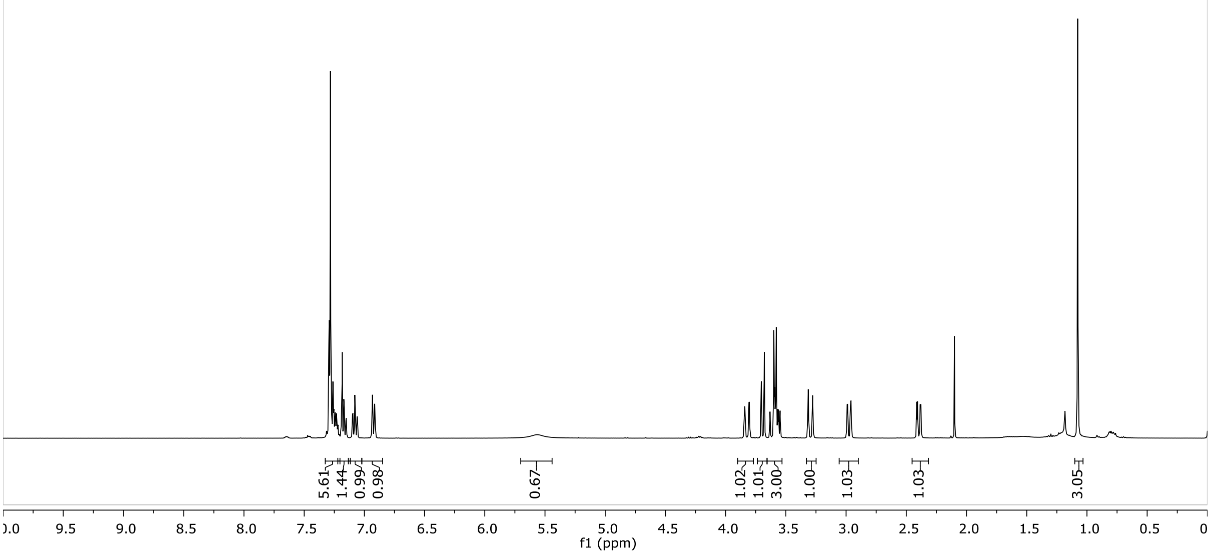
5.61
1.44
0.99
0.98
0.67
1.02
1.01
3.00
1.00
1.03
1.03
3.05
f1 (ppm)

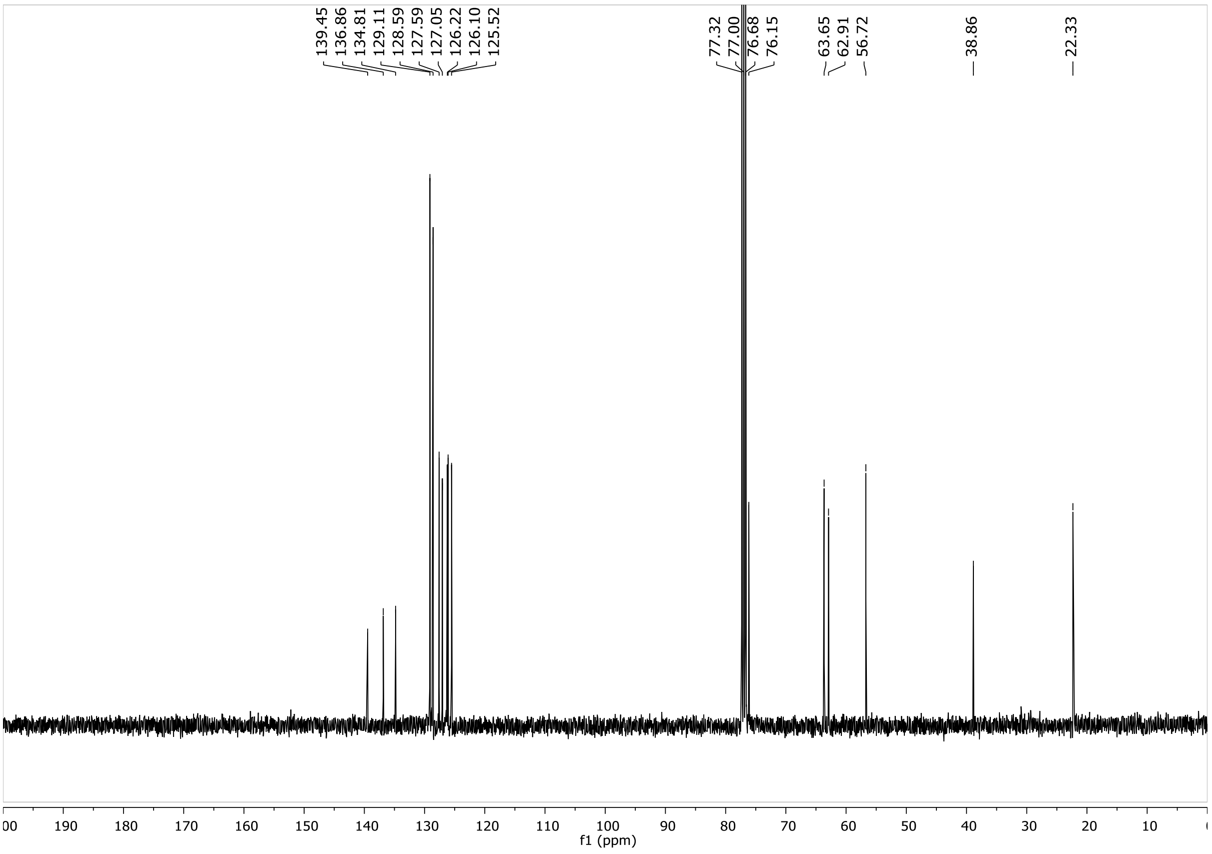
139.45
136.86
134.81
129.11
128.59
127.59
127.05
126.22
126.10
125.52
77.32
77.00
76.68
76.15
63.65
62.91
56.72
38.86
22.33
f1 (ppm)

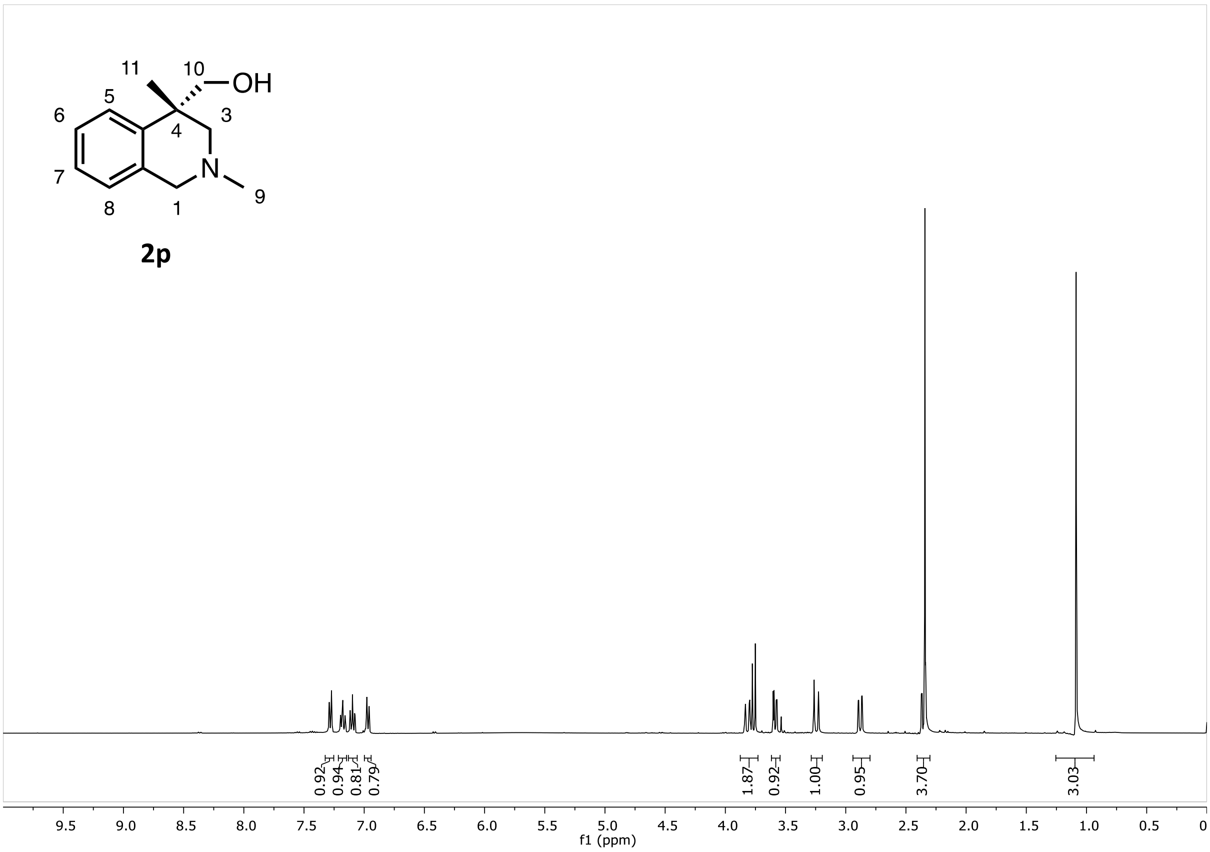

11
10
OH
6
5
4
3
N
7
9
8
1
2p
0.92
0.94
0.81
0.79
1.87
0.92
1.00
0.95
3.70
3.03
f1 (ppm)

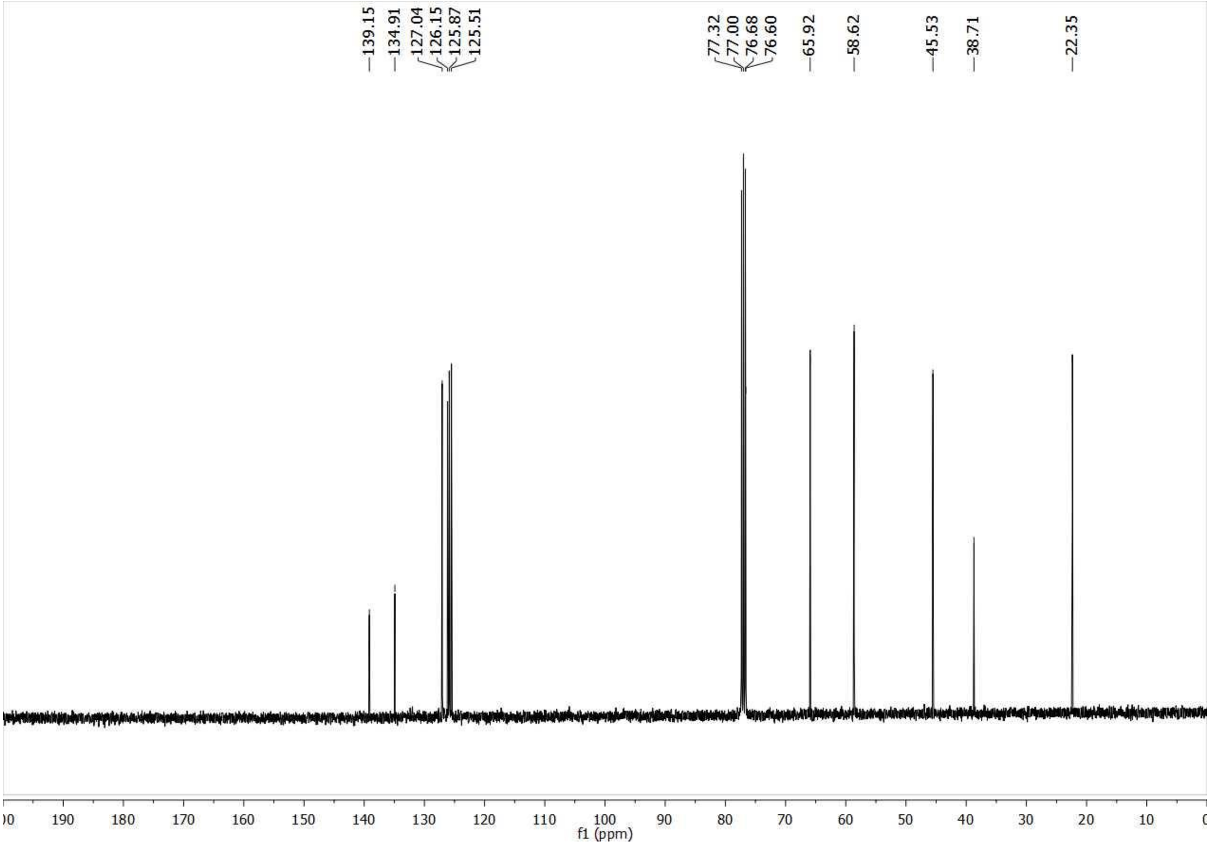

139.15
134.91
127.04
126.15
125.87
125.51
77.32
77.00
76.68
76.60
65.92
58.62
45.53
38.71
22.35
f1 (ppm)

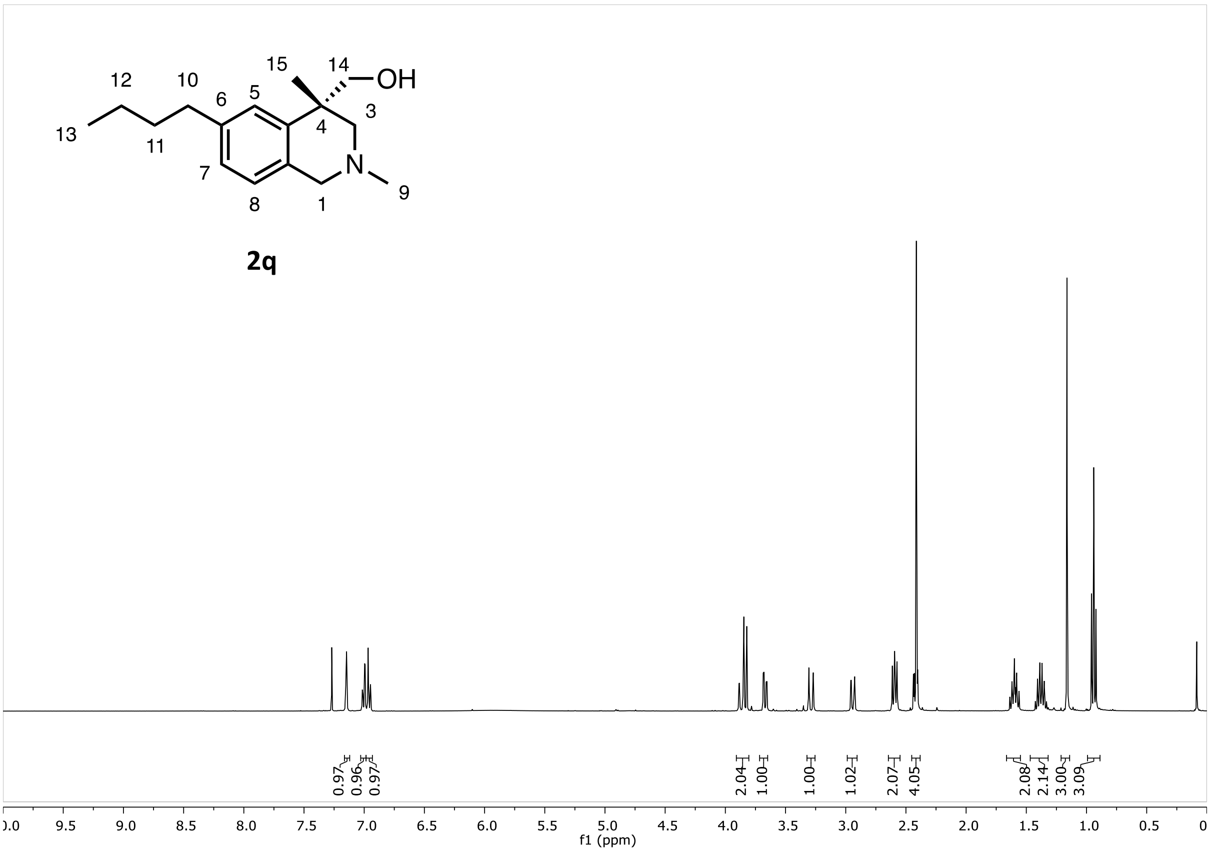

2q
0.97
0.96
0.97
2.04
1.00
1.00
1.02
2.07
4.05
2.08
2.14
3.00
3.09
f1 (ppm)

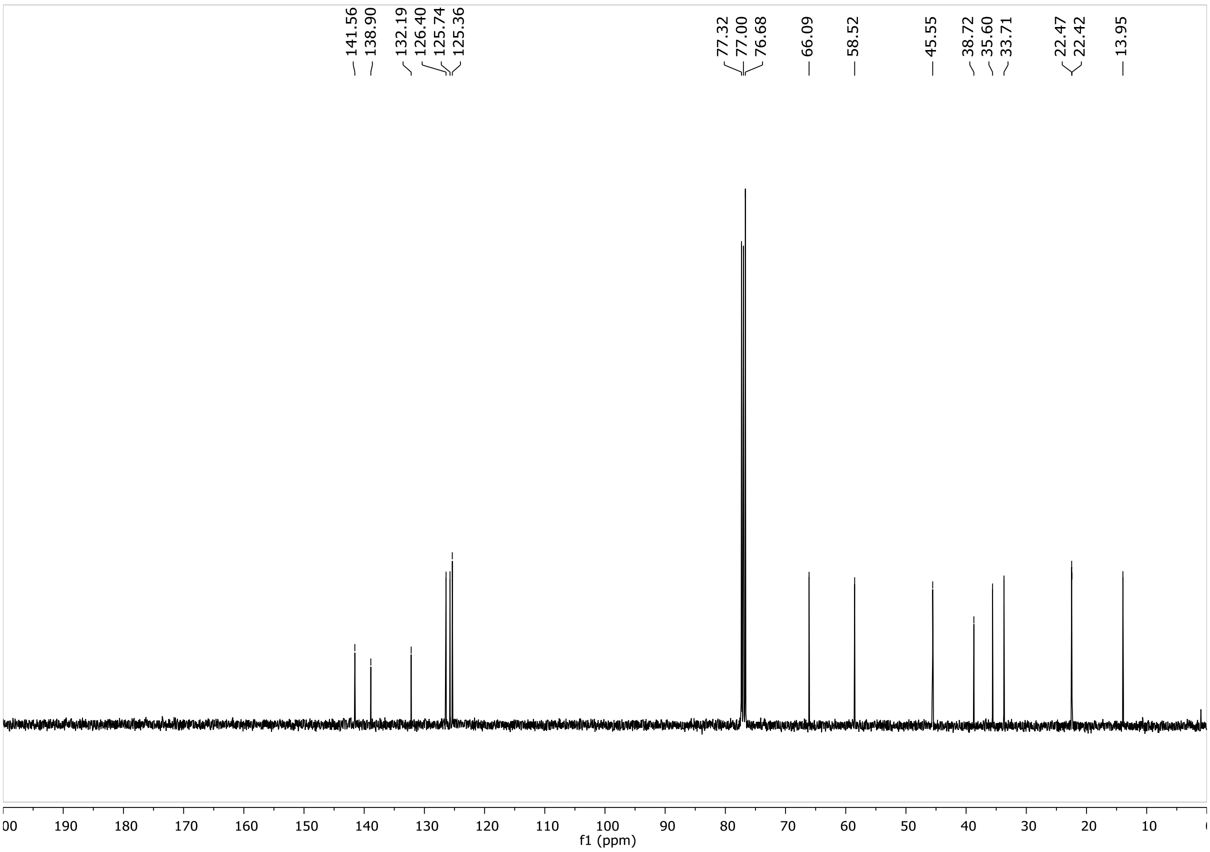

141.56
138.90
132.19
126.40
125.74
125.36
77.32
77.00
76.68
66.09
58.52
45.55
38.72
35.60
33.71
22.47
22.42
13.95
f1 (ppm)

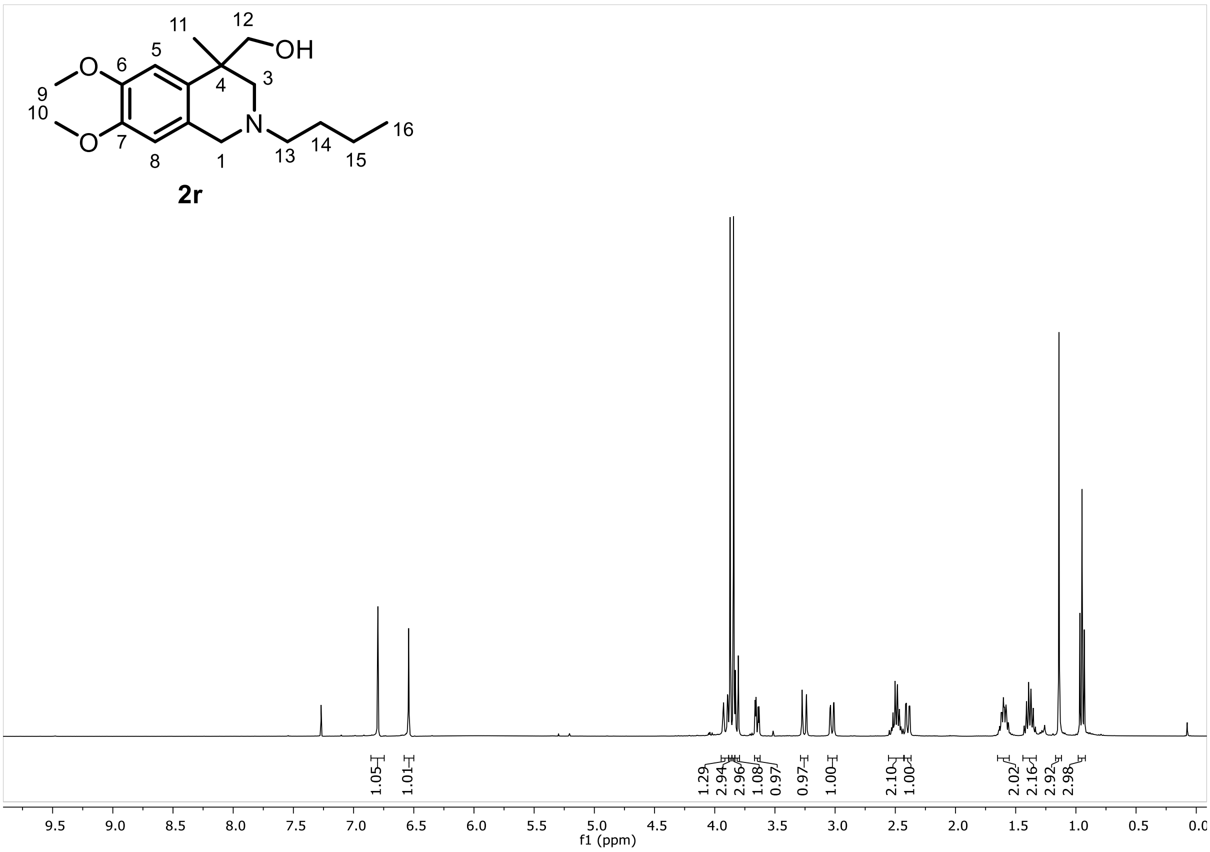

2r
1.05
1.01
1.29
2.94
2.96
1.08
0.97
0.97
1.00
2.10
1.00
2.02
2.16
2.92
2.98
f1 (ppm)

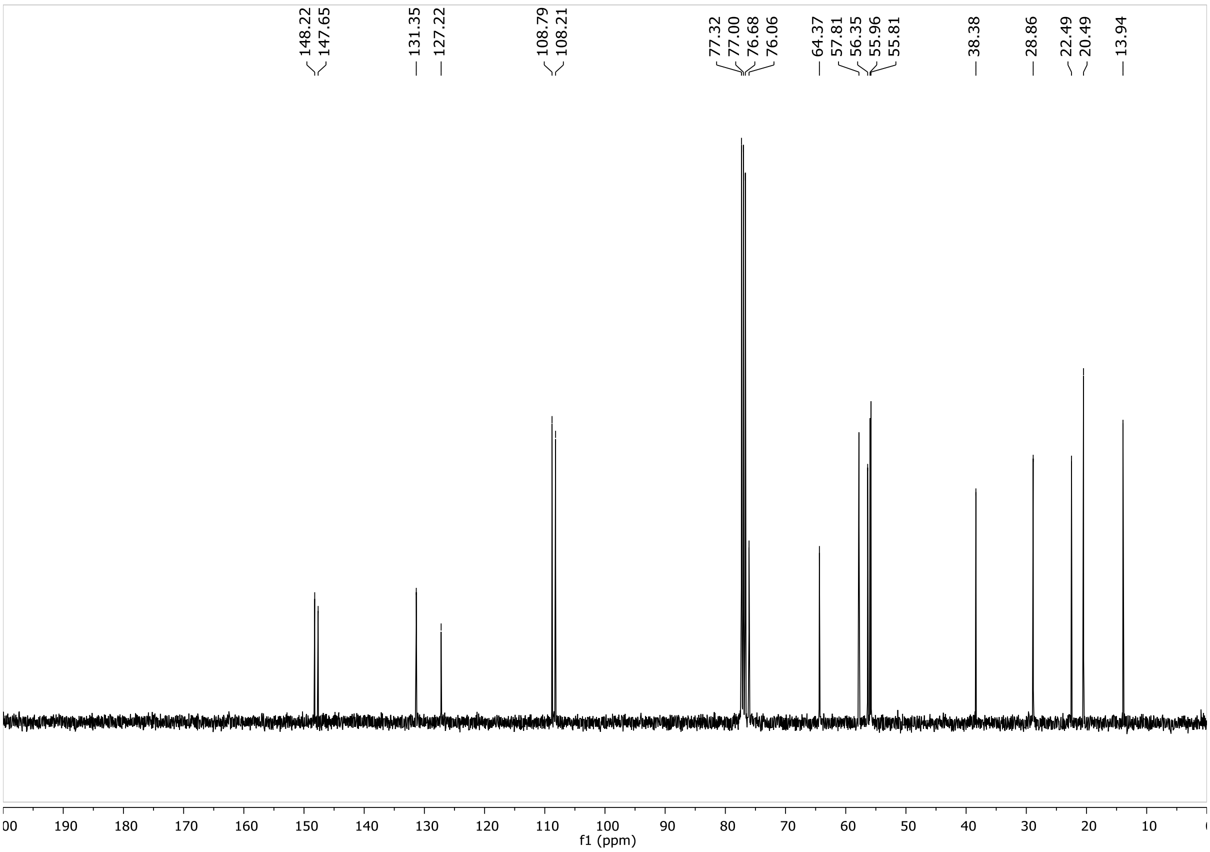

148.22
147.65
131.35
127.22
108.79
108.21
77.32
77.00
76.68
76.06
64.37
57.81
56.35
55.96
55.81
38.38
28.86
22.49
20.49
13.94
f1 (ppm)

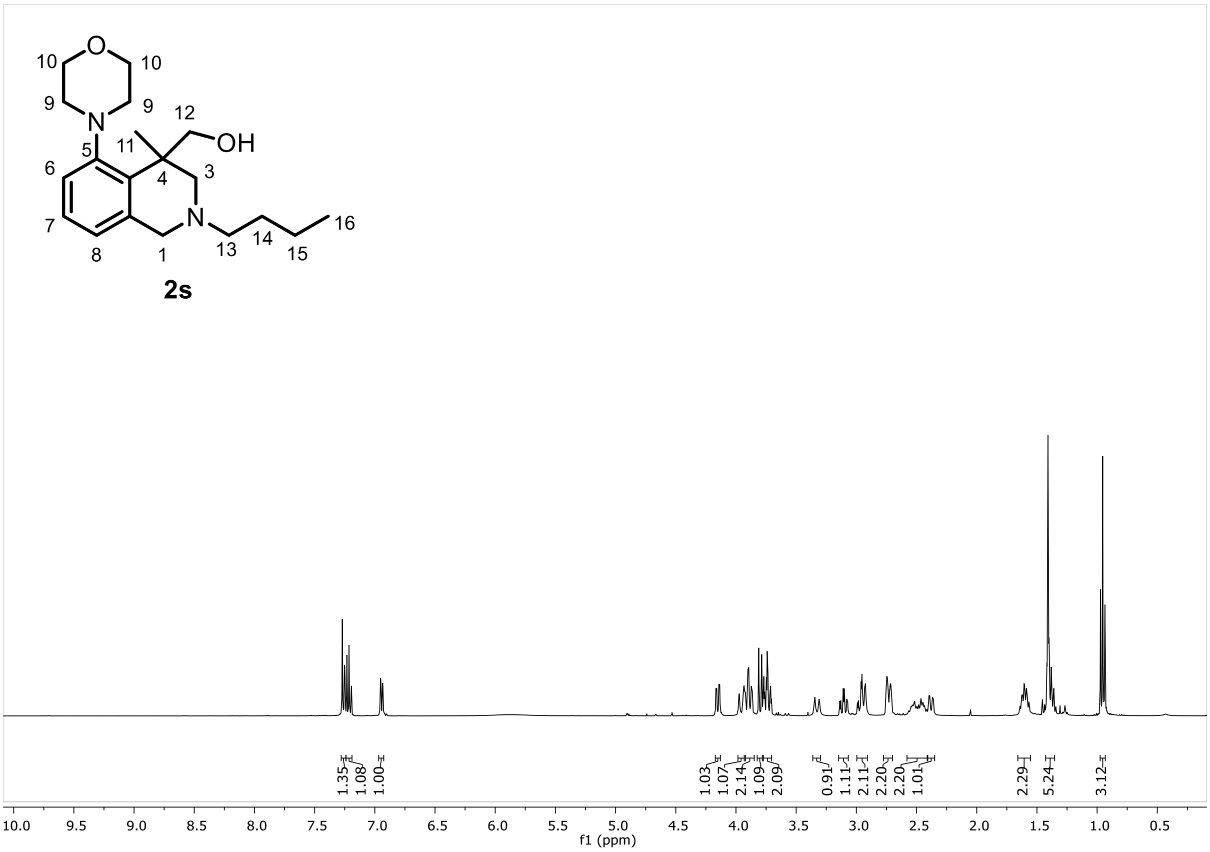
2s
1.35
1.08
1.00
1.03
1.07
2.14
1.09
2.09
0.91
1.11
2.11
2.20
2.20
1.01
2.29
5.24
3.12
f1 (ppm)

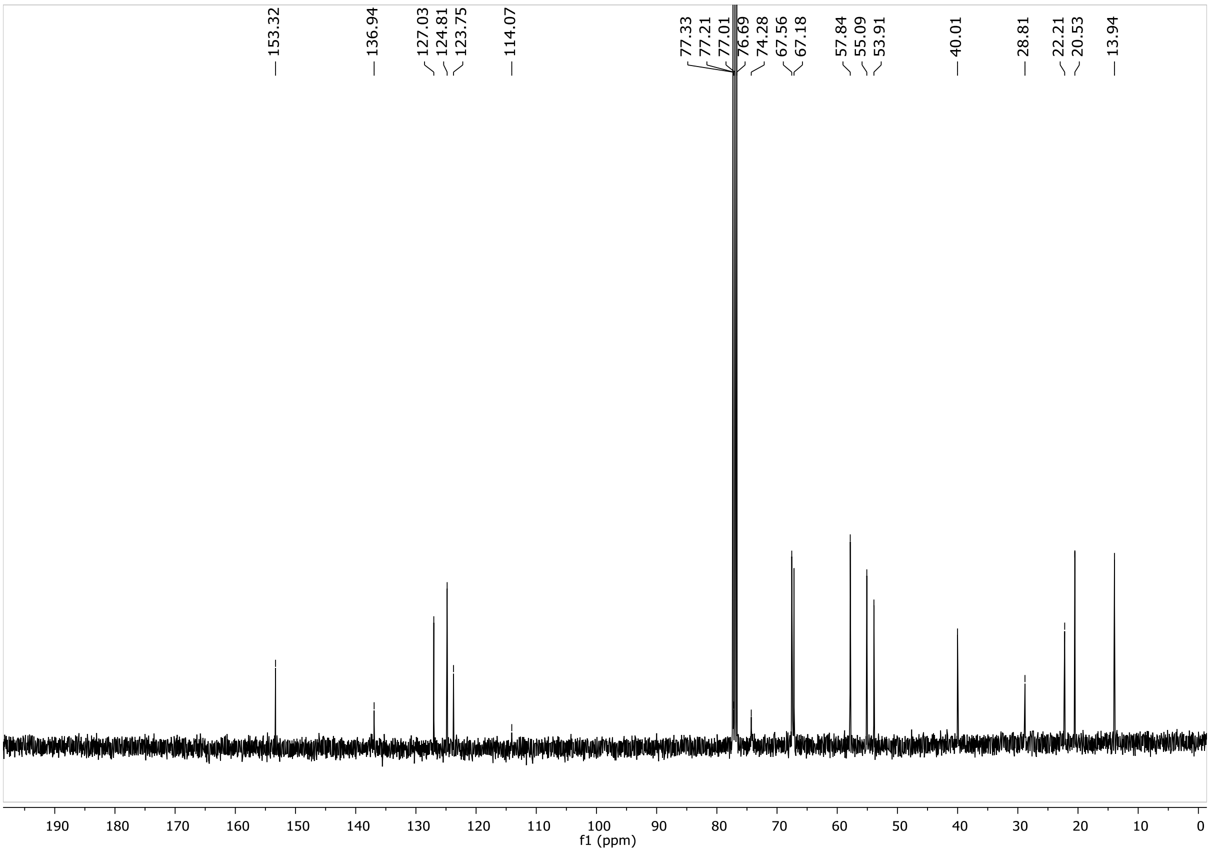
153.32
136.94
127.03
124.81
123.75
114.07
77.33
77.21
77.01
76.69
74.28
67.56
67.18
57.84
55.09
53.91
40.01
28.81
22.21
20.53
13.94
f1 (ppm)

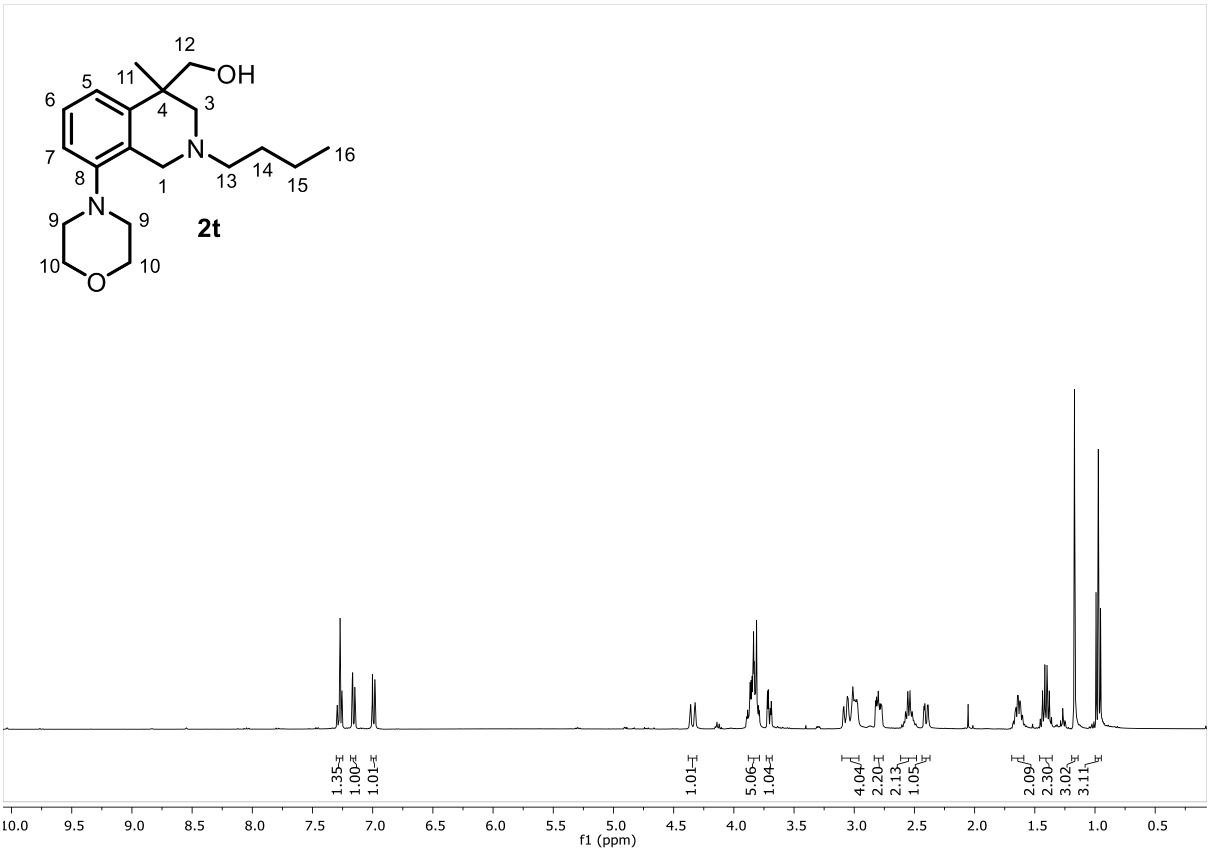
OH
N
O
2t
12
11
5
6
4
3
7
8
1
13
14
15
16
9
9
10
10
1.35
1.00
1.01
1.01
5.06
1.04
4.04
2.20
2.13
1.05
2.09
2.30
3.02
3.11
10.0 9.5 9.0 8.5 8.0 7.5 7.0 6.5 6.0 5.5 5.0 4.5 4.0 3.5 3.0 2.5 2.0 1.5 1.0 0.5
f1 (ppm)

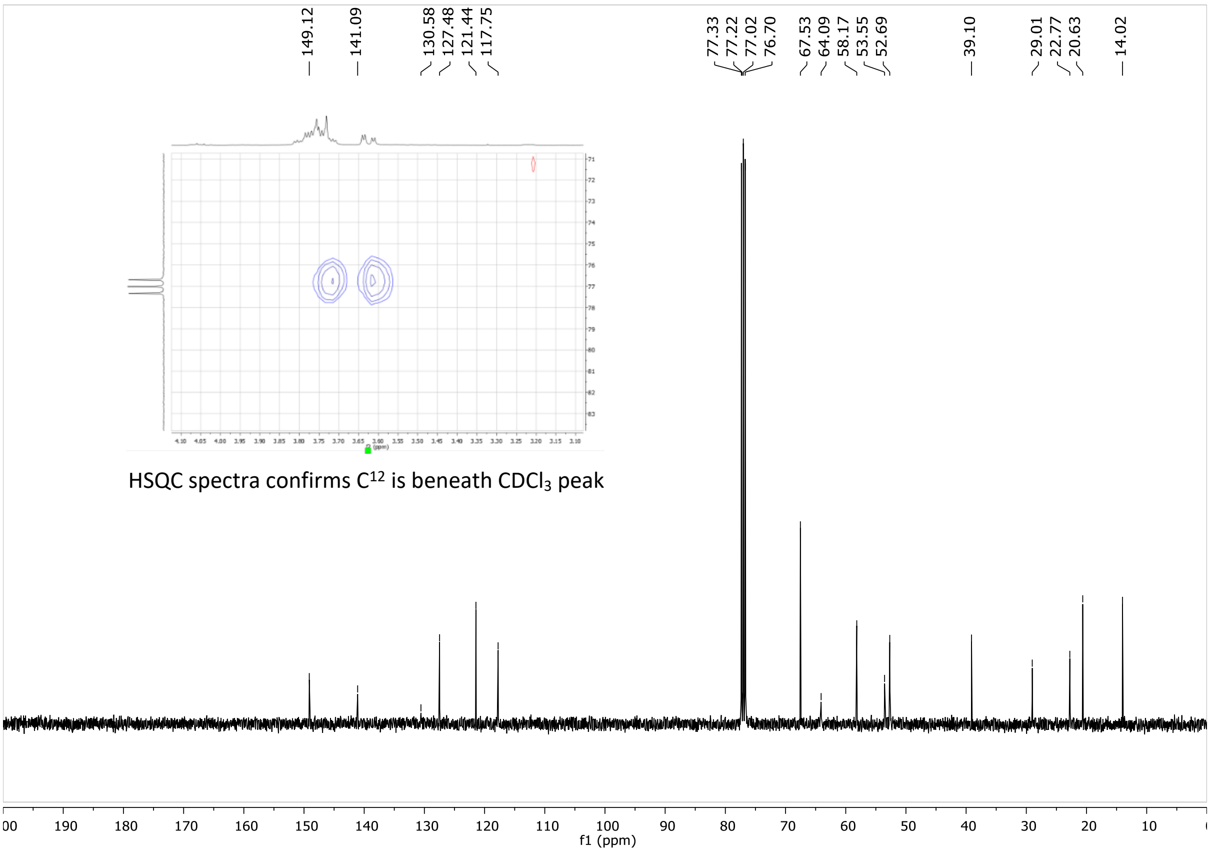
149.12
141.09
130.58
127.48
121.44
117.75
77.33
77.22
77.02
76.70
67.53
64.09
58.17
53.55
52.69
39.10
29.01
22.77
20.63
14.02
HSQC spectra confirms C12 is beneath CDCl3 peak
190 180 170 160 150 140 130 120 110 100 90 80 70 60 50 40 30 20 10
f1 (ppm)

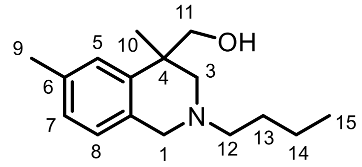
11
OH
9
5
10
4
3
6
N
7
8
1
12
13
14
15

2u

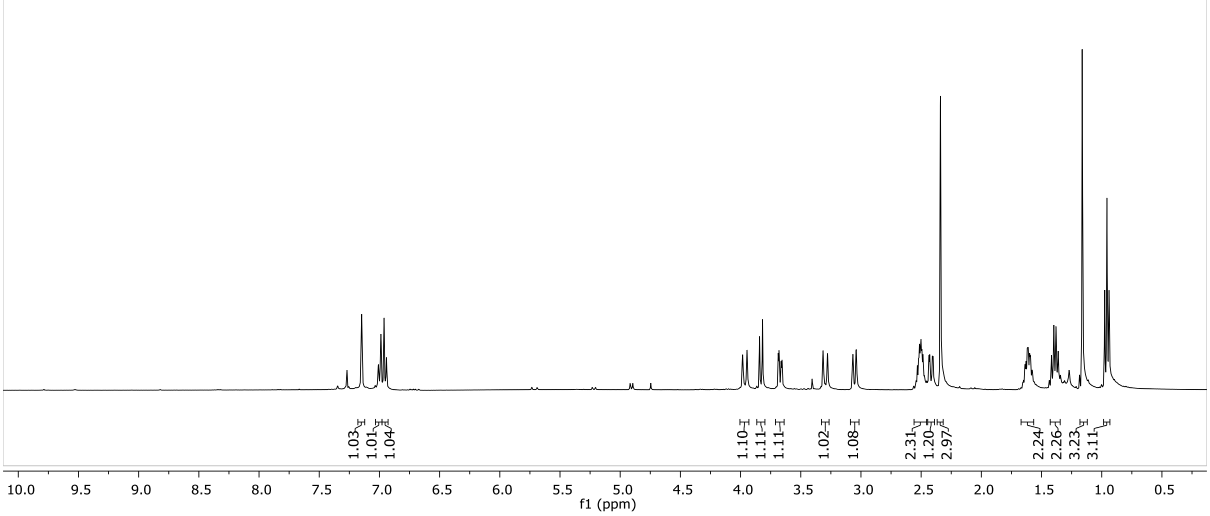
1.03
1.01
1.04
1.10
1.11
1.11
1.02
1.08
2.31
1.20
2.97
2.24
2.26
3.23
3.11
f1 (ppm)

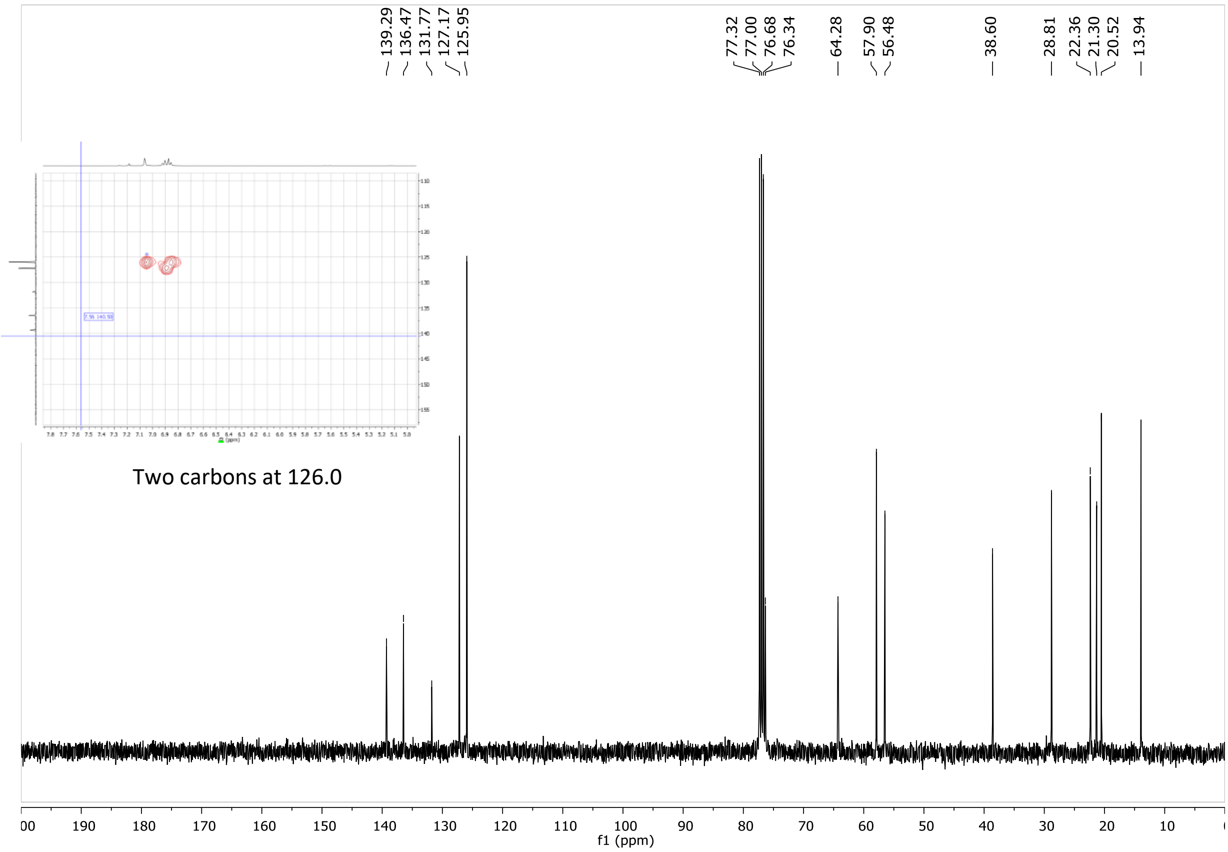
139.29
136.47
131.77
127.17
125.95
77.32
77.00
76.68
76.34
64.28
57.90
56.48
38.60
28.81
22.36
21.30
20.52
13.94
Two carbons at 126.0
f1 (ppm)

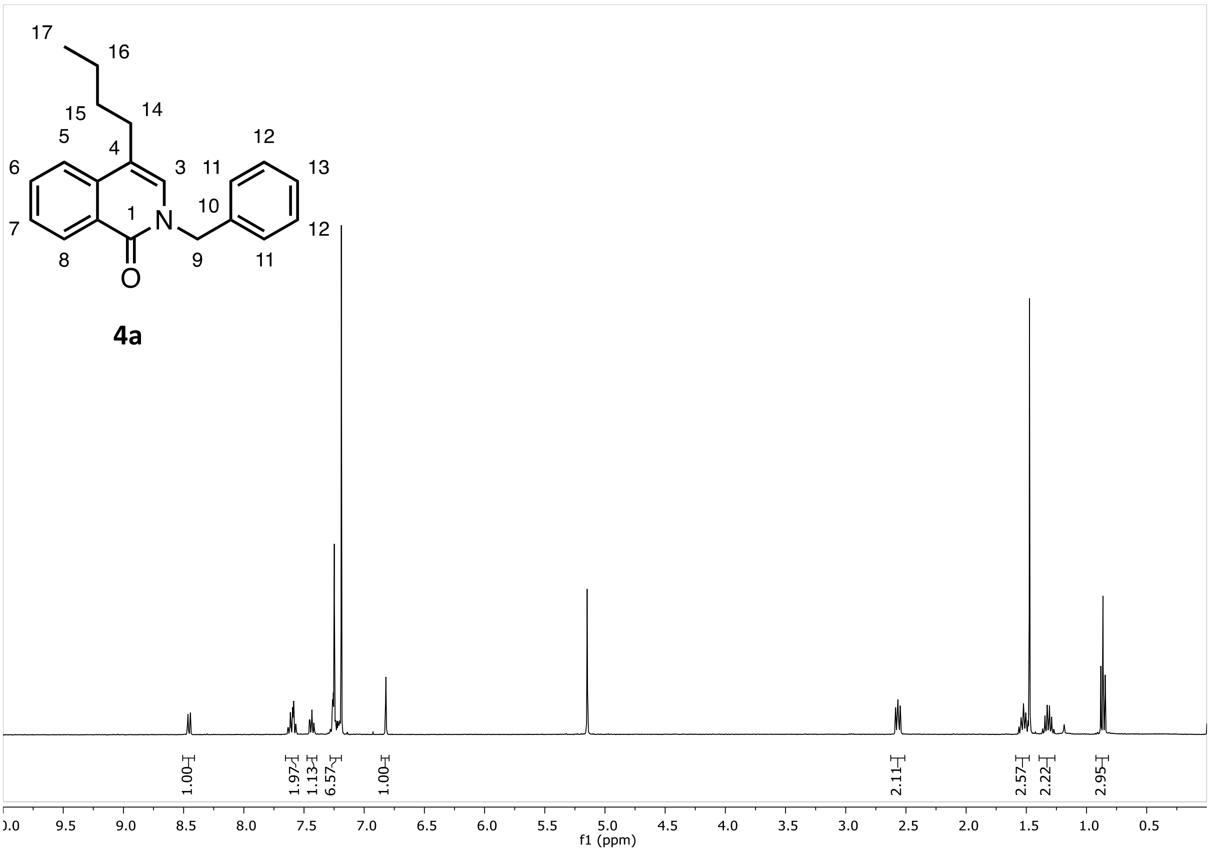
17
16
15
14
5
4
12
6
3
11
13
10
1
N
7
12
8
9
11
O
4a
1.00
1.97
1.13
6.57
1.00
2.11
2.57
2.22
2.95
9.5
9.0
8.5
8.0
7.5
7.0
6.5
6.0
5.5
5.0
4.5
4.0
3.5
3.0
2.5
2.0
1.5
1.0
0.5
f1 (ppm)

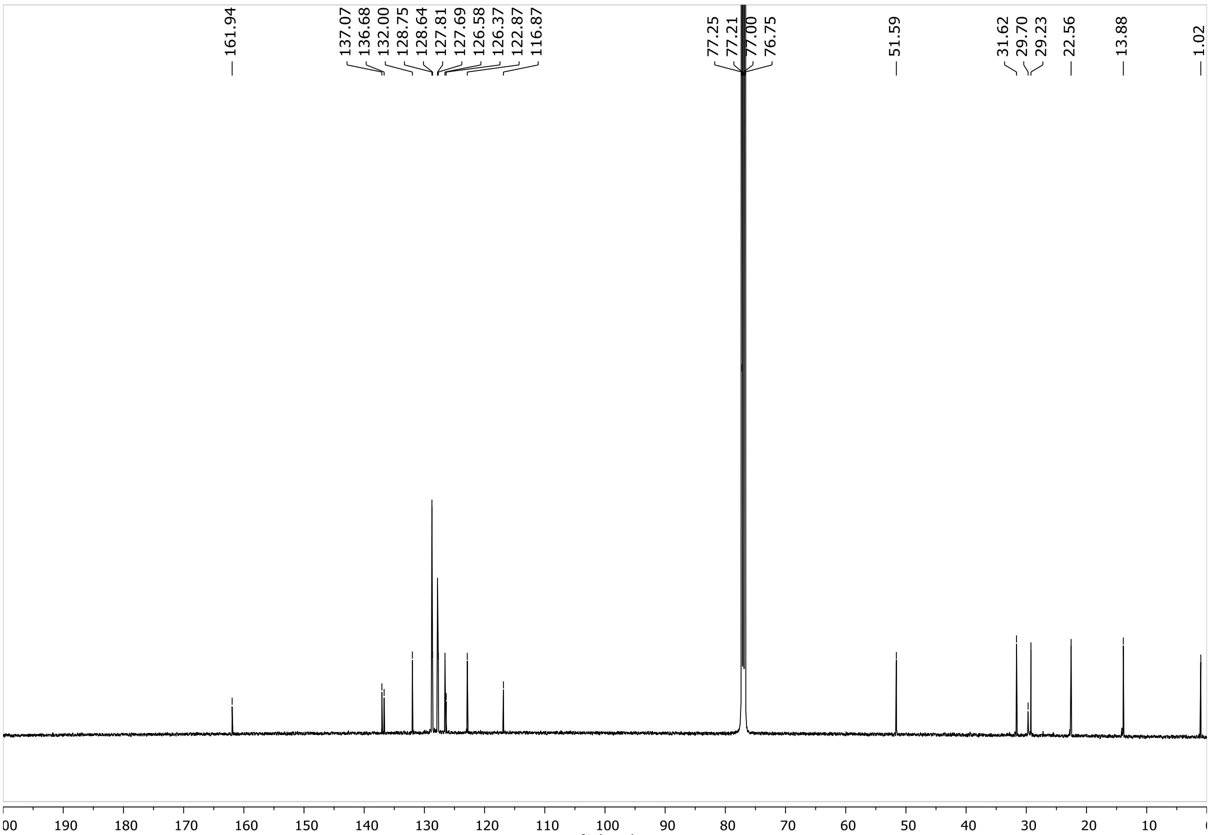
161.94
137.07
136.68
132.00
128.75
128.64
127.81
127.69
126.58
126.37
122.87
116.87
77.25
77.21
77.00
76.75
51.59
31.62
29.70
29.23
22.56
13.88
1.02
190
180
170
160
150
140
130
120
110
100
90
80
70
60
50
40
30
20
10
f1 (ppm)
